# Regioselective C─H Functionalization by the Combination of Enzymatic and Chemocatalytic Reactions in Water

**DOI:** 10.1002/anie.202504378

**Published:** 2025-07-04

**Authors:** Ran Zhu, Xuhua Mo, Tanja Gulder, Tobias A. M. Gulder

**Affiliations:** ^1^ Chair of Technical Biochemistry Department of Chemistry and Food Chemistry Technical University of Dresden Bergstraße 66 Dresden 01069 Germany; ^2^ Biosystems Chemistry, Faculty of Chemistry Technical University of Munich Lichtenbergstraße 4 Garching 85748 Germany; ^3^ Shandong Key Laboratory of Applied Mycology School of Life Science Qingdao Agricultural University Qingdao 266109 China; ^4^ Organic Chemistry – Biomimetic Catalysis Saarland University Saarbrücken 66123 Germany; ^5^ Synthesis of Natural‐Product Derived Drugs Helmholtz Institute for Pharmaceutical Research Saarland (HIPS) Helmholtz Centre for Infection Research (HZI) Saarbrücken 66123 Germany; ^6^ Department of Natural Product Biotechnology Helmholtz Institute for Pharmaceutical Research Saarland (HIPS) Helmholtz Centre for Infection Research (HZI) and Department of Pharmacy at Saarland University PharmaScienceHub (PSH) Campus E8.1 Saarbrücken 66123 Germany

**Keywords:** Acrylic acids, Chemoenzymatic cascades, Enzymatic halogenation, Green chemistry, Micelles

## Abstract

The combination of bio‐ and chemo‐catalytic processes has tremendous potential for the sustainable production of important synthetic building blocks. Such approaches capitalize on the often‐high selectivity of enzymes and the broad arsenal of synthetic transformations to access highly functionalized molecules from simple precursors. However, the strict requirement of enzymes for an aqueous reaction environment, often combined with their lack of stability under harsh reaction conditions, makes the development of efficient chemo‐enzymatic cascade reactions challenging. Within this work, we developed a chemo‐enzymatic platform that combines regioselective enzymatic **H** alogenation, chemo‐catalytic **H** eck coupling, and an enzyme‐catalyzed **H** ydrolysis reaction (H^3^ Catalytic Platform = H^3^CP) in a single reaction vessel. H^3^CP uses water as a reaction solvent to facilitate the enzymatic transformations, with the cross‐coupling reaction taking place in dynamic and protective super‐molecular reaction vessels composed of the commercially available TPGS‐705‐M. Our work utilizes various halogenating enzymes (FDHs and VHPOs), selectively functionalizing diverse substrates, thus enabling subsequent metal‐catalyzed cross‐coupling followed by enzymatic hydrolysis. H^3^CP gives access to a broad range of valuable acrylic acid building blocks with diverse functionalization at the aromatic portion.

## Graphical abstract

H^3^CP contributes to sustainable synthesis by integrating regioselective enzymatic halogenation, Pd‐catalyzed Heck coupling, and enzymatic hydrolysis. TPGS‐705‐M micelles enable the Pd‐catalyzed step, allowing seamless compatibility with enzymatic reactions in one vessel under aqueous conditions. Using this platform, diverse simple aromatic substrates are readily transformed into valuable acrylic acid building blocks.

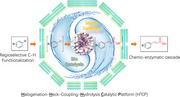

## Introduction

Pursuing sustainable and environmentally friendly chemical processes has increased interest in biocatalysis as a green alternative for producing valuable synthetic building blocks.^[^ ^1^, ^2^ ^]^ Utilizing enzymes to catalyze chemical reactions offers several advantages, including mild reaction conditions, high specificity, and reduced environmental impact.^[^ ^3^ ^]^ Despite these advantages, biocatalysis faces a critical limitation: enzymes are inherently restricted in the reaction types they can catalyze.^[^ ^4^, ^5^ ^]^ Many standard transformations of the chemical synthesis toolbox, particularly those involving carbon–carbon bond formation and other challenging transformations, fall outside the catalytic repertoire of natural enzymes.^[^ ^6^ ^]^ The broader synthetic arsenal, which includes traditional chemical catalysts and reagents, offers a more extensive toolkit for executing various reactions.^[^ ^7^ ^]^ However, these methods often require harsh conditions and toxic solvents and generate substantial waste, making them less sustainable compared to biocatalytic approaches.^[^ ^8^ ^]^ Combining the strengths of biocatalysis and synthetic chemistry presents a promising avenue for achieving greener, more versatile synthetic pathways.^[^ ^9^, ^10^, ^11^ ^]^ Yet, this combination poses significant challenges, mainly due to the different solvent requirements for enzymatic versus chemical reactions. Enzymes typically function optimally in aqueous environments, maintaining their structural integrity and catalytic activity. This requirement poses a major challenge when integrating enzymatic reactions with traditional synthetic methods, which usually require organic solvents incompatible with enzyme stability. Combining these two catalytic systems leads to reduced solubility and activity of enzymes in organic solvents.^[^ ^12^ ^]^ Furthermore, while an enzyme may require a neutral pH and room temperature, a subsequent chemical reaction might demand an acidic environment, elevated temperatures, and higher reactant concentrations. The differences in biocatalysis and synthetic chemistry often diminish the overall efficiency and sustainability of the combined processes and necessitate laborious and time‐consuming adjustments.^[^ ^13^ ^]^

A potential solution to this challenge lies in using reaction micelles, which enable organic reactions to occur in water and allow the selection of suitable reaction conditions for enzymatic transformations. Micelles are aggregates of surfactant molecules that can encapsulate hydrophobic reactants, facilitating their introduction and application in an aqueous medium while leading to high internal reagent concentrations. In addition, micelles can help to prevent the detrimental complexation of chemical reagents, such as transition‐metal catalysts, with the (denatured) protein. The pioneering work of the Lipshutz group has demonstrated the efficacy of micellar catalysis in promoting a wide range of organic reactions under environmentally benign conditions.^[^ ^14^, ^15^, ^16^, ^17^, ^18^ ^]^ The surfactant dl‐α‐tocopherol methoxy polyethylene glycol succinate (TPGS‐750‐M) has demonstrated the capacity to facilitate a multitude of cross‐coupling reactions in an aqueous medium, capitalizing on the ability to achieve high concentrations of reactants within the lipophilic core and providing a hydrophobic inner reactor in water to overcome the solubility and compatibility issues for metal‐catalytic reactions.^[^ ^15^ ^]^ Additionally, substantial research has demonstrated the feasibility of metal‐catalyzed reactions in the aqueous phase in recent years.^[^ ^19^, ^20^, ^21^, ^22^, ^23^ ^]^ Therefore, by enabling organic reactions to proceed in water, micellar systems offer a unique platform for integrating biocatalysis with synthetic chemistry, paving the way for more sustainable and versatile synthetic strategies.^[^ ^24^ ^]^

**Figure 1:**
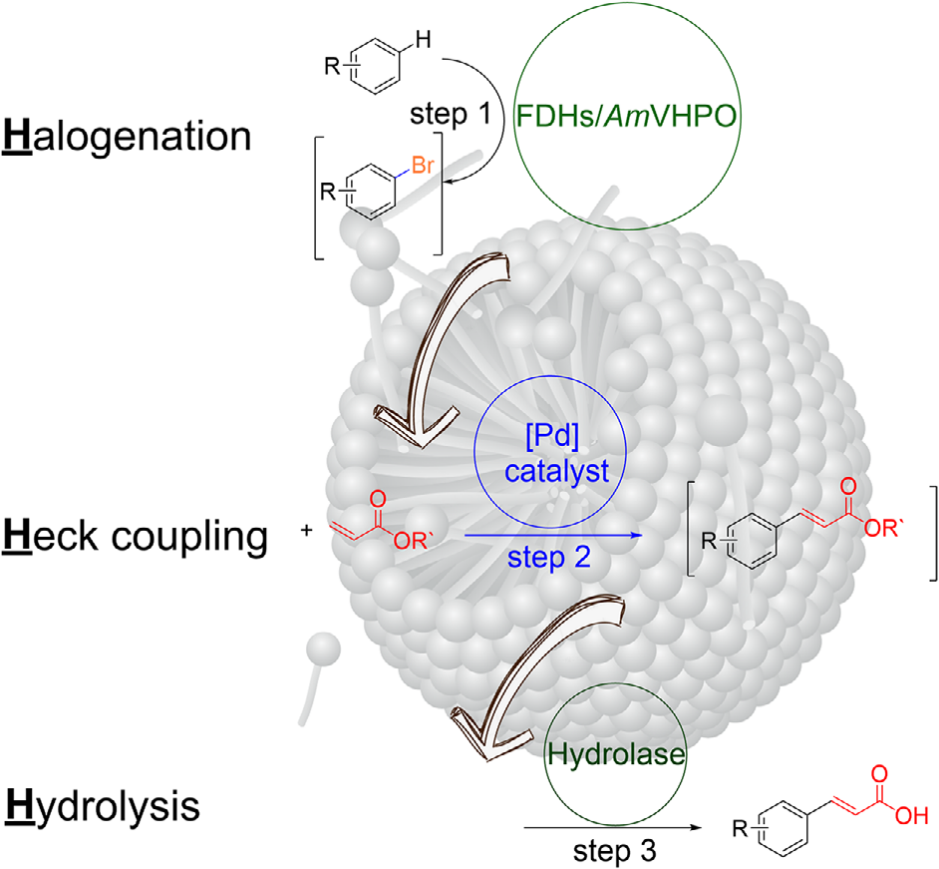
Overview of the Halogenation‐Heck‐Coupling‐Hydrolysis Catalytic Platform (H^3^CP) concept to generate functionalized acrylic acids.

Based on this concept, we developed an approach that combines enzymatic halogenation with Heck coupling and enzymatic hydrolysis in a single process, which we have named H^3^CP (Halogenation‐Heck‐Coupling‐Hydrolysis Catalytic Platform, Figure 1). This method leverages the benefits of micellar catalysis to bridge the gap between the aqueous environment required for enzymatic reactions and the preconditions to perform synthetic chemistry. H^3^CP enables the efficient and sustainable production of functionalized acrylic acids (Figure 1), which are important building blocks in synthesizing pharmaceuticals, agrochemicals, and advanced materials. Functionalized acrylic acids are particularly valuable due to their versatility in forming various carbon–carbon and carbon–heteroatom bonds, making them crucial intermediates in constructing complex molecules.

## Results and Discussion

### Biocatalytic Halogenation

**Figure 2:**
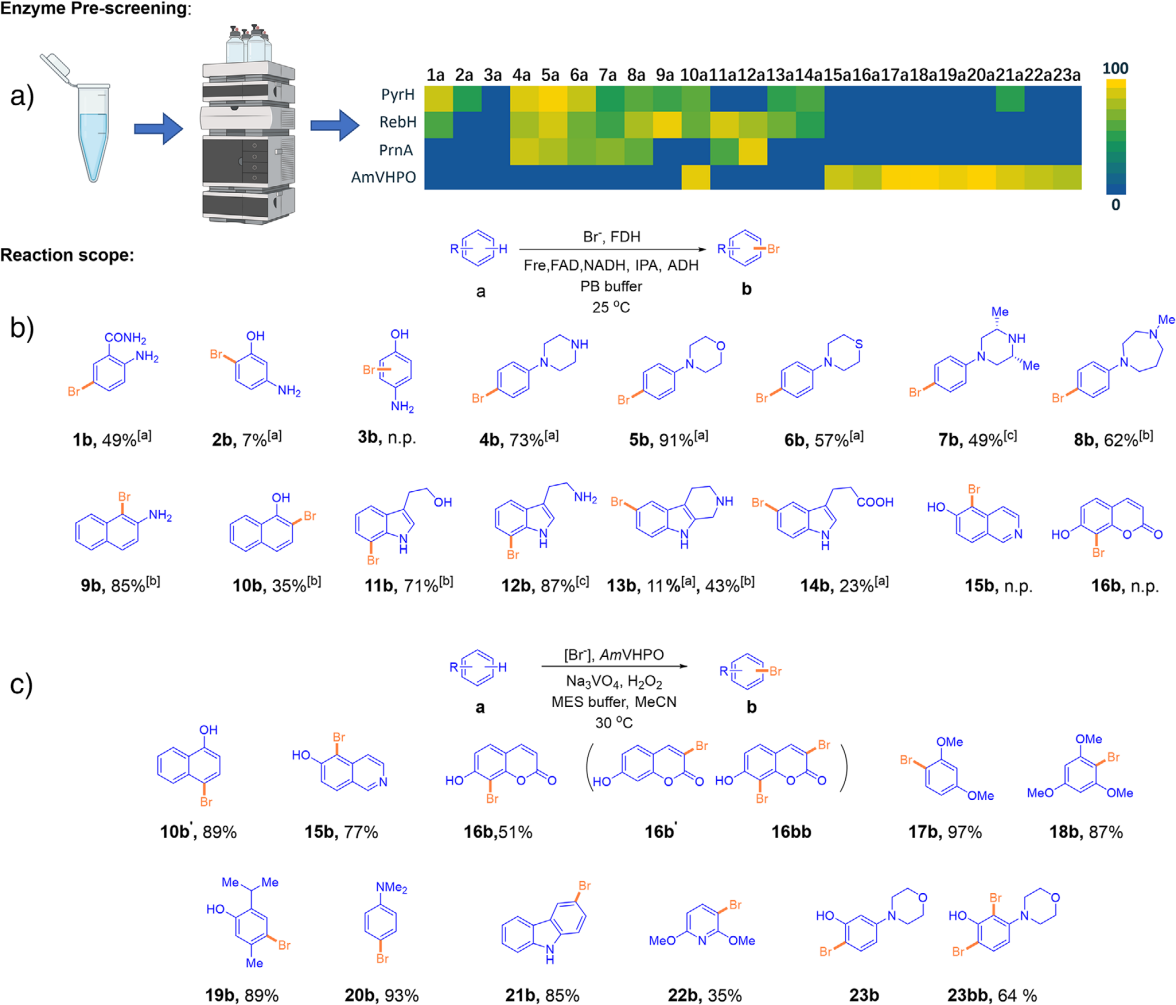
Scope of the regioselective enzymatic halogenation. Biocatalysts include FDHs (^[a]^PyrH, ^[b]^RebH, and ^[c]^PrnA) and a VHPO (***Am*VHPO**). a) Conversion in qualitative enzyme pre‐screening experiments was provided as a heatmap based on consumption of substrates **a** as determined by HPLC. Reaction conditions for FDH‐catalyzed halogenations: substrate **a** (2 mM), NaBr (50 mM), FAD (10 µM), purified FDHs (25 µM), Fre (1 µM), and NADH (20 mM) in PB buffer (pH 7.2) at 25 °C for 8 h. Reaction conditions for VHPOs‐catalyzed halogenations: substrate **a** (2 mM), NaBr (4 mM), *Am*VHPO (3.7 µM pre‐incubated with Na_3_VO_4_), H_2_O_2_ (2 eq, 4 mM), 2‐(*N*‐morpholino) ethanesulfonic acid (MES) buffer (50 mM), in MeCN/water (1/1), at 30 °C for 8 h. b) Determined structures and yields of products based on purified products **b**. Reaction conditions: substrate **a** (3 mM) with IPA (5%), FAD (10 µM), NaBr (50 mM), NADH (200 µM), ADH (1 µM), Fre (1.5 µM), and FDH (35 µM) in PB buffer (pH 7.2) at 25 °C. C) Determined structures and yields of products based on purified products **b**. Reaction conditions: substrate **a** (3 mM), NaBr (4 mM), *Am*VHPO (3 µM, pre‐incubated with Na_3_VO_4_), H_2_O_2_ (1.1 eq, 3.3 mM), MES buffer (50 mM), in MeCN/water (1/2) at 30 °C overnight. Experimental details are provided in the Supporting Information.

The regioselective biocatalytic introduction of bromide enabling downstream cross‐coupling reactions was initially investigated using flavin adenine dinucleotide (FAD)‐dependent halogenases (FDHs).^[^ ^25^, ^26^, ^27^, ^28^ ^]^ We selected three FDHs (RebH, PrnA, and PyrH)^[^ ^29^, ^30^, ^31^ ^]^ that were previously shown to have good enzymatic activity and stability and to perform on various substrates regioselectively.^[^ ^26^, ^32^ ^]^ The enzymes were recombinantly produced in *E. coli* and purified by affinity chromatography on Ni‐NTA beads (see Supporting Information). Reactions were performed in sodium phosphate buffer (PB buffer) at pH = 7.2 supplemented with NADH, FAD, and NaBr as the halide source. Initial test runs were qualitatively evaluated by HPLC analysis (Figure 2a) based on individual substrate consumption across 23 different substrates **a**. These investigations revealed distinct substrate preferences of the three FDHs, with PyrH best performing on **1a**, **2a**, **4a**–**6a**, and **14a**; PrnA on **7a** and **12a**; and RebH on **8a**–**11a** and **13a**. Substrates **3b** and **15a**–**23a** were not accepted by any of the tested enzymes.

Based on these initial qualitative analyses, preparative experiments were conducted using the best‐performing FDHs for each individual substrate **1a**–**14a** to enable product isolation, structural characterization, and determination of isolated yields (Figure 2b; for methods, see Supporting Information). For these experiments, an NADH cofactor regeneration system consisting of sacrificial isopropyl alcohol (IPA) and alcohol dehydrogenase (ADH) was employed to prevent the need for over‐stoichiometric use of NADH.^[^ ^33^ ^]^ As expected from the pre‐screening, **3a** delivered no product, even at a 3 mM reaction scale. All other desired compounds were readily produced using PyrH (**1b**: 49%; **2b**: 7%; **4b**: 73%; **5b**: 91%; **6b**: 57%; **14b**: 23%), PrnA (**7b**: 49%; **12b**: 89%), and RebH (**8b**: 62%; **9b**: 95%; **10b**: 35%; **11b**: 71%; **13b**: 43%).

We included a vanadium‐dependent haloperoxidase (VHPO) in our screening to further expand the substrate scope to **15a**–**23a**. We selected *Am*VHPO from the cyanobacterium *Acaryochloris marina* as a proven catalytically competent halogenation enzyme.^[^ ^34^, ^35^, ^36^, ^37^ ^]^ Unlike the FAD‐based mechanism of FDHs, substrate bromination by *Am*VHPOs involves the oxidation of halide at the vanadate cofactor, forming an electrophilic bromine species that subsequently reacts with aromatic substrates in an electrophilic substitution reaction (S_E_Ar).^[^ ^38^ ^]^ The pET28‐*Am*VHPO vector was transformed into *E. coli* (DE3) for recombinant protein production and enabled the isolation of more than 30 mg L^−1^ soluble biocatalyst (see Supporting Information). The purified enzyme was preincubated with orthovanadate salt Na_3_VO_4_ before its application on substrates **10a** and **15a**–**23a** in MES buffer at pH 6.0 supplemented with NaBr as the halide source, leading to significant formation of the desired products in most reactions (Figure 2c). Substrates **15a** and **16a** delivered products **15b** and **16b** in 77% and 51% yield, respectively. However, using substrate **16a**, the regioisomeric side product **16b′** and di‐brominated **16bb** were also obtained in minute amounts. *Am*VHPO also showed the desired selective mono‐bromination of anisoles **17a** and **18a** to the respective *ortho*‐brominated products **17b** (97%) and **18b** (87%). For substituted phenols and anilines **10a** and **19a**–**20a**, the enzyme showed high regioselectivity to form *para*‐brominated products **10b′** (89%), **19b** (89%), and **20b** (93%). Heterocyclic products **21b** (85%) and **22b** (35%) were also obtained with no dibromination observed. In contrast, for phenol **23a**, only the di‐brominated **23bb** (64%) was isolated. Despite shortened reaction times and continuous monitoring, the predominant products remained dihalogenated.

### Heck Coupling Optimization

After establishing conditions for the enzymatic halogenation step, we investigated suitable and mild reaction conditions for the subsequent Heck coupling in water, applying TPGS‐750‐M micelles as in situ reaction vessels. Therefore, we initially optimized the conditions of the Heck coupling step. Using 1‐bromo‐2,4‐dimethoxybenzene (**17b**) as the initial test substrate (Table 1), we systematically investigated the influence of various additives, reaction times, and reactant ratios on the yield. Table 1 shows no coupling reaction was observed when trimethylamine was the only additive (entry 1). When additionally adding salts at 3 M concentration, conversion to the desired product **17c** was observed, with increasing yields from Na_2_CO_3_ (entry 2, 76%) over K_2_CO_3_ (entry 3, 81%) to NaCl (entry 4, 95%). Salts have been shown to impact the swelling of the SDS micelles,^[^ ^39^ ^]^ affecting the reaction yields.^[^ ^40^ ^]^ Decreasing (1 M) or increasing (4 M) NaCl salt concentration led to strongly (27%) or slightly (93%) decreased yields, respectively (entries 5 and 6). Raising the amount of Pd from 2% to 5% using the determined optimal additive composition led to a comparable yield (entry 7, 95%). Extension of the reaction time from 12 to 18 h (entry 8, 98%) had a slightly positive impact on product yield, while further prolonging reaction time did not further influence the outcome (entries 9 to 11). Finally, the amount of the coupling partner, acrylate **A**, could be reduced to 1.5 equivalents without negatively affecting product yield (entry 12, 98%). Therefore, these optimized conditions (entry 12) were used in all subsequent Heck coupling reactions.

**Table 1:** Screening of reaction conditions for Heck coupling.^a)^

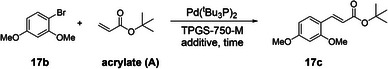			
Entry	Additive	Time (h)	Yield (%)
1	Et_3_N	12	–
2	Et_3_N + 3 M Na_2_CO_3_	12	76
3	Et_3_N + 3 M K_2_CO_3_	12	81
4	Et_3_N + 3 M NaCl	12	95
5	Et_3_N + 1 M NaCl	12	27
6	Et_3_N + 4 M NaCl	12	93
7^b)^	Et_3_N + 3 M NaCl	12	95
8	Et_3_N + 3 M NaCl	18	98
9	Et_3_N + 3 M NaCl	24	94
10	Et_3_N + 3 M NaCl	30	94
11	Et_3_N + 3 M NaCl	36	98
12^c)^	Et_3_N + 3 M NaCl	18	98

^a)^ Reaction conditions: 2 mol% Pd(^t^Bu_3_P)_2_, 1‐bromo‐2,4‐dimethoxybenzene (0.1 mmol), butyl acrylate, Et_3_N (0.3 mmol), NaCl, and 1 mL 5 wt% TPGS‐750‐M in H_2_O under inert atmosphere at 40 ^°^C.

^b)^ Reaction with 5 mol% Pd(^t^Bu_3_P)_2._

^c)^ Reaction with 1.5 eq. of butyl acrylate **A**. Yields were determined by product isolation. Et_3_N = triethylamine; TPGS‐750‐M = dl‐α‐tocopherol‐methoxypolyethylenglykol‐succinate solution.

### Combination of Enzymatic Halogenation and Heck Coupling

**Scheme 1:**
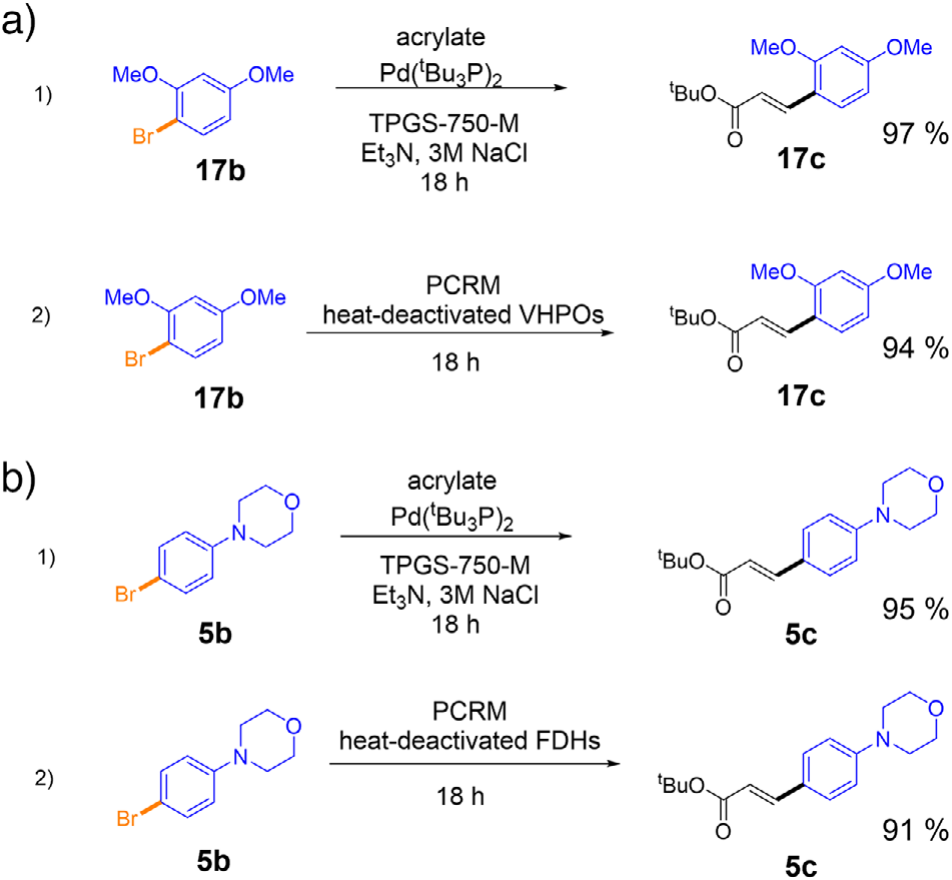
Effect of residues of the halogenation reactions on subsequent Heck coupling using PCRM (*tert*‐butyl acrylate (0.15 mmol), triethylamine (0.3 mmol), in 1 mL 5 wt% TPGS‐750‐M, 2% Pd catalyst). Comparison of optimized Heck coupling conditions (1; Table 1, entry 12) to PCRM‐based catalysis mimicking the reaction environment of enzymatic halogenation reactions with heat‐deactivated halogenase (2) at 0.1 mmol scale. a) Transformation of **17b**–**17c** with heat‐deactivated halogenase *Am*VHPO. b) Transformation of **5b**–**5c** with heat‐deactivated halogenase PyrH.

Next, we aimed to integrate the enzyme‐catalyzed halogenation reaction with the metal‐catalyzed coupling reaction to synthesize the desired coupling products in one pot. To achieve this goal, applying prefabricated catalyst‐reactant mixtures (PCRM), composed of the micelle and reagents required for the Heck coupling in the aqueous phase, would permit straightforward reaction handling.^[^ ^41^ ^]^ It was therefore assessed whether the aqueous‐phase metal‐catalyzed coupling reactions using PCRM work comparably well as the optimized aqueous Heck reactions conditions outlined above and if this system tolerates the presence of residuals (e.g., buffer, cofactors, enzymes) from the previous halogenation reactions. Using **17b** as the test substrate, we thus compared a clean test reaction using the defined conditions as optimized above to a reaction containing all components of the enzymatic halogenation step with thermally inactivated enzyme *Am*VHPO, thus mimicking the environment of a sequential two‐step, one‐pot reaction. To our delight, the reaction yields remained largely unaffected, with 97% versus 94% isolated yield of **17c** for clean versus complex reaction conditions, respectively (Scheme 1a). Similar results were obtained with substrate **5b** when using FDH PyrH (95% versus 91% yield). Therefore, adding inactivated enzymes and buffer components in the simulated reaction did not significantly decrease yield. (Scheme 1b).

**Figure 3:**
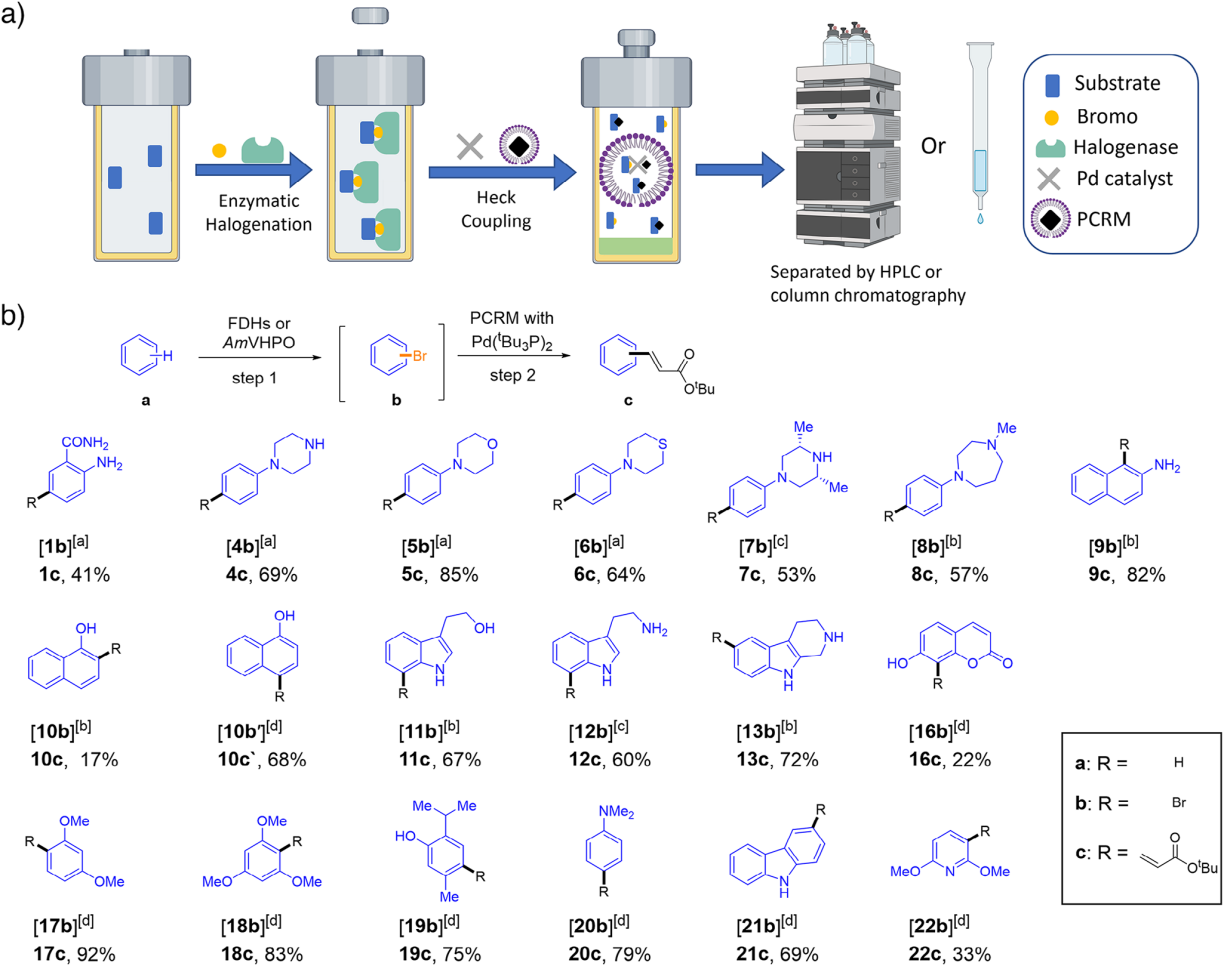
Combination of enzymatic halogenation and Heck coupling in water. a) Schematic overview of the integrated enzyme‐catalyzed halogenation and metal‐catalyzed coupling in one pot. b) Tested substrate scope. Previously optimized reaction conditions for the halogenation step (step 1, using FDHs: ^[a]^PyrH, ^[b]^RebH, ^[c]^PrnA; VHPOs: ^[d]^ *Am*VHPO) and the cross‐coupling step were integrated. Reaction conditions for FDH‐catalyzed halogenations in **step 1**: substrate **a** (3.5 mM) in 5% IPA, FAD (10 µM), NaBr (50 mM), NADH (200 µM), ADH (1 µM), purified Fre (1.5 µM), and halogenase FDH (40 µM) in PB buffer (pH 7.2), with shaking at 300 rpm at 25 °C. Reaction conditions for *Am*VHPO‐catalyzed halogenations in **step 1**: Substrate **a** (3.5 mM), NaBr (4 mM), *Am*VHPO (3.5 µM, preincubated with Na_3_VO_4_) in MES buffer (pH 6.0, 50 mM) in acetonitrile (ACN)/H_2_O (1/2) with shaking at 300 rpm at 30 °C. **Step 2**: addition of PCRM (NaCl (3 M) in 5 wt% TPGS‐750‐M/H_2_O (1 mL), 5%(v) THF, 15%(v) Et_3_N, *tert*‐butyl acrylate (1.5 eq.), and Pd (^t^Bu_3_P)_2_ (2%)) and stirring of the reaction at 40 °C for 18 h. Yields refer to isolated material. For experimental details, see Supporting Information.

Based on these preliminary experiments, the enzymatic halogenation step and the subsequent metal‐catalyzed coupling reaction should be directly combinable in the same pot, thus preventing intermediate purification steps and thereby enhancing overall reaction efficiency. After completing the biocatalytic transformation of starting materials **a** to brominated intermediates **b**, the enzyme can be deactivated by briefly heating the reaction mixture, and the reaction solution can directly be supplemented with the PCRM and the metal catalyst to deliver the desired products **c** (Figure 3a). By applying this method, we converted a selection of the 19 best‐performing substrates from the halogenation reaction screening (cf. Figure 2). Concerning the reactions initiated by FDH‐catalyzed bromination, aniline substrate **1a** was transformed into acrylate **1c** with an isolated yield of 41%. Substrates **4a**–**8a** delivered good to excellent isolated yields of the corresponding acrylates **4c**–**8c** (53%–85%). Functionalization of 2‐naphthylamine **9a** and 1‐naphthol **10a** resulted in naphthylamine‐acrylat **9c** (82%) and **10c** (17%). Concerning indole substrates **11a**–**13a**, generally good yields of acrylates **11c**–**13c** (60%–72%) were observed (Figure 3b).

For substrates **10a** and **16a–22a**, the enzyme catalyst was switched from FDHs to *Am*VHPO. Product **10c′** was obtained in 68% isolated yield as *para*‐substituted acrylate, thus delivering an alternative product compared to the FDH‐catalyzed formation of **10c**. The hydroxy‐substituted heterocyclic compound **16c** was formed in a low yield of 22% (formation of oxo‐addition by‐product). For anisole substrates **17a** and **18a**, the corresponding products **17c** and **18c** were obtained in excellent yields (83%–92%). Substrates **19a–21a** all furnished acrylates **19c–21c** in good isolated yields of 69%–79%. Acrylated pyridine **22c** was obtained in an isolated yield of 33% from **22a**. In all cases, the desired acrylates were isolated as single regioisomers. This demonstrates the strict selectivities of the employed halogenation enzymes and the broad utility of the developed combination of halogenation and cross‐coupling reactions.

### Combined Halogenation‐Heck Coupling‐Hydrolysis Catalytic Platform (H^3^CP)

To prepare for the integration of the final enzymatic ester saponification step, suitable conditions for the hydrolysis of the ^t^Bu ester of the acrylic acid analogs had to be evaluated. The enzyme of choice for this transformation was pig liver esterase (PLE), which is commonly used in preparative organic chemistry and typically employed as a catalyst in aqueous solution with broad applicability.^[^ ^42^ ^]^

**Figure 4:**
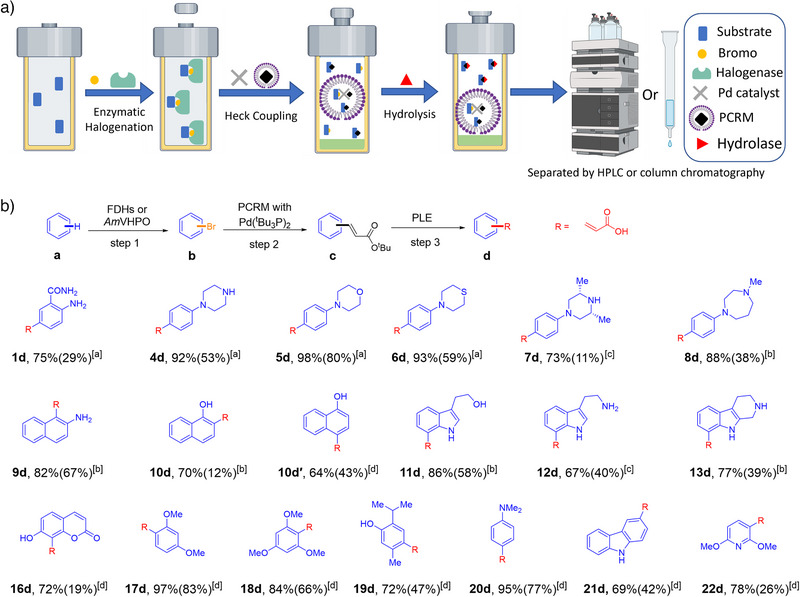
Halogenation‐Heck‐Coupling‐Hydrolysis Catalytic Platform (H^3^CP) tests. a) Conception of enzymatic halogenation, metal‐catalyzed coupling, and enzymatic hydrolysis in one pot. The starting material (SM) in the reactor undergoes enzymatic halogenation to form the brominated intermediate. Subsequently, it reacts with the PCRM introduced into the same reactor to produce the acrylate ester in the micelle. Final enzymatic saponification delivers acrylic acids. b) Halogenation‐Heck‐coupling‐hydrolysis cascade reactions. The conversion of the hydrolysis step was determined upon consumption of intermediate **c** by HPLC. Yield from **a** to **d** of the cascade reaction in brackets corresponds to isolated yields over the entire cascade. In the halogenation step (step 1), different halogenating enzymes are used as biocatalysts (halogenases [FDHs]: [a]PyrH, [b]RebH, [c]PrnA; haloperoxidases: [VHPOs]: [d]*Am*VHPO). Reaction conditions for FDH‐catalyzed halogenation in **step 1**: Substrate **a** (3.5 mM) in 5% IPA, FAD (10 µM), NaBr (50 mM), NADH (200 µM), ADH (1 µM), purified Fre (1.5 µM), and halogenase FDH (40 µM) in PB buffer (pH 7.2), with shaking at 300 rpm at 25 °C. Reaction conditions for *Am*VHPO‐catalyzed halogenations in **step 1**: Substrate a (3.5 mM), NaBr (4 mM), *Am*VHPO (3.5 µM, preincubated with Na_3_VO_4_), in MES buffer (pH 6.0, 50 mM) in ACN/H_2_O (1/2) with shaking at 300 rpm at 30 °C. **Step 2**: addition of PCRM (NaCl (3 M) in 5 wt% TPGS‐750‐M/H_2_O (1 mL), 5%(v) THF, 15%(v) Et_3_N, *tert*‐butyl acrylate (1.5 eq.), and Pd (^t^Bu_3_P)_2_ (2%)) and stirring of the reaction at 40 °C for 18 h. **Step 3**: addition of PB buffer (pH 6.6), 5% ^t^BuOH (5% v) and PLE (100 units), and stirring of the reaction at 25 °C for 18 h. 2). Yields refer to isolated products. Methods are detailed in the Supporting Information.

We first added the PCRM to the enzymatic saponification reaction to test compatibility with the previous reaction conditions. Treatment of substrate **17b** with PLE at room temperature in 4‐(2‐hydroxyethyl)‐1‐piperazineethanesulfonic acid buffer (HEPES buffer, pH 7.5) directly delivered yields of 68% of the desired acid **17d** (Table 2, entry 1). It was imperative to neutralize the Heck reaction mixture before introducing the enzyme for the subsequent step.^[^ ^14^ ^]^ To enhance the performance of this step, PB buffer (pH 6.6) was used instead of HEPES, which led to an increased product yield of 79% (entry 2). Significantly elevating (60 °C, entry 3) or lowering (0 °C, entry 4) the reaction temperature led to a dramatic reduction (9%–27%) of the reaction yield. However, product yield was slightly increased when running the reaction at 40 °C (81%, entry 5). Changing the overall reaction environment to be slightly more basic (PB buffer, pH 7.4) or acidic (PB buffer, pH 5.8) led to less favorable reaction outcomes (entries 6 and 7). The addition of ^t^BuOH (20%)^[^ ^43^ ^]^ as a co‐solvent, however, did significantly enhance product yield (entry 8, 91%). This effect was maintained even at lower concentrations of ^t^BuOH of 10% (89% yield) or 5% (91% yield). Using the conditions elaborated above (entry 10), we next utilized the H^3^CP methodology on all substrates that were successfully transformed in the halogenation‐Heck coupling sequence outlined above, aiming at a one‐pot conversion of substrates **a** via brominated compounds **b** to acrylic acid esters **c** to finally deliver the corresponding free acids **d** (Figure 4a). The measured substrate conversions were good to excellent for all tested substrates **1**, **4–11**, **13**, and **16–22**, ranging from 64% to 98% (Figure 4b). Isolated yields for most substrates ranged from 38% to 83%, with lower‐yielding exceptions being aniline **1d** (29%), piperazine **7d** (11%), naphthol **10d** (12%), coumarin **16d** (19%), and pyridine **22d** (26%).

**Table 2:** Optimization of the reaction conditions for the Heck coupling‐hydrolysis cascade.^a)^

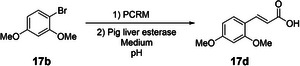					
Entry	Medium	T (°C)	pH value	Additive	Yield (%)
**1**	HEPES buffer	25	7.5	–	68
**2**	PB buffer	25	6.6	–	79
**3**	PB buffer	60	6.6	–	27
**4**	PB buffer	0	6.6	–	9
**5**	PB buffer	40	6.6	–	81
**6**	PB buffer	40	7.4	–	37
**7**	PB buffer	40	5.8	–	49
**8**	PB buffer	40	6.6	^t^BuOH(20%v)	91
**9**	PB buffer	40	6.6	^t^BuOH(10%v)	89
**10**	PB buffer	40	6.6	^t^BuOH(5%v)	91

^a)^ Yields were determined by isolation; reaction conditions: 1) **17b** (0.1 mmol), PCRM (*tert*‐butyl acrylate (0.15 mmol), triethylamine (0.3 mmol), in 1 mL 5 wt% TPGS‐750‐M, 2% Pd catalyst), at 40 ^°^C for 18 h. 2) addition of pig liver esterase (100 units) and buffer (1 mL).

### Upscaling of the Cascade Reaction Employing the H^3^CP Approach

Next, we aimed to scale H^3^CP to 1 mmol substrate to allow the synthesis of preparatively useful amounts of the desired acrylic acids. During initial tests, we realized that product yields at larger scales were significantly reduced, primarily because of lower performance during the enzymatic halogenation step due to insufficient stability of the halogenating enzymes. Therefore, in the upscaled reactions, portion‐wise addition of the enzymes appeared promising. In addition, it has to be taken into account that the halogenating enzymes employed are not commercially available and thus need to be prepared by recombinant production and purification at a larger scale. We hence targeted two alternative approaches that have the potential to resolve these challenges: the application of cross‐linked enzyme aggregates (CLEAs)^[^ ^44^ ^]^ and the encapsulation of enzymes in tubings with molecular‐weight cutoff (MWCO) membranes.

**Figure 5:**
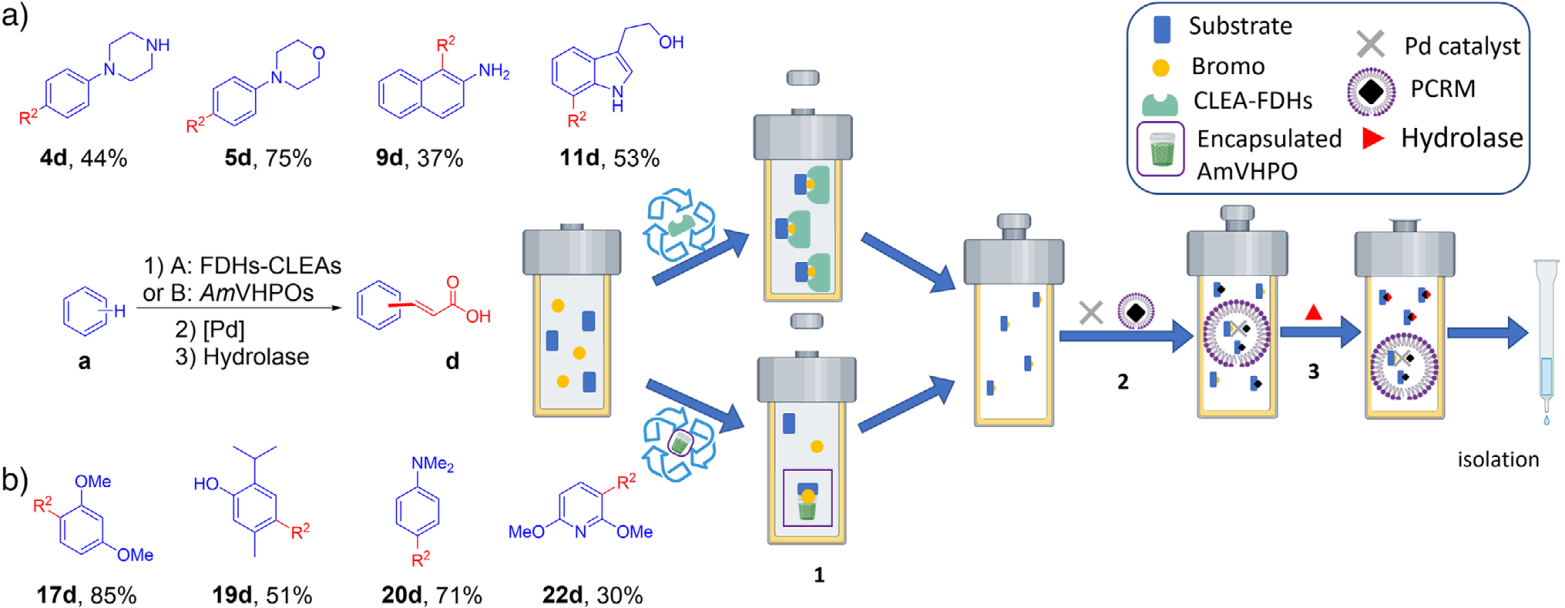
Scaling of the H^3^CP approach. Three reactions integrated to transform substrates **a** to desired acids **d**. **Step 1**: a) FDH‐CLEAs (prepared from 1.5 L *E. coli* cultures) as catalyst for bromination of substrate **a** (2 mM, 1 mmol), FAD (10 µM), NaBr (50 mM), NADH (200 µM), and PB buffer (20 mM, pH 7.2) with 5% IPA (v/v) in a total volume of 500 mL. The reaction was shaken at 200 rpm at 25 °C. b) *Am*VHPO (prepared from 3 L *E. coli* cultures) encapsulated in 10 kDa MWCO tubing as a catalyst for bromination of substrate **a** (3 mM, 1 mmol), NaBr (3.3 mM), and Na_3_VO_4_ (185 µM) dissolved in 330 mL MES buffer (pH 6.0, 50 mM) in ACN/H_2_O (1/2). The reaction was shaken at 300 rpm at 30 °C for 48 h, and H_2_O_2_ (1.1 eq, 3.3 mM) was added five times every 8 h. **Step 2**: Addition of PCRM (NaCl (3 M) in 5 wt% TPGS‐750‐M/H_2_O (10 mL), 5%(v) THF, 15%(v) Et_3_N, *tert*‐butyl acrylate (1.5 eq.), and Pd (^t^Bu_3_P)_2_ (2%)) and stirring of reaction mixture at 40 °C for 24 h. **Step 3**: Addition of PB buffer (pH 6.6) with 5% ^t^BuOH (v/v), PLE (1000 units, 55 mg), and stirring of the reaction mixture at 25 °C for 48 h. Yields refer to isolated products. Methods are detailed in the Supporting Information.

The CLEA approach was exemplarily applied to the FDH enzymes. Enzyme cross‐linking was directly performed on lysed cell pellets of the recombinant FDH production host, thus omitting enzyme purification, mixed with reductase and ADH. Cross‐linking was achieved using (NH_4_)_2_SO_4_ and glutaraldehyde (see Supporting Information). The obtained CLEAs were stored at 4 °C until further use. Immobilized FDH‐CLEAs were added to the reaction mixture in the course of the reaction in equal portions, ensuring the availability of fresh, active enzymes throughout the reaction course. Upon completing the enzymatic bromination step, CLEAs were removed by centrifugation (10 000 rpm, 20 min). The recovered CLEAs were reactivated for further use by washing with PB buffer (pH 7.4), leading to more than 90% restored enzyme activity even after application in three reaction cycles (see Supporting Information). Using this system for the initial halogenation step, the four substrates **4a**, **5a**, **9a**, and **11a** were exemplarily functionalized using H^3^CP at a 1 mM scale. This indeed readily delivered the desired acrylic acids **4d** (44% isolated yield, 102.2 mg), **5d** (75%, 174.9 mg), **9d** (37%, 78.9 mg), and **11d** (53%, 122.6 mg) (Figure 5a).

The molecular weight cutoff (MWCO) tubing approach was tested for *Am*VHPO‐catalyzed transformations. We employed a semipermeable membrane with an MWCO of 10 kDa that efficiently retains the biocatalyst (MW of 73.13 kDa) while allowing all substrates, cofactors, and products to pass through. Similar to the step‐wise addition of CLEAs outlined above, we regularly exchanged the enzyme‐loaded tubings every 8 h during the enzymatic bromination reaction. Enzyme regeneration in used tubings was achieved by washing withtris(hydroxymethyl)aminomethane buffer (tris‐buffer), leading to up to 100% retained activity due to the notable stability of the *Am*VHPO.^[^ ^34^ ^]^ *Am*VHPO‐initiated acrylate synthesis at 1 mM scale was exemplarily tested for substrates **17a**, **19a**, **20a**, and **22a**, in all cases successfully delivering the desired acrylates **17d** (85%, 177.6 mg), **19d** (51%, 112.3 mg), **20d** (71%, 135.8 mg), and **22d** (30%, 62.8 mg). (Figure 5b).

## Conclusion

Cascade reactions combining enzymatic and small‐molecule catalysis steps are powerful tools for building complex molecules in an economically viable and environmentally sustainable way. Therefore, it is not surprising that they have lately garnered significant interest from the synthetic community. Halogenation‐triggered multistep reactions are advantageous for a manifold of different transformations, such as C,C‐bond formations. Especially, biohalogenation‐cross‐coupling chemistry offers improved step economics and selectivities while reducing resources compared to conventional organic synthesis.^[^ ^10^, ^11^, ^25^, ^45^, ^46^, ^47^, ^48^ ^]^ It is thus not surprising that in the last decade important contributions from different groups were witnessed, particularly involving flavin‐dependent halogenases.^[^ ^49^, ^50^, ^51^, ^52^, ^53^ ^]^

Our study showcases a streamlined approach to combining biocatalysis and synthetic chemistry through the development of the H^3^CP (Halogenation‐Heck Coupling‐Hydrolysis Catalytic Platform), which integrates two types of regioselective enzyme‐catalyzed halogenations, a palladium‐catalyzed Heck coupling, and an enzymatic hydrolysis catalyzed by pig liver esterase. This combined approach efficiently converts simple aromatic scaffolds, such as anilines, anisoles, and indoles, into selectively functionalized acrylic acids.

To address the challenges of reaction incompatibility between enzymatic and chemical steps, micelles formed using TPGS‐750‐M were employed. This micellar system creates a reaction environment that effectively separates enzymatic and synthetic steps, enhancing the overall efficiency of the cascade. The pre‐incubation strategy allowed the formation of a reactant mixture, PCRM, which nicely enables the integration of metal‐catalyzed and enzymatic reactions. Using CLEAs for FDHs and encapsulated *Am*VHPO helped to increase biocatalyst efficiency, enabling lower catalyst loading, minimizing cross‐reaction interference, and supporting sustained use by recycling.

In summary, the H^3^CP presented here represents a significant advancement in C─H activation and modification chemistry,^[^ ^32^ ^]^ demonstrating the potential of combining biocatalysis and synthetic metal catalysis in a single process. By operating under mild, aqueous conditions and by effectively managing the distinct requirements of bio‐ and metal‐catalyzed steps, this approach provides a new framework for developing versatile and sustainable chemo‐enzymatic cascades. This study underscores the value of micellar catalysis in reconciling the challenges of mixed catalysis, contributing to paving the way for future innovations in sustainable chemical synthesis.

## Supporting Information

The authors have cited additional references within the Supporting Information.^[^ ^52^, ^54^, ^55^, ^56^, ^57^, ^58^, ^59^, ^60^, ^61^, ^62^, ^63^, ^64^, ^65^, ^66^ ^]^

## Conflict of Interests

The authors declare no conflict of interest.

## Supporting information



Supporting Information

## Data Availability

The data that support the findings of this study are available in the Supporting Information of this article.
