# Supplementary material for: Regioselective C─H Functionalization by the Combination of Enzymatic and Chemocatalytic Reactions in Water
Source: Angew Chem Int Ed Engl. 2025 Jul 4;64(34):e202504378. doi: 10.1002/anie.202504378 (PMC12363612; doi:10.1002/anie.202504378)
Supplement: Supplementary file 1 — Supporting Information [file ANIE-64-e202504378-s001.pdf]

# Supplementary Information

## Regioselective C-H Functionalization by the Combination of Enzymatic and Chemo-Catalytic Reactions in Water

Ran Zhu,[a,b] Xuhua Mo,[a,c] Tanja Gulder,[d,e] and Tobias A. M. Gulder*[a,f]

[a] R. Zhu, X. Mo, Prof. T. A. M. Gulder
Chair of Technical Biochemistry, Department of Chemistry and Food Chemistry,
Technical University of Dresden
Bergstraße 66, 01069 Dresden (Germany)

[b] R. Zhu
Biosystems Chemistry, Faculty of Chemistry
Technical University of Munich
Lichtenbergstraße 4, 85748 Garching (Germany)

[c] X. Mo
Shandong Key Laboratory of Applied Mycology, School of Life Science
Qingdao Agricultural University
Qingdao, 266109 China

[d] Prof. T. Gulder
Organic Chemistry – Biomimetic Catalysis, Saarland University, 66123 Saarbruecken, Germany

[e] Prof. T. Gulder
Synthesis of Natural-Product Derived Drugs, Helmholtz Institute for Pharmaceutical Research Saarland (HIPS) Helmholtz Centre for Infection Research (HZI), 66123 Saarbruecken, Germany

[f] Prof. T. A. M. Gulder
Department of Natural Product Biotechnology
Helmholtz Institute for Pharmaceutical Research Saarland (HIPS), Helmholtz Centre for Infection Research (HZI) and Department of Pharmacy at Saarland University, PharmaScienceHub (PSH)
Campus E8.1, 66123 Saarbrücken, Germany. E-mail: Tobias.gulder@helmholtz-hips.de

# Content


1. Materials and Equipment .......... 3
   1.1 Materials .......... 3
   1.2 Chromatography (HPLC, LC-MS) .......... 3
   1.3 NMR .......... 3
2. Biological Methods .......... 4
   2.1 Plasmid construction and transformation .......... 4
   2.2 Protein expression .......... 4
   2.3 Protein purification .......... 5
   2.4 Supplementary information tables .......... 6
   2.5 SDS-PAGE .......... 8
   2.6 Preparation of cross-linked enzyme aggregates (CLEA)-FDH .......... 9
   2.7 Preparation of *Am*VHPO encapsulated in molecular weight cutoff (MWCO) tubings .......... 9
   2.8 Activity recovery and efficiency .......... 9
3. Chemical Methods .......... 10
   3.1 General halogenation methods .......... 10
   3.2 Heck-coupling condition screening method .......... 11
   3.3 General methods for preparation of PCRMs: .......... 11
   3.4 General methods combining enzymatic halogenation and Heck coupling .......... 13
   3.5 General hydrolysis method .......... 14
   3.6 General combined halogenation-Heck-coupling-hydrolysis catalytic platform ($H^3CP$) .......... 14
   3.7 General HPLC/LCMS methods .......... 16
   3.8 Determination of optimum substrate/enzyme pairs for enzymatic bromination .......... 18
   3.9 LCMS Monitoring of formation of 4c and side-product 4cc .......... 19
4. Compounds .......... 20
   4.1 Substances obtained by enzymatic halogenation .......... 20
   4.2 Compounds obtained by combination of bromination and Heck coupling reaction .......... 28
   4.3 Compounds made using $H^3CP$ .......... 35
5. NMR spectra .......... 43
6. References .......... 107

# 1. Materials and Equipment

## 1.1 Materials

Unless otherwise noted, all reagents were obtained from commercial suppliers and used without further purification. Commercial materials and solvents were purchased from commercial providers including Acros Organics, Alfa Aesar, Carbolution, Carl Roth, Merck, Sigma Aldrich, VWR, TCI Chemicals, BLD Pharma, and Thermo Fisher Scientific, ensuring the highest commercially available quality. Deuterated solvents were obtained from Sigma. Silica gel F254 (Merck) was used for analytical thin-layer chromatography (TLC), and column chromatography was performed using SiliCycle SilicaFlash silica gel (230-400 mesh), both with UV detection at 254 and 365 nm. Column chromatography was performed on silica gel 60 Geduran® Si 60 (40-60 µm) (Merck). Air-sensitive reactions were performed under an argon atmosphere. Before application, the flasks were repeatedly evacuated (with external heating) and backfilled with argon. Esterase from porcine liver used for hydrolysis and alcohol dehydrogenase (ADH) from *Saccharomyces cerevisiae* were purchased from Sigma-Aldrich. HisPur Ni-NTA columns from Thermo Fisher Scientific were used for protein purification. PD-10 columns and Vivaspin 2 Hydrosart membrane columns (MWCO) were purchased from VWR. Protein production, enrichment, and purification were monitored by SDS-PAGE analysis (BioRad Mini Protean® Tetra System) using an Unstained Protein MW Marker (Thermo Scientific). Plasmid pHis8-TEV with kanamycin resistance was used as a vector to express proteins.

## 1.2 Chromatography (HPLC, LC-MS)

Solvents were purchased from Fisher Scientific and VWR with a purity of over 99% (HPLC-grade). Water was purified and deionized using a TKA GenPure water treatment system. High Performance Liquid Chromatography (HPLC) analysis was performed on an Azura HPLC device manufactured by Knauer, consisting of the following components: AS 6.1L sampler, P 6.1L pump, DAD 2.1L detector. LC-MS analyses were performed on a Bruker UHPLC, which consisted of an Elute autosampler and a HPG 1300 pump. This was coupled to an Impact II mass spectrometer equipped with an ESI source and Q-TOF mass analyzer manufactured by Bruker. Water (A) and acetonitrile (B), both supplemented with 0.05% formic acid, were used as eluents. For MS/MS, auto-MS/MS mode with 20–50 eV collision energy ($N_2$) was used. The system was controlled by Bruker Compass® HyStar software, while analysis was conducted using Bruker Compass® Data Analysis software.

## 1.3 NMR

The $^{1}H$- and $^{13}C$ NMR spectra were recorded on Bruker AV400 and Bruker AV600 instruments using $CDCl_3$, DMSO-$d_6$, or $CD_3OD$ as the solvent. The chemical shifts are given in δ-values (ppm) and are calibrated on the residual peak of the deuterated solvent

($CDCl_3$: $\delta_H$ = 7.26 ppm, $\delta_C$ = 77.16 ppm, DMSO-*d*$_6$: $\delta_H$ = 2.50 ppm, $\delta_C$ = 39.52 ppm; $CD_3OD$: $\delta_H$ = 3.31 ppm, $\delta_C$ = 49.00 ppm). The coupling constants *J* are given in Hertz [Hz], assuming first-order spin-spin coupling. The following abbreviations were used for the allocation of signal multiplicities: br – broad signal, s – singlet, d – doublet, dd – doublet of doublets, ddd – doublet of doublet of doublets, t – triplet, dt – doublet of triplets, ddt – doublet of doublet of triplets, q – quartet, pp = pseudopentet, sx – sextet, m – multiplet.

# 2. Biological Methods

All gene fragments were amplified by PCR using Q5 high-fidelity DNA polymerase (NEB). All DNA fragments were purified using PCR and DNA Cleanup Kit (NEB). Sequence- and Ligation-Independent Cloning (SLIC) was used for DNA assembly and transformation according to the protocol of the manufacturer. After transformation, cells were plated on LB agar plates using suitable antibiotics for selection of positive transformants at 37 °C. Positive clones were screened by colony PCR using Taq DNA polymerase (NEB).

## 2.1 Plasmid construction and transformation

For the flavin adenine dinucleotide (FAD)-dependent halogenase (FDH) genes, *pyrH*, *rebH*, and *prnA*, genes were amplified from the respective pET28-vectors available in our lab using the primers PyrH-expE/F, RebH-expE/F, and PrnA-expE/F, respectively. The linear pHis8-TEV vector was amplified from the pHis8-TEV vector with the primers pHis8-TEVE/F. Then, the FDH genes *pyrH*, *rebH*, and *prnA* were assembled into the pHis8-TEV vector by SLIC to generate the plasmids pHis8-TEV-*pyrH*, pHis8-TEV-*rebH*, and pHis8-TEV-*prnA*, which were transformed into *E.coli* BL21 (DE3) for protein overexpression. The *fre* gene was amplified from a pET28b-Fre lab stock with primers Fre-expE/F. Subsequently, *fre* was assembled with the linear pHis8-TEV vector using SLIC to generate pHis8-TEV-Fre, which was introduced to *E. coli* BL21(DE3) for protein expression. The plasmid pET28-*Am*VHPO containing *Am*VHPO was present as a lab stock and transformed into *E. coli* BL21 (DE3) for protein production.

## 2.2 Protein expression

**FDHs**: The recombinant *E. coli* strains BL21(DE3), harboring plasmids with the corresponding genes, were grown in Erlenmeyer flasks containing 250 mL of LB medium (6 flasks, 1.5 L of LB in total) supplemented with kanamycin at a final concentration of 50 µg/mL. The cultures were incubated at 37 °C with shaking at 180 rpm until reaching an $OD_{600}$ of 0.6. Subsequently, isopropyl-β-D-thiogalactopyranoside (IPTG) was added at a final concentration of 0.1 mM. The cultures were further incubated at either 24 °C (for strains carrying plasmids pHis8-TEV-*pyrH*, pHis8-TEV-*rebH*, and pHis8-TEV-*prnA*) for 12 hours or at 18 °C (for strains carrying plasmids pHis8-TEV-Fre, and pHis8-TEV-*prnA*) with shaking at 180 rpm. The cells were then harvested by centrifugation (10000 rpm, 20 min) and used for subsequent protein purification.

***Am*VHPO**: Single colonies of *Am*VHPO transformant were picked from freshly prepared LB plates. The recombinant *E. coli* strain BL21(DE3) containing the plasmid was grown in

Erlenmeyer flasks containing 250 mL of LB medium (12 flasks, 3 L of LB in total) supplemented with kanamycin solution (50 mg/mL). The cultures were incubated at 37 °C with shaking at 180 rpm to an $OD_{600}$ of 0.6. Isopropylthiogalactoside (IPTG) solution (1 mM) was added, and then incubated at 180 rpm, 18 °C overnight. Cell pellets were harvested by centrifugation (10000 rpm, 20 min) and washed with 50 mM tris(hydroxymethyl)aminomethane (Tris) buffer pH 7.0 and 300 mM NaCl, then directly used or stored at 4°C.

## 2.3 Protein purification

**FDHs:** Cell pellets were resuspended in 30 mL of equilibration buffer (50 mM $NaH_2PO_4$, 300 mM NaCl, 10 mM imidazole). After sonication (10 min each, pulse: 10 s on, 10 s off, amplitude 50%, 4 °C) was performed on ice, the cellular debris and insoluble proteins were removed by centrifugation (15000 rpm, 30 min, 4 ºC), and the resulting supernatant was purified using nickel-nitrilotriacetic acid (Ni-NTA) affinity chromatography according to the protocol of the manufacturer (Novagen, CA, USA). The targeted proteins were eluted from the column with 2.5 mL of elution buffer (50 mM $NaH_2PO_4$, 300 mM NaCl, 400 mM imidazole), and then desalted using PD-10 columns. Protein concentrations were determined using the Pierce BCA Protein Assay Kit (Thermo Scientific, USA), and the protein purity was assessed by SDS-PAGE. The purified proteins were finally stored in 100 mM sodium phosphate buffer (PB buffer) (pH 7.2) with 10% glycerol at -80ºC for further use.

***Am*VHPO**: Cell pellets were resuspended in washing buffer (50 mM Tris pH 7.0, 300 mM NaCl, and 30 mM imidazole). Sonication (10 min each, pulse: 10 s on, 10 s off, amplitude 50%, 4 °C) was performed on ice. The resulting mixture was centrifugated at 21000 rpm for 30 min to remove cellular debris and insoluble proteins. The supernatant was then heated to 65°C for 30 min, followed by another centrifugation at 21000 rpm for 30 min. Next, the supernatant was loaded onto a 5 mL Histrap FF column (GE Healthcare) that had been equilibrated with washing buffer. The column was washed with 6 column volumes of the same buffer before the target protein was eluted using a 10 × column volume gradient ranging from 30 to 500 mM imidazole. SDS-PAGE was employed to analyze the fractions and those containing the target proteins were collected and desalted using PD-10 columns. Protein concentrations were determined using the Pierce BCA Protein Assay Kit (Thermo Scientific, USA). The purified proteins were finally stored in 50mM Tris buffer (pH 7.0) with 10% glycerol at -80ºC for further use.

**Fre**: Cell pellets from above were resuspended in 20 mL equilibration buffer (50 mM $KH_2PO_4$. pH 7.4) and sonicated 2 x 5 mins (5 min each, pulse: 10 s on, 10 s off, amplitude 50%, 4 °C), before being centrifuged at 10000 rpm for 30 min. The Ni-NTA column was equilibrated with 10 mM imidazole buffer prior to loading of clarified lysate supernatant. The column was then washed with 6 column volumes of the same buffer before elution of the target protein with a 10 × column volume gradient from 20 to 200 mM imidazole elution buffer (50 mm $NaH_2PO_4$, 300 mm NaCl, imidazole). The fractions were analyzed by SDS-PAGE and collected. The buffer was exchanged to 100 mM PB buffer (pH 7.4) containing glycerol (10% v/v final) and samples stored at -20°C until further use. The activity units (μmol/min) of purified Fre were determined using the standard spectrophotometric $NAD^+$/NADH reduction/oxidation method.

# 2.4 Supplementary information tables

**Table S1. Strains and plasmids used in the study.**

| Strains/Plasmids | Relevant properties | Source |
|---|---|---|
| *E. coli* DH5α | Host for general plasmid construction | NEB |
| *E. coli* BL21 | Host for protein overexpression | Novagen |
| pHis8-TEV- vector | Vector for cloning the genes | Lab stock |

**Table S2. List of primers.**

| Primers | Sequences (5'-3') |
|---|---|
| PyrH-expF | gtgagaatctttattttcagggaatggaaaggcggaagcgtg |
| PyrH-expR | gacggagctcgaattcggatcctcattggatgctggcgaggt |
| pHis8-TEVR | tccctgaaaataaagattctcac |
| pHis8-TEVF | ggatccgaattcgagctcc |
| RebH-expF | agaatctttattttcagggaatgtccggcaagattgacaa |
| RebH-expR | gacggagctcgaattcggatcctcagcggccgtgctgttgc |
| Fre-expF | tggtgagaatctttattttcagggaatgacaaccttaagctgtaaag |
| Fre-expR | cgacggagctcgaattcggatcctcagataaatgcaaacgcatc |
| PrnA-expF | tggtgagaatctttattttcagggaatgaacaagccgatcaaga |
| PrnA-expR | cgacggagctcgaattcggatccctacaggctttcctgcgctg |

**Table S3. Protein sequence information.**

| Protein | Sequences |
|---|---|
| pHis8-TEV-*pyrH* | MKHHHHHHHHGGENLYFQGMERRKRERLGSLGRPTKKELRMIRSVVIVG<br>GGTAGWMTASYLKAAFDDRIDVTLVESGNVRRIGVGEATFSTVRHFFDYL<br>GLDEREWLPRCAGGYKLGIRFENWSEPGEYFYHPFERLRVVDGFNMAE<br>WWLAVGDRRTSFSEACYLTHRLCEAKRAPRMLDGSLFASQVDESLGRST<br>LAEQRAQFPYAYHFDADEVARYLSEYAIARGVRHVVDDVQHVGQDERGW<br>ISGVHTKQHGEISGDLFVDCTGFRGLLINQTLGGRFQSFSDVLPNNRAVAL<br>RVPRENDEDMRPYTTATAMSAGWMWTIPLFKRDGNGYVYSDEFISPEEA<br>ERELRSTVAPGRDDLEANHIQMRIGRNERTWINNCVAVGLSAAFVEPLES<br>TGIFFIQHAIEQLVKHFPGERWDPVLISAYNERMAHMVDGVKEFLVLHYKG<br>AQREDTPYWKAAKTRAMPDGLARKLELSASHLLDEQTIYPYYHGFETYS |

| | |
|---|---|
| | WITMNLGLGIVPERPRPALLHMDPAPALAEFERLRREGDELIAALPSCYEY<br>LASIQ |
| pHis8-TEV-*rebH* | MKHHHHHHHHGGENLYFQGMSGKIDKILIVGGGTAGWMAASYLGKALQ<br>GTADITLLQAPDIPTLGVGEATIPNLQTAFFDFLGIPEDEWMRECNASYKVA<br>IKFINWRTAGEGTSEARELDGGPDHFYHSFGLLKYHEQIPLSHYWFDRSY<br>RGKTVEPFDYACYKEPVILDANRSPRRLDGSKVTNYAWHFDAHLVADFLR<br>RFATEKLGVRHVEDRVEHVQRDANGNIESVRTATGRVFDADLFVDCSGF<br>RGLLINKAMEEPFLDMSDHLLNDSAVATQVPHDDDANGVEPFTSAIAMKS<br>GWTWKIPMLGRFGTGYVYSSRFATEDEAVREFCEMWHLDPETQPLNRIR<br>FRVGRNRRAWVGNCVSIGTSSCFVEPLESTGIYFVYAALYQLVKHFPDKS<br>LNPVLTARFNREIETMFDDTRDFIQAHFYFSPRTDTPFWRANKELRLADG<br>MQEKIDMYRAGMAINAPASDDAQLYYGNFEEEFRNFWNNSNYYCVLAGL<br>GLVPDAPSPRLAHMPQATESVDEVFGAVKDRQRNLLETLPSLHEFLRQQ<br>HGR* |
| pHis8-TEV-*prnA* | MKHHHHHHHHGGENLYFQGMNKPIKNIVIVGGGTAGWMAASYLVRALQQ<br>QANITLIESAAIPRIGVGEATIPSLQKVFFDFLGIPEREWMPQVNGAFKAAIK<br>FVNWRKSPDPSRDDHFYHLFGNVPNCDGVPLTHYWLRKREQGFQQPM<br>EYACYPQPGALDGKLAPCLSDGTRQMSHAWHFDAHLVADFLKRWAVER<br>GVNRVVDEVVDVRLNNRGYISNLLTKEGRTLEADLFIDCSGMRGLLINQAL<br>KEPFIDMSDYLLCDSAVASAVPNDDARDGVEPYTSSIAMNSGWTWKIPML<br>GRFGSGYVFSSHFTSRDQATADFLKLWGLSDNQPLNQIKFRVGRNKRAW<br>VNNCVSIGLSSCFLEPLESTGIYFIYAALYQLVKHFPDTSFDPRLSDAFNAEI<br>VHMFDDCRDFVQAHYFTTSRDDTPFWLANRHDLRLSDAIKEKVQRYKAG<br>LPLTTTSFDDSTYYETFDYEFKNFWLNGNYYCIFAGLGMLPDRSLPLLQH<br>RPESIEKAEAMFASIRREAERLRTSLPTNYDYLRSLRDGDAGLSRGQRGP<br>KLAAQESL |
| pHis8-TEV-*fre* | MKHHHHHHHHGGENLYFQGMTTLSCKVTSVEAITDTVYRVRIVPDAAFSF<br>RAGQYLMVVMDERDKRPFSMASTPDEKGFIELHIGASEINLYAKAVMDRIL<br>KDHQIVVDIPHGEAWLRDDEERPMILIAGGTGFSYARSILLTALARNPNRDI<br>TIYWGGREEQHLYDLCELEALSLKHPGLQVVPVVEQPEAGWRGRTGTVL<br>TAVLQDHGTLAEHDIYIAGRFEMAKIARDLFCSERNAREDRLFGDAFAFI* |
| pET28-*Am*VHPO | MNTRRQQAQNIRNNAAELAANRPHPQHINNKEEYEYRRPKKDGNEPSHI<br>ANFTKGLPHDEHTGLLLNSADYDQFVLGIQSGDTTDFARTPLGPAELPKV |

| | |
|---|---|
| | HGCLSKQKIDCDDDHRSGFWKSQIAQGAAGGDGAKLRAWESAGAGLVF DLEGPDAQAVTMPPAPRLESPELTSEIAEVYSQALLRDIHFSQLRDPGLGD QVNACDSCPTQLSIYEAIDILNTVQIEGQNWFSANCCDLTDDEQARQRPL VTRQNIFRGIAPGDDVGPYLSQFLLIGNNALGGGVFGQEAGHIGYGAIRID QRVRKATPCKDFMTNFETWLDVQNGADLRGLETYVDADPGKCREFPAY RVITTPRDLATYVHYDALYEAYLNACLILLGMGAPFDPGIPFQKPDVEDKQ QGFAHFGGPQILTLVCEAATRGLKAVRFQKFNVHRRLRPEALGGLVDRYK HGKGAGDELKPVAALVEALENVGLLSKVVAHNQLQNQNLDRSGDPSSAG DNYFLPMAFPEGSPMHPSYGAGHATVAGACVTMLKAFFDHGWQLNLGM ANGKYISYEPNQDGSSLQQVLLDCPLTVEGELNKIAANISIGRDWAGVHYF TDYIESLRLGEKIAIGILEEQKLTYGENFTMTVPLYDGGSIQI |

## 2.5 SDS-PAGE

The SDS-PAGE for determination of enzyme purity was performed on 40% acrylamide gels, using a Consort EV243 electrophoresis apparatus from Sigma-Aldrich. PAGE ruler unstained broad range ladder (120–5 kDa) from Thermo Fisher Scientific was used as a reference marker. Figure S1 shows a typical gel of purified proteins after staining and destaining.

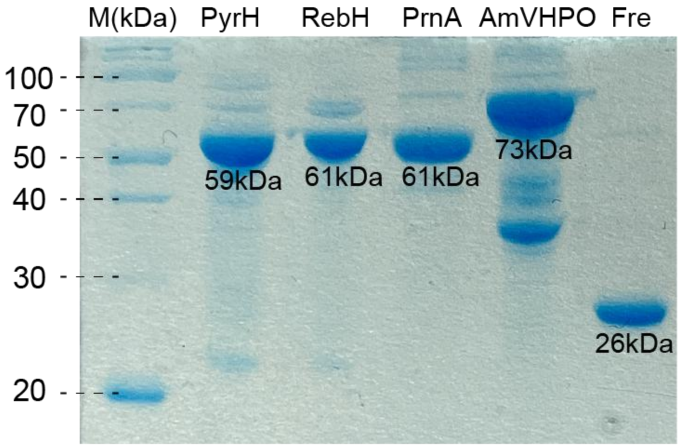


**Figure S1.** SDS-PAGE of purified FDHs: PyrH, RebH, PrnA; *Am*VHPO and reductase Fre. Molecular weight markers are shown on the left of the gel. Marker with given protein sizes in kDa.

## 2.6 Preparation of cross-linked enzyme aggregates (CLEA)-FDH

For FDHs, which were overexpressed in *E. coli* from a total of 1.5 L culture volume, pellets were resuspended in 40 mL lysis buffer (100 mM $Na_2HPO_4$ pH 7.4) and subjected to two rounds of sonication (10 min each, pulse: 10 s on, 10 s off, amplitude 50%, 4 °C). The samples were then centrifuged twice (12000 rpm) for 30 min at 4 °C. Subsequently, the purified Fre (2.5 U $ml^{-1}$) and ADH (1 U $ml^{-1}$) was added into 25 mL of the supernatant and thoroughly mixed. After the addition of 16.2 g ammonium sulfate at 4 °C over 2 h, precipitating protein aggregates were formed. Glutaraldehyde was then added, and the mixture was incubated for 2 h at 4 °C to achieve a final concentration of 0.5% w/v. The aggregates were collected by centrifugation (12000 rpm, 20 min, 4 °C) and washed three times with 30 mL of PB buffer (100 mM, pH 7.4). CLEA-FDHs were directly used or stored at 4 °C for future use.

## 2.7 Preparation of *Am*VHPO encapsulated in molecular weight cutoff (MWCO) tubings

For *Am*VHPO overexpressed in *E. coli* from a total of 3 L of culture volume, the cells were resuspended in 50 mL of buffer (50 mM Tris, pH 7.0, 300 mM NaCl) and lysed twice by sonication (10 min, pulse: 10 s on, 10 s off, amplitude 50%, at 4 °C). The resulting supernatant was then heated to 65°C for 30 min, followed by centrifugation at 21000 rpm for 30 min. Next, flash purification was performed using a Histrap FF column (GE Healthcare) that had been washed with the same buffer. Elution was carried out using a solution containing 50 mM Tris, pH 7.0, 300 mM NaCl, and 100 mM imidazole. The eluted protein was transferred into 3.5 mL of Tris buffer (pH 7.0, 100 mM) using a PD-10 column. For applying biocatalysis in reactions, every 0.5 mL of enzyme was encapsulated in tubing with a 10 kDa MWCO, resulting in a total of 7 portions. These portions could be directly added to reactions or stored at 4 °C prior to use.

## 2.8 Activity recovery and efficiency

CLEA-FDHs were tested over 5 recycling rounds. The substrates **5a** and **9a** were used to exemplarily test the enzymatic activity after each round. Enzyme resulting from a total of 1.5 L of *E. coli* culture was used in the recycling enzyme test reaction with 2 mM substrates. After the bromination step was completed, the CLEA-FDHs were removed by centrifugation at 10000 rpm for 20 min. The CLEA-FDHs were washed with PB buffer (pH 7.4, 10 mL) and THF (3 mL), followed by another washing step with PB buffer (pH 7.4, 5 mL). They were stored at 4 °C for subsequent cycles. The biocatalyst recycling was carried out for five cycles, and the yield in each cycle was determined by product isolation. When comparing the yields, the first three recycling cycles did not show a significant difference, with a slight decrease of efficacy afterwards. Similarly, *Am*VHPO encapsulated in MWCO tubings was also tested for 5 cycles. Substrate **17a** was used as the test substrate. Enzyme resulting from a total of 3 L of *E. coli* culture was used in the recycling enzyme test reaction with 3 mM substrate. After the

bromination step was completed, the encapsulated *Am*VHPO in tubing was collected and washed with Tris buffer (pH 7.0, 50 mM, 5 mL). It was then stored at 4 °C with subsequent use in the next cycle. The product yield of each cycle was determined by isolation.

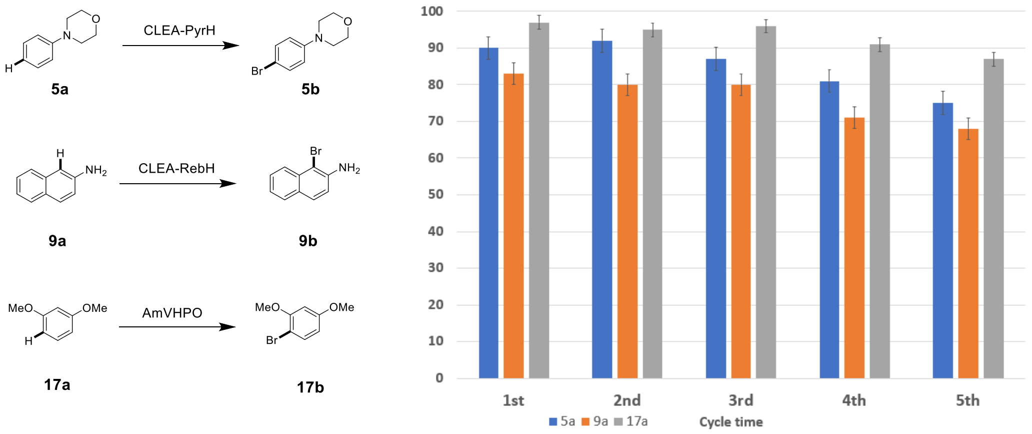


**Figure S2.** CLEA-FDHs and *Am*VHPO encapsulated in MWCO tubing were tested in 5 subsequent reactions upon recycling.

# 3. Chemical Methods

## 3.1 General halogenation methods

**Analytical scale enzymatic bromination Method 1 (for FDHs):** Substrate **a** (2 mM), NaBr (50 mM), FAD (10 μM), purified FDHs (25 μM), and Fre (1 μM) in 100 mM PB buffer (pH 7.2) were combined in a tube and NADH (20 mM) was added to a total volume of 200 μL. The mixture was incubated at room temperature (r.t.) 25 °C for 8 h with shaking at 800 rpm. The reaction was stopped by addition of methanol, further shaking for 10 min, and heating to 98 °C for 10 min. Subsequently, the mixture was centrifuged at 12600 rpm for 10 min. The resulting supernatant was analyzed using analytical HPLC, method 1.

**Scale-up enzymatic bromination Method 2 (for FDHs):** Substrate **a** (3.0 mM) in isopropanol (IPA) (5%), FAD (10 μM), NaBr (50 mM), and NADH (200 μM) were dissolved in a 100 mM PB buffer (pH 7.2). The alcohol dehydrogenase (1 μM), Fre (1.5 μM), and FDH (35 μM) were added to the solution, resulting in a total volume of 20 mL. The mixture was incubated at 25 °C overnight with shaking at 300 rpm. The reaction was quenched by heating to 98 °C for 20 min. Centrifugation, filtration, and extraction of the supernatant with ethyl acetate were performed. Organic phases were combined and solvents were removed under reduced pressure and the resulting product was purified by column chromatography.

**Analytical scale enzymatic bromination Method 3 (for *Am*VHPO):** Substrate **a** (2 mM), NaBr (4 mM), *Am*VHPO (3.7 µM, 15 µL, 4 mg/mL, preincubated with 15 µL of 20 mM $Na_3VO_4$), MES buffer (pH 6.0, 50 mM), and $H_2O_2$ (2 eq, 4 mM) were combined in a tube in acetonitrile (ACN)/$H_2O$ (1/1) to achieve a total volume of 200 µL. The mixture was then incubated at 30 °C for 8 hours with shaking at 600 rpm. The reaction was quenched by addition of 100 µL saturated NaCl and 200 µL saturated $(NH_4)_2SO_4$, followed by addition of ethyl acetate for extraction. The resulting organic solution was analyzed using analytical HPLC, method 2.

**Scale-up enzymatic bromination Method 4 (for *Am*VHPO):** Substrate **a** (3.0 mM), NaBr (4 mM), *Am*VHPO (3 µM, 600 µL, 4 mg/mL, preincubated with 400 µL of 60 mM $Na_3VO_4$), and MES buffer (pH 6.0, 50 mM) were combined in ACN/$H_2O$ (1/2) to a total volume of 20 mL, and $H_2O_2$ (1.1 eq, 3.3 mM) was added. The mixture was incubated at 30 °C overnight with shaking at 300 rpm. The reaction was stopped by addition of 10 mL saturated NaCl and 20 mL saturated $(NH_4)_2SO_4$, and ethyl acetate was added for extraction. The organic phases were combined, solvents were removed under reduced pressure, and products were purified by column chromatography.

## 3.2 Heck-coupling condition screening method

**Heck-coupling reaction condition screening:** Under an argon atmosphere, to a dried flask were added Pd($^tBu_3P)_2$ (2–5 mol-%), substrate 1-bromo-2,4-dimethoxybenzene **17b** (0.1 mmol), *tert*-butyl acrylate (1.5–2 eq.), and $Et_3N$ (0.3 mmol, 3 eq.). The flask was capped with a rubber septum, followed by addition of a solution of salts (NaCl, $Na_2CO_3$, etc.) in 5 wt % TPGS-750-M/$H_2O$ (1 mL). After stirring at 40°C under argon for 12-36 h, the pH value was adjusted to 7 with 1M HCl. After extraction with ethyl acetate (EA), the solvent was removed under reduced pressure, and product yields were determined after purification by column chromatography (pentane/ethyl acetate 80/1).

## 3.3 General methods for preparation of PCRMs:

After being incubated with a co-solvent, micelles expand the size of the reaction cavities in water, facilitating improved reactant access. The material obtained after this treatment is referred to as prefabricated catalyst-reactant mixture (PCRM), which can be directly used in the tested coupling reaction.

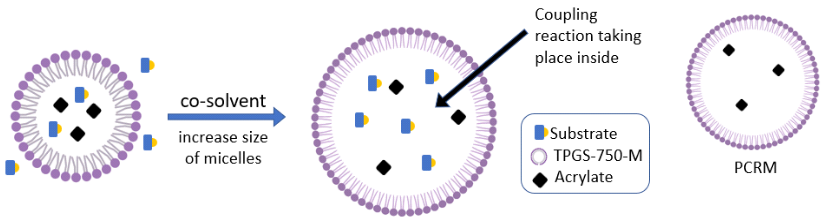


**Figure S3**. Schematic overview on prefabricated catalyst-reactant mixtures (PCRM).

Standard reactions were conducted at a scale of 0.1 mmol. Under an argon atmosphere, a dried Schlenk flask was charged with a solution of NaCl (3M) in 5 wt % TPGS-750-M/$H_2O$ (1 mL), 5%(v) THF, 15%(v) $Et_3N$ and *tert*-butyl acrylate (0.15 mmol, 1.5 eq.). The resulting mixture was stirred at 25 °C under argon until the mixture transformed from clear into milky white. Pd ($^tBu_3P)_2$ (2%) was added under inert atmosphere and stirring was continued for 10 min. A syringe was used for addition of the PCRM into the 0.1 mmol scale reactions.

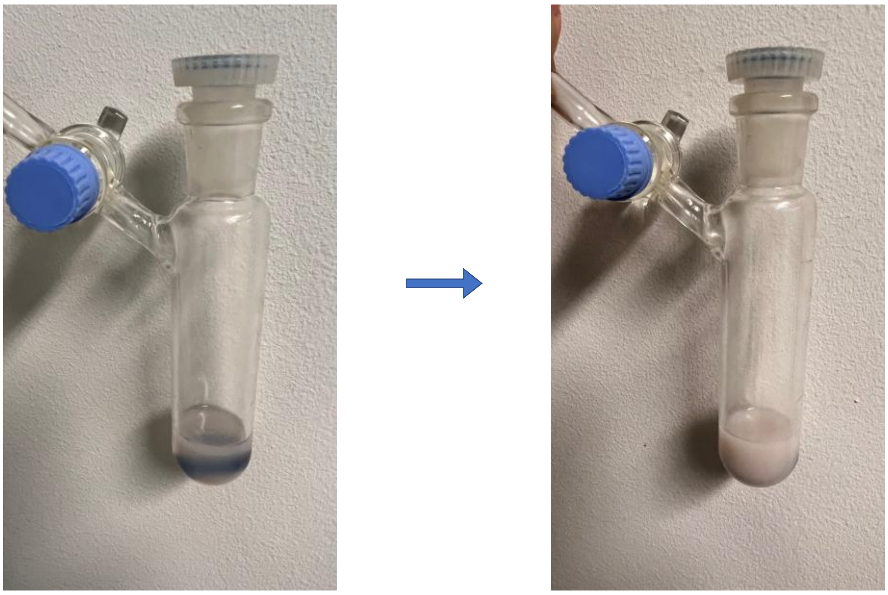

before after

**Figure S4.** Formation of PCRM. The left picture shows the solution when no micelle and cosolvent were added. The right picture shows the state of the solution after adding micelle and cosolvent to form the PCRM material.

**General methods to mimic combination reactions:**

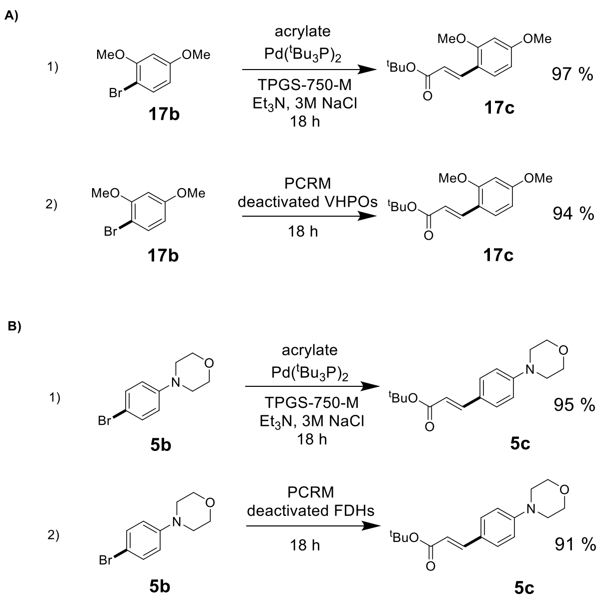

**A:** 1) Substrate **17b** (0.1 mmol) was reacted using optimized Heck-coupling reaction conditions. *tert*-butyl acrylate (1.5 equiv), $Et_3N$ (3 equiv), and NaCl (3M) were added to a dried flask with $Pd(^tBu_3P)_2$ (2%). The flask was capped with a rubber septum, followed by addition of a solution of 3M NaCl in 5 wt % TPGS-750-M/$H_2O$ (1 mL). After stirring at 40 °C under argon for 18 h, the pH value was adjusted to 7 with 1M HCl. The mixture was extracted with ethyl acetate, the solvent removed under reduced pressure, and the yield determined after purification of the desired product by column chromatography.

2) Halogenase *Am*VHPO (3 μM, 600 μL, 4 mg/mL, with 400 μL of 60 mM $Na_3VO_4$) in 20mL ACN/$H_2O$ (1/2) was heated to 98 °C for 20 min, and added to the reaction mixture. Substrate **17b** (0.1 mmol), PCRM (contains tert-butyl acrylate (0.15 mmol), triethylamine (0.3 mmol), in 1 mL 5 wt% TPGS-750-M, 2% Pd catalyst) was incubated with a mixture of heat-deactivated halogenase *Am*VHPO residual at 40 °C for 18 h under inert atmosphere. The pH was adjusted to 7 with 1M HCl. The solution was extracted with ethyl acetate, the solvent removed under reduced pressure, and yield determined by purification of the desired product by column chromatography.

**B)** 1) Substrate **5b** (0.1 mmol) was reacted under optimized Heck-coupling reaction conditions, *tert*-butyl acrylate (1.5 equiv), $Et_3N$ (3 equiv), and NaCl (3M) were added to a dried flask with $Pd(^tBu_3P)_2$ (2%). The flask was capped with a rubber septum, followed by addition of a solution of 3M NaCl in 5 wt % TPGS-750-M/$H_2O$ (1 mL). After stirring at 40 °C under argon for 18 h, the pH was adjusted to 7 with 1M HCl. The solution was extracted with ethyl acetate, the solvent removed under reduced pressure, and yield determined after purification of the desired product by column chromatography.

2) Halogenase PyrH (40 mM, pre incubated with 50 mM NaBr in 100 mM PB buffer (pH 7.2, 20 mL) and 1 μM heat-deactivated Fre, 10 μM FAD, 1 μM heat-deactivated ADH with 5% isopropanol were mixed, was heated at 98 °C for 20 min, and added for mimicking enzyme reaction conditions. Substrate **5b** (0.1 mmol), PCRM (contains tert-butyl acrylate (0.15 mmol), triethylamine (0.3 mmol), in 1 mL 5 wt% TPGS-750-M, 2% Pd catalyst), with heat-deactivated halogenase PyrH residuals at 40 °C for 18 h under inert atmosphere. The pH was adjusted to 7 with 1M HCl. The solution was extracted with ethyl acetate, the solvent removed under reduced pressure, and yield determined after purification of the desired product by column chromatography.

## 3.4 General methods combining enzymatic halogenation and Heck coupling

**General Method 5 using FDHs:** Substrate **a** (3.5 mM) in 5% IPA, FAD (10 μM), NaBr (50 mM), and NADH (200 μM) were dissolved in 100 mM PB buffer (pH 7.2). The alcohol dehydrogenase (1 μM), purified Fre (1.5 μM) and halogenase FDH (40 μM) were added to a total volume of 30 mL before being incubated at r.t. for 24h with shaking at 300 rpm. The

reaction was stopped by heating to 98 °C for 20 min. After cooling down to 25 °C, the reaction mixture was degassed by sonication, and backfilled with inert gas before being capped with a rubber septum. 1 mL PCRM (conducted at a scale of 0.1 mmol) was added into the supernatant using a syringe and the reaction mixture was stirred at 40 °C for 18 h. The pH value of the solution was adjusted to 7 with 1 M HCl (unless specified otherwise). Then, the solution was extracted with ethyl acetate. Organic phases were combined, concentrated in vacuo, analyzed by analytical HPLC method 3, and purified by column chromatography to afford corresponding product.

**General Method 6 using *Am*VHPO:** Substrate **a** (3.5 mM), NaBr (4 mM), *Am*VHPO (3.5 μM, preincubated with 400 μL of 60 mM $Na_3VO_4$), MES buffer (pH 6.0, 50 mM) in ACN/$H_2O$ (1/2) were combined in a total volume of 30 mL. $H_2O_2$ (1.1 eq., 3.3 mM) was added using a syringe pump. The solution was incubated at 30 °C for 24h with shaking at 300 rpm. Before heating at 98 °C for 20 min for enzyme deactivation, the pH of the reaction solution was neutralized using 1 M $NaHCO_3$. The reaction was cooled down to 25 °C, the solution degassed using ultrasound, inert gas atmosphere was established and the reaction tube closed with a rubber septum. 1 mL PCRM (conducted at a scale of 0.1 mmol) was added using a syringe, and then the reaction mixture was stirred at 40 °C for 18 h. After completion of the reaction, the pH value of the solution was adjusted to 7 with 1 M HCl (unless specified otherwise). Then, the solution was extracted with ethyl acetate, the organic phases were combined, and concentrated in vacuo. Reactions were analyzed by HPLC method 4, and purified by column chromatography to afford the corresponding product.

## 3.5 General hydrolysis method

**Integrated coupling-hydrolysis reaction condition screen:** The reaction was started by addition of **17b** (0.1 mmol) into PCRM (0.1 mmol scale showed above), then the reaction mixture was stirred at 40 °C for 18 h. 1 mL Medium (buffers, 100 mM) and additive $^t$BuOH (v/v) was added to the solution followed by pig liver esterase (PLE) (100 units, 6 mg). After stirring at room temperature for 25 °C 18h, the solution was acidified by addition of 1M HCl. Extraction with ethyl acetate, solvent removal under vacuum delivered the crude product, which was treated with ether and filtered to offer the product.

## 3.6 General combined halogenation-Heck-coupling-hydrolysis catalytic platform ($H^3CP$)

**General $H^3CP$ Method 7 using FDHs:** Halogenation and Heck coupling were performed as described for Method 5 above. The reaction solution was cooled down to 25 °C and neutralized by addition of PB buffer (pH 6.6), followed by addition of 5% $^t$BuOH (5%v) and PLE (100 units, 6 mg). After stirring at 25 °C for 18h, the solution was acidified by addition of 1M HCl and filtered through celite. The filtrate was extracted with ethyl acetate and the filter cake was washed with

ethyl acetate. Combined organic phases were concentrated under reduced pressure. The residual was treated with ether and filtered, analyzed by analytical HPLC method 5, and then purified by column chromatography to give the desired product.

**General H$^3$CP Method 8 using *Am*VHPO:** Halogenation and Heck coupling were performed as described for Method 6 above. The reaction solution was cooled down to 25 °C and neutralized by addition of PB buffer (pH 6.6), followed by addition of 5% $^t$BuOH (5%v) and PLE (100 units, 6 mg). After stirring at 25 °C for 18h, the solution was acidified by addition of 1M HCl and filtered through celite. The filtrate was extracted with ethyl acetate and the filter cake was washed with ethyl acetate. Combined organic phases were concentrated under reduced pressure. The residual was treated with ether and filtered, analyzed by analytical HPLC method 5, and then purified by column chromatography to give the desired product.

**Scaling of H$^3$CP Method 9 using FDH-CLEAs:** FDH-CLEAs prepared from protein derived of 1.5 L *E. coli.* cultures were suspended in flasks containing a solution of the substrate **a** (2 mM, 1 mmol), FAD (10 µM), NaBr (50 mM), and NADH (200 µM) dissolved in 500 mL PB buffer (20 mM, pH 7.2) with 5% IPA (v/v). The reaction was then shaken at 200 rpm at 25 °C. After 48 h, another equal portion of FDH-CLEA was added and the reaction kept stirring for 48h. The biocatalyst was removed by centrifugation at 10000 rpm for 20 min. The precipitated CLEAs were collected and washed with PB buffer (pH 7.4, 10 mL) and THF (3 mL), then washed with PB buffer (pH 7.4, 5 mL) and stored at 4 °C until next use. The combined supernatant was heated at 98 °C for 30 min to quench residual enzyme activity. After cooling down to 25 °C, the reaction mixture was degassed by sonication and filled with inert gas before being capped with a rubber septum. PCRM was added into the supernatant using a syringe, and then the reaction mixture was stirred at 40 °C for 24 h. (PCRM was prepared following the method outlined above with NaCl (3M) in 5 wt% TPGS-750-M/$H_2O$ (10 mL), 5%(v) THF, 15%(v) $Et_3N$, *tert*-butyl acrylate (1.5 eq.) and Pd ($^tBu_3P)_2$ (2%)). After cooling the reaction down to 25 °C and neutralization using PB buffer (pH 6.6), 5% $^t$BuOH(v/v) and PLE (1000 units, 55 mg) were added. The reaction was kept stirring at 25 °C for 48 h and was subsequently filtered using celite. The residual was acidified with 2 M HCl and extracted with ethyl acetate. Combined organic phases were evaporated under reduced pressure. The residual was washed with ether and filtered to give the desired acid product without further purification.

**Scaling of H$^3$CP Method 10 using encapsulated *Am*VHPO:** Encapsulated *Am*VHPO was used for halogenation (for its preparation, see above). Seven equal portions (30 mM, encapsulated in MWCO) of encapsulated *Am*VHPO were resulting from protein derived of 3 L *E. coli* cultures. One portion was added to a flask containing solution of substrate **a** (3 mM, 1 mmol), NaBr (3.3 mM), $Na_3VO_4$ (185 µM) dissolved in 330 mL MES buffer (pH 6.0, 50 mM) in ACN/$H_2O$ (1/2). The reaction was then shaken at 30 °C with 300 rpm for 48 h and $H_2O_2$ (1.1 eq, 3.3 mM) was added 5 times after 8 h each. The encapsulated *Am*VHPO in tubing was changed every 8 h. The replaced biocatalysts was washed with Tris buffer (pH 7.0, 50 mM, 5 mL) and stored at 4 °C for further use. Residual enzyme was deactivated by heating at 98 °C for 30 min. After cooling the solution down to 25 °C, the pH was neutralized with 2 M sodium hydrogen carbonate. Then, the reaction mixture was degassed by sonication, and backfilled with inert

gas before being capped with a rubber septum. PCRM was added into the supernatant using a syringe and then the reaction mixture was stirred at 40 °C for 24 h. (PCRM was prepared following the method above with NaCl (3M) in 5 wt % TPGS-750-M/$H_2O$ (10 mL), 5%(v) THF, 15%(v) $Et_3N$, *tert*-butyl acrylate (1.5 eq.) and Pd ($^tBu_3P)_2$ (2%)). The reaction was cooled down to 25 °C and neutralized using PB buffer (pH 6.6), followed by addition of 5% $^t$BuOH(v/v) and PLE (1000 units, 55 mg). The reaction was kept stirring at room temperature for 48 h and then filtered over celite. The solvent was removed under reduced pressure. The residual was acidified using 2 M HCl and extracted with ethyl acetate. Combined organic solutions were evaporated under reduced pressure. The residual was washed with ether and filtered to give the final product without further purification.

## 3.7 General HPLC/LCMS methods

**Analytical HPLC Method 1:** 10 µL of solution was injected onto a Phenomenex Luna 3µ C18(2) column (100 Å, 150 × 4,6 mm, 3µm) and separation performed using a gradient of 0–2 min 5% B, 2–26 min 5–95% B, 26–28 min 95% B, 28.2–30 min 95–5% B, 30–31 min 5% B. Mobile phase A: $H_2O$ + 0.05% trifluoroacetic acid (TFA); mobile phase B: acetonitrile (ACN) + 0.05% TFA. Flow rate was maintained at 1.0 mL $min^{-1}$ and UV absorbance detected at 220, 254, 280 and 319 nm throughout.

**Analytical HPLC Method 2:** 10 µL of solution was injected onto a 150 2 x 4 mm Eurospher II column (KNAUER®, 100 Å, 5 µm, C18, 25°C) and separation performed using of 0–2 min 10% B, 2–23 min 10–98% B, 23–25 min 98% B, 25.5–26 min 98–5% B, 26–28 min 5% B. Mobile phase A: $H_2O$ + 0.05% trifluoroacetic acid (TFA); mobile phase B: acetonitrile (ACN) + 0.05% TFA. Flow rate was maintained at 1.0 mL $min^{-1}$ and UV absorbance detected at 220, 254, 280 and 319 nm throughout.

**Analytical HPLC Method 3:** 10 µL of solution was injected onto a Phenomenex Luna 3µ C18(2) column (100 Å, 150 × 4,6 mm, 3µm) and separation performed using a gradient of 0–1 min 5% B, 1–28 min 5–95% B, 28–30 min 95% B, 30.1–31 min 95–5% B, 31 min 5% B. Mobile phase A: $H_2O$ + 0.05% trifluoroacetic acid (TFA); mobile phase B: acetonitrile (ACN) + 0.05% TFA. Flow rate was maintained at 1.0 mL $min^{-1}$ and UV absorbance detected at 220, 254, 280 and 319 nm throughout.

**Analytical HPLC Method 4:** 10 µL of solution was injected onto a 150 2 x 4 mm Eurospher II (KNAUER®, 100 Å, 5 µm, C18, 25°C) and separation performed using a gradient of 0–2 min 15% B, 2–25 min 20–98% B, 25–25.5 min 98% B, 25.5–26 min 98–5% B, 26–28 min 5% B. Mobile phase A: $H_2O$ + 0.05% trifluoroacetic acid (TFA); mobile phase B: acetonitrile (ACN) + 0.05% TFA. Flow rate was maintained at 1.0 mL $min^{-1}$ and UV absorbance detected at 220, 254, 280 and 319 nm throughout.

**Analytical HPLC Method 5 for H$^3$CP reaction:** 10 µL of solution was injected onto Phenomenex Luna 3µ C18(2) column (100 Å, 150 × 4,6 mm, 3µm), with a gradient of 0–2 min

5% B, 2–25 min 5–95% B, 28.0–28.1 min 95%–5% B, 28.1–30 min 5% B. Mobile phase A: $H_2O$ + 0.1% trifluoroacetic acid (TFA); mobile phase B: acetonitrile (ACN) + 0.1% TFA. Flow rate was maintained at 0.5 mL $min^{-1}$ and UV absorbance detected at 220, 254, 280 and 319 nm throughout.

**LCMS Method:** 2 μL of solution was injected onto Bruker Intensity Solo 2 C18, particle size 1.8 μm, 100 mm × 2.1 mm (in column oven: 40 °C), with a gradient of 0–2 min 95% A, 2–25 min 95%A–5%A, 25–28 min 5% A, 28–30 min 95% A; A: $H_2O$ + 0.1% trifluoroacetic acid (TFA); mobile phase B: acetonitrile (ACN) + 0.1% TFA. Flow rate was maintained at 0.3 mL $min^{-1}$ and UV absorbance detected at 220, 254, 280 and 319 nm throughout.

# 3.8 Determination of optimum substrate/enzyme pairs for enzymatic bromination

**Table S4.** Conversion of substrate **a** catalyzed by FDHs and VHPOs. The table depicts consumption of substrate as determined by HPLC analyses using HPLC methods 1 to 4.

Analytical scale:

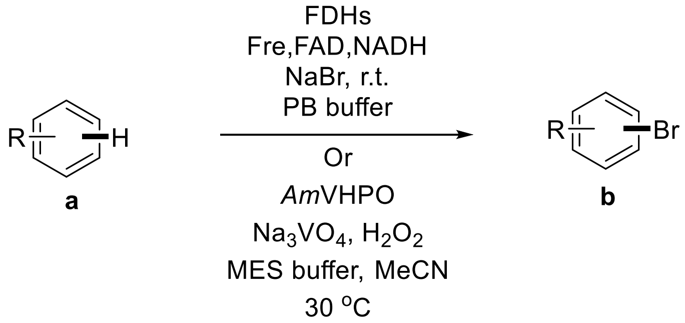


| | PyrH | RebH | PrnA | *Am*VHPO |
|---|---|---|---|---|
| **1b** | 77 | 23 | 0 | 0 |
| **2b** | 7 | 0 | 0 | 0 |
| **3b** | 0 | 0 | 0 | 0 |
| **4b** | 85 | 61 | 77 | 0 |
| **5b** | 97 | 81 | 65 | 0 |
| **6b** | 75 | 39 | 41 | 0 |
| **7b** | 5 | 17 | 49 | 0 |
| **8b** | 29 | 64 | 33 | 0 |
| **9b** | 17 | 92 | 0 | 0 |
| **10b** | 31 | 35 | 0 | 89 |
| **11b** | 0 | 82 | 23 | 0 |
| **12b** | 0 | 61 | 89 | 0 |
| **13b** | 11 | 39 | 0 | 0 |
| **14b** | 23 | 9 | 0 | 0 |
| **15b** | 0 | 0 | 0 | 77 |
| **16b** | 0 | 0 | 0 | 67 |
| **17b** | 0 | 0 | 0 | 98 |
| **18b** | 0 | 0 | 0 | 99 |
| **19b** | 0 | 0 | 0 | 92 |
| **20b** | 0 | 0 | 0 | 99 |
| **21b** | 9 | 0 | 0 | 84 |
| **22b** | 0 | 0 | 0 | 74 |
| **23b** | 0 | 0 | 0 | 66 |

The analytical reactions were with 2 mM substrate reacted for 8 h by using the general method 1&3 shown in Chapter 3.1. Conversion was measured based on consumption of substrates **a** as determined by HPLC method 1 to 4.

# 3.9 LCMS Monitoring of formation of 4c and side-product 4cc

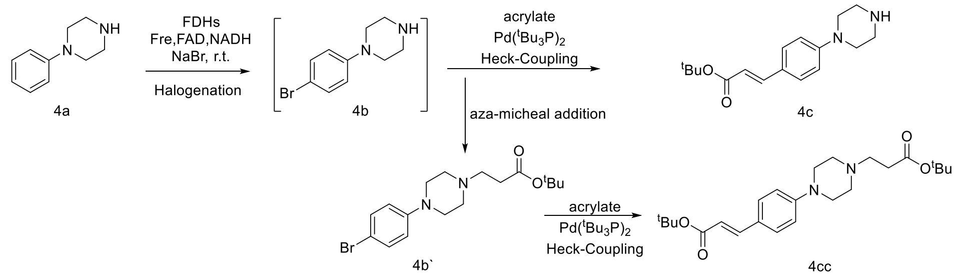


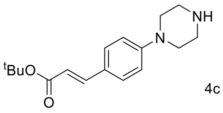


Chemical Formula : $C_{17}H_{25}N_2O_2$
Exact Mass $[M+H]^+$: 289.1911

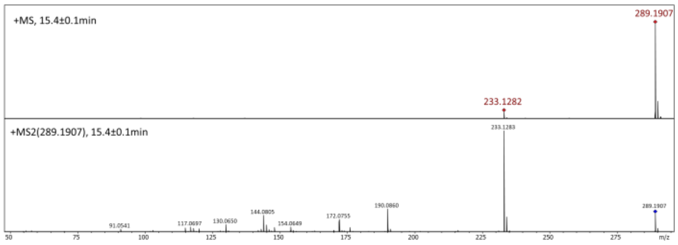


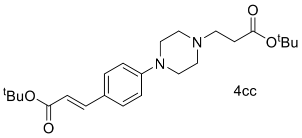


Chemical Formula: $C_{24}H_{37}N_2O_4$
Exact Mass $[M+H]^+$: 417.2748

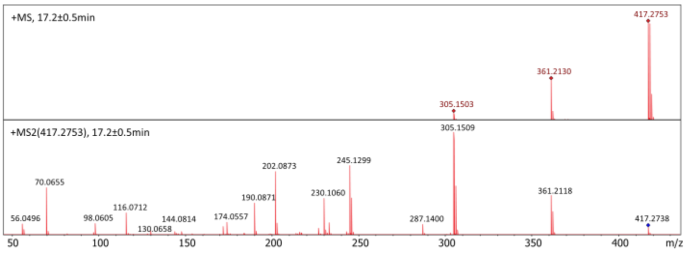


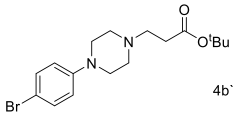


Chemical Formula: $C_{17}H_{26}BrN_2O_2$
Exact Mass $[M+H]^+$ : 369.1172

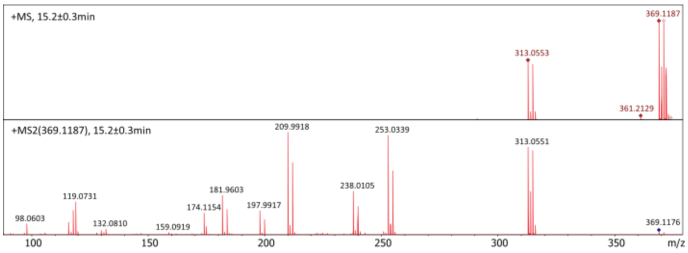


**Figure S5.** Side-product formation was observed during coupling reaction of **4b**. Determined structures of products after purification of scale-up bromination reactions using method 5. Substrate **a** (3 mM) in IPA, FAD (10 μM), NaBr (50 mM), NADH (200 μM), ADH (1 μM), Fre (1.5 μM), and halogenase PyrH (35 μM) in PB buffer (pH 7.2).

# 4. Compounds

## 4.1 Substances obtained by enzymatic halogenation

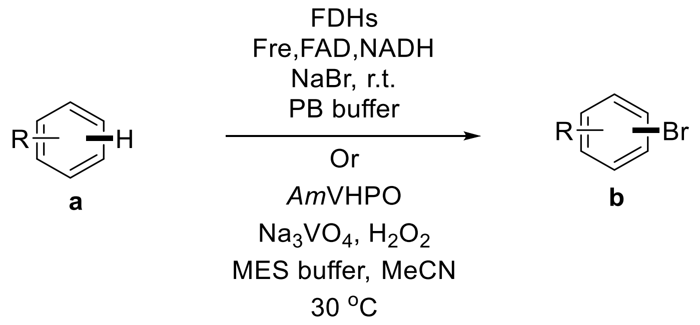


**2-amino-5-bromobenzamide (1b)**

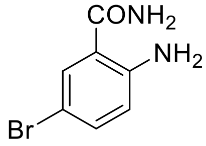


Compound **1b** was prepared from 8.2 mg (60 µmol) 2-amino-benzamide (**1a**) using the general method 2 detailed above in Chapter 3.1 with halogenase PyrH and purified on silica gel with pentane (PE)/ethyl acetate (EA)/TEA (1/2/1%) to afford 6.3 mg of a yellow solid (29 µmol, 49 %). $^{1}$H NMR (300 MHz, $CD_3OD$) δ [ppm] = 7.66 (d, *J* = 2.3 Hz, 1H), 7.26 (dd, *J* = 8.8, 2.3 Hz, 1H), 6.67 (d, *J* = 8.8 Hz, 1H). $^{13}$C NMR (75 MHz, $CD_3OD$) δ [ppm] = 173.3, 150.4, 136.2, 132.1, 120.0, 117.1, 107.7. HRMS *m/z* calculated for $C_7H_7{}^{79}BrN_2O$ [M+H$^+$] 214.9815, found: 214.9819.

**5-amino-2-bromophenol (2b)**

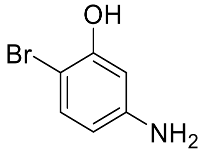


Compound **2b** was prepared from 6.5 mg (60 µmol) 3-aminophenol (**2a**) using the general method 2 detailed above in Chapter 3.1 with halogenase PyrH and purified on silica gel with dichloromethane (DCM)/methanol/TEA (25/1/1%) to afford 2.2 mg of a brown solid (12 µmol, 7 %). $^{1}$H NMR (600 MHz, DMSO-*d*$_6$) δ 9.61 (s, 1H), 7.00 (d, *J* = 8.5 Hz, 1H), 6.19 (d, *J* = 2.5 Hz, 1H), 5.97 (dd, *J* = 8.5, 2.5 Hz, 1H), 5.14 (s, 2H). $^{13}$C NMR (151 MHz, DMSO-*d*$_6$) δ 154.2, 149.3, 132.4, 107.2, 101.8, 94.5. HRMS *m/z* calculated for $C_6H_7{}^{79}BrNO$ [M+H$^+$] 187.9706 found: 187.9693.

**1-(4-bromophenyl)piperazine (4b)**

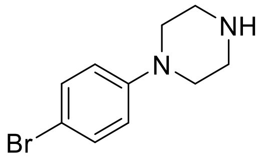

Compound **4b** was prepared from 9.7 mg (60 µmol) 1-phenylpiperazine (**4a**) using the general method 2 detailed above in Chapter 3.1 with halogenase PyrH and purified on silica gel with DCM/Methanol (30/1) to afford 10.5 mg of a light-yellow solid (44 µmol, 73 %). $^{1}$H NMR (300 MHz, $CDCl_3$) δ 7.34 (d, *J* = 9.0 Hz, 2H), 6.79 (d, *J* = 9.0 Hz, 2H), 3.16 – 3.06 (m, 4H), 3.05 – 2.95 (m, 4H). $^{13}$C NMR (75 MHz, $CDCl_3$) δ 151.0, 132.0, 117.8, 111.9, 50.4, 46.2. HRMS *m/z* calculated for $C_{10}H_{14}{}^{79}BrN_2$ [M+H$^+$] 241.0335, found: 241.0321.

**4-(4-bromophenyl)morpholine (5b)**

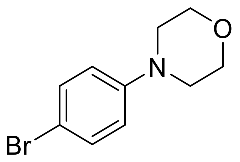


Compound **5b** was prepared from 9.7 mg (60 µmol) 1-phenylmorpholine (**5a**) using the general method 2 detailed above in Chapter 3.1 with halogenase PyrH, and purified on silica gel with PE/EA (25/1) to afford 13.3 mg of a white solid (55 µmol, 91 %). $^{1}$H NMR (300 MHz, $CDCl_3$) δ 7.36 (d, *J* = 9.0 Hz, 2H), 6.81 (d, *J* = 8.5 Hz, 2H), 3.93 – 3.77 (m, 4H), 3.18 – 3.06 (m, 4H). HRMS *m/z* calculated for $C_{10}H_{13}{}^{79}BrNO$ [M+H$^+$] 242.0175, found: 242.0177. The analytical data is in agreement with the literature.[1]

**4-(4-bromophenyl)thiomorpholine (6b)**

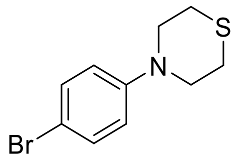


Compound **6b** was prepared from 10.7 mg (60 µmol) 4-phenylthiomorpholine (**6a**) using the general method 2 detailed above in Chapter 3.1 with halogenase PyrH, and purified on silica gel with PE/EA (20/1) to afford 8.8 mg of a yellow solid (35 µmol, 57 %). $^{1}$H NMR (300 MHz, $CDCl_3$) δ 7.61 – 7.42 (m, 2H), 7.38 – 7.28 (m, 2H), 3.72 – 3.47 (m, 4H), 3.25 – 2.88 (m, 4H). $^{13}$C NMR (151 MHz, DMSO-$d_6$) δ 160.7, 128.7, 117.7, 110.2, 51.9, 20.5. HRMS *m/z* calculated for $C_{10}H_{12}{}^{79}BrNS$ [M+H$^+$] 257.9947, found: 257.9938.

**(3R,5S)-1-(4-bromophenyl)-3,5-dimethylpiperazine (7b)**

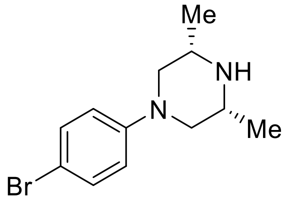


Compound **7b** was prepared from 11.4 mg (60 µmol) (3R,5S)-3,5-dimethyl-1-phenylpiperazine (**7a**) using the general method 2 detailed above in Chapter 3.1 with halogenase PrnA, and purified on silica gel with DCM/Methanol (10/1) to afford 8.1 mg of a dark-brown solid (30 µmol, 49 %). $^{1}$H NMR (300 MHz, $CD_3OD$) δ 7.39 (d, *J* = 9.1 Hz, 2H), 6.96 (d, *J* = 9.0 Hz, 2H), 3.86 – 3.70 (m, 2H), 3.46 – 3.34 (m, 2H), 2.71 – 2.53 (m, 2H), 1.35 (d, *J* = 6.5 Hz, 6H). $^{13}$C NMR (75 MHz, $CD_3OD$) δ 153.2, 135.8, 122.3, 116.6, 56.8, 55.4, 19.4. HRMS *m/z* calculated for $C_{12}H_{18}{}^{79}BrN_3$ [M+H$^+$] 269.0648, found: 269.0648.

**1-(4-bromophenyl)-4-methyl-1,4-diazepane (8b)**

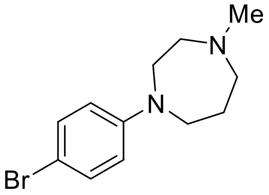


Compound **8b** was prepared from 11.4 mg (60 µmol) 1-methyl-4-phenyl-1,4-diazepane (**8a**) using the general method 2 detailed above in Chapter 3.1 with halogenase RebH, and purified on silica gel with DCM/Methanol (20/1) to afford 9.9 mg of a brown solid (37 µmol, 62 %). $^{1}$H NMR (600 MHz, $CD_3OD$) δ 7.27 (d, *J* = 9.2 Hz, 2H), 6.67 (d, *J* = 9.1 Hz, 2H), 3.61 – 3.56 (m, 2H), 3.49 (t, *J* = 6.3 Hz, 2H), 2.82 – 2.77 (m, 2H), 2.68 – 2.63 (m, 2H), 2.43 (s, 3H), 2.08 – 2.02 (m, 2H). $^{13}$C NMR (75 MHz, $CDCl_3$) δ 152.2, 135.5, 117.2, 111.3, 61.4, 60.6, 51.4, 51.3, 49.1, 30.6. HRMS *m/z* calculated for $C_{12}H_{18}{}^{79}BrN_3$ [M+H$^+$] 269.0648, found: 269.0660.

**1-bromonaphthalen-2-amine (9b)**

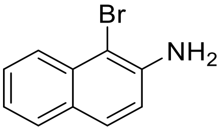


Compound **9b** was prepared from 8.6 mg (60 µmol) 2-aminenaphthalen (**9a**) using the general method 2 detailed above in Chapter 3.1 with halogenase RebH, and purified on silica gel with DCM/Methanol (25/1) to afford 11.0 mg of a dark brown solid (51 µmol, 85 %). $^{1}$H NMR (300 MHz, DMSO-*d*$_6$) δ 7.86 (d, J = 8.5 Hz, 1H), 7.72 (d, J = 8.1 Hz, 1H), 7.66 (d, J = 8.9 Hz, 1H), 7.48 (ddd, J = 8.4, 6.9, 1.4 Hz, 1H), 7.22 (ddd, J = 8.1, 6.9, 1.1 Hz, 1H), 7.13 (d, J = 8.8 Hz, 1H), 5.75 (s, 2H). $^{13}$C NMR (75 MHz, DMSO-*d*$_6$) δ 144.7, 133.2, 128.8, 128.6, 128.1, 127.7, 124.1, 122.3, 118.8, 101.0. HRMS *m/z* calculated for $C_{10}H_9{}^{79}BrN$ [M+H$^+$] 221.9913, found: 221.9913.

**2-bromo-1-naphthol (10b)**

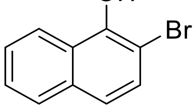


Compound **10b** was prepared from 8.6 mg (60 µmol) 1-naphthol (**10a**) using the general method 2 detailed above in Chapter 3.1 with halogenase RebH, and purified on silica gel with DCM/Methanol (25/1) to afford 4.6 mg of a pale-white solid (21 µmol, 35 %). $^{1}$H NMR (300 MHz, DMSO-*d*$_6$) δ 9.92 (s, 1H), 8.27 – 8.19 (m, 1H), 7.91 – 7.85 (m, 1H), 7.60 – 7.50 (m, 3H), 7.43 – 7.35 (m, 1H). $^{13}$C NMR (75 MHz, DMSO-*d*$_6$) δ 154.6, 138.6, 135.0, 133.0, 131.8, 131.2, 131.1, 127.3, 126.1, 110.0. HRMS *m/z* calculated for $C_{10}H_8{}^{79}BrO$ [M+H$^+$] 222.9759, found: 222.9739.

**7-bromo-tryptophol (11b)**

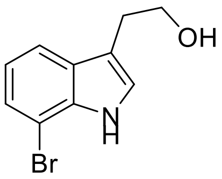


Compound **11b** was prepared from 9.7 mg (60 µmol) tryptophol (**11a**) using the general method 2 detailed above in Chapter 3.1 with halogenase RebH. And purified on silica gel with DCM/Methanol (25/1) to afford 10.2 mg of a pale-yellow solid (43 µmol, 71 %). $^{1}$H NMR (300 MHz, $CDCl_3$) δ 8.23 (s, 1H), 7.57 (d, *J* = 8.0 Hz, 1H), 7.36 (d, *J* = 7.7 Hz, 1H), 7.16 (s, 1H), 7.01 (t, *J* = 7.8 Hz, 1H), 3.91 (t, *J* = 6.4 Hz, 2H), 3.02 (t, *J* = 6.3 Hz, 2H). $^{13}$C NMR (75 MHz, $CDCl_3$) δ 135.2, 128.8, 124.7, 123.1, 120.8, 118.3, 113.9, 105.0, 62.8, 29.0. HRMS *m/z* calculated for $C_{10}H_{11}{}^{79}BrNO$ [M+H$^+$] 240.0019, found: 240.0011.

**7-bromotryptamine (12b)**

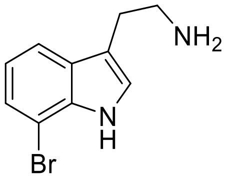


Compound **12b** was prepared from 9.7 mg (60 µmol) tryptophane (**12a**) using the general method 2 detailed above in Chapter 3.1 with halogenase PrnA. And purified on silica gel with DCM/Methanol (15/1) to afford 12.5 mg of a yellow solid (52 µmol, 87 %). $^{1}$H NMR (300 MHz, Methanol-*d*$_4$) δ 7.57 (d, *J* = 8.0 Hz, 1H), 7.30 (d, *J* = 7.6 Hz, 1H), 7.21 (s, 1H), 6.96 (t, *J* = 7.7 Hz, 1H), 3.09 – 2.91 (m, 5H). $^{13}$C NMR (75 MHz, Methanol-*d*$_4$) δ 136.7, 130.2, 125.0, 124.9, 121.0, 118.70 114.0, 105.6, 42.5, 28.2. HRMS *m/z* calculated for $C_{10}H_{12}{}^{79}BrN_2$ [M+H$^+$] 239.0184, found: 239.0182.

**6-bromo-1,2,3,4-tetrahydro-β-carboline (13b)**

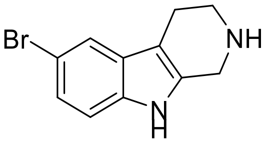


Compound **13b** was prepared from 10.3 mg (60 µmol) tetrahydrobetacarboline (**13a**) using the general method 2 detailed above in Chapter 3.1 with halogenase RebH. And purified on silica gel with DCM/Methanol (11/1) to afford 6.5 mg of a yellow oil (26 µmol, 43%). $^{1}$H NMR (300 MHz, DMSO-*d*$_6$) δ 11.04 (s, 1H, NH from indole), 7.56 (d, *J* = 1.9 Hz, 1H), 7.27 (d, *J* = 8.5 Hz, 1H), 7.13 (dd, *J* = 8.5, 2.0 Hz, 1H), 3.16 (s, 2H), 2.79 – 2.64 (m, 4H). $^{13}$C NMR (151 MHz, DMSO-*d*$_6$) δ 134.9, 133.1, 129.0, 123.6, 120.3, 113.5, 111.6, 106.7, 49.1, 46.0, 20.6. HRMS *m/z* calculated for $C_{11}H_{12}{}^{79}BrN_2$ [M+H$^+$] 251.0178, found: 251.0173.

### 3-(5-bromo-1H-indol-3-yl)propanoic acid (14b)

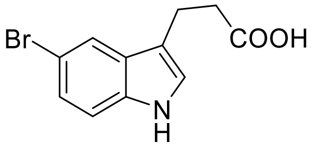


Compound **14b** was prepared from 16.0 mg (60 µmol) indole-3-acetic acid (**14a**) using the general method 2 detailed above in Chapter 3.1 with halogenase PyrH. And purified on silica gel with DCM/Methanol/TFA (5/1/5%) to afford 3.6 mg of a brown oil (13 µmol, 23%). And structure was determined by LCMS. HRMS *m/z* calculated for $C_{11}H_{10}{}^{79}BrNO_2$ [M+H$^+$] 267.9968, found: 267.9967. The analytical data is in agreement with the literature. [2]

### 4-bromo-1-naphthol (10b′)

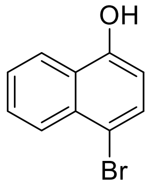


Compound **10b′** was prepared from 8.6 mg (60 µmol) 1-naphthol (**10a**) using the general method 4 detailed above in Chapter 3.1 with halogenase *Am*VHPO. And purified on silica gel with DCM/Methanol (25/1) to afford 11.7 mg of a white solid (53 µmol, 89%). $^1$H NMR (300 MHz, DMSO-*d*$_6$) δ 10.51 (br, 1H), 8.19 (d, *J* = 9.0 Hz, 1H), 8.01 (d, *J* = 8.3 Hz, 1H), 7.69 – 7.62 (m, 2H), 7.55 (ddd, *J* = 8.2, 6.8, 1.2 Hz, 1H), 6.82 (d, *J* = 8.2 Hz, 1H). HRMS *m/z* calculated for $C_{10}H_8{}^{79}BrO$ [M+H$^+$] 222.9753, found: 222.9743. The analytical data is in agreement with the literature.[3]

### 5-bromoisoquinolin-6-ol (15b)

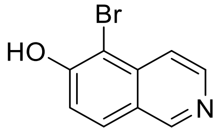


Compound **15b** was prepared from 8.6 mg (60 µmol) isoquinolin-6-ol (**15a**) using the general method 4 detailed above in Chapter 3.1 with halogenase *Am*VHPO. And purified on silica gel with DCM/Methanol (5/1) to afford 10.3 mg of a yellow solid (46 µmol, 77%). $^1$H NMR (300 MHz, DMSO-*d*$_6$) δ 8.64 (s, 1H), 8.11 (d, *J* = 6.0 Hz, 1H), 7.56 (d, *J* = 8.9 Hz, 1H), 7.38 (d, *J* = 6.0 Hz, 1H), 6.92 (d, *J* = 8.9 Hz, 1H). HRMS *m/z* calculated for $C_9H_7{}^{79}BrN$ [M+H$^+$] 223.9706, found: 223.9710. The analytical data is in agreement with the literature.[4]

### 8-bromoumbelliferone (16b)

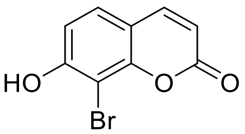


Compound **16b** was prepared from 9.7 mg (60 µmol) 7-methylcoumarin (**16a**) using the general method 4 detailed above in Chapter 3.1 with halogenase *Am*VHPO. And purified on silica gel

with DCM/Methanol (7/1) to afford 7.4 mg of a bright-yellow solid (31 µmol, 51%).$^1$H NMR (300 MHz, $CD_3OD$) δ 7.87 (d, *J* = 9.5 Hz, 1H), 7.47 (d, *J* = 8.6 Hz, 1H), 6.92 (d, *J* = 8.6 Hz, 1H), 6.25 (d, *J* = 9.5 Hz, 1H). HRMS *m/z* calculated for $C_9H_6{}^{79}BrO_3$ [M+H$^+$] 240.9495, found: 240.9482. The analytical data is in agreement with the literature. [4]

**3-bromo-7-hydroxycoumarin (16b′)**

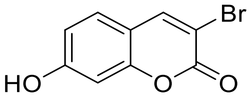


Compound **16b′** was prepared from 9.7 mg (60 µmol) 7-methylcoumarin (**16a**) using the general method 4 detailed above in Chapter 3.1 without halogenase *Am*VHPO and 5 eq. peroxide. And purified on silica gel with DCM/Methanol (7/1) to afford a light-yellow solid as side product. $^1$H NMR (300 MHz, DMSO-$d_6$) δ 10.73 (s, 1H), 8.50 (s, 1H), 7.53 (d, *J* = 8.6 Hz, 1H), 6.82 (dd, *J* = 8.5, 2.3 Hz, 1H), 6.75 (d, *J* = 2.3 Hz, 1H). HRMS *m/z* calculated for $C_9H_6{}^{79}BrO_3$ [M+H$^+$] 240.9495, found: 240.9491. The analytical data is in agreement with the literature.[5]

**3,8-dibromo-7-methylcoumarin (16bb)**

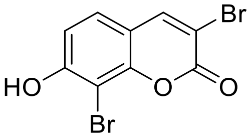


Compound **16bb** was prepared from 9.7 mg (60 µmol) 7-methylcoumarin (**16a**) using the general method 4 detailed above in Chapter 3.1 with halogenase *Am*VHPO and extra 3 eq. peroxide. And purified on silica gel with DCM/Methanol (10/1) to afford a bright-yellow solid as side product. $^1$H NMR (300 MHz, $CD_3OD$) δ 8.19 (s, 1H), 7.34 (d, *J* = 8.5 Hz, 1H), 6.82 (d, *J* = 8.6 Hz, 1H). HRMS *m/z* calculated for $C_9H_5{}^{79}Br_2O_3$ [M+H$^+$] 318.8600, found: 318.8597. The analytical data is in agreement with the literature.[6]

**1-bromo-2,4-dimethoxybenzene (17b)**

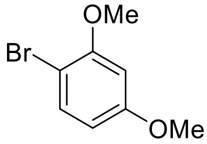


Compound **17b** was prepared from 8.3 mg (60 µmol) 1,3-dimethoxybenzene (**17a**) using the general method 4 detailed above in Chapter 3.1 with halogenase *Am*VHPO. And purified on silica gel with PE/EA (100/1) to afford 12.6 mg of a slightly yellow oil (58 µmol, 97%). $^1$H NMR (300 MHz, $CDCl_3$) δ 7.39 (d, *J* = 8.7 Hz, 1H), 6.48 (d, *J* = 2.7 Hz, 1H), 6.39 (dd, *J* = 8.7, 2.7 Hz, 1H), 3.86 (s, 3H), 3.79 (s, 3H). HRMS *m/z* calculated for $C_8H_{10}{}^{79}BrO_2$ [M+H$^+$] 216.9859, found: 216.9851. The analytical data is in agreement with the literature.[7]

**1-bromo-2,4,6-trimethoxybenzene (18b)**

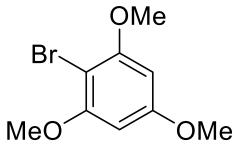

Compound of **18b** was prepared from 10.0 mg (60 µmol) 1,3,5-trimethoxybenzene (**18a**) using the general method 4 detailed above in Chapter 3.1 with halogenase *Am*VHPO. And purified on silica gel with PE/EA (120/1) to afford 12.8 mg of a white solid (52.2 µmol, 87%). $^1$H NMR (300 MHz, $CDCl_3$) δ 6.17 (s, 2H), 3.88 (s, 6H), 3.82 (s, 3H). HRMS *m/z* calculated for $C_8H_{10}{}^{79}BrO_2$ [M+H$^+$] 246.9964, found: 246.9952. The analytical data is in agreement with the literature.[7]

**4-bromothymol (19b)**

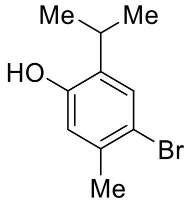

Compound **19b** was prepared from 9.0 mg (60 µmol) thymol (**19a**) using the general method 4 detailed above in Chapter 3.1 with halogenase *Am*VHPO. And purified on silica gel with PE/EA (30/1) to afford 12.2 mg of a light-yellow oil (53 µmol, 89%). $^1$H NMR (300 MHz, $CDCl_3$) δ 7.29 (s, 1H), 6.64 (s, 1H), 3.12 (p, *J* = 6.9 Hz, 1H), 2.30 (s, 3H), 1.23 (d, *J* = 6.9 Hz, 6H). HRMS *m/z* calculated for $C_{10}H_{14}{}^{79}BrO$ [M+H$^+$] 229.0223, found: 229.0222. The analytical data is in agreement with the literature.[7]

**4-Bromo-*N,N*-dimethylaniline (20b)**

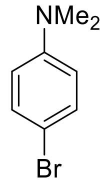

Compound **20b** was prepared from 7.3 mg (60 µmol) *N,N*-dimethylaniline (**20a**) using the general method 4 detailed above in Chapter 3.1 with halogenase *Am*VHPO. And purified on silica gel with PE/EA (80/1) to afford 11.0 mg of a grey solid (56 µmol, 93%). $^1$H NMR (300 MHz, DMSO-$d_6$) δ 7.29 (d, *J* = 9.2 Hz, 2H), 6.65 (d, *J* = 9.2 Hz, 2H), 2.87 (s, 6H). HRMS *m/z* calculated for $C_8H_{11}{}^{79}BrN$ [M+H$^+$] 200.0069, found: 200.0056. The analytical data is in agreement with the literature.[8]

**3-bromo-9H-carbazole (21b)**

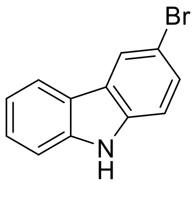

Compound **21b** was prepared from 10.0 mg (60 µmol) 9H-carbazole (**21a**) using the general method 4 detailed above in Chapter 3.1 with halogenase *Am*VHPO. And purified on silica gel with PE/EA (80/1) to afford 12.5 mg of a grey solid (51 µmol, 85%). $^1$H NMR (300 MHz, $CDCl_3$) δ 8.19 (d, *J* = 2.0 Hz, 1H), 8.03 (dd, *J* = 7.8, 1.3 Hz, 1H), 7.50 (dd, *J* = 8.6, 1.9 Hz, 1H), 7.46 – 7.42 (m, 2H), 7.42 – 7.35 (m, 1H), 7.32 (d, *J* = 8.6 Hz, 1H). HRMS *m/z* calculated for $C_7H_9{}^{79}BrNO_2$ [M+H$^+$] 245.9913, found: 245.9918. The analytical data is in agreement with the literature.[9]

**3-bromo-2,6-dimethoxypyridine (22b)**

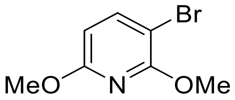


Compound **22b** was prepared from 8.3 mg (60 µmol) 2,6-dimethoxypyridine (**22a**) using the general method 4 detailed above in Chapter 3.1 with halogenase *Am*VHPO. And purified on silica gel with PE/EA (40/1) to afford the title compound as 4.5 mg of a colorless oil (21 µmol, 35%). $^1$H NMR (300 MHz, $CDCl_3$) δ 7.63 (d, *J* = 8.3, 1H), 6.23 (d, *J* = 8.3, 1H), 4.00 (s, 3H), 3.90 (s, 3H). $^{13}$C NMR (75 MHz, $CDCl_3$) δ 162.2, 158.5, 143.7, 102.8, 95.5, 54.3, 53.9. HRMS *m/z* calculated for $C_7H_9{}^{79}BrNO_2$ [M+H$^+$] 217.9811, found: 217.9802.

**2,4-dibromo-3-(4-morpholino)phenol (23bb)**

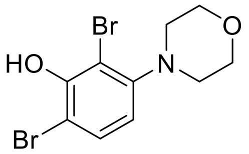


Compound of **23bb** was prepared from 15.4 mg (60 µmol) 3-(4-morpholino)phenol (**23a**) using the general method 4 detailed above in Chapter 3.1 with halogenase *Am*VHPO. And purified on silica gel with PE/EA (5/1) to afford 12.8 mg of a red solid (38 µmol, 64%). $^1$H NMR (300 MHz, $CD_3OD$) δ 7.44 (d, *J* = 8.6 Hz, 1H), 6.63 (d, *J* = 8.7 Hz, 1H), 3.90 – 3.78 (m, 4H), 3.05 – 2.95 (m, 4H). $^{13}$C NMR (75 MHz, $CD_3OD$) δ 151.7, 151.3, 131.4, 113.0, 108.9, 105.1, 66.8, 52.0. HRMS *m/z* calculated for $C_{10}H_{12}{}^{79}Br_2NO_2$ [M+H$^+$] 335.9229, found: 335.9213.

## 4.2 Compounds obtained by combination of bromination and Heck coupling reaction

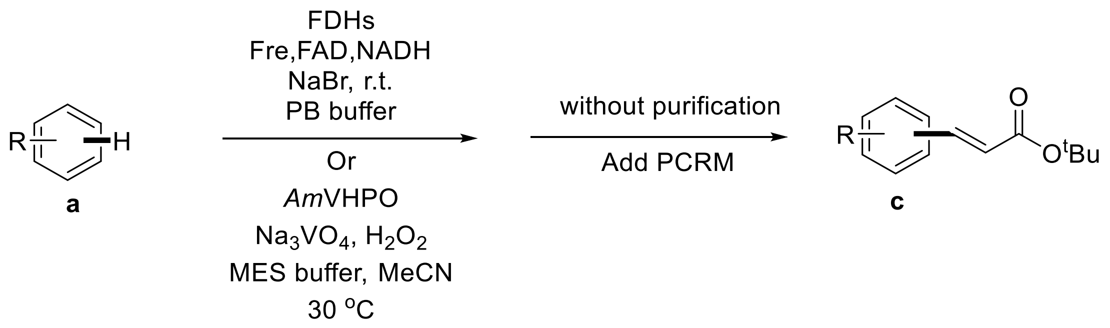


***tert*-butyl (*E*)-3-(4-amino-3-carbamoylphenyl)acrylate (1c)**

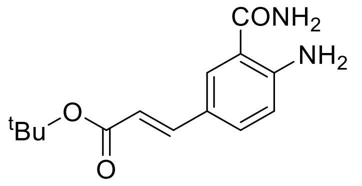


Compound **1c** was prepared from 14.3 mg (105 µmol) 2-amino-benzamide (**1a**) using the general method 5 detailed above in Chapter 3.4 with halogenase PyrH and purified on silica gel with PE/EA (20/1) to afford 11.3 mg of a pale-yellow oil (43 µmol, 41%). $^1$H NMR (600 MHz, $CD_3OD$) δ 7.79 (d, $J$ = 2.0 Hz, 1H), 7.53 – 7.45 (m, 2H), 6.76 (d, $J$ = 8.6 Hz, 1H), 6.24 (d, $J$ = 15.9 Hz, 1H), 1.53 (s, 9H). $^{13}$C NMR (101 MHz, $CD_3OD$) δ 172.5, 167.7, 152.1, 144.0, 131.3, 130.0, 121.8, 117.0, 114.3, 113.2, 79.9, 27.1. HRMS *m/z* calculated for $C_{14}H_{19}N_2O_3$ [M+H$^+$] 263.1390, found: 263.1401.

***tert*-butyl (*E*)-3-(4-(piperazin-1-yl)phenyl)acrylate (4c)**

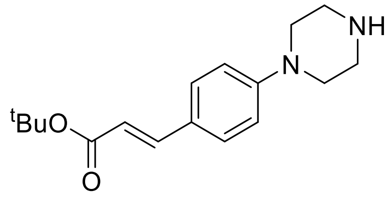


Compound **4c** was prepared from 17.0 mg (105 µmol) 1-phenylpiperazine (**4a**) using the general method 5 detailed above in Chapter 3.4 with halogenase PyrH and purified on silica gel with PE/EA (1/1) to afford 20.95 mg of a yellow oil (72 µmol, 69%). $^1$H NMR (400 MHz, $CD_3OD$) δ 7.55 – 7.46 (m, 3H), 7.01 (d, $J$ = 8.8 Hz, 2H), 6.27 (d, $J$ = 15.9 Hz, 1H), 3.70 – 3.66 (m, 2H), 3.62 – 3.59 (m, 2H), 3.35 – 3.32 (m, 2H), 3.31 – 3.26 (m, 2H), 1.54 (s, 9H). $^{13}$C NMR (101 MHz, $CD_3OD$) δ 167.4, 161.8, 152.4, 143.8, 129.2, 115.8, 115.6, 80.0, 48.9, 45.1, 27.1. HRMS *m/z* calculated for $C_{17}H_{25}N_2O_2$ [M+H$^+$] 289.1911, found: 289.1913.

**tert-butyl (*E*)-3-(4-(4-(3-(tert-butoxy)-3-oxopropyl)piperazin-1-yl)phenyl)acrylate (4cc)**

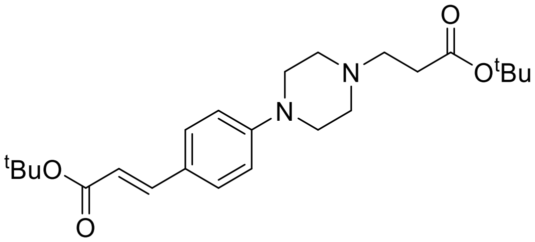


Compound **4cc** was prepared from 17.0 mg (105 µmol) 1-phenylpiperazine (**4a**) using the general method 5 detailed above in Chapter 3.4 with halogenase PyrH and purified on silica gel with PE/EA (1/2) to afford a yellow oil as side product. $^1$H NMR (600 MHz, $CDCl_3$) δ 7.51 (d, *J* = 15.9 Hz, 1H), 7.43 – 7.38 (m, 2H), 6.88 – 6.83 (m, 2H), 6.20 (d, *J* = 15.9 Hz, 1H), 3.31 – 3.25 (m, 4H), 2.77 – 2.71 (m, 2H), 2.64 (t, *J* = 5.1 Hz, 4H), 2.47 (t, *J* = 7.4 Hz, 2H), 1.52 (s, 9H), 1.45 (s, 9H). $^{13}$C NMR (75 MHz, $CDCl_3$) δ 171.8, 167.1, 152.3, 143.6, 129.5, 125.5, 116.5, 115.1, 80.6, 80.2, 53.8, 52.8, 48.1, 33.6, 28.4, 28.3. HRMS *m/z* calculated for $C_{24}H_{37}N_2O_4$ [M+H$^+$] 417.2748, found: 417.2749.

**tert-butyl (*E*)-3-(4-morpholinophenyl)acrylate (5c)**

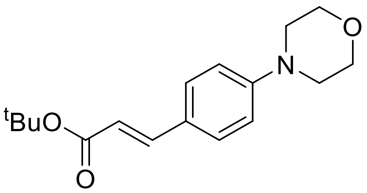


Compound **5c** was prepared from 17.0 mg (105 µmol) 4-phenylmorpholine (**5a**) using the general method 5 detailed above in Chapter 3.4 with halogenase PyrH and purified on silica gel with PE/EA (5/1) to afford 25.8 mg of a yellow solid (89 µmol, 85%). $^1$H NMR (300 MHz, $CDCl_3$) δ 7.62 – 7.47 (m, 5H), 6.34 (d, *J* = 16.0 Hz, 1H), 4.18 (t, *J* = 5.1 Hz, 4H), 3.42 (t, *J* = 4.9 Hz, 4H), 1.53 (s, 9H). HRMS *m/z* calculated for $C_{17}H_{24}NO_3$ [M+H$^+$] 290.1751, found:290.1758. The analytical data is in agreement with the literature.[10]

**tert-butyl (*E*)-3-(4-thiomorpholinophenyl)acrylate (6c)**

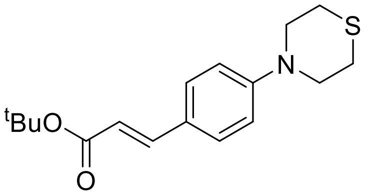


Compound **6c** was prepared from 18.8 mg (105 µmol) 4-phenylthiomorpholine (**6a**) using the general method 5 detailed above in Chapter 3.4 with halogenase PyrH and purified on silica gel with PE/EA (6/1) to afford 20.5 mg of a brown solid (67 µmol, 64%). $^1$H NMR (600 MHz, $CDCl_3$) δ 7.99 – 7.88 (m, 2H), 7.65 (dd, *J* = 18.2, 8.6 Hz, 2H), 7.55 (dd, *J* = 16.0, 6.0 Hz, 1H), 6.41 (dd, *J* = 16.0, 10.5 Hz, 1H), 4.50 – 4.25 (m, 2H), 4.26 – 4.09 (m, 2H), 3.60 – 3.54 (m, 2H), 3.16 – 3.12 (m, 2H), 1.54 (s, 9H). $^{13}$C NMR (75 MHz, $CDCl_3$) δ 167.5, 157.6, 144.4, 143.5, 129.7, 116.5, 115.7, 80.2, 51.1, 28.4, 26.3., HRMS *m/z* calculated for $C_{17}H_{24}NSO_2$ [M+H$^+$] 306.1522, found: 306.1531.

**tert-butyl (E)-3-(4-((3R,5S)-3,5-dimethylpiperazin-1-yl)phenyl)acrylate (7c)**

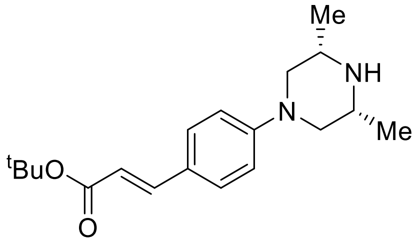


Compound **7c** was prepared from 19.9 mg (105 µmol) (3R,5S)-3,5-dimethyl-1-phenylpiperazine (**7a**) using the general method 5 detailed above in Chapter 3.4 with halogenase PrnA and purified on silica gel with PE/EA (1/3) to afford 17.8 mg of a brown solid (56 µmol, 53%). $^{1}H$ NMR (300 MHz, $CD_3OD$) δ 7.55 – 7.42 (m, 3H), 7.03 – 6.92 (m, 2H), 6.23 (d, $J$ = 15.9 Hz, 1H), 3.79 – 3.66 (m, 2H), 2.97 (dp, $J$ = 12.9, 6.4, 3.2 Hz, 2H), 2.35 (dd, $J$ = 12.2, 10.7 Hz, 2H), 1.54 (s, 9H), 1.19 (s, 3H), 1.17 (s, 3H). $^{13}C$ NMR (75 MHz, $CD_3OD$) δ 167.5, 152.5, 144.0, 129.2, 124.8, 115.1, 114.8, 79.9, 54.0, 50.3, 27.1, 17.7. HRMS *m/z* calculated for $C_{19}H_{29}N_2O_2$ [M+H$^+$] 317.2224, found:317.2221.

**tert-butyl (E)-3-(4-(4-methyl-1,4-diazepan-1-yl)phenyl)acrylate (8c)**

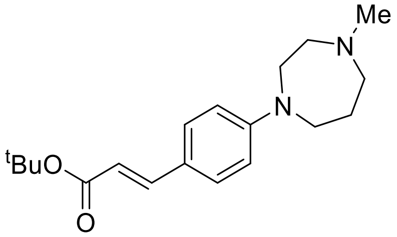


Compound **8c** was prepared from 19.9 mg (105 µmol) 1-methyl-4-phenyl-1,4-diazepane (**8a**) using the general method 5 detailed above in Chapter 3.4 with halogenase RebH and purified on silica gel with PE/EA (1/3) to afford 18.9 mg of a brown solid (60 µmol, 57%).$^{1}H$ NMR (300 MHz, $CD_3OD$) δ 7.54 – 7.42 (m, 3H), 6.86 – 6.77 (m, 2H), 6.19 (d, $J$ = 15.7 Hz, 1H), 3.83 – 3.73 (m, 2H), 3.60 (t, $J$ = 6.3 Hz, 2H), 3.25 – 3.17 (m, 2H), 3.14 – 3.05 (m, 2H), 2.73 (s, 3H), 2.27 – 2.15 (m, 2H), 1.53 (s, 9H). $^{13}C$ NMR (75 MHz, $CD_3OD$) δ 167.8, 150.4, 144.3, 129.6, 122.6, 113.7, 111.4, 79.8, 57.1, 56.3, 47.1, 46.1, 44.6, 27.1, 25.8. HRMS *m/z* calculated for $C_{19}H_{29}N_2O_2$ [M+H$^+$] 317.2224, found:317.2232.

**tert-butyl (E)-3-(2-aminonaphthalen-1-yl)acrylate (9c)**

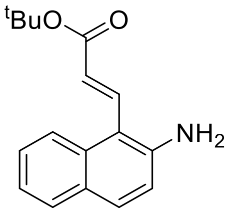


Compound **9c** was prepared from 15.0 mg (105 µmol) 2-Naphthylamine (**9a**) using the general method 5 detailed above in Chapter 3.4 with halogenase RebH and purified on silica gel with PE/EA (1/3) to afford 23.2 mg of a brown solid (86 µmol, 82%). $^{1}H$ NMR (300 MHz, $CDCl_3$) δ 8.11 (d, $J$ = 16.3 Hz, 1H), 7.90 (dq, $J$ = 8.5, 0.9 Hz, 1H), 7.74 – 7.59 (m, 2H), 7.45 (ddd, $J$ = 8.5,

6.8, 1.4 Hz, 1H), 7.31 – 7.23 (m, 1H), 6.94 (d, *J* = 8.8 Hz, 1H), 6.36 (d, *J* = 16.3 Hz, 1H), 4.24 (s, 2H), 1.58 (s, 9H). $^{13}$C NMR (75 MHz, $CDCl_3$) δ 166.6, 142.9, 139.5, 130.7, 128.6, 128.1, 128.1, 127.3, 125.0, 123.0, 122.8, 118.6, 111.9, 80.8, 28.4. HRMS *m/z* calculated for $C_{17}H_{20}NO_2$ [M+H$^+$] 270.1489, found:270.1491.

**_tert_-butyl (_E_)-3-(1-hydroxynaphthalen-2-yl)acrylate (10c)**

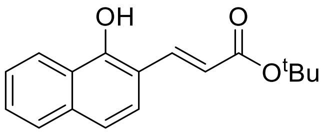


Compound **10c** was prepared from 15.0 mg (105 µmol) 1-naphthol (**10a**) using the general method 5 detailed above in Chapter 3.4 with halogenase RebH and purified on silica gel with PE/EA (10/1) to afford 4.8 mg of a light yellow solid (18 µmol, 17%). $^1$H NMR (300 MHz, $CD_3OD$) δ 8.31 (d, *J* = 16.0 Hz, 1H), 8.13 (dd, *J* = 8.8, 1.0 Hz, 1H), 7.85 – 7.73 (m, 2H), 7.52 (ddd, *J* = 8.5, 6.9, 1.4 Hz, 1H), 7.34 (ddd, *J* = 8.0, 6.8, 1.1 Hz, 1H), 7.16 (d, *J* = 8.9 Hz, 1H), 6.85 (d, *J* = 16.0 Hz, 1H), 1.59 (s, 9H). $^{13}$C NMR (75 MHz, $CD_3OD$) δ 168.3, 155.8, 136.7, 133.2, 131.2, 128.7, 128.4, 126.9, 123.1, 122.8, 121.8, 117.6, 112.8, 80.1, 27.1. HRMS *m/z* calculated for $C_{17}H_{19}O_3$ [M+H$^+$] 271.1329, found:271.1341.

**_tert_-butyl (_E_)-3-(4-hydroxynaphthalen-1-yl)acrylate (10c′)**

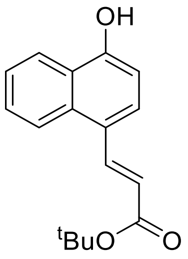


Compound **10c′** was prepared from 15.0 mg (105 µmol) 1-naphthol (**10a**) using the general method 6 detailed above in Chapter 3.4 with halogenase *Am*VHPO and purified on silica gel with PE/EA (9/1) to afford 19.3 mg of a brown solid (71 µmol, 68%). $^1$H NMR (300 MHz, $CDCl_3$) δ 8.38 (d, *J* = 15.7 Hz, 1H), 8.29 – 8.08 (m, 2H), 7.65 (d, *J* = 8.1 Hz, 1H), 7.61 – 7.46 (m, 2H), 6.84 (d, *J* = 8.0 Hz, 1H), 6.37 (d, *J* = 15.7 Hz, 1H), 1.57 (s, 9H). $^{13}$C NMR (75 MHz, $CDCl_3$) δ 167.0, 153.7, 140.5, 132.9, 127.5, 125.7, 125.6, 124.7, 124.5, 123.4, 122.5, 120.2, 108.7, 80.6, 28.4. HRMS *m/z* calculated for $C_{17}H_{19}O_3$ [M+H$^+$] 271.1329, found: 271.1329.

**_tert_-butyl (_E_)-3-(3-(2-hydroxyethyl)-1H-indol-7-yl)acrylate (11c)**

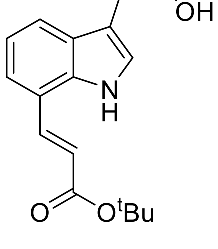

Compound **11c** was prepared from 16.9 mg (105 µmol) tryptophol (**11a**) using the general method 5 detailed above in Chapter 3.4 with halogenase RebH and purified on silica gel with PE/EA (1/1) to afford 20.2 mg of a light brown solid (70 µmol, 67%).$^{1}$H NMR (300 MHz, $CD_3OD$) δ 8.07 (d, *J* = 16.0 Hz, 1H), 7.70 (dd, *J* = 8.0, 1.0 Hz, 1H), 7.46 (d, *J* = 7.4 Hz, 1H), 7.23 (s, 1H), 7.13 (t, *J* = 7.7 Hz, 1H), 6.59 (d, *J* = 16.0 Hz, 1H), 3.88 (t, *J* = 7.2 Hz, 2H), 3.05 (td, *J* = 7.1, 0.8 Hz, 2H), 1.63 (s, 9H). $^{13}$C NMR (151 MHz, $CD_3OD$) δ 167.2, 140.5, 134.9, 128.9, 123.2, 121.1, 120.7, 118.6, 118.6, 118.2, 112.2, 80.2, 62.2, 28.2, 27.1. HRMS *m/z* calculated for $C_{17}H_{19}O_3$ [M+H$^{+}$] 288.1594, found: 288.1605.

***tert*-butyl (*E*)-3-(3-(2-aminoethyl)-1H-indol-7-yl)acrylate (12c)**

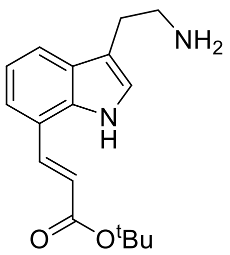


Compound **12c** was prepared from 16.9 mg (105 µmol) tryptophane (**12a**) using the general method 5 detailed above in Chapter 3.4 with halogenase PrnA and purified on silica gel with DCM/MeOH (20/1) to afford 18 mg of a yellow solid (63 µmol, 60%). $^{1}$H NMR (400 MHz, DMSO-*d*$_6$) δ 8.03 (d, *J* = 16.0 Hz, 1H), 7.60 (d, *J* = 7.8 Hz, 1H), 7.48 (d, *J* = 7.3 Hz, 1H), 7.20 (s, 1H), 7.03 (t, *J* = 7.6 Hz, 1H), 6.57 (d, *J* = 15.9 Hz, 1H), 3.65 (td, *J* = 7.3, 5.3 Hz, 2H), 2.89 – 2.78 (m, 2H), 1.51 (s, 9H). $^{13}$C NMR (151 MHz, DMSO-*d*$_6$) δ 166.0, 140.1, 134.5, 128.8, 123.8, 121.1, 120.8, 118.8, 118.7, 117.9, 112.5, 79.8, 61.6, 28.0, 25.8. HRMS *m/z* calculated for $C_{17}H_{23}N_2O_2$ [M+H$^{+}$] 287.1754, found: 287.1761.

***tert*-butyl (*E*)-3-(2,3,4,9-tetrahydro-1H-pyrido[3,4-b]indol-6-yl)acrylate (13c)**

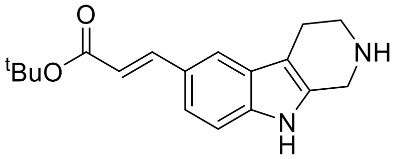


Compound **13c** was prepared from 18.6 mg (105 µmol) tetrahydrobetacarboline (**13a**) using the general method 5 detailed above in Chapter 3.4 with halogenase RebH and purified on silica gel with DCM/MeOH (5/1) to afford 22.6 mg of a brown solid (76 µmol, 72%). $^{1}$H NMR (300 MHz, $CD_3OD$) δ 7.71 (d, *J* = 15.9 Hz, 1H), 7.65 (d, *J* = 1.5 Hz, 1H), 7.41 – 7.30 (m, 2H), 6.35 (d, *J* = 15.8 Hz, 1H), 4.64 (s, 2H), 3.80 (t, *J* = 5.7 Hz, 2H), 2.81 (t, *J* = 5.9 Hz, 2H), 1.56 (s, 9H). $^{13}$C NMR (101 MHz, $CD_3OD$) δ 167.0, 144.4, 143.8, 129.3, 128.6, 127.7, 123.5, 119.4, 118.1, 118.0, 115.4, 80.2, 60.2, 27.3, 27.1, 27.0. HRMS *m/z* calculated for $C_{18}H_{23}N_2O_2$ [M+H$^{+}$] 299.1754, found: 299.1768.

**_tert_-butyl (_E_)-3-(7-hydroxy-2-oxo-2H-chromen-8-yl)acrylate (16c)**

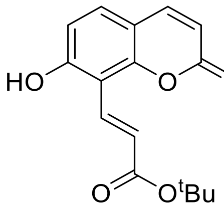


Compound **16c** was prepared from 17.1 mg (105 µmol) tetrahydrobetacarboline (**13a**) using the general method 6 detailed above in Chapter 3.4 with halogenase *Am*VHPO and purified on silica gel with DCM/MeOH (15/1) to afford 6.6 mg of a bright yellow solid (23 µmol, 22%). $^1$H NMR (300 MHz, $CD_3OD$) δ 8.09 (d, *J* = 16.3 Hz, 1H), 7.88 (d, *J* = 9.5 Hz, 1H), 7.49 (d, *J* = 8.6 Hz, 1H), 7.05 (d, *J* = 16.3 Hz, 1H), 6.90 (d, *J* = 8.7 Hz, 1H), 6.27 (d, *J* = 9.5 Hz, 1H), 1.57 (s, 9H). $^{13}$C NMR (151 MHz, $CD_3OD$) δ 167.7, 161.3, 154.2, 144.9, 132.2, 130.1, 123.8, 112.8, 111.7, 111.1, 111.0, 109.3, 80.2, 27.0. HRMS *m/z* calculated for $C_{16}H_{17}O_5$ [M+H$^+$] 289.1071, found: 289.1078.

**_tert_-butyl (_E_)-3-(2,4-dimethoxyphenyl)propenoate (17c)**

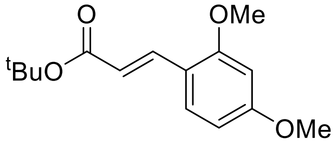


Compound **17c** was prepared from 14.5 mg (105 µmol) 1,3-bimethoxybenzene (**17a**) using the general method 6 detailed above in Chapter 3.4 with halogenase *Am*VHPO and purified on silica gel with PE/EA (90/1) to afford 25.6 mg of a colorless oil (97 µmol, 92%). $^1$H NMR (300 MHz, $CDCl_3$) δ 7.82 (d, *J* = 16.1 Hz, 1H), 7.42 (d, *J* = 8.5 Hz, 1H), 6.52 – 6.41 (m, 2H), 6.35 (d, *J* = 16.1 Hz, 1H), 3.85 (s, 3H), 3.83 (s, 3H), 1.52 (s, 9H). HRMS *m/z* calculated for $C_{15}H_{21}O_4$ [M+H$^+$] 265.1434, found: 265.1441. The analytical data is in agreement with the literature.[11]

**_tert_-butyl (_E_)-3-(2,4,6-dimethoxyphenyl)propenoate (18c)**

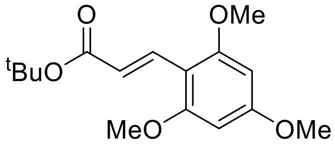


Compound **18c** was prepared from 17.6 mg (105 µmol) 1,3,5-trimethoxybenzene (**18a**) using the general method 6 detailed above in Chapter 3.4 with halogenase *Am*VHPO and purified on silica gel with PE/EA (85/1) to afford 25.7 mg of a grey solid (87 µmol, 83%). $^1$H NMR (300 MHz, $CDCl_3$) δ 8.01 (d, *J* = 16.2 Hz, 1H), 6.66 (d, *J* = 16.2 Hz, 1H), 6.10 (s, 2H), 3.85 (s, 6H), 3.83 (s, 3H), 1.51 (s, 9H). HRMS *m/z* calculated for $C_{16}H_{23}O_5$ [M+H$^+$] 295.1540, found: 295.1547. The analytical data is in agreement with the literature.[12]

### ***tert*-butyl (*E*)-3-(4-hydroxy-5-isopropyl-2-methylphenyl)acrylate (19c)**

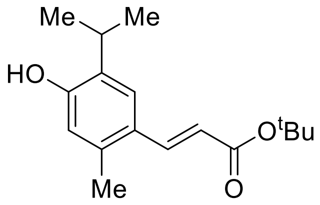

Compound **19c** was prepared from 15.7 mg (105 µmol) thymol (**19a**) using the general method 6 detailed above in Chapter 3.4 with halogenase *Am*VHPO and purified on silica gel with PE/EA (35/1) to afford 21.8 mg of a pale-yellow oil (78 µmol, 75%). $^1$H NMR (300 MHz, $CDCl_3$) 7.83 (d, *J* = 15.8 Hz, 1H), 7.42 (s, 1H), 6.57 (s, 1H), 6.21 (d, *J* = 15.8 Hz, 1H), 3.19 – 3.12 (m, 1H), 2.35 (s, 3H), 1.54 (s, 9H), 1.26 (s, 3H), 1.25 (s, 3H). $^{13}$C NMR (151 MHz, $CDCl_3$) δ 167.0, 154.3, 141.2, 141.1, 136.9, 132.5, 124.9, 118.0, 117.3, 80.2, 28.3, 26.8, 22.5, 19.2. HRMS *m/z* calculated for $C_{17}H_{25}O_3$ [M+H$^+$] 277.1798, found: 277.1806.

### ***tert*-butyl (*E*)-3-(4-(dimethylamino)phenyl)acrylate (20c)**

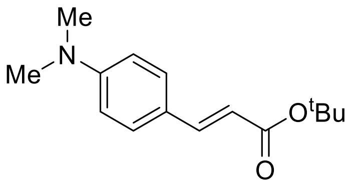

Compound **20c** was prepared from 12.7 mg (105 µmol) *N,N*-dimethylaniline (**20a**) using the general method 6 detailed above in Chapter 3.4 with halogenase *Am*VHPO and purified on silica gel with PE/EA (100/1) to afford 20.5 mg of a colorless oil (83 µmol, 79%). $^1$H NMR (300 MHz, $CDCl_3$) δ 7.53 (d, *J* = 15.9 Hz, 1H), δ 7.44 (d, *J* = 8.8 Hz, 2H), 6.88 (br, 2H), 6.20 (d, *J* = 16.0 Hz, 1H), 3.04 (s, 6H), 1.52 (s, 9H). HRMS *m/z* calculated for $C_{15}H_{22}NO_2$ [M+H$^+$] 248.1645, found: 248.1639. The analytical data is in agreement with the literature.[13]

### **(*E*)-*tert*-butyl 3-(9H-carbazol-6-yl)acrylate (21c)**

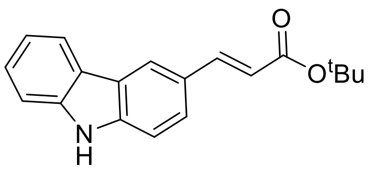

Compound **21c** was prepared from 17.5 mg (105 µmol) 9H-carbazole (**21a**) using the general method 6 detailed above in Chapter 3.4 with halogenase *Am*VHPO and purified on silica gel with PE/EA (50/1) to afford 18 mg of a white solid (72 µmol, 69%). $^1$H NMR (400 MHz, DMSO-*d*$_6$) δ 8.35 (s, 1H), 8.17 (d, *J* = 7.8, 1H), 7.84 (d, *J* = 15.9 Hz, 1H), 7.71 (dd, *J* = 8.5, 1.7 Hz, 1H), 7.52 (dd, *J* = 8.5, 2.3, 2H), 7.49 – 7.43 (m, 1H), 7.30 – 7.23 (m, 1H), 6.50 (d, *J* = 15.9 Hz, 1H), 1.63 (s, 9H). $^{13}$C NMR (151 MHz, DMSO-*d*$_6$) δ 169.0, 146.9, 142.9, 142.0, 127.2, 126.7, 126.5, 124.8, 124.2, 122.1, 121.2, 120.4, 116.9, 112.2, 112.0, 81.4, 28.5. HRMS *m/z* calculated for $C_{19}H_{20}NO_2$ [M+H$^+$] 294.1489, found: 294.1479.

**tert-butyl (E)-3-(2,6-dimethoxypyridin-3-yl)acrylate (22c)**

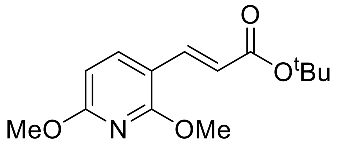


Compound **22c** was prepared from 14.6 mg (105 µmol) 2,6-dimethoxypyridine (**22a**) using the general method 6 detailed above in Chapter 3.4 with halogenase *Am*VHPO and purified on silica gel with PE/EA (70/1) to afford 9.2 mg of a colorless oil (35 µmol, 33%). $^1$H NMR (600 MHz, $CDCl_3$) δ 7.69 (d, *J* = 16.1 Hz, 1H), 7.65 (d, *J* = 8.2, 1H), 6.36 (d, *J* = 16.1 Hz, 1H), 6.32 (d, *J* = 8.2 Hz, 1H), 4.00 (s, 3H), 3.94 (s, 3H), 1.52 (s, 9H). $^{13}$C NMR (151 MHz, $CDCl_3$) δ 167.2, 163.9, 161.3, 140.6, 138.0, 118.7, 109.7, 102.1, 80.2, 53.9, 53.7, 28.4. HRMS *m/z* calculated for $C_{14}H_{20}NO_4$ [M+H$^+$] 266.1387, found: 266.1379.

# 4.3 Compounds made using H$^3$CP

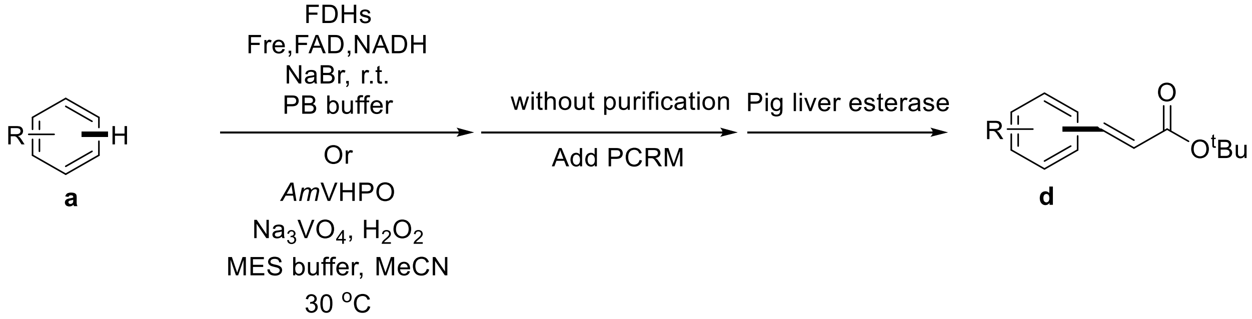


**(E)-3-(4-(piperazin-1-yl)phenyl)acrylic acid (1d)**

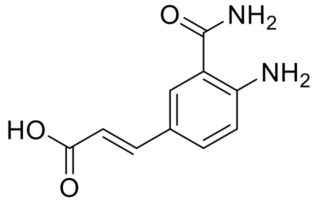


Compound **1d** was prepared from 14.2 mg (105 µmol) 2-amino-benzamide (**1a**) using the general method 7 detailed above in Chapter 3.6 with halogenase PyrH, and purified on silica gel with DCM/MeOH/TFA (20/1/5%) to afford 6.3 mg of a brown solid (30 µmol, 29%). $^1$H NMR (300 MHz, DMSO-$d_6$) δ 12.34 (br, 1H), 8.10 (s, 1H), 7.78 (d, *J* = 2.1 Hz, 1H), 7.60 (dd, *J* = 8.6, 2.2 Hz, 1H), 7.46 (d, *J* = 15.9 Hz, 1H), 7.27 (s, 1H), 6.66 (d, *J* = 8.5 Hz, 1H), 6.21 (d, *J* = 15.9 Hz, 1H). $^{13}$C NMR (151 MHz, DMSO-$d_6$) δ 168.0, 162.4, 148.6, 144.2, 132.8, 128.3, 122.5, 114.7, 114.2, 113.2. HRMS *m/z* calculated for $C_{10}H_{11}N_2O_3$ [M+H$^+$] 207.0764, found: 207.0771.

**(E)-3-(4-(piperazin-1-yl)phenyl)acrylic acid (4d)**

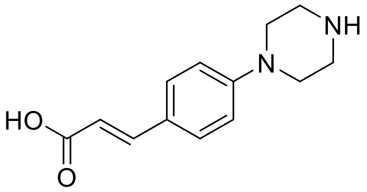

Compound **4d** was prepared from 17.0 mg (105 µmol) 1-phenylpiperazine (**4a**) using the general method 7 detailed above in Chapter 3.6 with halogenase PyrH, and purified on silica gel with DCM/MeOH/TFA (20/1/3%) to afford 12.9 mg of a dark-brown solid (55 µmol, 53%). The larger-scale reaction using the general method 9 detailed above in Chapter 3.6 to afford 102.2 mg of **4d** (44%). $^1$H NMR (300 MHz, DMSO-*d*$_6$) δ 12.17 (br, 1H), 8.08 (s, 1H), 7.55 – 7.45(m, 3H), 6.98 (d, *J* = 8.9 Hz, 2H), 6.30 (d, *J* = 15.9 Hz, 1H), 3.55 – 3.44 (m, 7H, contains $D_2O$ peak), 3.30 (t, *J* = 6.5 Hz, 2H), 3.24 (t, *J* = 6.5 Hz, 2H). $^{13}$C NMR (75 MHz, DMSO-*d*$_6$) δ 168.5, 161.4, 152.3, 144.5, 130.1, 125.0, 115.6, 48.7, 47.5. HRMS *m/z* calculated for $C_{13}H_{16}N_2O_2$ [M+H$^+$] 233.1285, found: 233.1290.

### (*E*)-3-(4-(4-(2-carboxyethyl)piperazin-1-yl)phenyl)acrylic acid (4dd)

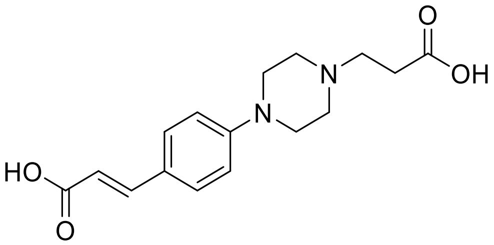


Side product **4dd** was found from the reaction which prepared from 17.0 mg (105 µmol) 1-phenylpiperazine (**4a**) using the general method 7 detailed above in Chapter 3.6 with halogenase PyrH. $^1$H NMR (300 MHz, DMSO-*d*$_6$) δ 11.75 (br, 2H), 7.57 (d, *J* = 8.4 Hz, 2H), 7.50 (d, *J* = 15.9 Hz, 1H), 7.02 (d, *J* = 8.5 Hz, 2H), 6.34 (d, *J* = 15.9 Hz, 1H), 3.44 – 3.34 (m, 8H), 2.79 (t, *J* = 7.4 Hz, 2H). $^{13}$C NMR (75 MHz, DMSO-*d*$_6$) δ 172.1, 168.4, 151.2, 144.3, 130.1, 125.8, 116.0, 115.7, 51.8, 51.3, 45.1, 29.2. HRMS *m/z* calculated for $C_{16}H_{21}N_2O_4$ [M+H$^+$] 305.1501, found: 305.1500.

### *p*-Morpholino-cinnamic acid (5d)

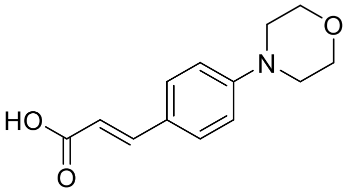


Compound **5d** was prepared from 17.0 mg (105 µmol) 4-phenylmorpholinee (**5a**) using the general method 7 detailed above in Chapter 3.6 with halogenase PyrH, and purified on silica gel with DCM/MeOH/TFA (50/1/10%) to afford 19.6 mg of a light-yellow solid (84 µmol, 80%). The larger-scale reaction using the general method 9 detailed above in Chapter 3.6 to afford 174.9 mg of **5d** (75%). $^1$H NMR (600 MHz, DMSO-*d*$_6$) δ 7.55 (d, *J* = 8.9 Hz, 2H), 7.50 (d, *J* = 15.9 Hz, 1H), 6.97 (d, *J* = 8.9 Hz, 2H), 6.30 (d, *J* = 15.9 Hz, 1H), 3.75 – 3.73 (m, 7H, contains $D_2O$ peak), 3.23 – 3.20 (m, 4H). $^{13}$C NMR (151 MHz, DMSO-*d*$_6$) δ 168.5, 152.6, 144.6, 130.0, 125.1, 115.3, 114.9, 66.3, 47.9. HRMS *m/z* calculated for $C_{13}H_{16}NO_3$ [M+H$^+$] 234.1125, found: 234.1127.

**(*E*)-3-(4-thiomorpholinophenyl)acrylic acid (6d)**

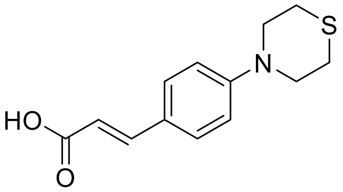


Compound **6d** was prepared from 18.8 mg (105 µmol) 4-phenylthiomorpholine (**6a**) using the general method 7 detailed above in Chapter 3.6 with halogenase PyrH, and purified on silica gel with DCM/MeOH/TFA (50/1/10%) to afford 15.4 mg of a yellow solid (62 µmol, 59%). $^{1}$H NMR (300 MHz, DMSO-$d_6$) δ 12.09 (br, 1H), 7.56 – 7.39 (m, 3H), 6.96 – 6.83 (m, 2H), 6.27 (d, *J* = 16.0 Hz, 1H), 3.76 – 3.61 (m, 4H), 2.67 – 2.58 (m, 4H). $^{13}$C NMR (75 MHz, DMSO-$d_6$) δ 168.5, 151.4, 144.6, 130.3, 124.0, 115.2, 114.8, 50.4, 25.3. HRMS *m/z* calculated for $C_{13}H_{16}NO_2S$ [M+H$^+$] 250.0896, found: 250.0903.

**(*E*)-3-(4-((3R,5S)-3,5-dimethylpiperazin-1-yl)phenyl)acrylic acid (7d)**

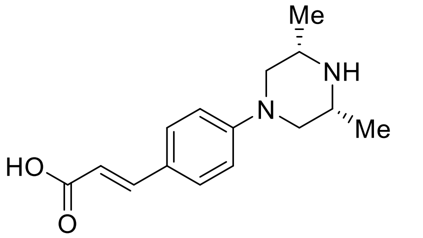


Compound **7d** was prepared from 19.9 mg (105 µmol) (3R,5S)-3,5-dimethyl-1-phenylpiperazine (**7a**) using the general method 7 detailed above in Chapter 3.6 with halogenase PrnA, and purified on silica gel with DCM/MeOH/TFA (10/1/3%) to afford 3 mg of a brown solid (12 µmol, 11%). $^{1}$H NMR (300 MHz, DMSO-$d_6$) δ 12.16 (s, 1H), 7.57 (d, *J* = 8.8 Hz, 2H), 7.50 (d, *J* = 15.9 Hz, 1H), 7.08 – 7.00 (m, 2H), 6.34 (d, *J* = 16.0 Hz, 1H), 4.11 – 3.88 (m, 3H), 2.80 – 2.59 (m, 3H), 1.27 (s, 3H), 1.25 (s, 3H). $^{13}$C NMR (75 MHz, DMSO-$d_6$) δ 168.4, 151.0, 144.3, 132.4, 130.1, 125.5, 115.9, 115.6, 114.8, 51.0, 50.7, 16.2. HRMS *m/z* calculated for $C_{15}H_{21}N_2O_2$ [M+H$^+$] 261.1598, found: 261.1599.

**(*E*)-3-(4-(4-methyl-1,4-diazepan-1-yl)phenyl)acrylic acid (8d)**

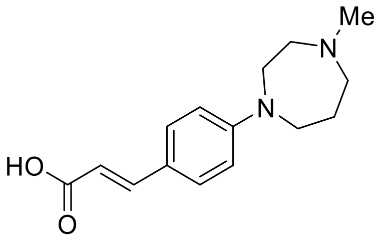


Compound **8d** was prepared from 19.9 mg (105 µmol) 1-methyl-4-phenyl-1,4-diazepane (**8a**) using the general method 7 detailed above in Chapter 3.6 with halogenase RebH, and purified on silica gel with DCM/MeOH/TFA (5/1/5%) to afford 10.4 mg of a dark-red solid (40 µmol, 38%). $^{1}$H NMR (300 MHz, $CD_3OD$) δ 7.60 (d, *J* = 15.9 Hz, 1H), 7.51 (d, *J* = 8.6 Hz, 2H), 6.86 (d, *J* = 8.6 Hz, 2H), 6.26 (d, *J* = 15.9 Hz, 1H), 3.62 (d, *J* = 5.6 Hz, 3H), 3.51 (d, *J* = 7.0 Hz, 2H), 3.30 – 3.23 (m, 2H), 3.07 – 2.99 (m, 2H), 2.30 (s, 3H), 1.42 – 1.30 (m, 2H). $^{13}$C NMR (75 MHz,

$CD_3OD$) δ 169.8, 150.1, 145.3, 129.8, 123.5, 112.9, 111.9, 56.9, 55.9, 46.6, 43.6, 24.4. HRMS *m/z* calculated for $C_{15}H_{21}N_2O_2$ [M+H$^+$] 261.1598, found: 261.1595.

### (*E*)-3-(2-aminonaphthalen-1-yl)acrylic acid (9d)

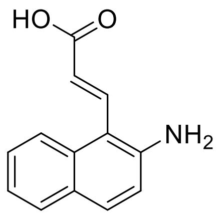


Compound **9d** was prepared from 15.0 mg (105 µmol) 2-Naphthylamine (**9a**) using the general method 7 detailed above in Chapter 3.6 with halogenase RebH, and purified on silica gel with DCM/MeOH/TFA (10/1/1%) to afford 15.0 mg of a brown solid (70 µmol, 67%). The larger-scale reaction using the general method 9 detailed above in Chapter 3.6 to afford 78.9 mg of **5d** (37%). $^1$H NMR (300 MHz, DMSO-$d_6$) δ 12.12 (s, 1H), 8.79 (d, *J* = 16.3 Hz, 1H), 8.52 (d, *J* = 8.5 Hz, 1H), 8.03 (d, *J* = 8.9 Hz, 1H), 7.95 (dd, *J* = 8.0, 1.4 Hz, 1H), 7.67 (ddd, *J* = 8.4, 6.9, 1.4 Hz, 1H), 7.57 – 7.43 (m, 2H), 6.64 (d, *J* = 16.3 Hz, 1H). $^{13}$C NMR (75 MHz, DMSO-$d_6$) δ 162.3, 138.7, 136.4, 132.1, 129.7, 129.3, 129.2, 128.3, 125.4, 122.2, 121.5, 116.8, 113.1. HRMS *m/z* calculated for $C_{13}H_{12}NO_2$ [M+H$^+$] 214.0863, found: 214.0870.

### (*E*)-3-(1-hydroxynaphthalen-2-yl)acrylic acid (10d)

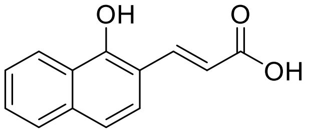


Compound **10d** was prepared from 15.0 mg (105 µmol) 1-naphthol (**10a**) using the general method 7 detailed above in Chapter 3.6 with halogenase RebH, and purified on silica gel with DCM/MeOH/TFA (40/1/5%) to afford 2.7 mg of a light-yellow solid (12.6 µmol, 12%). $^1$H NMR (300 MHz, DMSO-$d_6$) δ 10.75 (s, 1H), 8.21 (d, *J* = 16.0 Hz, 1H), 8.13 – 8.01 (m, 1H), 7.83 (d, *J* = 8.6 Hz, 2H), 7.53 (ddd, *J* = 8.4, 6.8, 1.5 Hz, 1H), 7.35 (ddd, *J* = 8.0, 6.9, 1.0 Hz, 1H), 7.26 (d, *J* = 8.9 Hz, 1H), 6.79 (d, *J* = 16.0 Hz, 1H). $^{13}$C NMR (75 MHz, DMSO) δ 169.0, 156.5, 137.3, 133.1, 132.0, 129.2, 128.5, 127.9, 123.6, 122.9, 122.5, 118.8, 112.8. HRMS *m/z* calculated for $C_{13}H_{11}O_3$ [M+H$^+$] 215.0703, found: 215.0711.

### (*E*)-3-(4-hydroxynaphthalen-1-yl)acrylic acid (10d′)

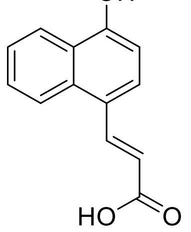

Compound **10d′** was prepared from 15.0 mg (105 µmol) 1-naphthol (**10a**) using the general method 8 detailed above in Chapter 3.6 with halogenase *Am*VHPO, and purified on silica gel with DCM/MeOH/TFA (40/1/5%) to afford 9.7 mg of a yellow solid (45 µmol, 43%). $^{1}$H NMR (300 MHz, $CD_3OD$) δ 8.39 (dd, *J* = 15.7, 2.5 Hz, 1H), 8.29 (dd, *J* = 8.4, 1.5 Hz, 1H), 8.15 (d, *J* = 8.5 Hz, 1H), 7.76 (d, *J* = 8.0 Hz, 1H), 7.60 (ddd, *J* = 8.5, 6.9, 1.5 Hz, 1H), 7.50 (ddd, *J* = 8.1, 6.8, 1.2 Hz, 1H), 6.88 (d, *J* = 8.0 Hz, 1H), 6.40 (d, *J* = 15.6 Hz, 1H). $^{13}$C NMR (75 MHz, $CD_3OD$) δ 167.4, 156.0, 140.6, 132.8, 126.9, 126.0, 124.9, 124.4, 122.7, 122.2, 122.1, 117.9, 107.6. HRMS *m/z* calculated for $C_{13}H_{11}O_3$ [M+H$^+$] 215.0703, found: 215.0707.

**(*E*)-3-(3-(2-hydroxyethyl)-1H-indol-7-yl)acrylic acid (11d)**

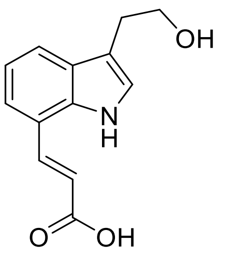


Compound **11d** was prepared from 16.9 mg (105 µmol) tryptophol (**11a**) using the general method 7 detailed above in Chapter 3.6 with halogenase RebH, and purified on silica gel with DCM/MeOH/TFA (10/1/5%) to afford 14.1 mg of a yellow oil (61 µmol, 58%). The larger-scale reaction using the general method 9 detailed above in Chapter 3.6 to afford 122.6 mg of **11d** (53%). $^{1}$H NMR (300 MHz, DMSO-$d_6$) δ 11.43 (s, 1H), 8.09 (d, *J* = 16.0 Hz, 1H), 7.68 (d, *J* = 7.7 Hz, 1H), 7.53 (d, *J* = 7.4 Hz, 1H), 7.29 (d, *J* = 2.5 Hz, 1H), 7.07 (t, *J* = 7.6 Hz, 1H), 6.65 – 6.53 (m, 1H), 4.61 (t, *J* = 6.8 Hz, 2H), 3.16 (t, *J* = 6.9 Hz, 2H). $^{13}$C NMR (75 MHz, DMSO-$d_6$) δ 168.3, 140.6, 135.0, 128.3, 124.9, 121.3, 121.2, 119.4, 119.0, 118.7, 110.4, 66.8, 24.1. HRMS *m/z* calculated for $C_{13}H_{14}NO_3$ [M+H$^+$] 232.0968, found: 232.0976.

**(*E*)-3-(3-(2-aminoethyl)-1H-indol-7-yl)acrylic acid (12d)**

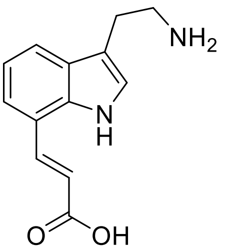


Compound **12d** was prepared from 16.9 mg (105 µmol) tryptophane (**12a**) using the general method 7 detailed above in Chapter 3.6 with halogenase PrnA, and purified on silica gel with DCM/MeOH/TFA (3/1/1%) to afford 9.7 mg of a dark-yellow solid (42 µmol, 40%). $^{1}$H NMR (400 MHz, $CD_3OD$) δ 8.11 (d, *J* = 16.0 Hz, 1H), 7.64 (dd, *J* = 7.9, 1.0 Hz, 1H), 7.42 (d, *J* = 7.4 Hz, 1H), 7.16 (s, 1H), 7.08 (t, *J* = 7.7 Hz, 1H), 6.56 (d, *J* = 16.0 Hz, 1H), 3.82 (t, *J* = 7.2 Hz, 2H), 2.98 (td, *J* = 7.2, 0.9 Hz, 2H). $^{13}$C NMR (151 MHz, $CD_3OD$) δ 170.8, 142.9, 136.4, 130.3, 124.6, 122.3, 122.2, 112.0, 119.5, 118.5, 113.7, 63.5, 29.6. HRMS *m/z* calculated for $C_{13}H_{15}N_2O_2$ [M+H$^+$] 231.1128, found: 231.1134.

**(*E*)-3-(2,3,4,9-tetrahydro-1H-pyrido[3,4-b]indol-6-yl)acrylic acid (13d)**

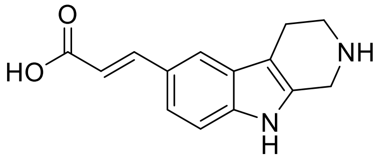


Compound **13d** was prepared from 18.0 mg (105 µmol) tetrahydrobetacarboline (**13a**) using the general method 7 detailed above in Chapter 3.6 with halogenase RebH, and purified on flash chromatography with DCM/MeOH/TFA (3/1/1%) to afford 9.9 mg of a dark-brown oil (42 µmol, 39%). $^1$H NMR (300 MHz, $CD_3OD$) δ 7.82 (d, *J* = 15.9 Hz, 1H), 7.76 (s, 1H), 7.55 – 7.36 (m, 2H), 6.44 (d, *J* = 15.9 Hz, 1H), 4.47 (s, 2H), 3.63 (t, *J* = 6.1 Hz, 2H), 3.14 (t, *J* = 6.1 Hz, 2H). $^{13}$C NMR (75 MHz, $CD_3OD$) δ 169.6, 146.8, 138.1, 136.7, 126.5, 126.2, 121.7, 119.3, 114.5, 111.5, 106.7, 42.3, 40.8, 18.0. HRMS *m/z* calculated for $C_{14}H_{15}N_2O_2$ [M+H$^+$] 243.1128, found: 243.1139.

**(*E*)-3-(7-hydroxy-2-oxo-2H-chromen-8-yl)acrylic acid (16d)**

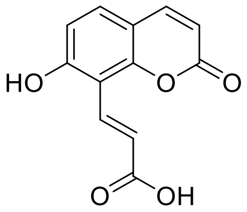


Compound **16d** was prepared from 17.0 mg (105 µmol) 7-methylcoumarin (**16a**) using the general method 8 detailed above in Chapter 3.6 with halogenase *Am*VHPO, and purified on flash chromatography with DCM/MeOH/TFA (10/1/5%) to afford 4.6 mg of a bright yellow solid (20 µmol, 19%). $^1$H NMR (300 MHz, DMSO-*d*$_6$) δ 12.38 (s, 1H), 11.52 (s, 1H), 7.99 (d, *J* = 2.8 Hz, 1H), 7.95 (d, *J* = 16.3 Hz, 1H), 7.58 (d, *J* = 8.6 Hz, 1H), 7.00 – 6.89 (m, 2H), 6.30 (d, *J* = 16.3 Hz, 1H). $^{13}$C NMR (75 MHz, DMSO-*d*$_6$) δ 168.7, 161.3,161.1 160.3, 154.33, 145.44, 133.2, 131.3, 123.2, 123.1, 112.0, 111.9. HRMS *m/z* calculated for $C_{12}H_9O_5$ [M+H$^+$] 233.0444, found: 233.0451.

**(*E*)-3-(2,4-dimethoxyphenyl)acrylic acid (17d)**

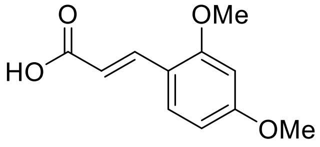


Compound **17d** was prepared from 14.5 mg (105 µmol) 1,3-dimethoxybenzene (**17a**) using the general method 8 detailed above in Chapter 3.6 with halogenase *Am*VHPO, and purified on silica gel with PE/EA (8/1) to afford 18.2 mg of a yellow solid (81 µmol, 83%). The larger-scale reaction using the general method 10 detailed above in Chapter 3.6 to afford 177.6 mg of **17d** (85%). $^1$H NMR (300 MHz, DMSO-*d*$_6$) δ 12.11 (s, 1H), 7.74 (d, *J* = 16.1 Hz, 1H), 7.61 (d, *J* = 8.5 Hz, 1H), 6.65 – 6.51 (m, 2H), 6.37 (d, *J* = 16.1 Hz, 1H), 3.86 (s, 3H), 3.81 (s, 3H). HRMS *m/z* calculated for $C_{11}H_{13}O_4$ [M+H$^+$] 209.0808, found: 209.0811. The analytical data is in agreement with the literature.[14]

### (*E*)-3-(2,4,6-trimethoxyphenyl)acrylic acid (18d)

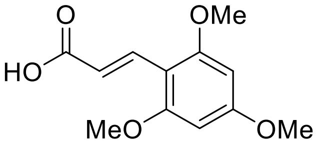


Compound **18d** was prepared from 17.6 mg (105 µmol) 1,3,5-trimethoxybenzene (**18a**) using the general method 8 detailed above in Chapter 3.6 with halogenase *Am*VHPO, and purified on silica gel with PE/EA (8/1) to afford 16.5 mg of a yellow solid (69 µmol, 66%). $^1$H NMR (300 MHz, DMSO-$d_6$) δ 11.87 (s, 1H), 7.86 (d, *J* = 16.2 Hz, 1H), 6.55 (d, *J* = 16.2 Hz, 1H), 6.28 (s, 2H), 3.85 (s, 6H), 3.83 (s, 3H). HRMS *m/z* calculated for $C_{12}H_{15}O_4$ [M+H$^+$] 239.0914, found: 239.0917. The analytical data is in agreement with the literature.[15]

### (*E*)-3-(4-hydroxy-5-isopropyl-2-methylphenyl)acrylic acid (19d)

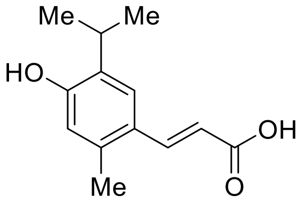


Compound **19d** was prepared from 15.7 mg (105 µmol) thymol (**19a**) using the general method 8 detailed above in Chapter 3.6 with halogenase *Am*VHPO, and purified on silica gel with PE/EA/TFA (1/1/5%) to afford 10.9 mg of a brown oil (49 µmol, 47%). The larger-scale reaction using the general method 10 detailed above in Chapter 3.6 to afford 112.3 mg of **19d** (51%). $^1$H NMR (300 MHz, DMSO-$d_6$) δ 9.74 (s, 1H), 7.72 (d, *J* = 15.8 Hz, 1H), 7.44 (s, 1H), 6.71 – 6.55 (m, 1H), 6.25 (d, *J* = 15.8 Hz, 1H), 3.14 (p, *J* = 6.9 Hz, 1H), 2.26 (s, 3H), 1.18 (s, 3H), 1.15 (s, 3H). $^{13}$C NMR (75 MHz, DMSO-$d_6$) δ 168.5, 157.0, 142.0, 136.8, 133.2, 125.1, 124.0, 117.3, 116.2, 26.7, 22.8, 19.4. HRMS *m/z* calculated for $C_{13}H_{17}O_3$ [M+H$^+$] 221.1172, found: 221.1175.

### *p*-dimethylaminocinnamic acid (20d)

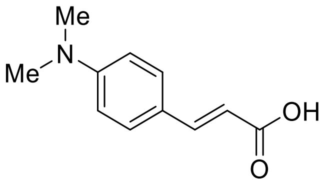


Compound **20d** was prepared from 12.7 mg (105 µmol) *N,N*-dimethylaniline (**20a**) using the general method 8 detailed above in Chapter 3.6 with halogenase *Am*VHPO, and purified on silica gel with PE/EA (5/1) to afford 15.5 mg of a brown oil (81 µmol, 77%). The larger-scale reaction using the general method 10 detailed above in Chapter 3.6 to afford 135.8 mg of **17d** (71%). $^1$H NMR (300 MHz, DMSO-$d_6$) δ 7.57 – 7.42 (m, 3H), 6.77 (d, *J* = 8.8 Hz, 2H), 6.23 (d, *J* = 15.9 Hz, 1H), 2.97 (s, 6H). HRMS m/z calculated for $C_{11}H_{14}NO_2$ [M+H$^+$] 192.1019, found: 192.1017. The analytical data is in agreement with the literature.[16]

**(*E*)-3-(9H-carbazol-3-yl)acrylic acid (21d)**

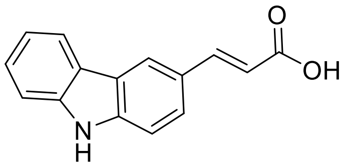


Compound **21d** was prepared from 17.5 mg (105 µmol) 9H-carbazole (**21a**) using the general method 8 detailed above in Chapter 3.6 with halogenase *Am*VHPO, and purified on silica gel with PE/EA/TFA (5/1/1%) to afford 10.4 mg of a brown oil (44 µmol, 42%). $^{1}$H NMR (300 MHz, DMSO-*d*$_6$) δ 12.14 (br, 1H), 11.51 (s, 1H), 8.50 (s, 1H), 8.17 (d, *J* = 7.8 Hz, 1H), 7.84 – 7.69 (m, 2H), 7.55 – 7.46 (m, 2H), 7.41 (ddd, *J* = 8.2, 7.0, 1.2 Hz, 1H), 7.20 (ddd, *J* = 8.0, 7.0, 1.1 Hz, 1H), 6.52 (d, *J* = 15.9 Hz, 1H). $^{13}$C NMR (75 MHz, DMSO-*d*$_6$) δ 168.0, 145.5, 141.0, 140.2, 126.1, 125.9, 125.0, 122.8, 122.4, 122.3, 121.2, 120.5, 119.2, 115.4, 111.3. HRMS *m/z* calculated for $C_{15}H_{12}NO_2$ [M+H$^+$] 238.0863, found: 238.0862.

**(*E*)-3-(2,6-dimethoxypyridin-3-yl)acrylic acid (22d)**

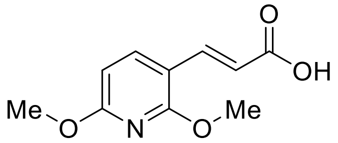


Compound **22d** was prepared from 14.5 mg (105 µmol) 2,6-dimethoxypyridine (**22a**) using the general method 8 detailed above in Chapter 3.6 with halogenase *Am*VHPO, and purified on flash chromatography with PE/EA/TFA (3/1/5%) to afford 5.8 mg of a white solid (27 µmol, 26%). The larger-scale reaction using the general method 10 detailed above in Chapter 3.6 to afford 62.8 mg of **17d** (30%). $^{1}$H NMR (300 MHz, DMSO-*d*$_6$) δ 12.18 (br, 1H), 8.03 (d, *J* = 8.3 Hz, 1H), 7.64 (d, *J* = 16.1 Hz, 1H), 6.52 – 6.36 (m, 2H), 3.97 (s, 3H), 3.91 (s, 3H). $^{13}$C NMR (75 MHz, DMSO-*d*$_6$) δ 168.4, 164.0, 161.0, 141.5, 138.1, 117.9, 109.2, 102.7, 54.1, 54.1. HRMS *m/z* calculated for $C_{10}H_{12}NO_4$ [M+H$^+$] 210.0761, found: 210.0765.

# 5. NMR spectra

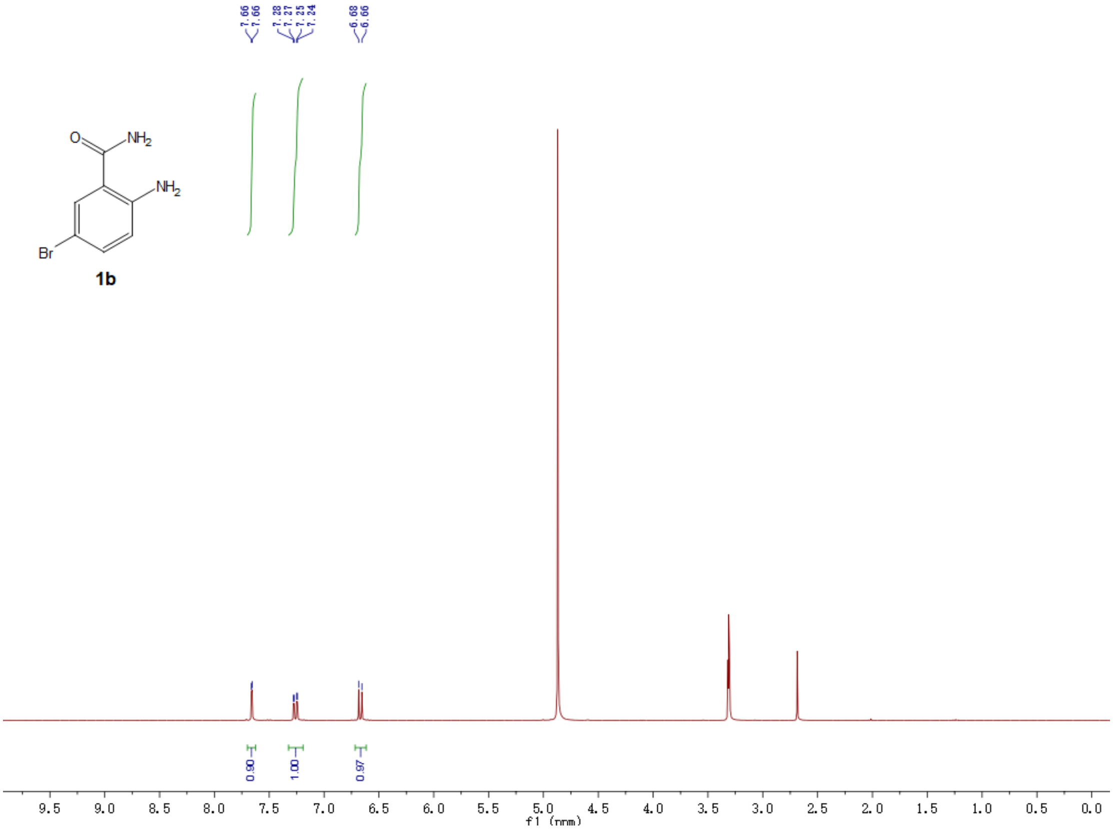


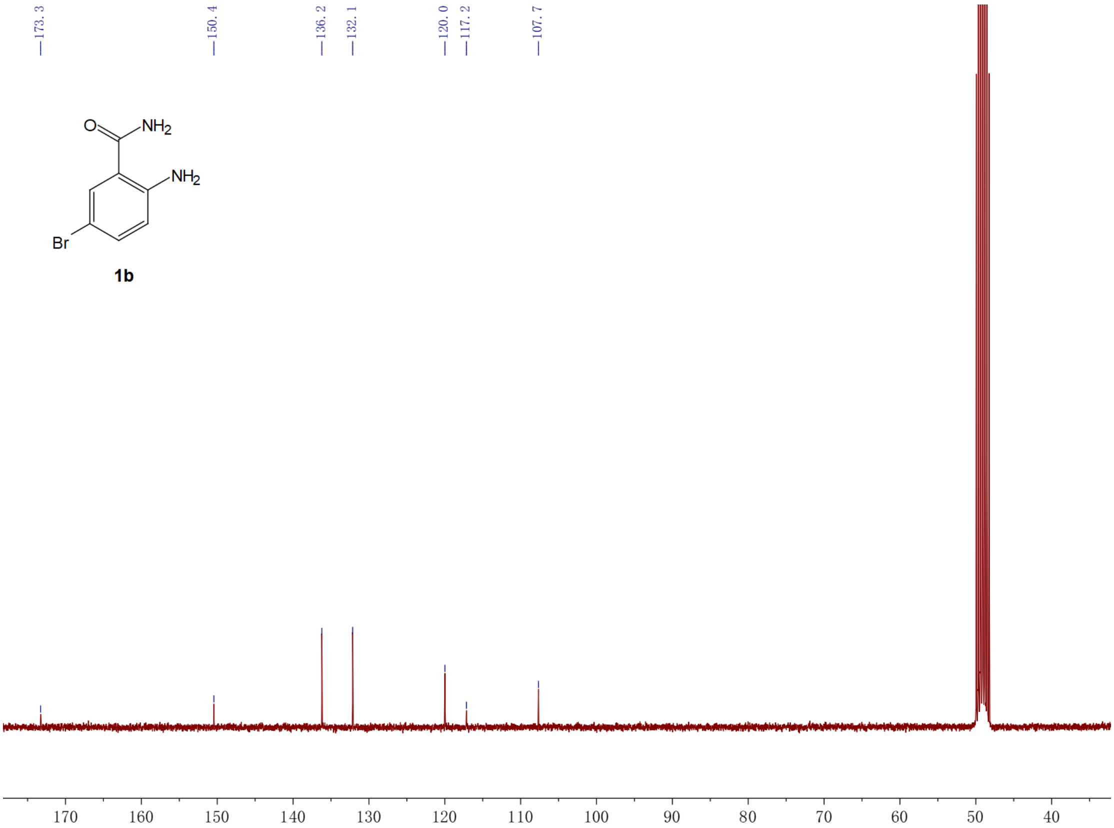


**Figure S6.** $^{1}H$ NMR and $^{13}C$ NMR of 2-amino-5-bromobenzamide (**1b**) recorded in $CD_3OD$.

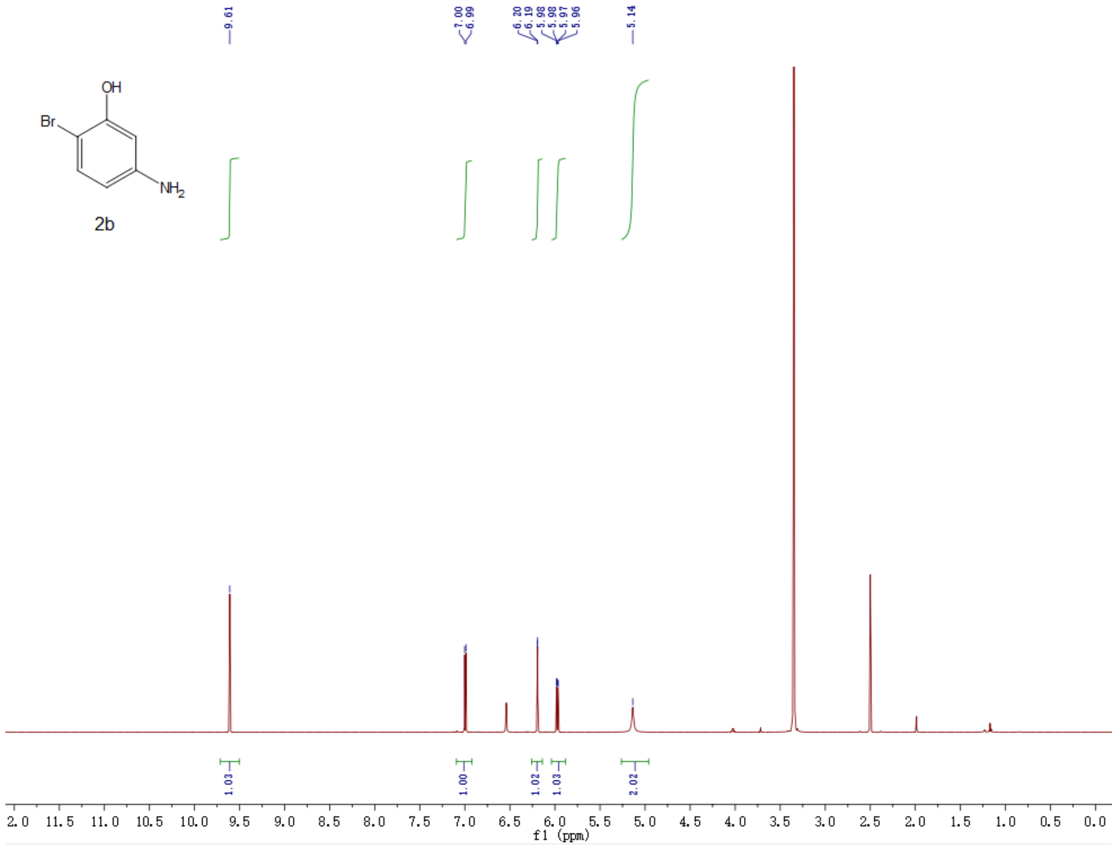


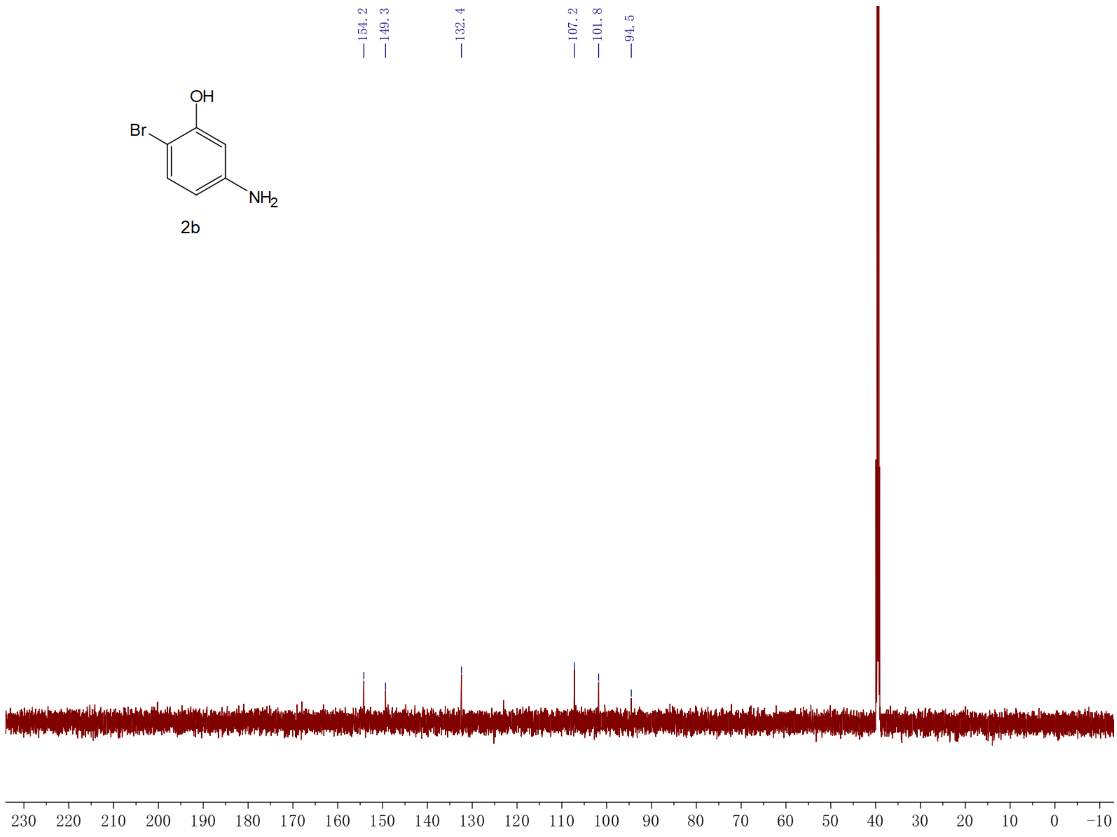


**Figure S7.** $^{1}H$ NMR and $^{13}C$ NMR of 5-amino-2-bromophenol (**2b**) recorded in DMSO-$d_6$.

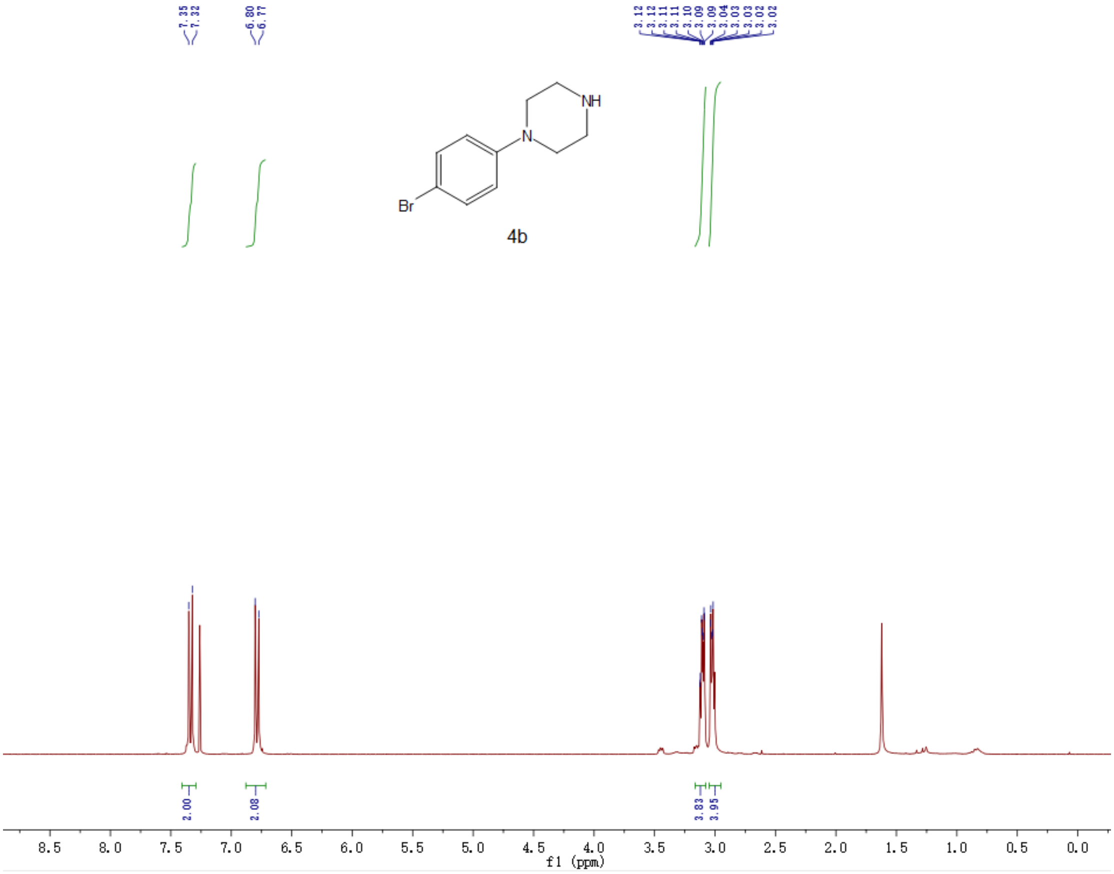


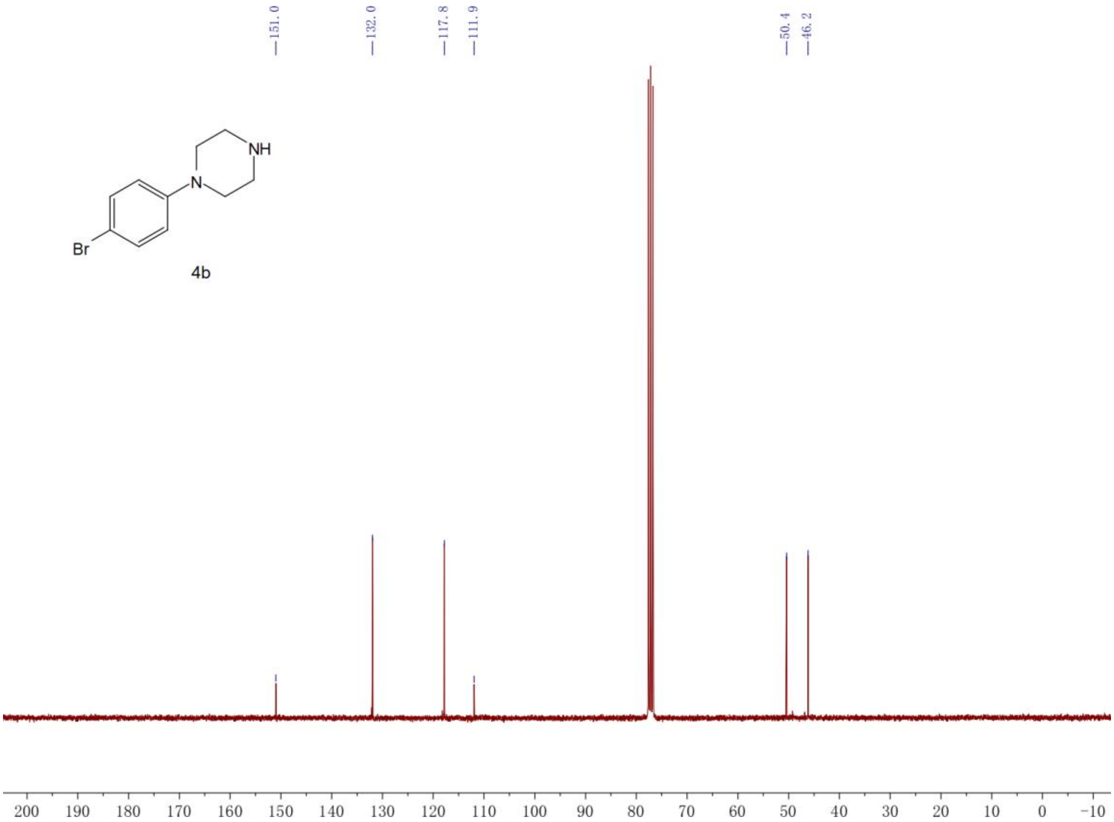


**Figure S8.** $^{1}H$ NMR and $^{13}C$ NMR of 1-(4-bromophenyl)piperazine (**4b**) recorded in $CDCl_3$.

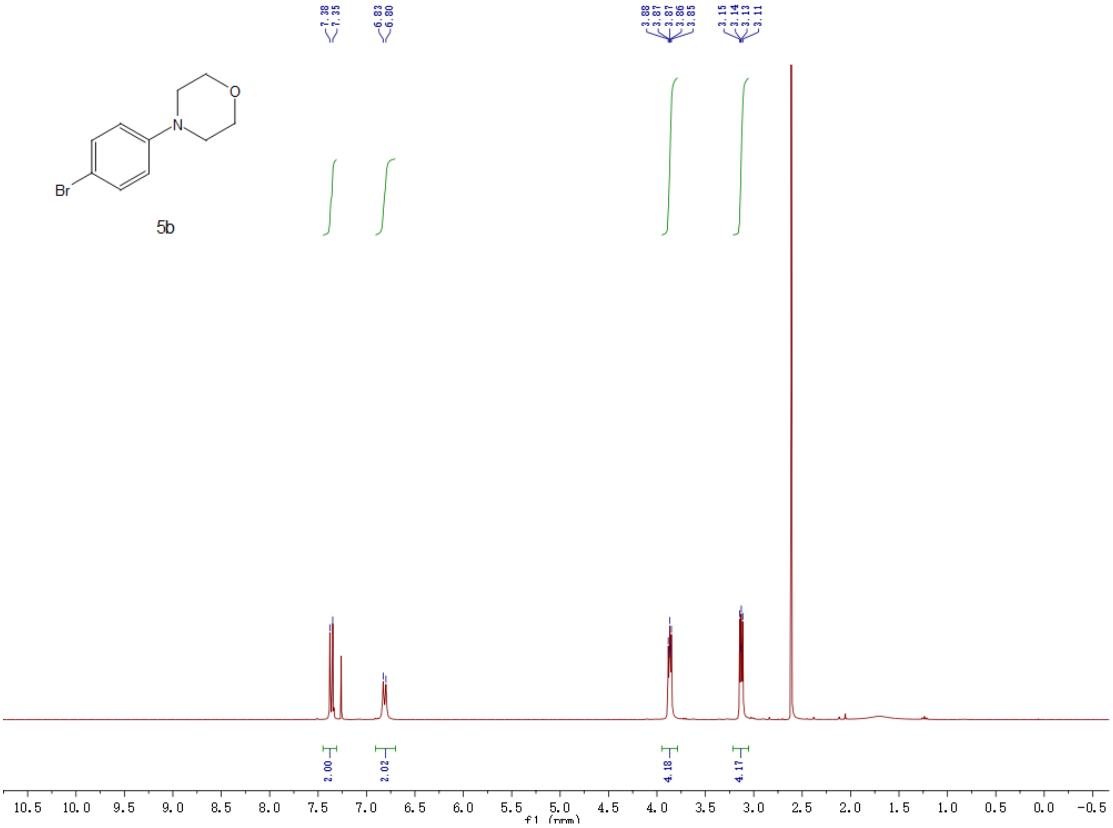

**Figure S9.** $^1H$ NMR of 4-(4-bromophenyl)morpholine (**5b**) recorded in $CDCl_3$.

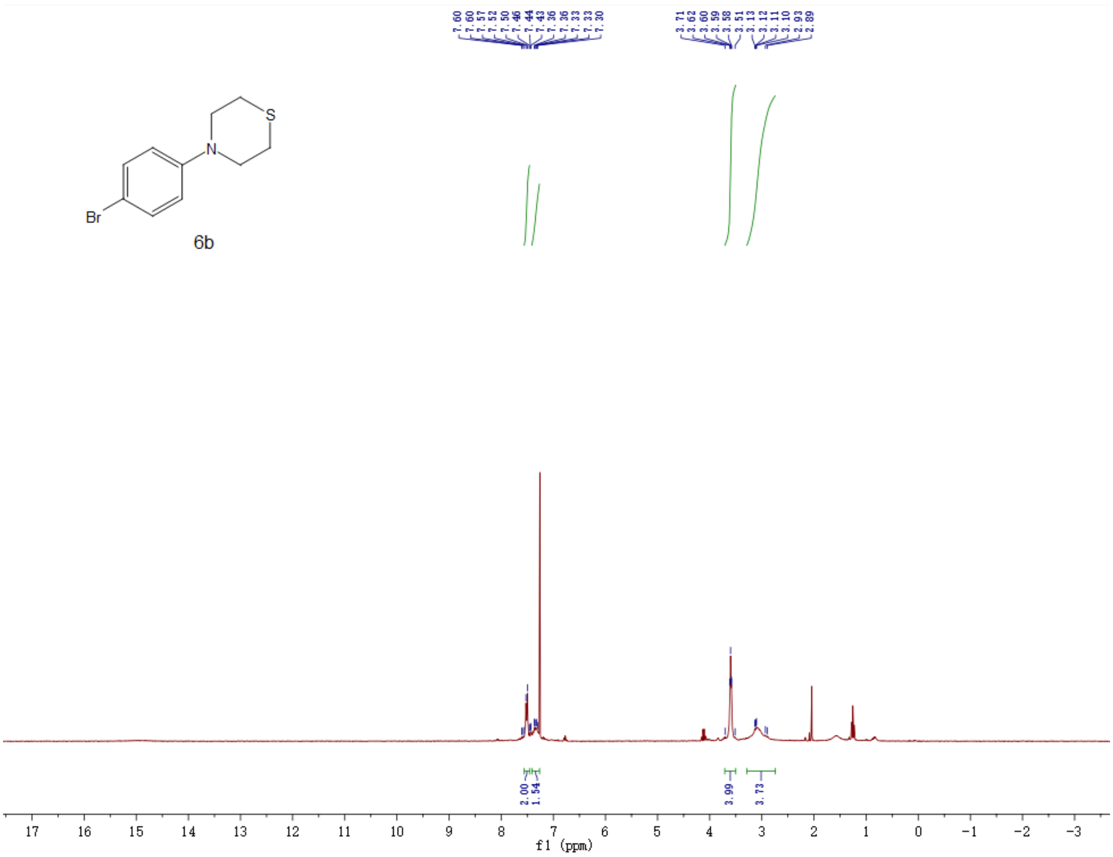


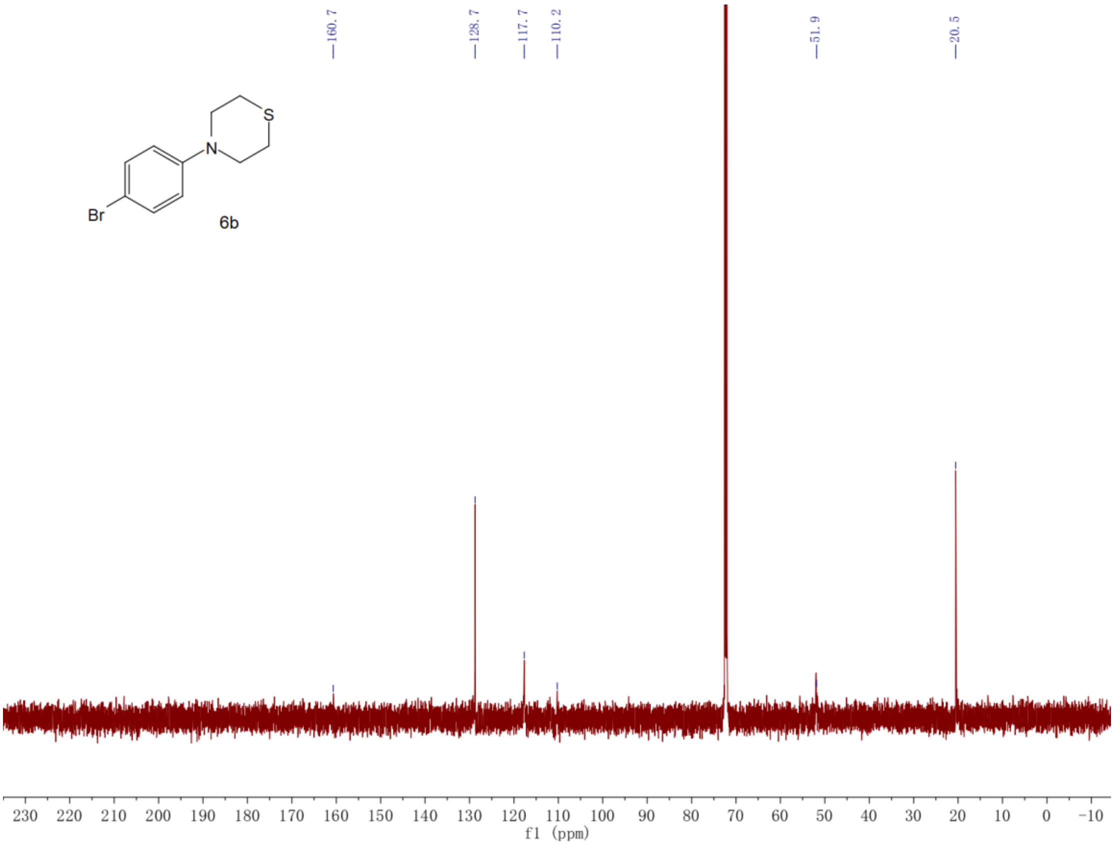


**Figure S10.** $^{1}H$ NMR and $^{13}C$ NMR of 4-(4-bromophenyl)thiomorpholine (**6b**) recorded in $CDCl_3$ and DMSO-$d_6$..

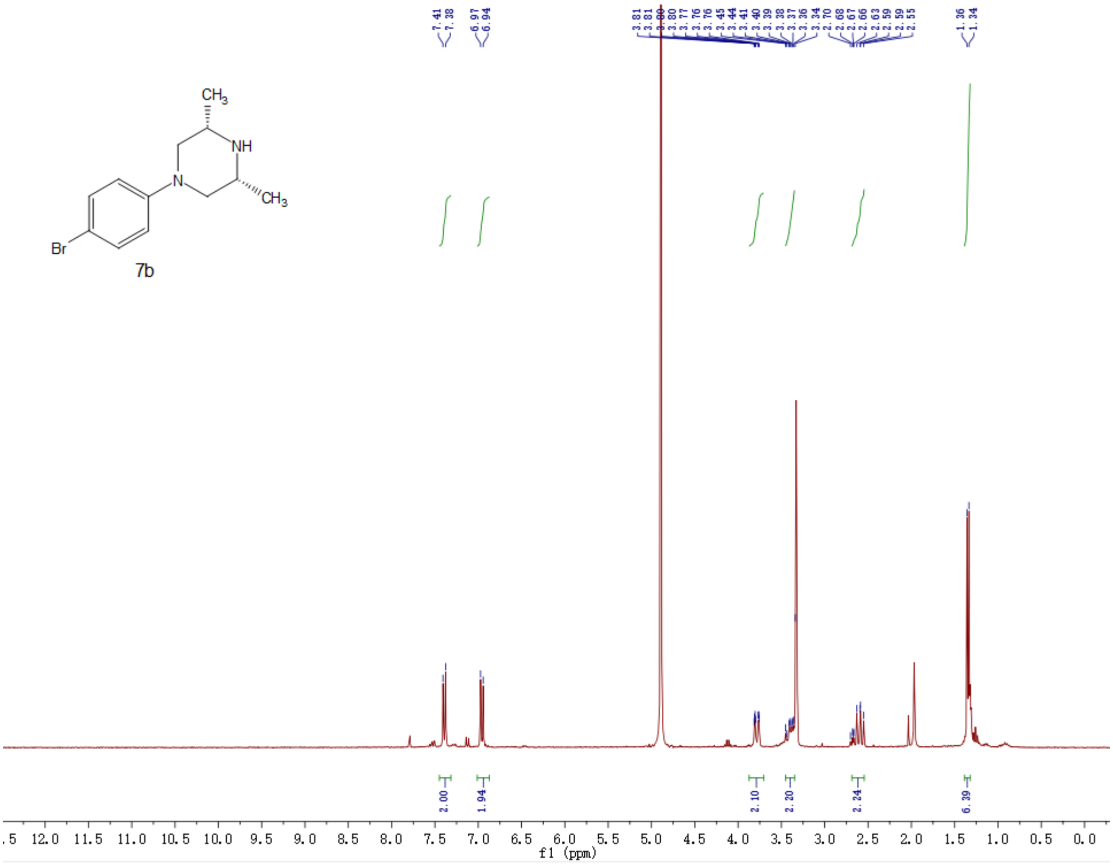

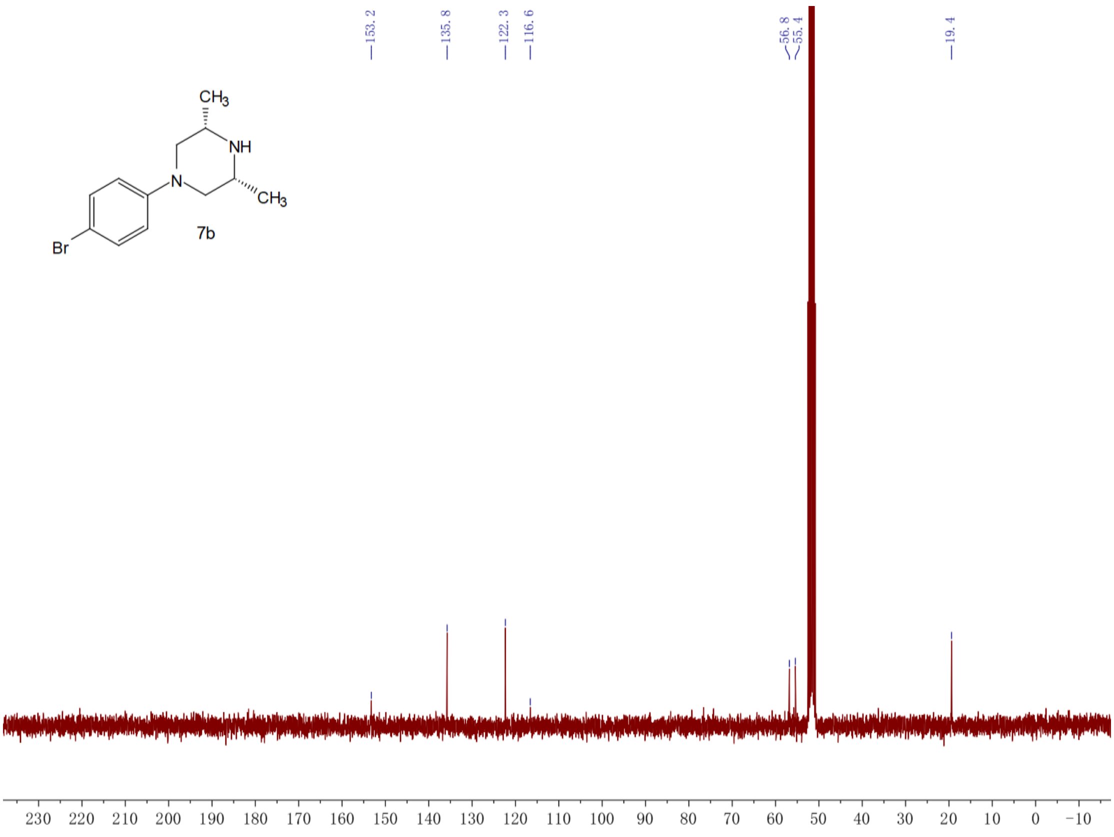

**Figure S11.** $^{1}H$ NMR and $^{13}C$ NMR of (3R,5S)-1-(4-bromophenyl)-3,5-dimethylpiperazine (**7b**) recorded in $CD_3OD$.

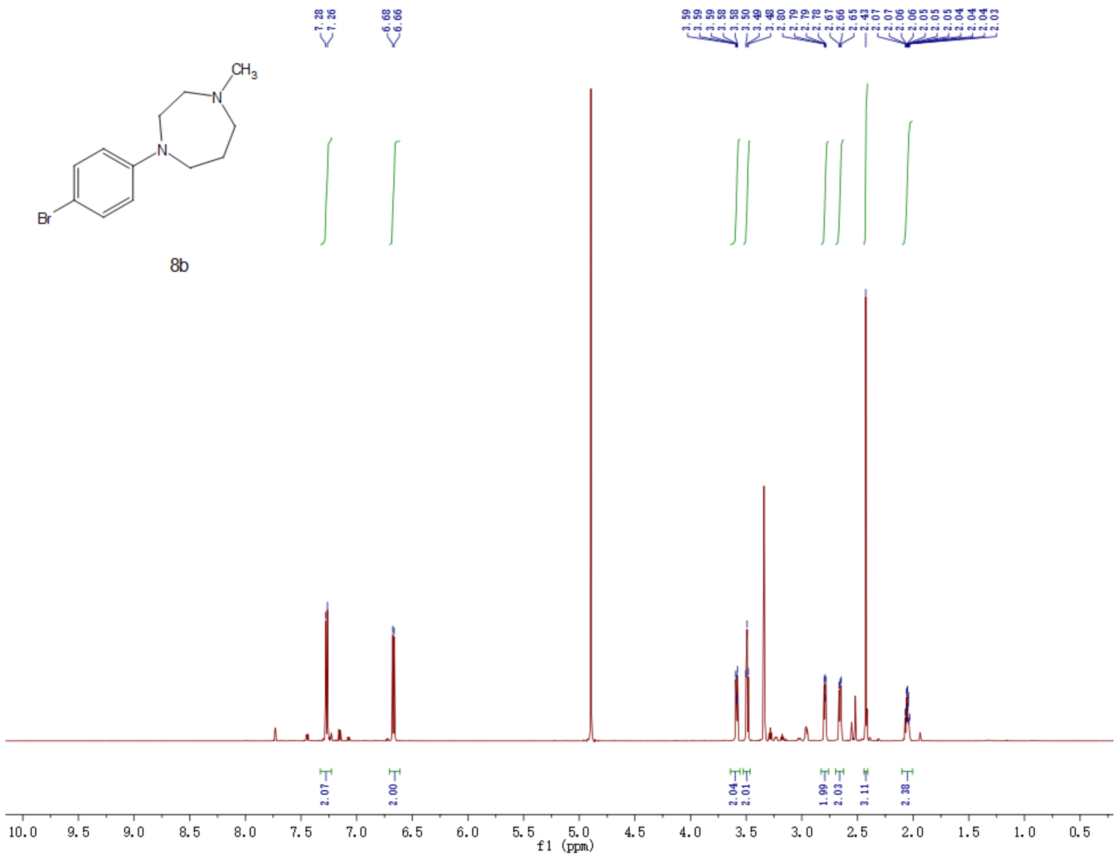

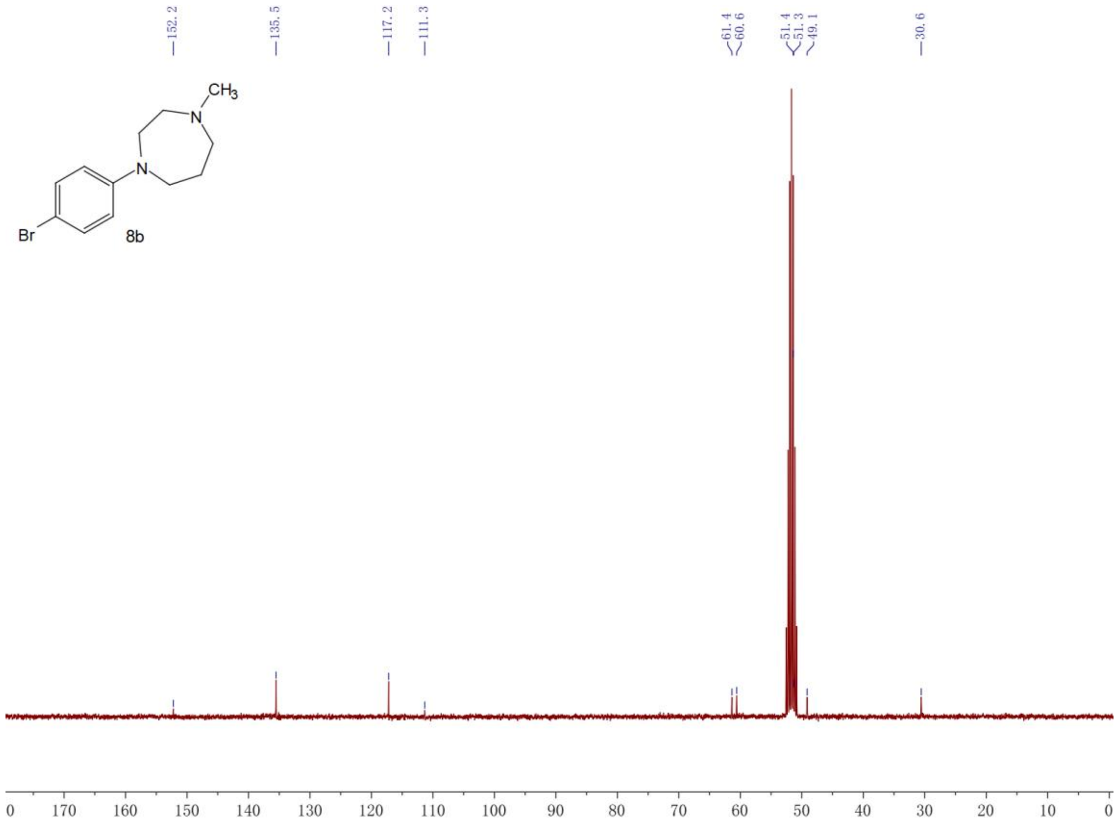

**Figure S12.** $^{1}H$ NMR and $^{13}C$ NMR of 1-(4-bromophenyl)-4-methyl-1,4-diazepane (**8b**) recorded in $CD_3OD$ and $CDCl_3$.

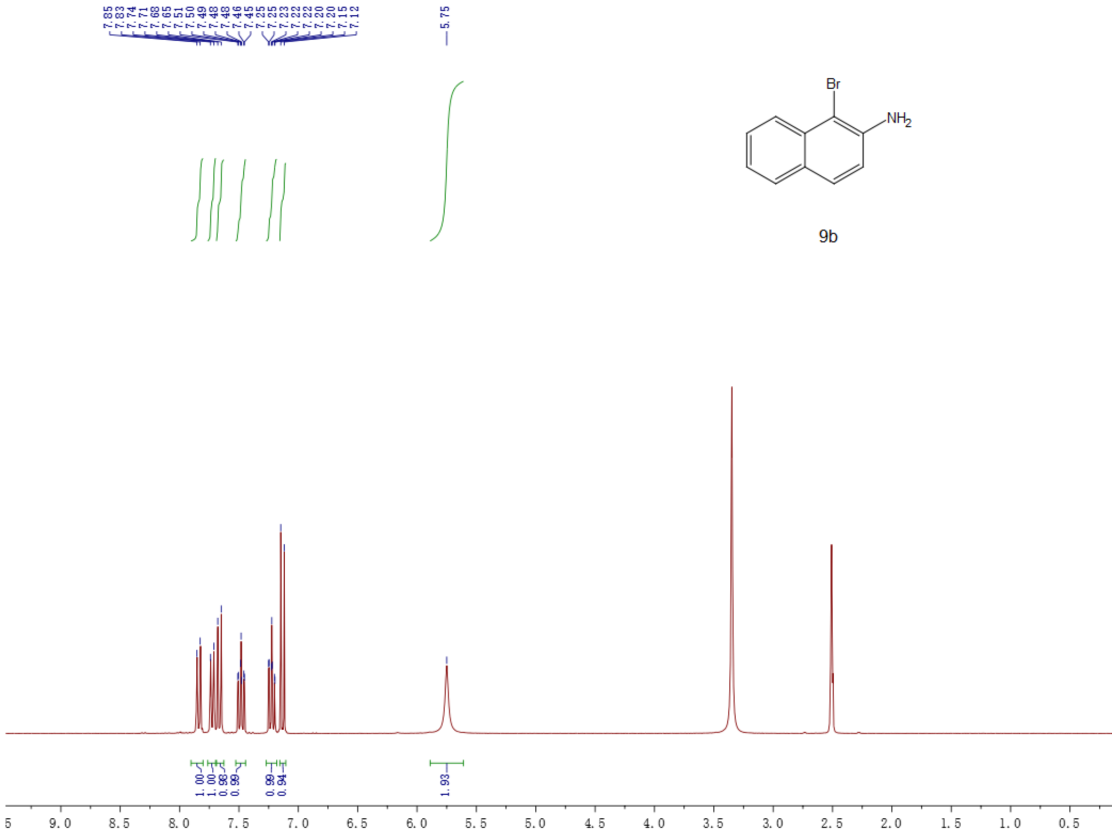


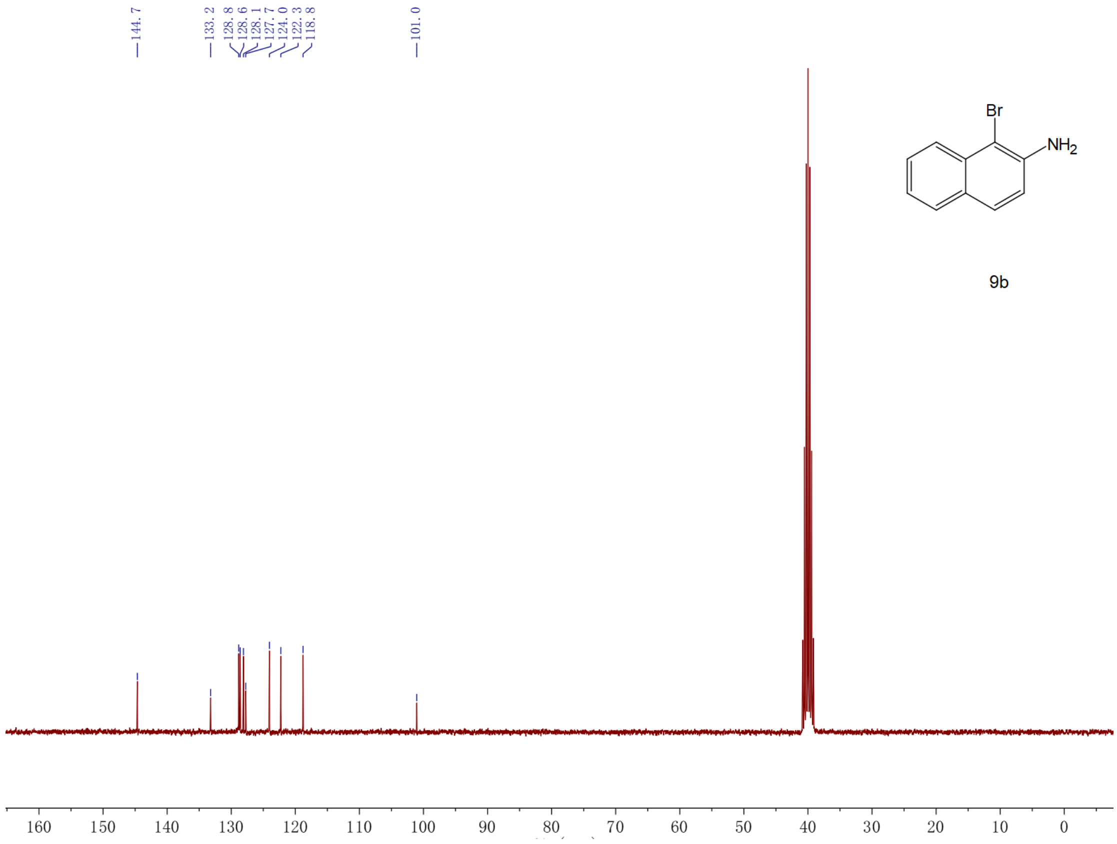


**Figure S13.** $^{1}H$ NMR and $^{13}C$ NMR of 1-bromonaphthalen-2-amine (**9b**) recorded in DMSO-$d_6$.

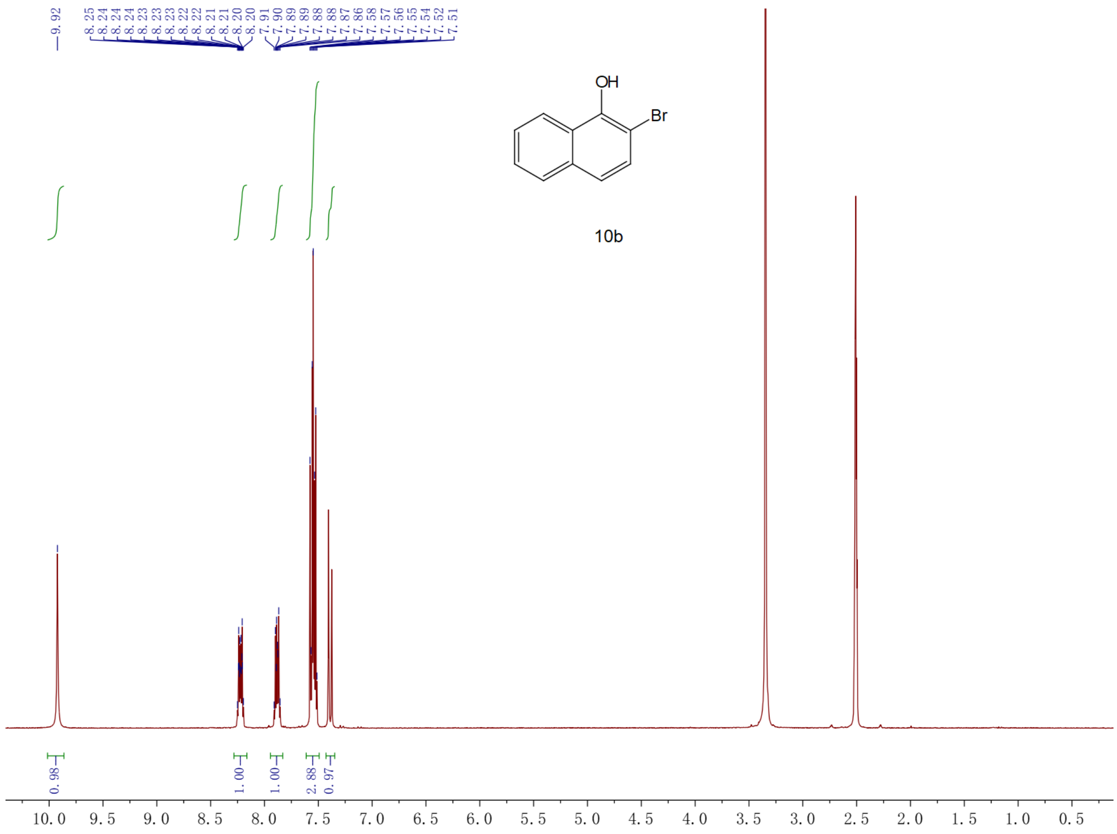

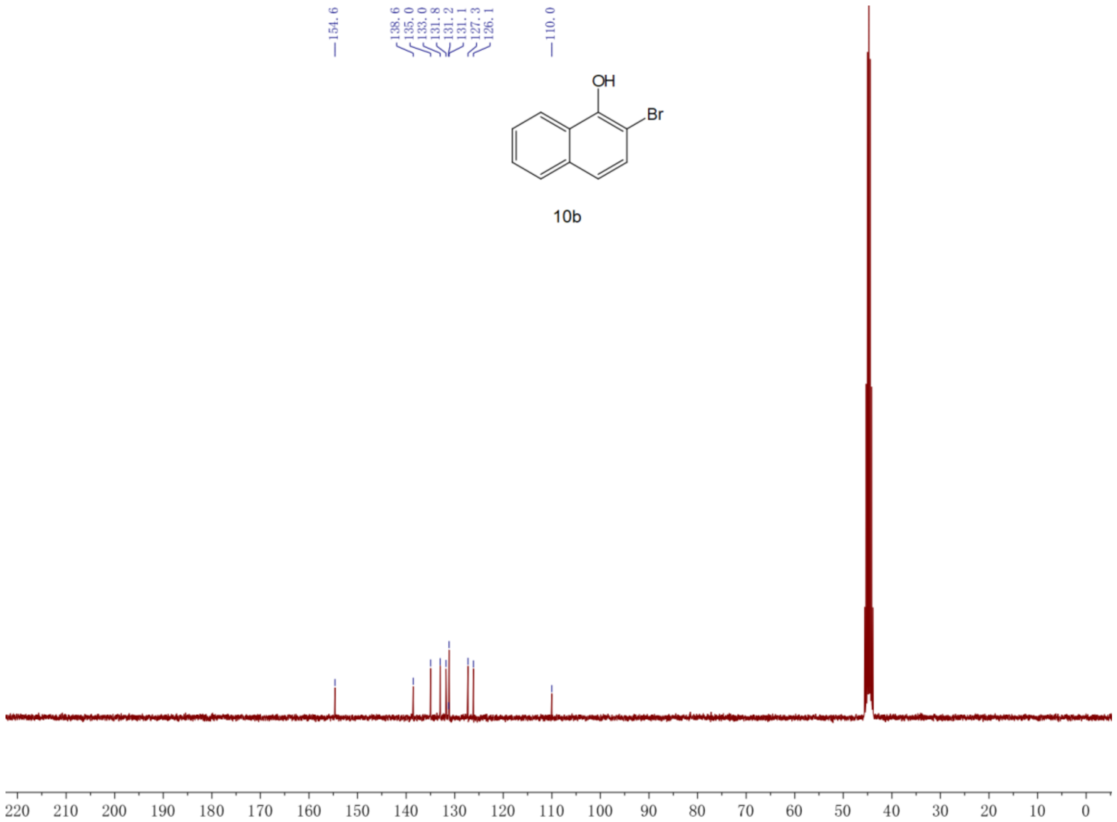

**Figure S14.** $^{1}H$ NMR and $^{13}C$ NMR of 2-bromo-1-naphthol (**10b**) recorded in DMSO-$d_6$..

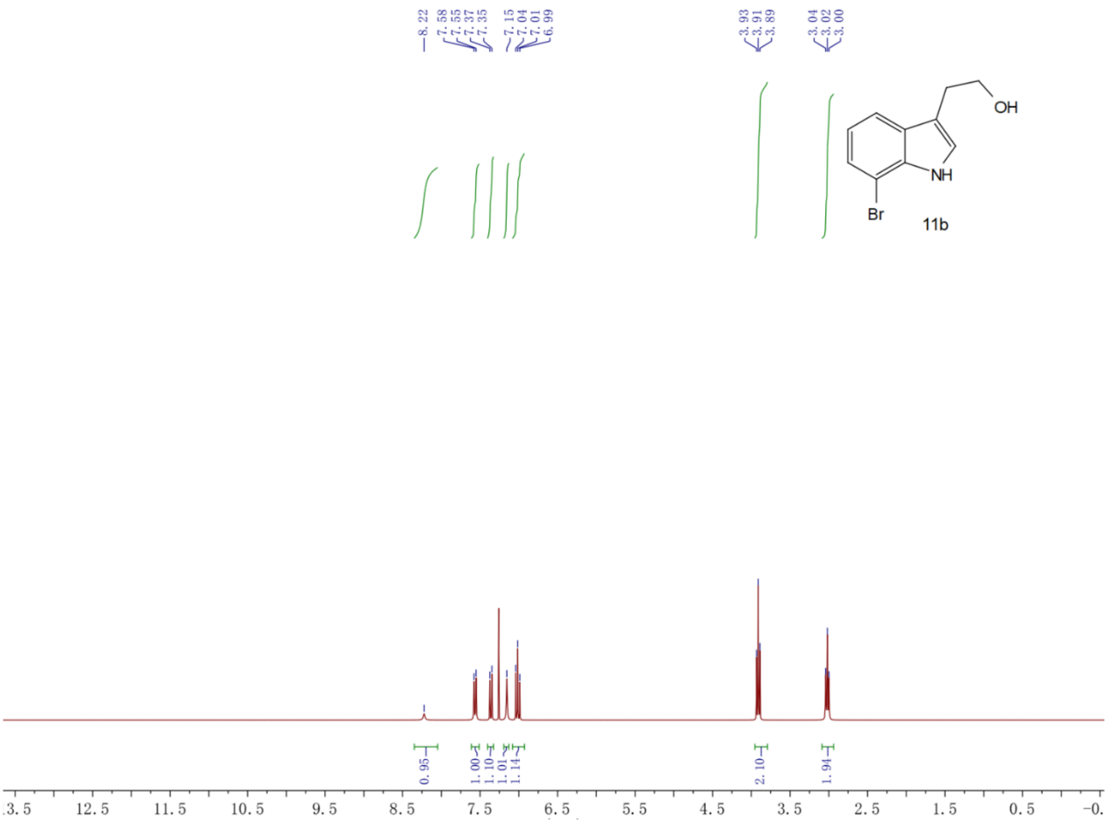


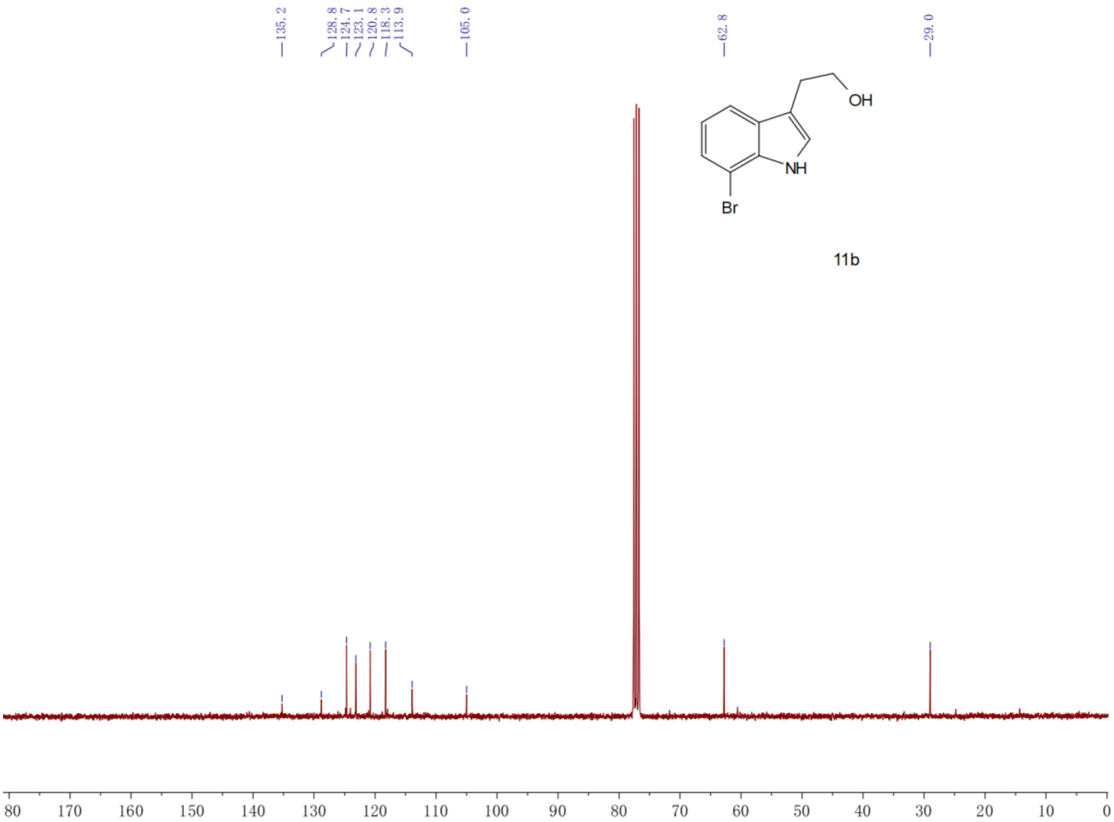


**Figure S15.** $^{1}H$ NMR and $^{13}C$ NMR of 7-bromo-tryptophol (**11b**) recorded in $CDCl_3$.

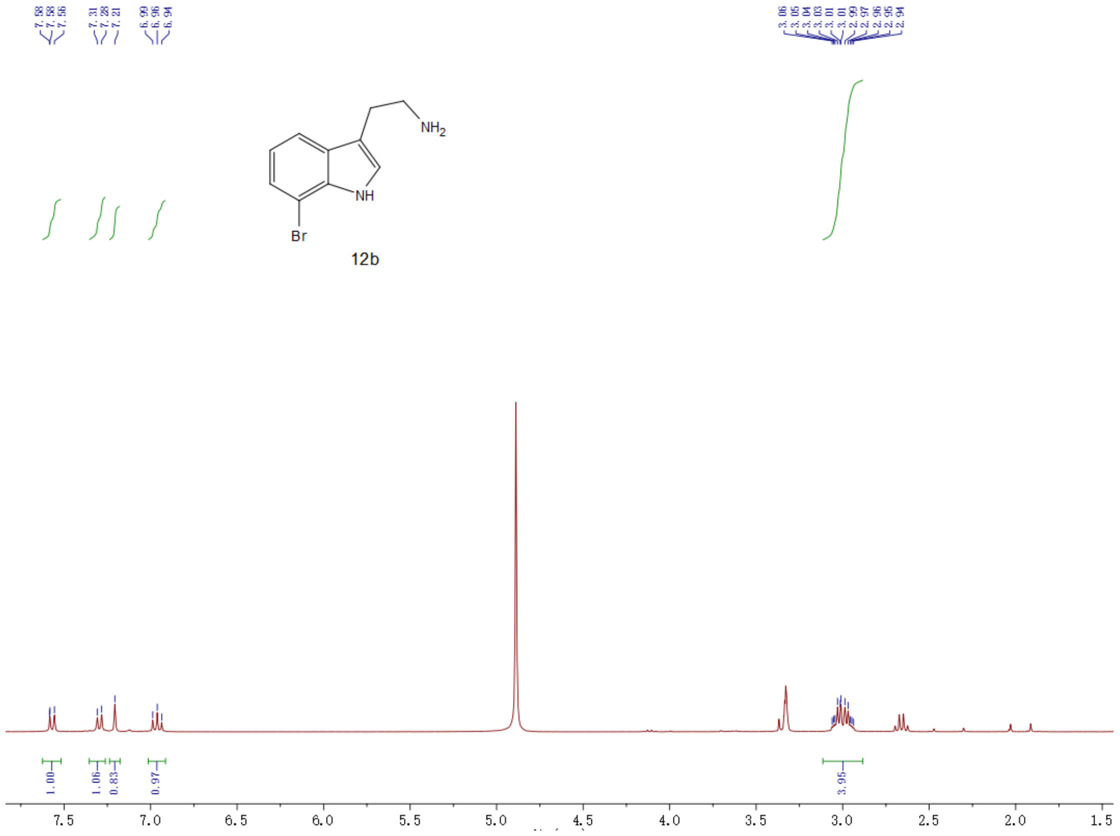

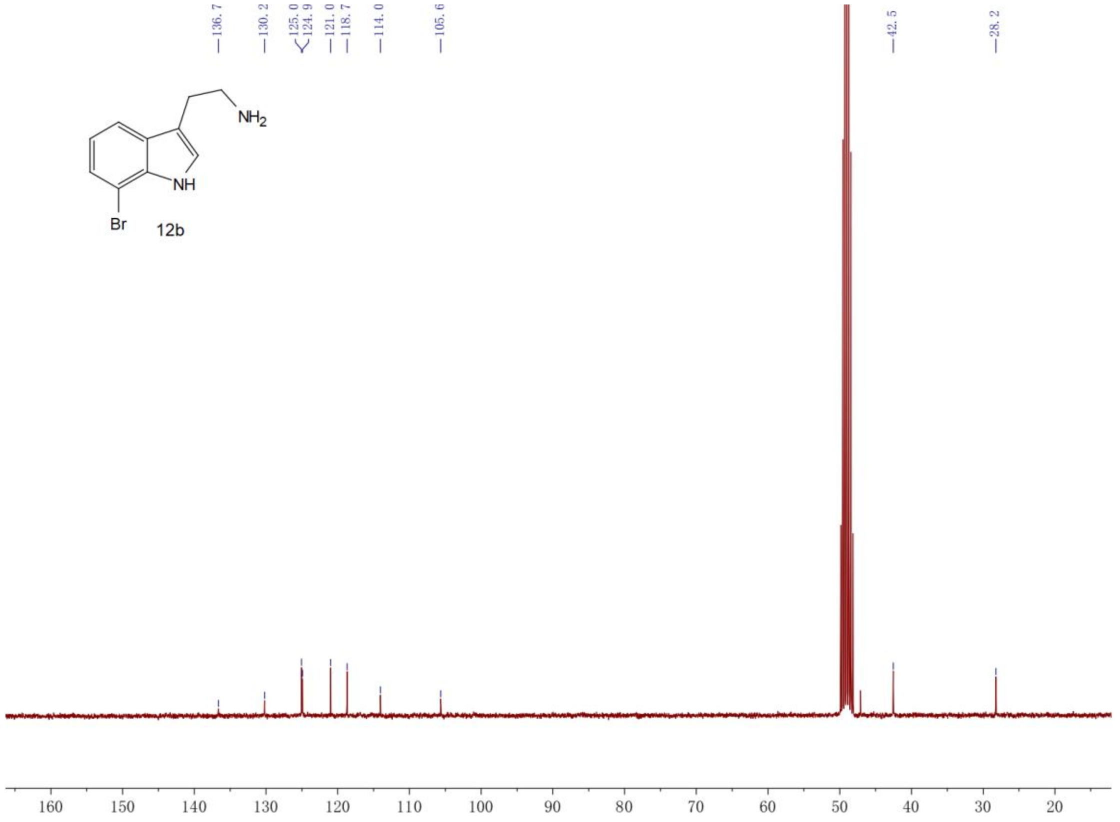

**Figure S16.** $^{1}H$ NMR and $^{13}C$ NMR of 7-bromotryptamine (**12b**) recorded in $CD_3OD$.

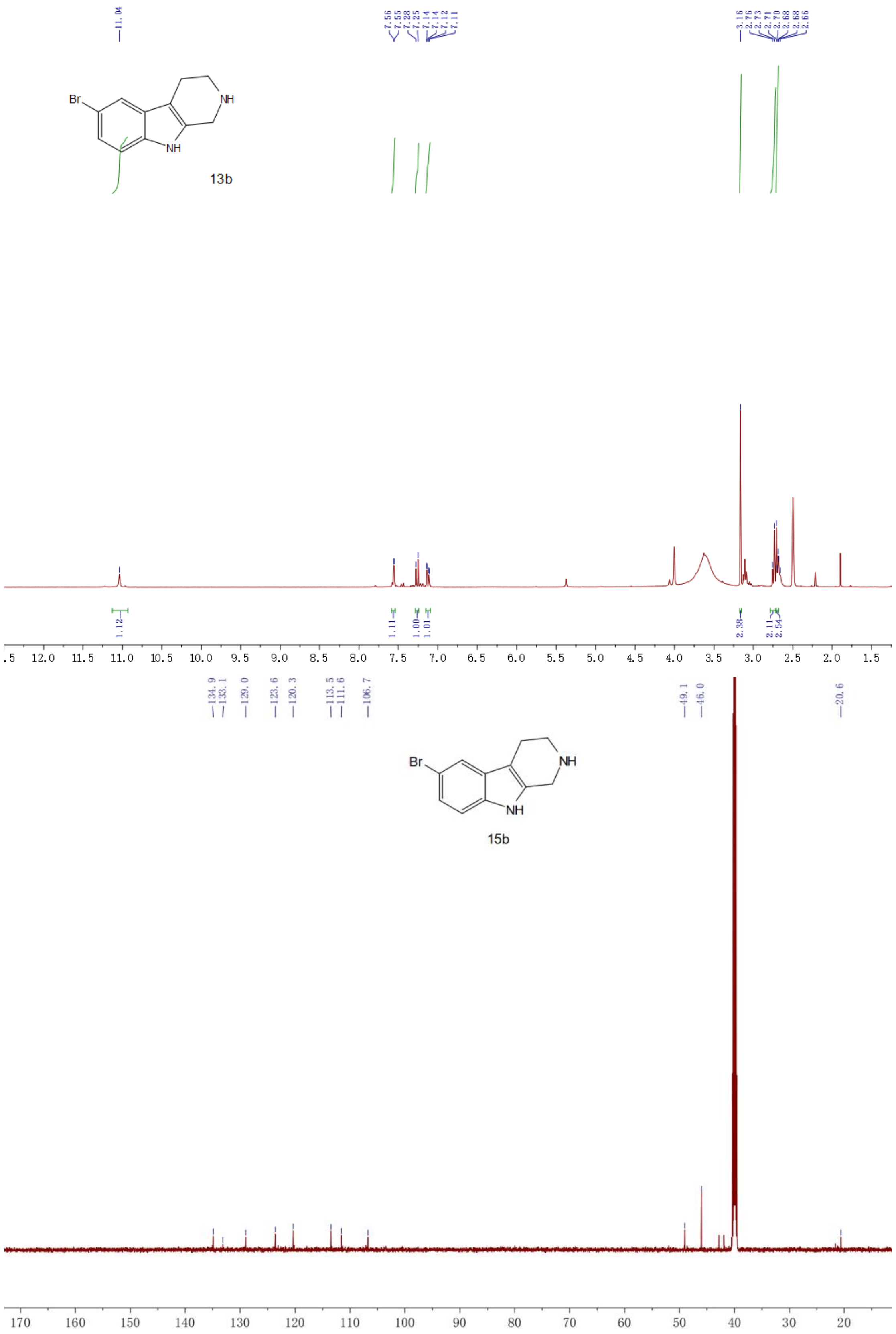


**Figure S17.** $^1H$ NMR and $^{13}C$ NMR of 6-bromo-1,2,3,4-tetrahydro-β-carboline (**13b**) recorded in DMSO-$d_6$.

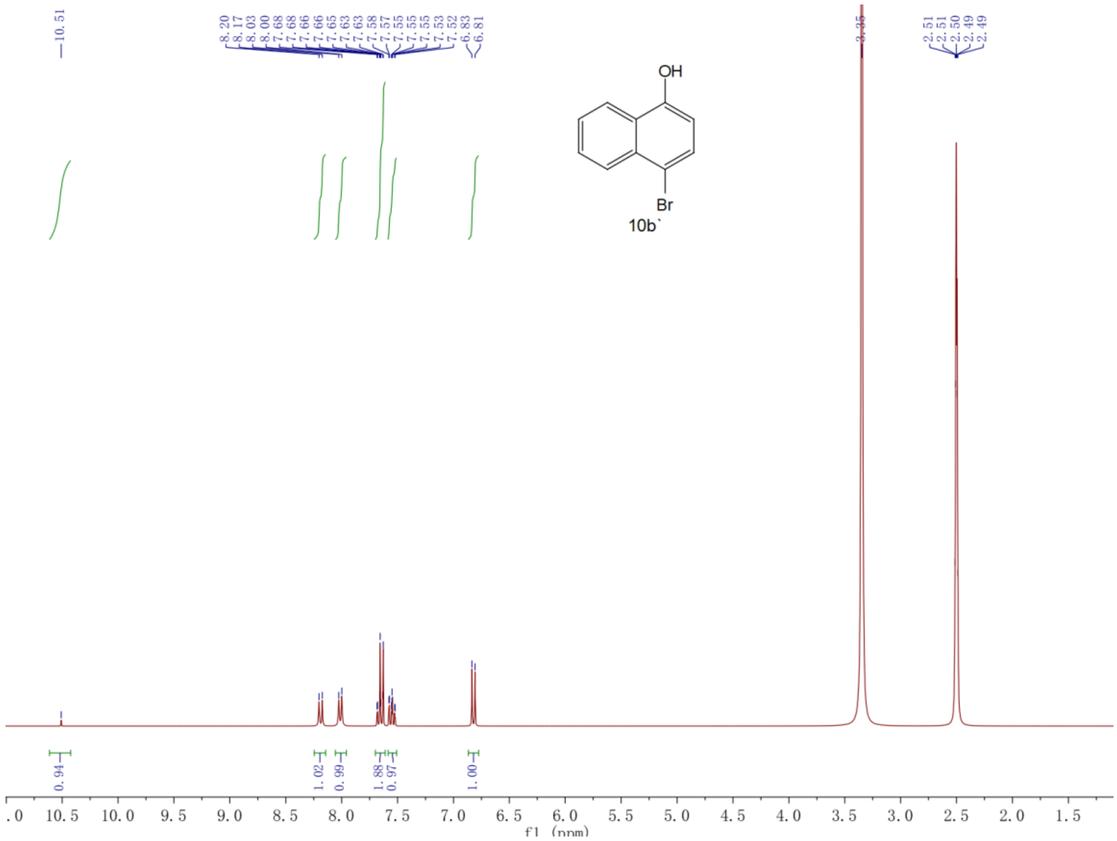


**Figure S18.** $^{1}H$ NMR of 4-bromo-1-naphthol (**10b'**) recorded in DMSO-$d_6$

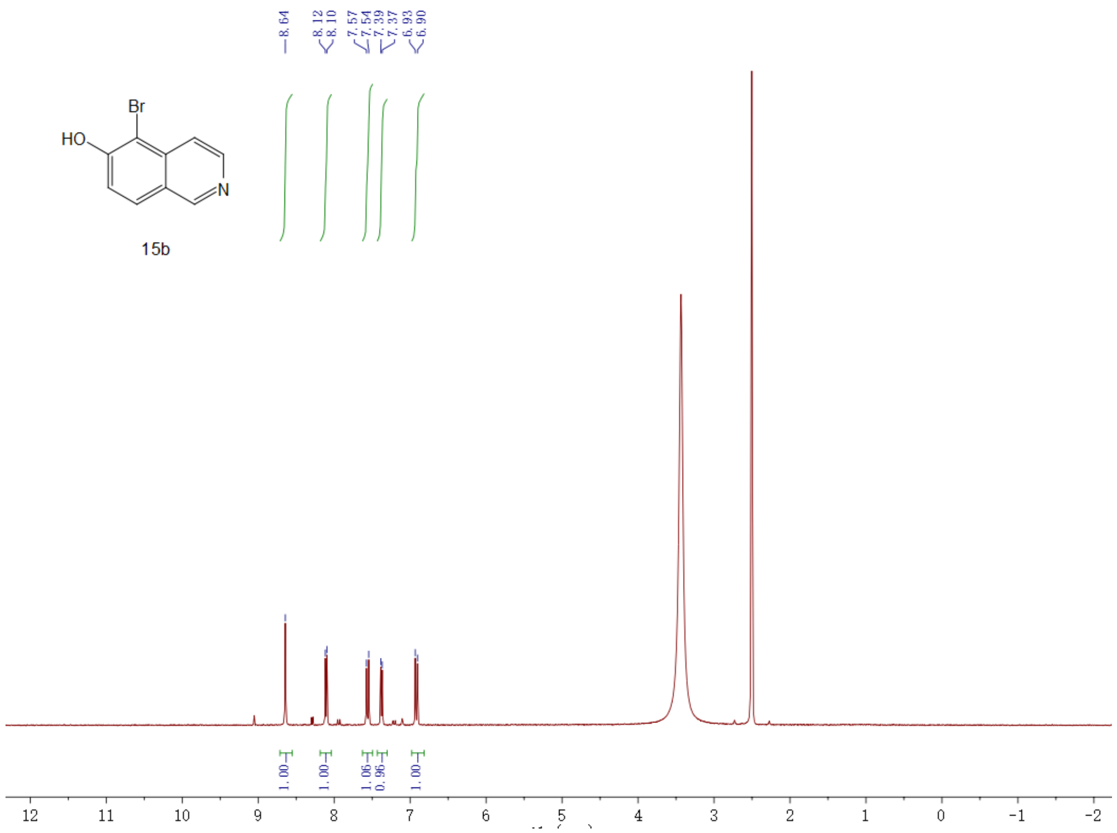


**Figure S19.** $^1$H NMR of 5-bromoisoquinolin-6-ol (**15b**) recorded in DMSO-$d_6$.

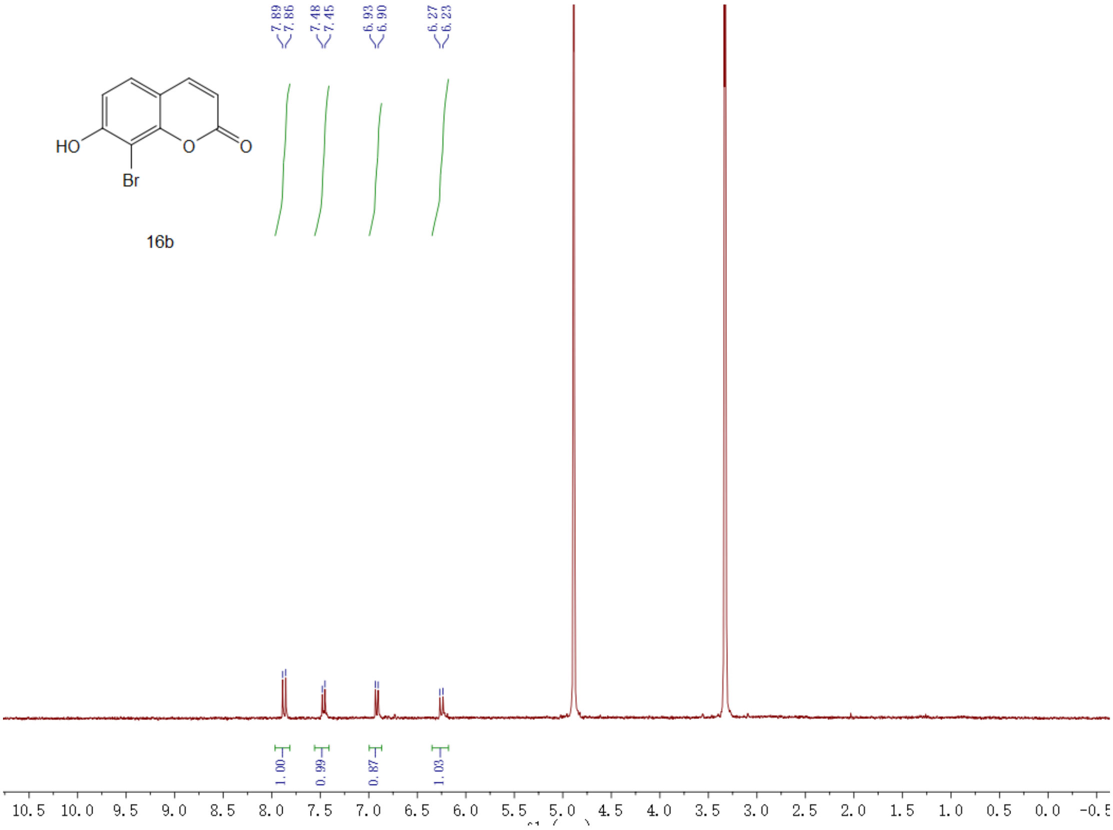


**Figure S20.** $^1$H NMR of 8-bromoumbelliferone (**16b**) recorded in $CD_3OD$.

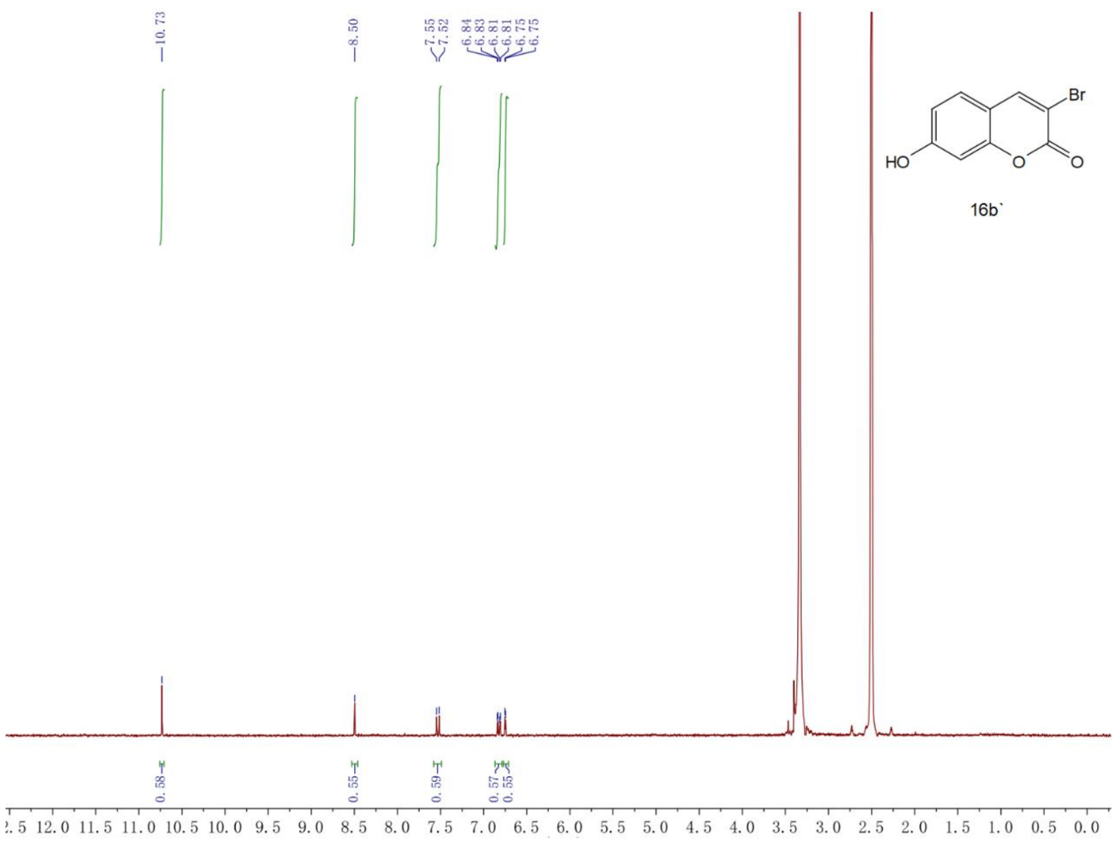


**Figure S21.** $^1$H NMR of 3-bromo-7-hydroxycoumarin (**16b'**) recorded in DMSO-$d_6$.

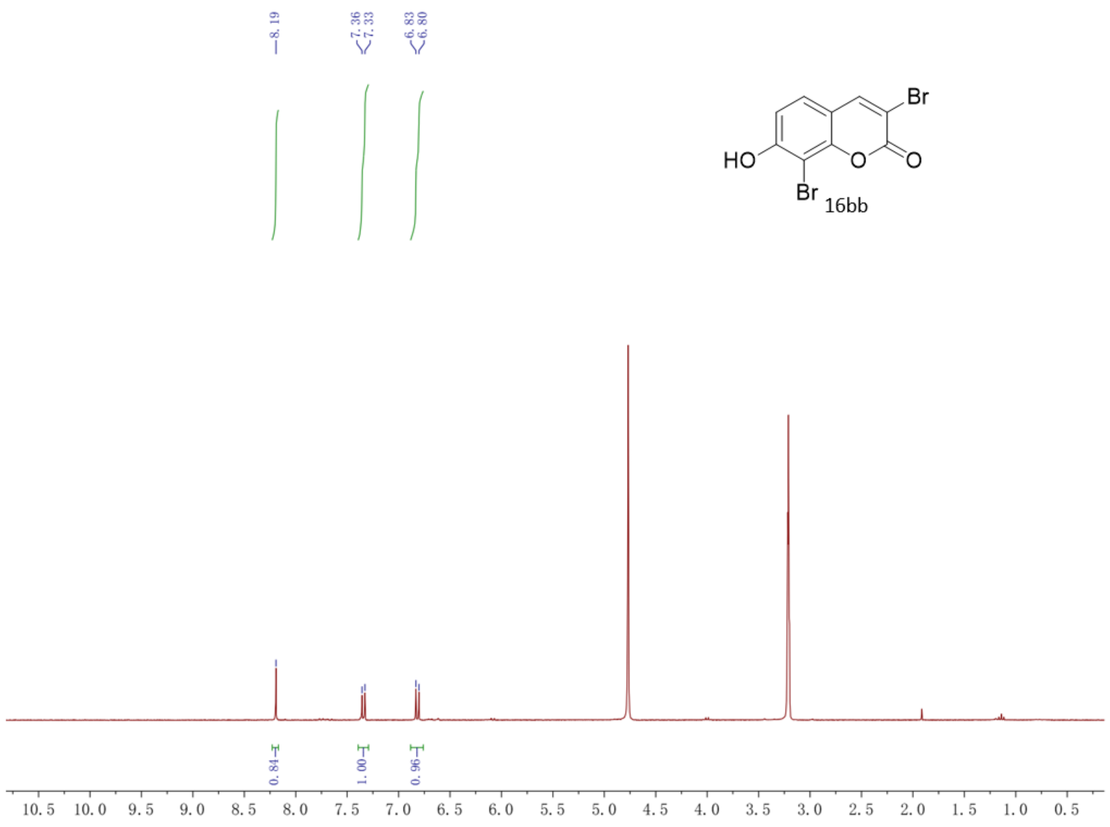


**Figure S22.** $^1$H NMR of 3,8-dibromo-7-methylcoumarin (**16bb**) recorded in $CD_3OD$.

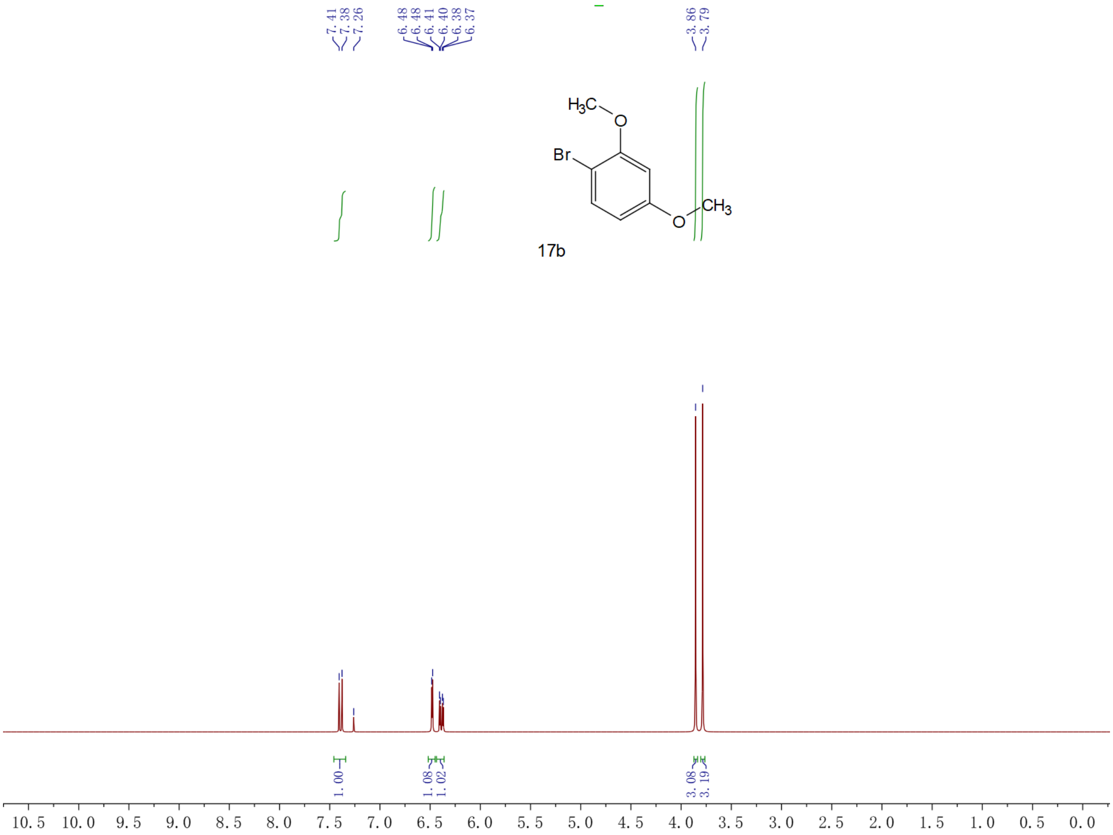


**Figure S23.** $^{1}$H NMR of 1-bromo-2,4-dimethoxybenzene (**17b**) recorded in $CDCl_3$.

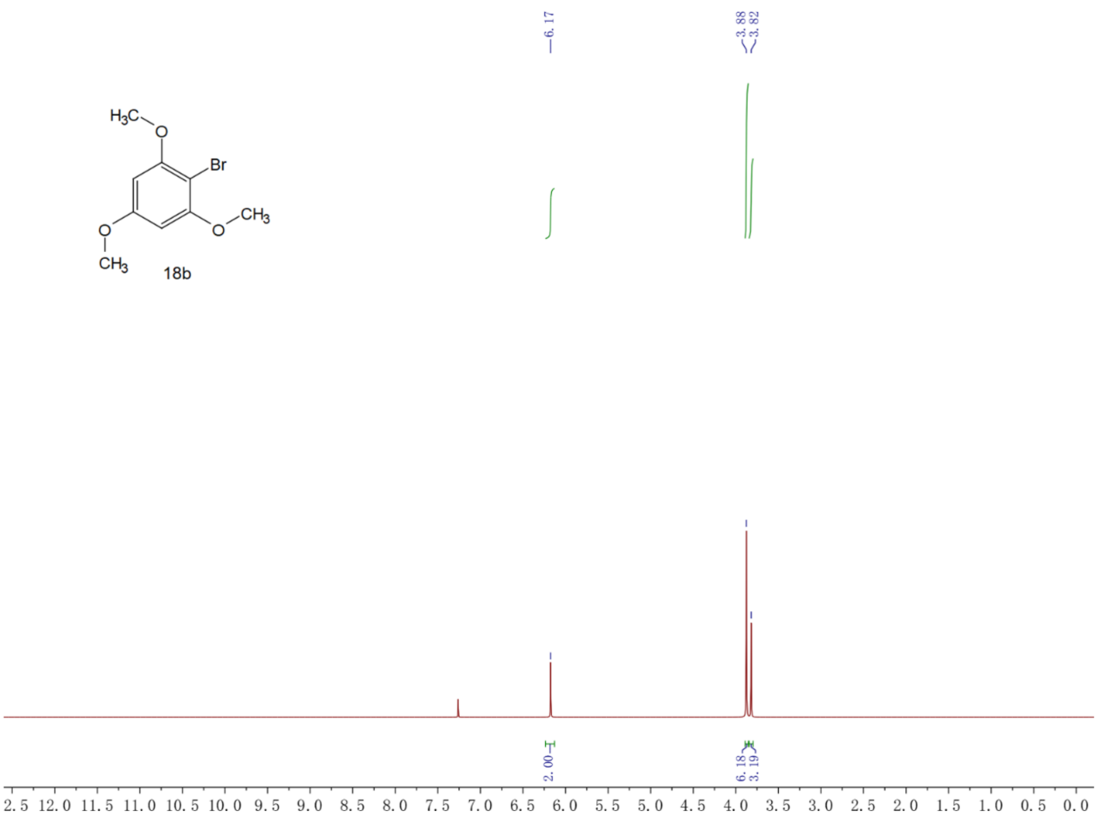


**Figure S24.** $^{1}H$ NMR of 1-bromo-2,4,6-trimethoxybenzene (**18b**) recorded in $CDCl_3$.

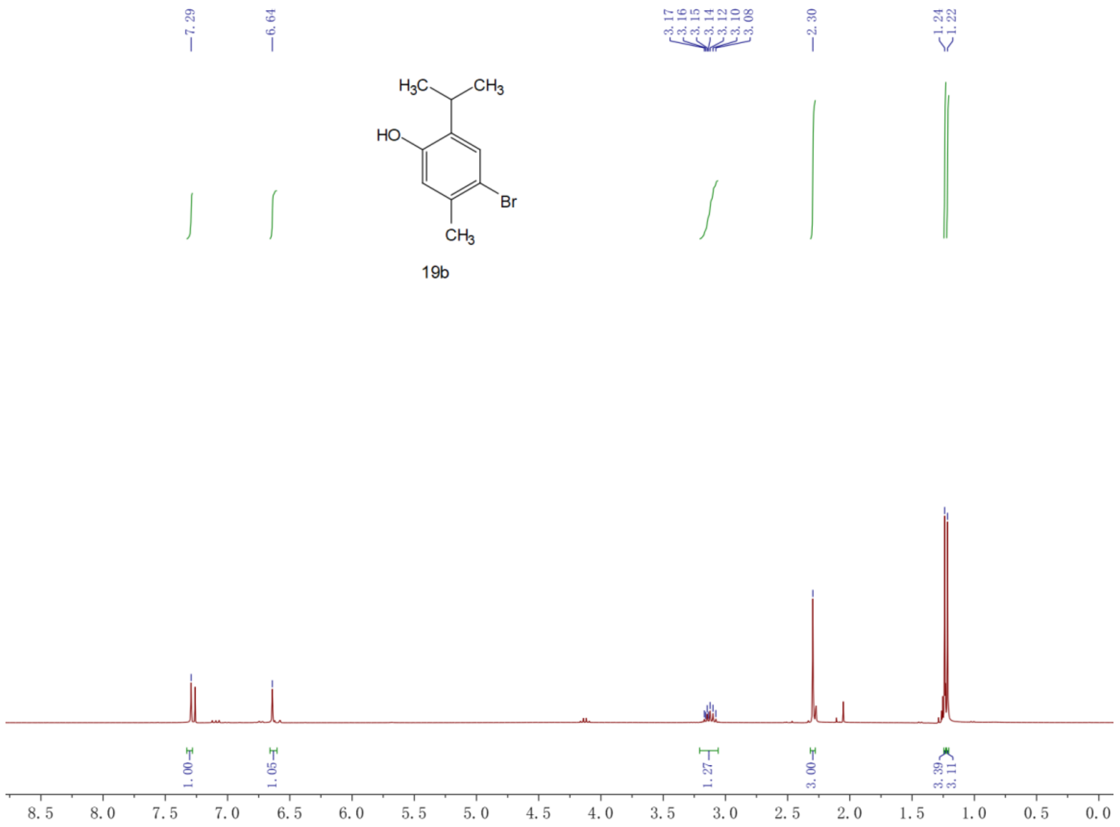


**Figure S25.** $^{1}H$ NMR of 4-bromothymol (**19b**) recorded in $CDCl_3$.

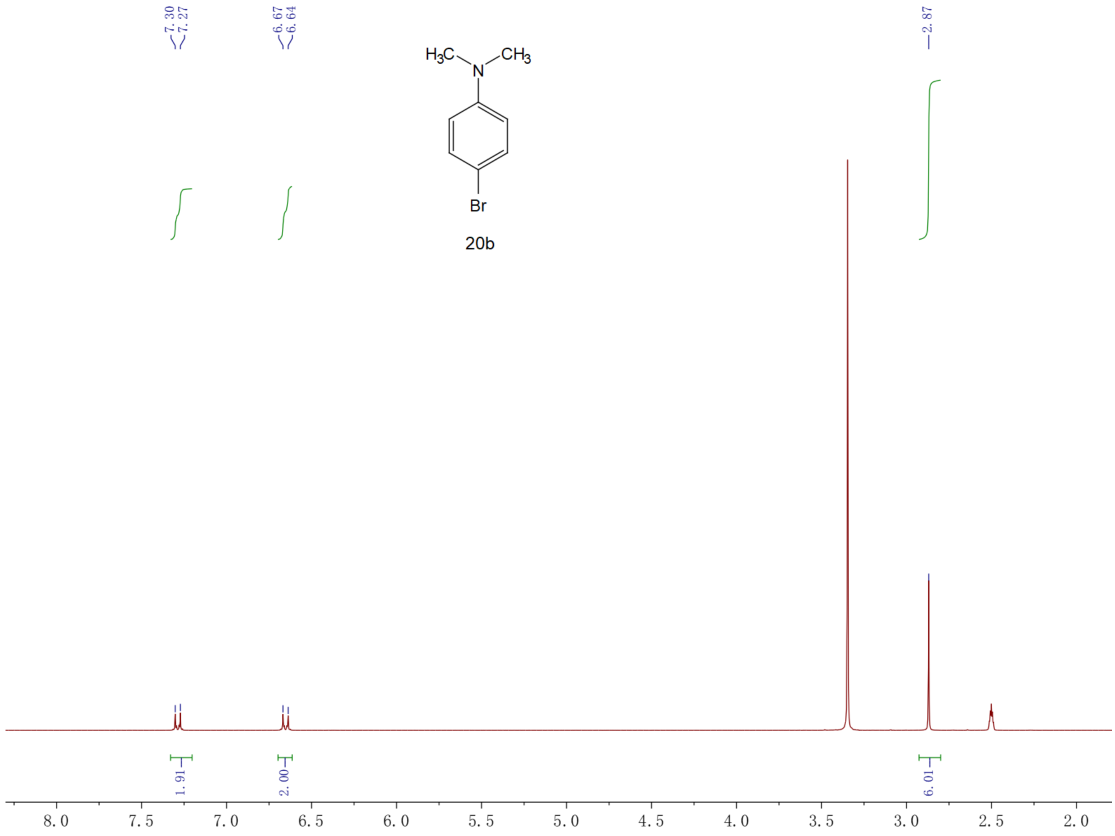


**Figure S26.** $^1H$ NMR of 4-Bromo-N,N-dimethylaniline (**20b**) recorded in DMSO-$d_6$.

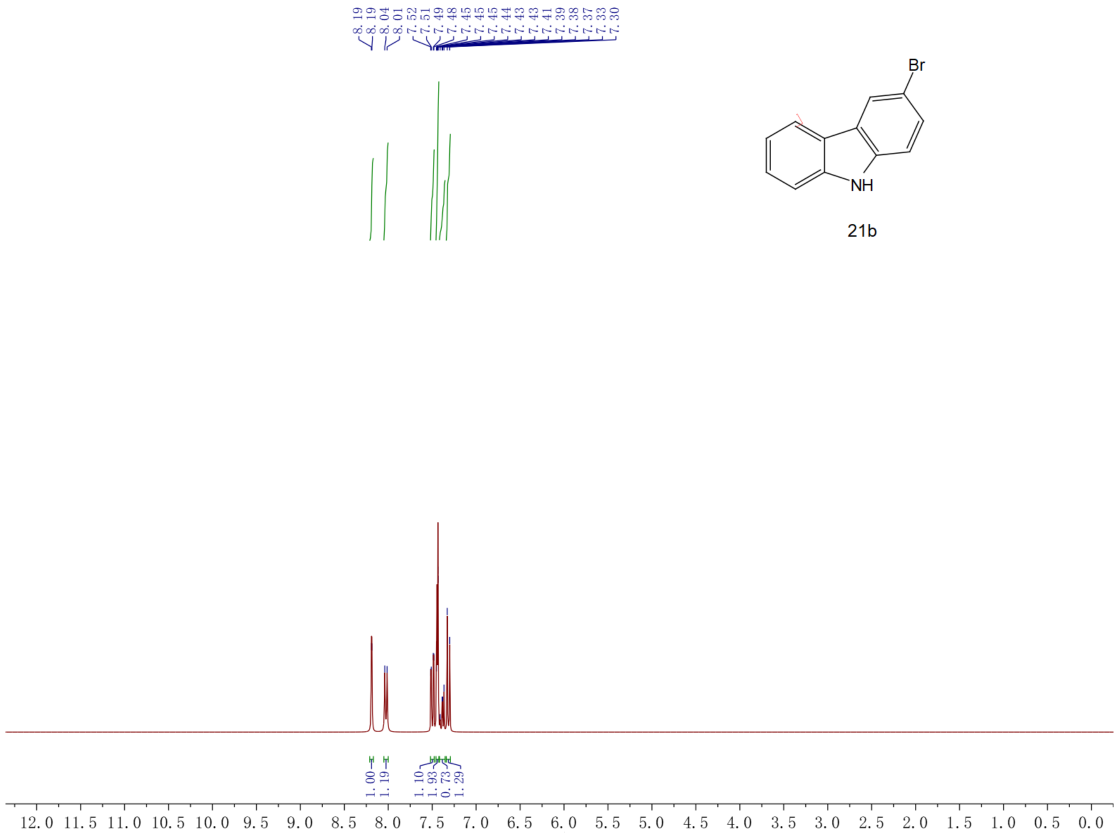


**Figure S27.** $^1$H NMR of 3-bromo-9H-carbazole (**21b**) recorded in $CDCl_3$.

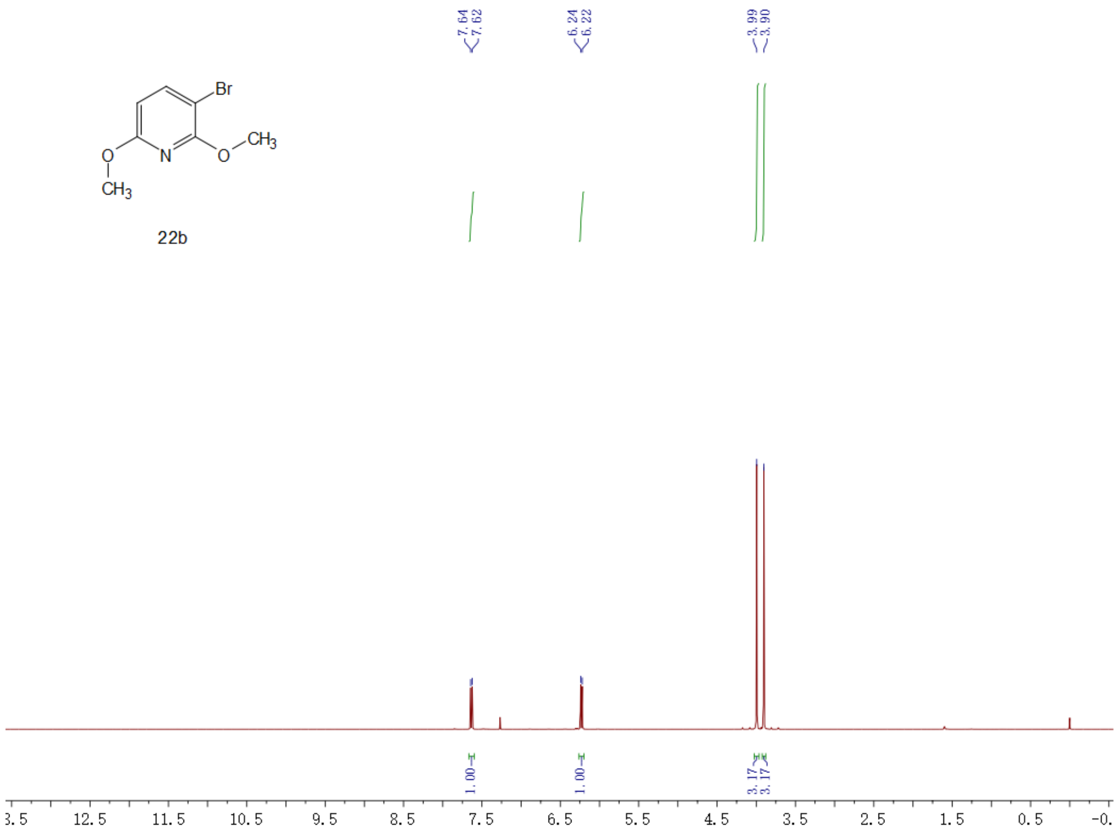


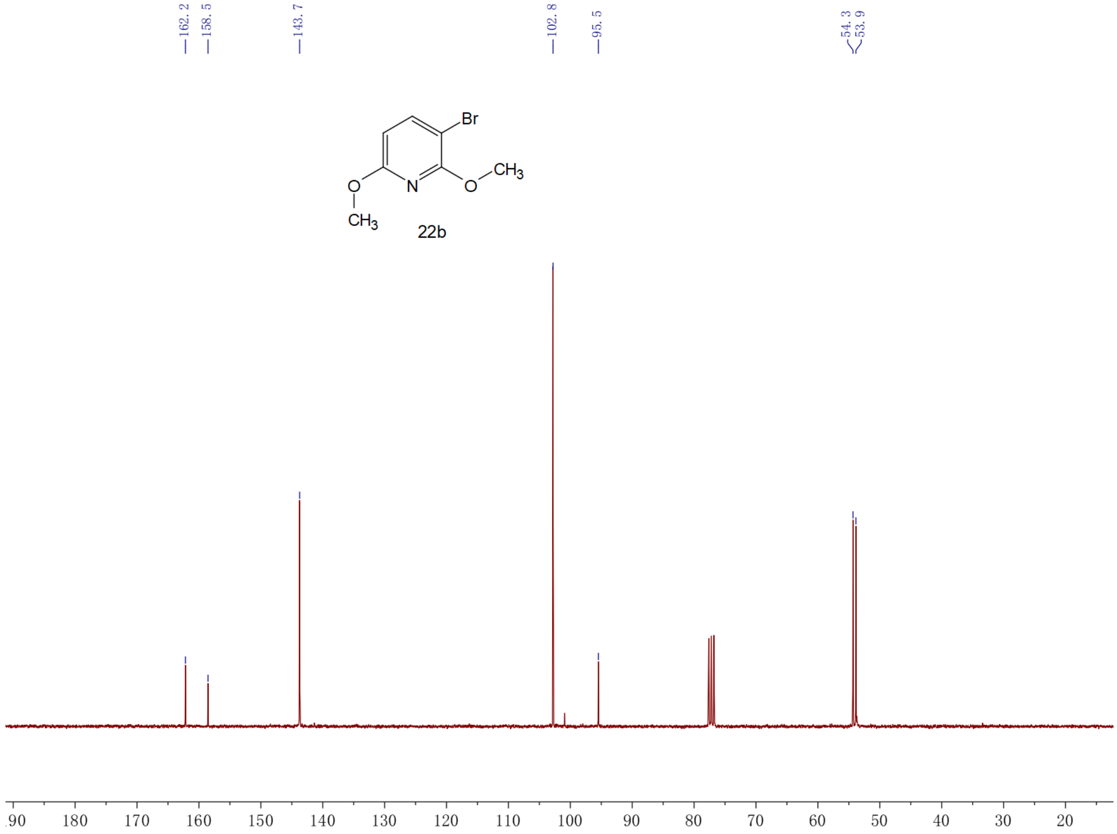


**Figure S28.** $^{1}H$ NMR and $^{13}C$ NMR of 3-bromo-2,6-dimethoxypyridine (**22b**) recorded in $CDCl_3$.

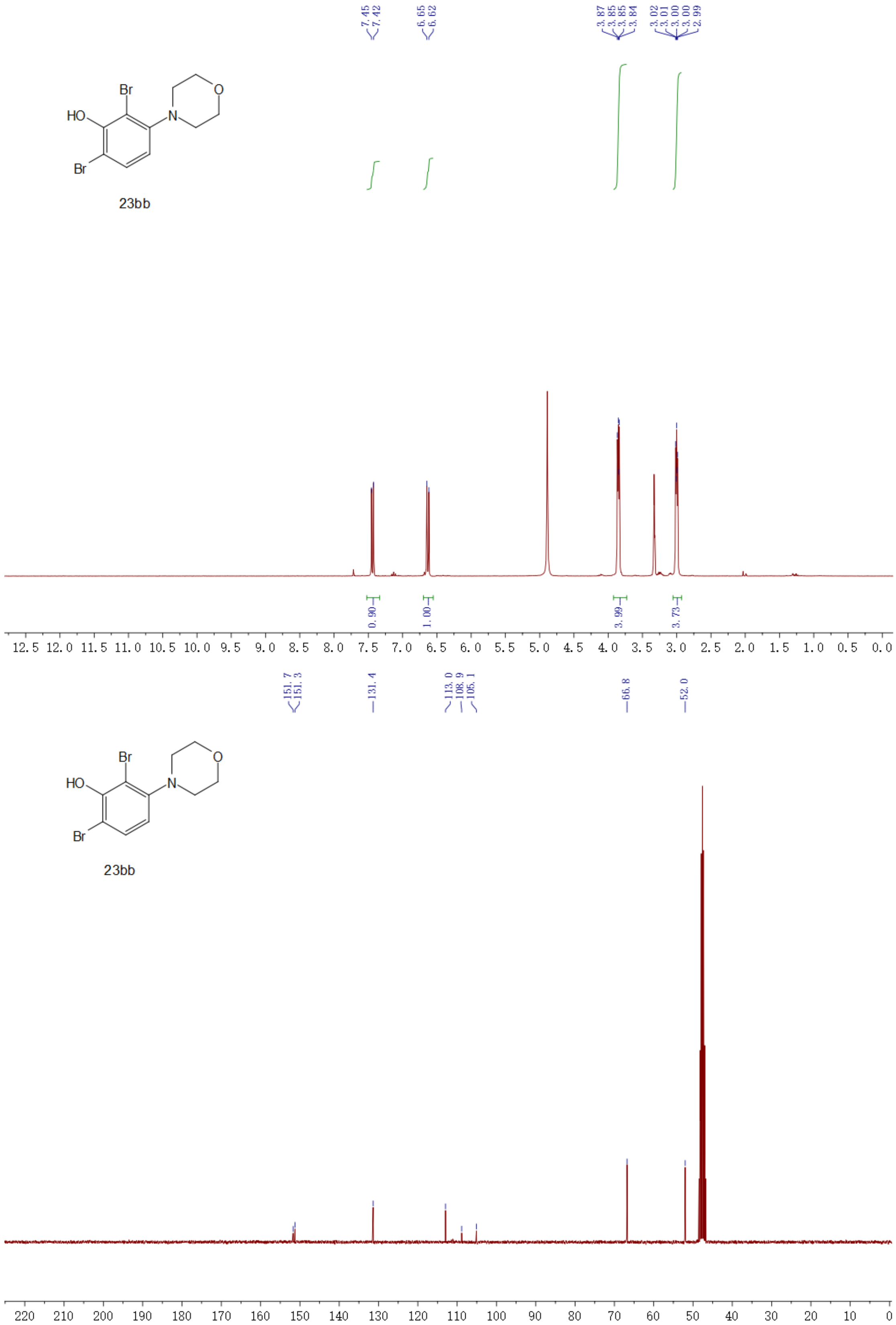


**Figure S29.** $^{1}H$ NMR and $^{13}C$ NMR of 2,4-dibromo-3-(4-morpholino)phenol (**23bb**) recorded in $CD_3OD$.

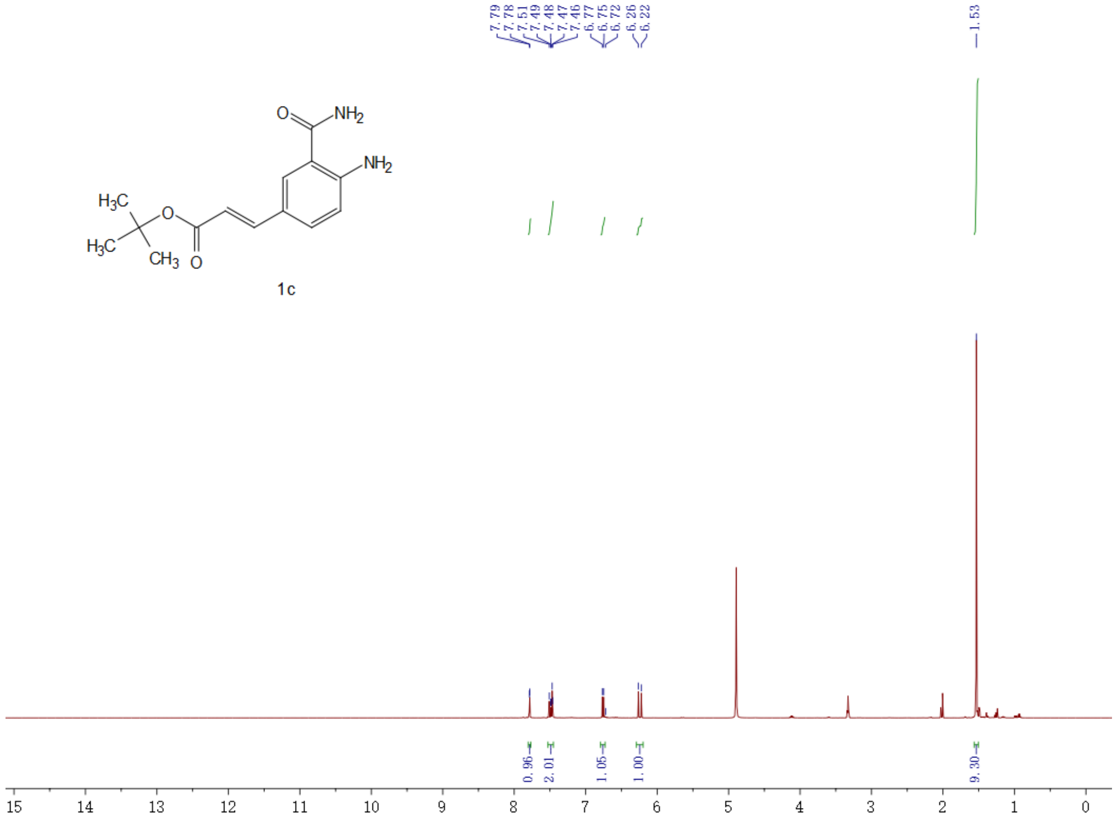


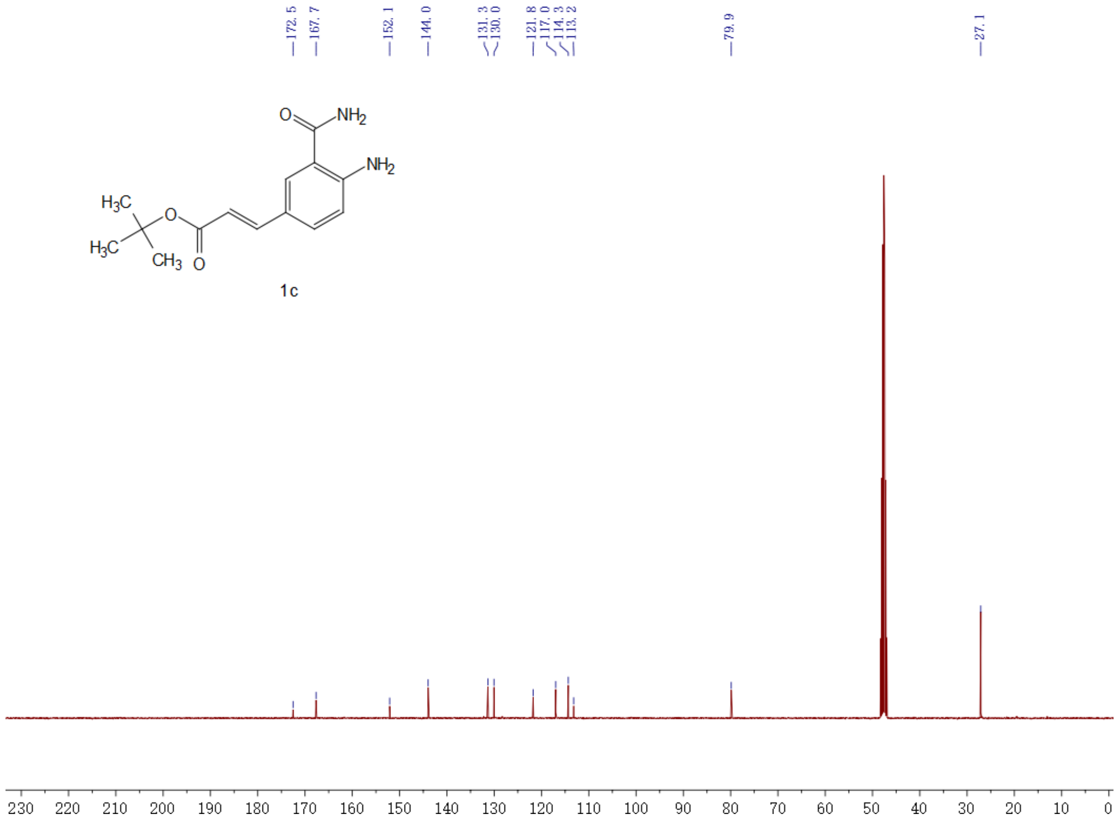


**Figure S30.** $^{1}H$ NMR and $^{13}C$ NMR of *tert*-butyl (*E*)-3-(4-amino-3-carbamoylphenyl)acrylate (**1c**) recorded in $CD_3OD$.

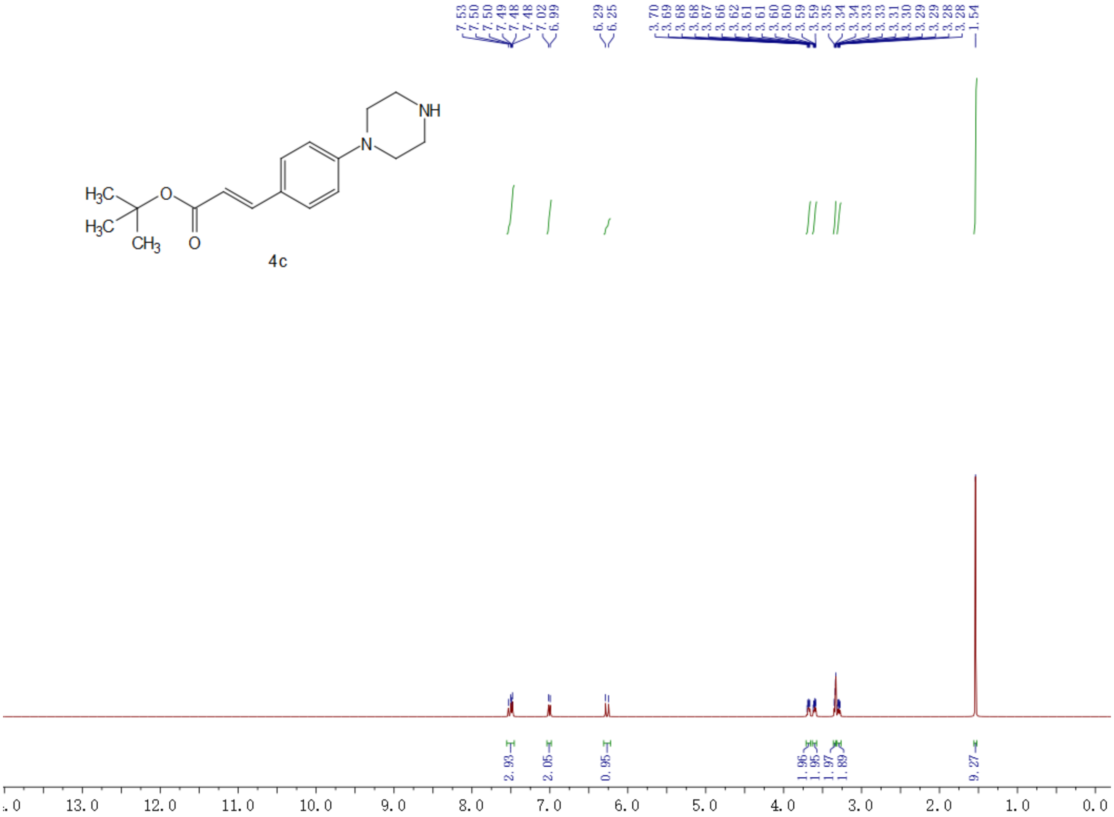


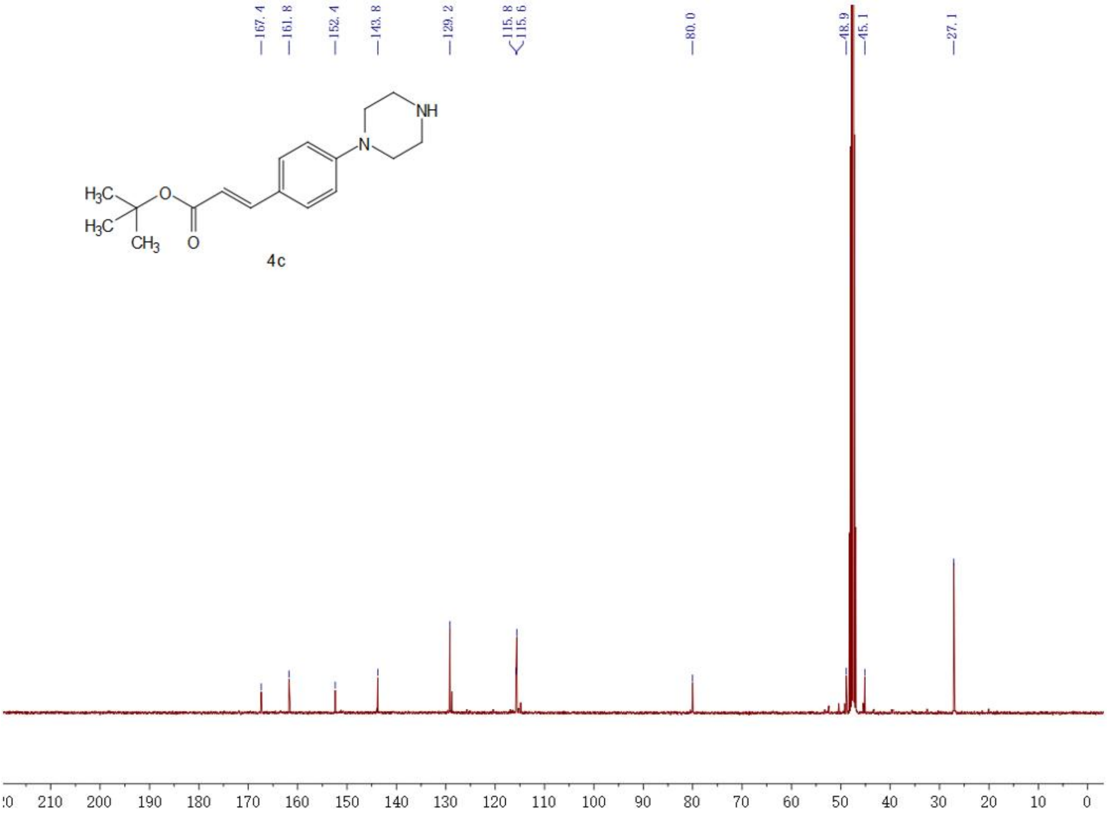


**Figure S31.** $^1$H NMR and $^{13}$C NMR of *tert*-butyl (*E*)-3-(4-(piperazin-1-yl)phenyl)acrylate (**4c**) recorded in $CD_3OD$.

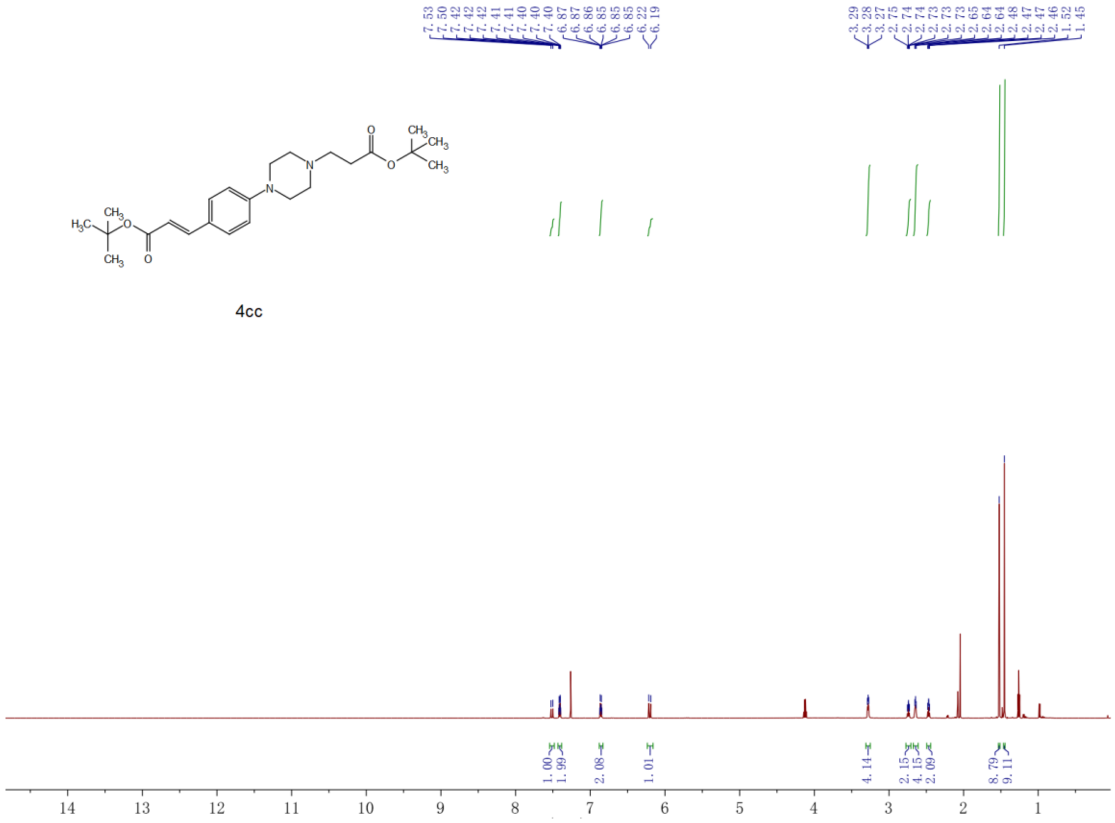


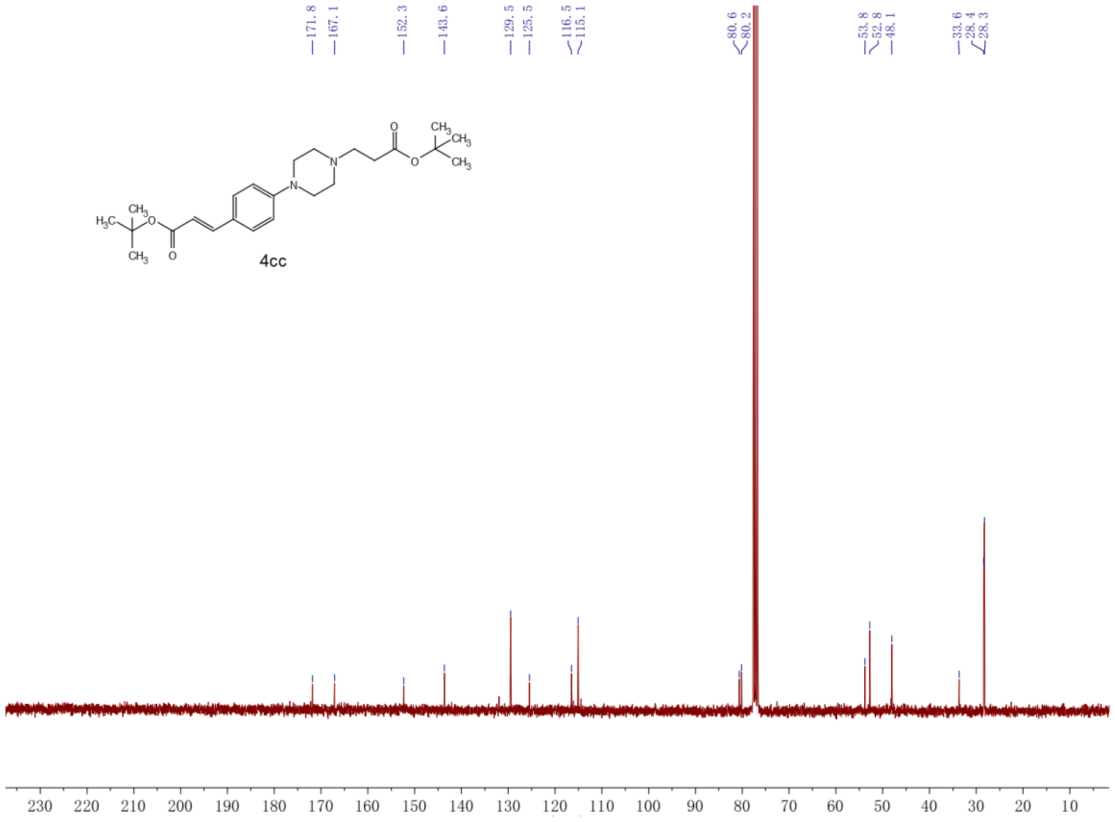


**Figure S32.** $^1H$ NMR and $^{13}C$ NMR of *tert*-butyl (*E*)-3-(4-(4-(3-(tert-butoxy)-3-oxopropyl)piperazin-1-yl)phenyl)acrylate (**4cc**) recorded in $CDCl_3$.

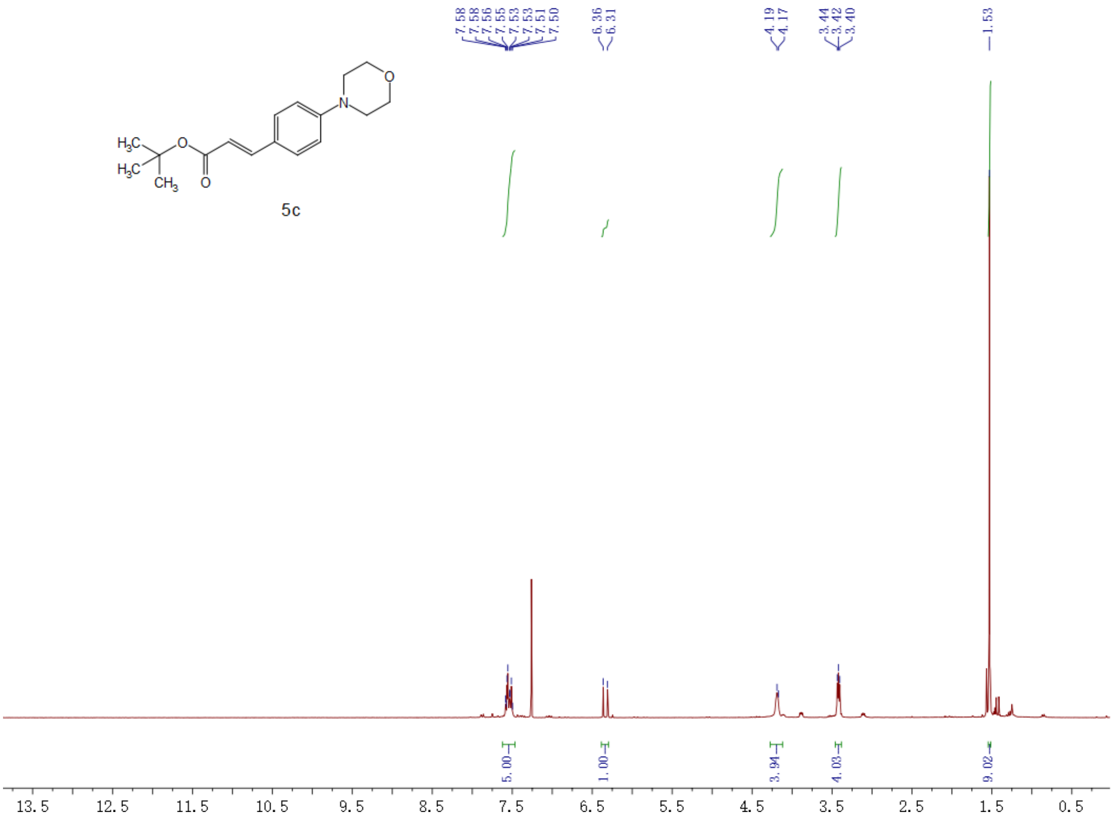

**Figure S33.** $^{1}$H NMR of *tert*-butyl (*E*)-3-(4-morpholinophenyl)acrylate (**5c**) recorded in $CDCl_3$.

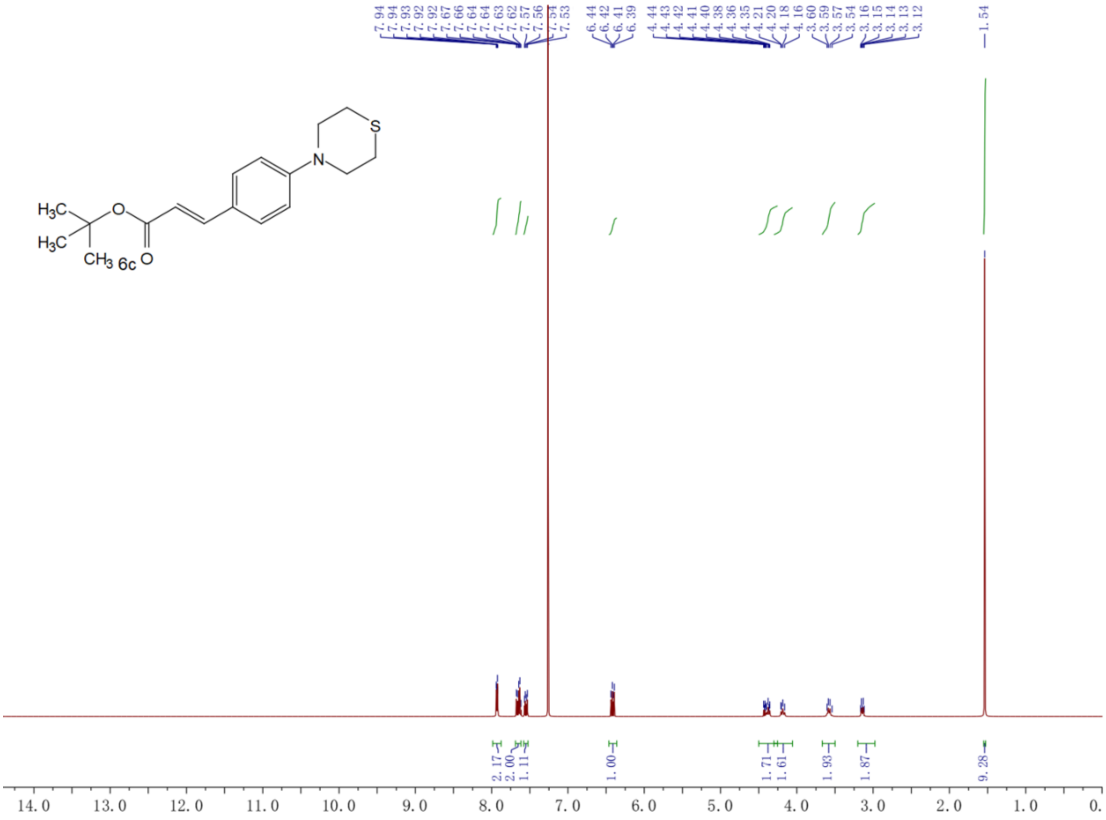

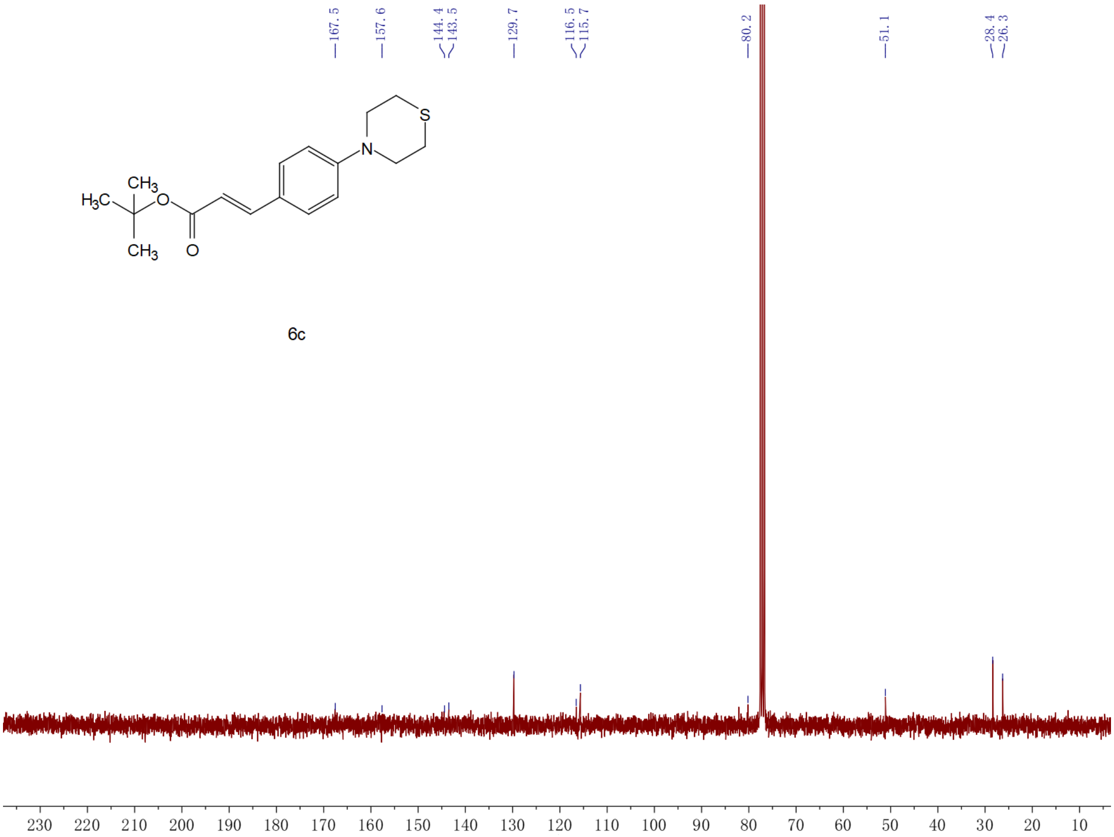

**Figure S34.** $^{1}H$ NMR and $^{13}C$ NMR of *tert*-butyl (*E*)-3-(4-thiomorpholinophenyl)acrylate (**6c**) recorded in $CDCl_3$.

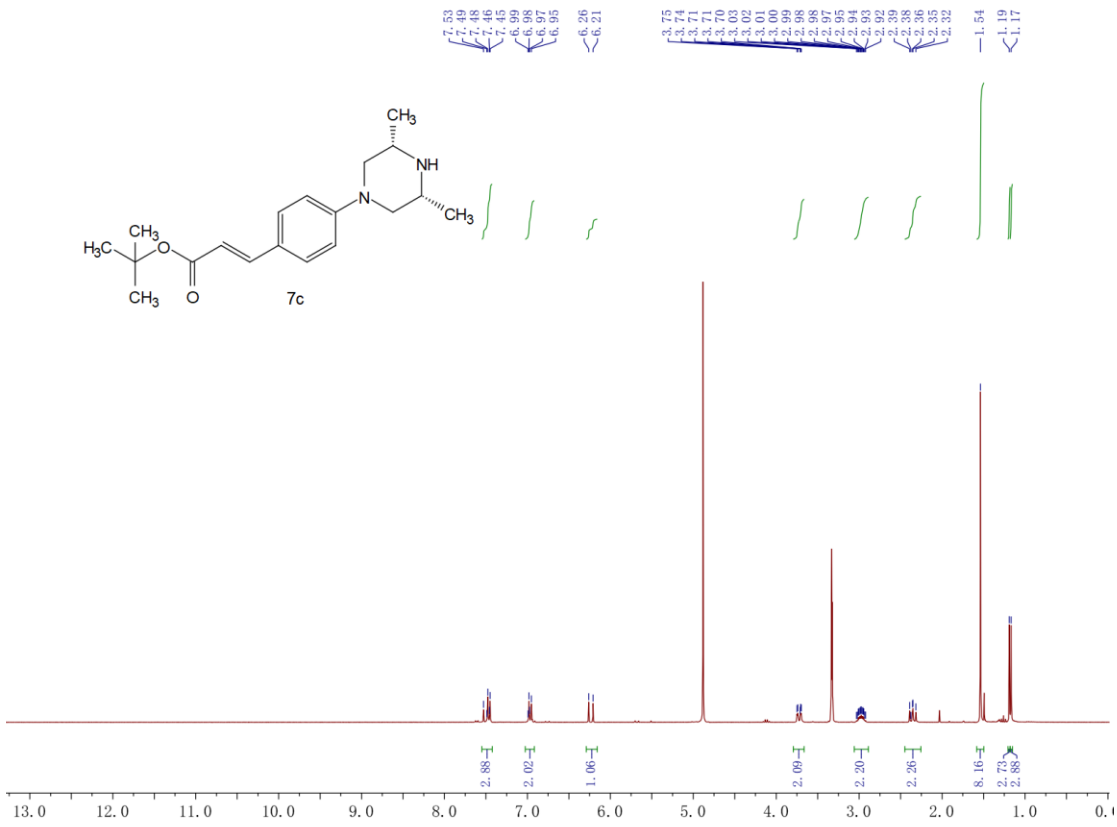

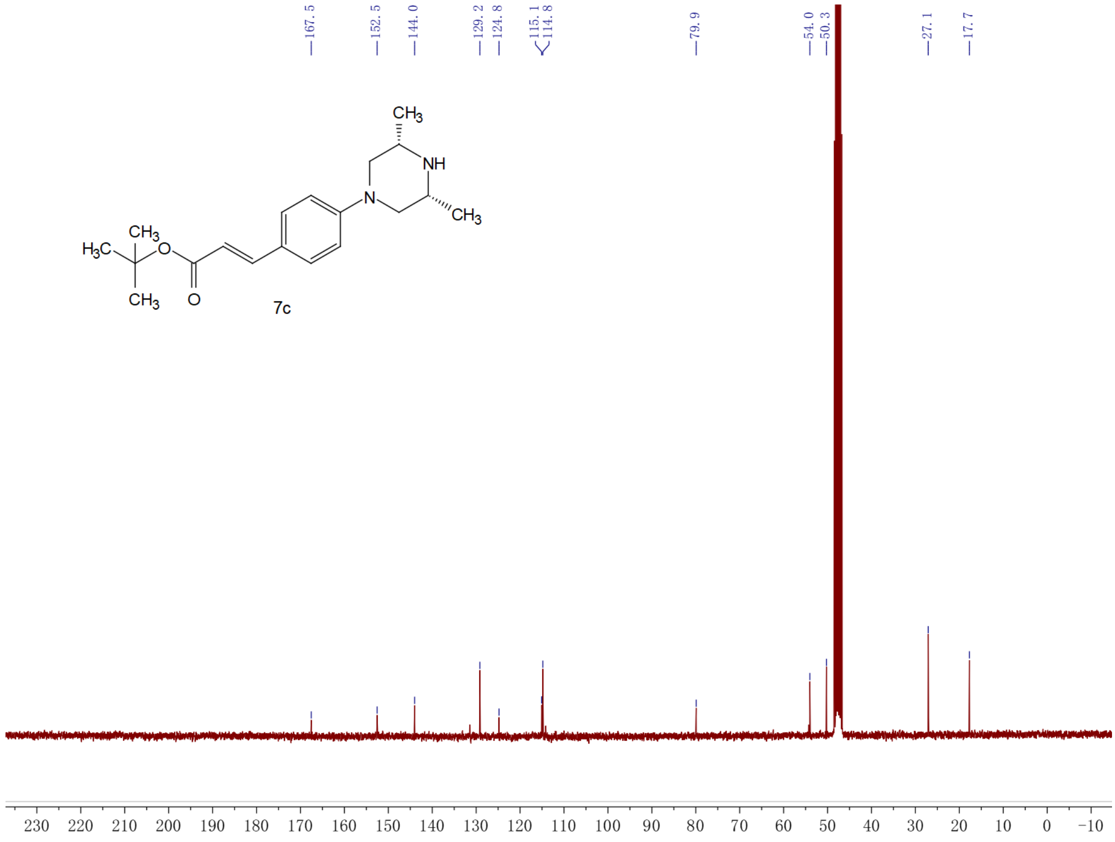

**Figure S35.** $^{1}H$ NMR and $^{13}C$ NMR of *tert*-butyl (*E*)-3-(4-((3R,5S)-3,5-dimethylpiperazin-1-yl)phenyl)acrylate (**7c**) recorded in $CD_3OD$.

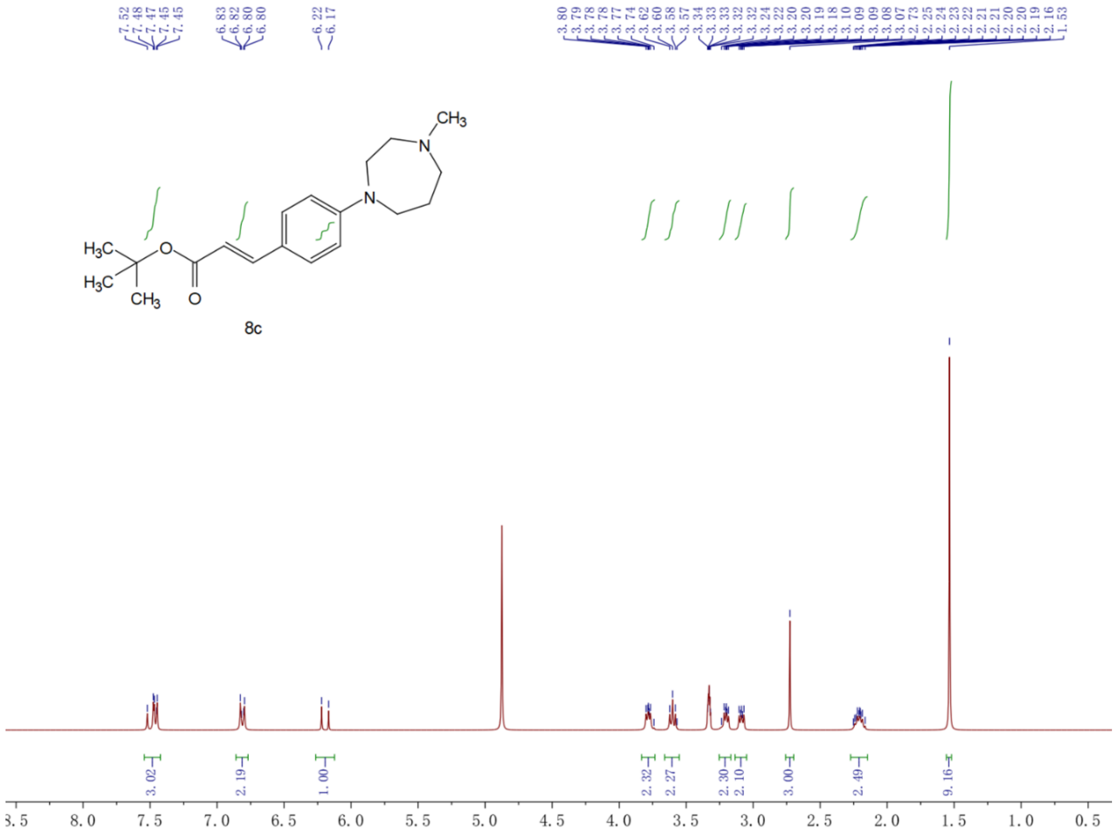

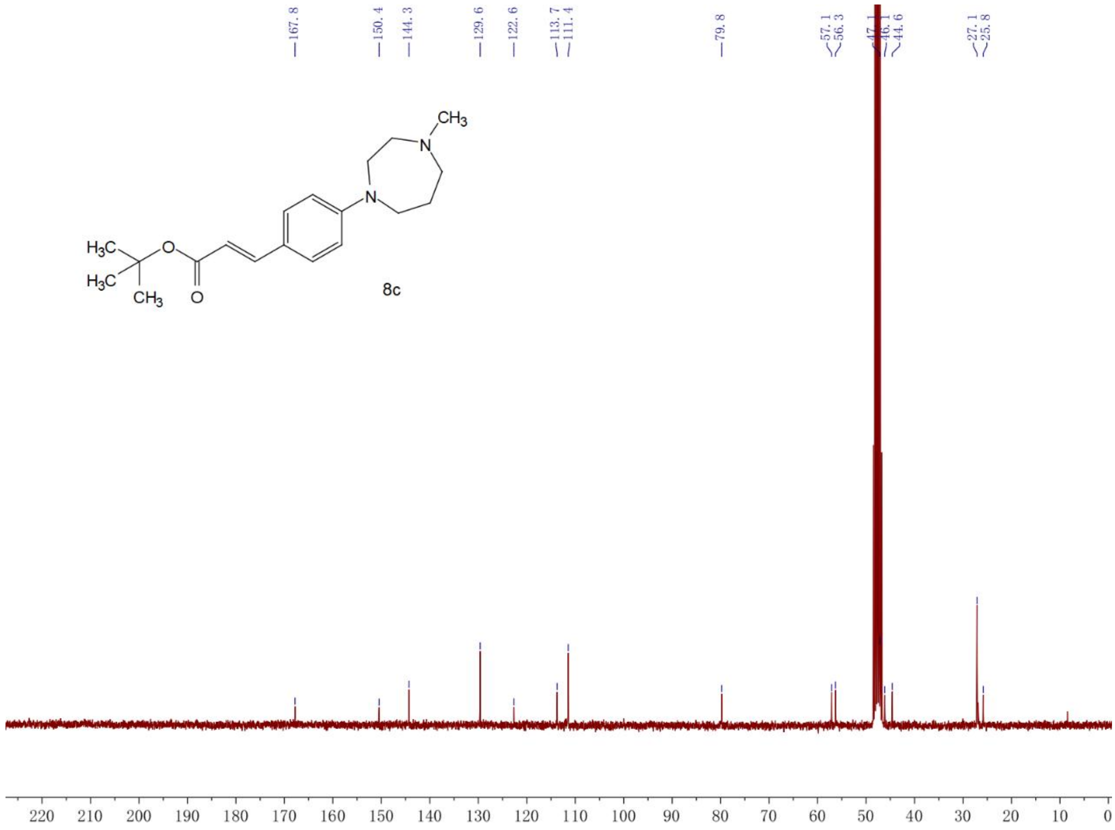

**Figure S36.** $^{1}H$ NMR and $^{13}C$ NMR of *tert*- butyl (*E*)-3-(4-(4-methyl-1,4-diazepan-1-yl)phenyl)acrylate (**8c**) recorded in $CD_3OD$.

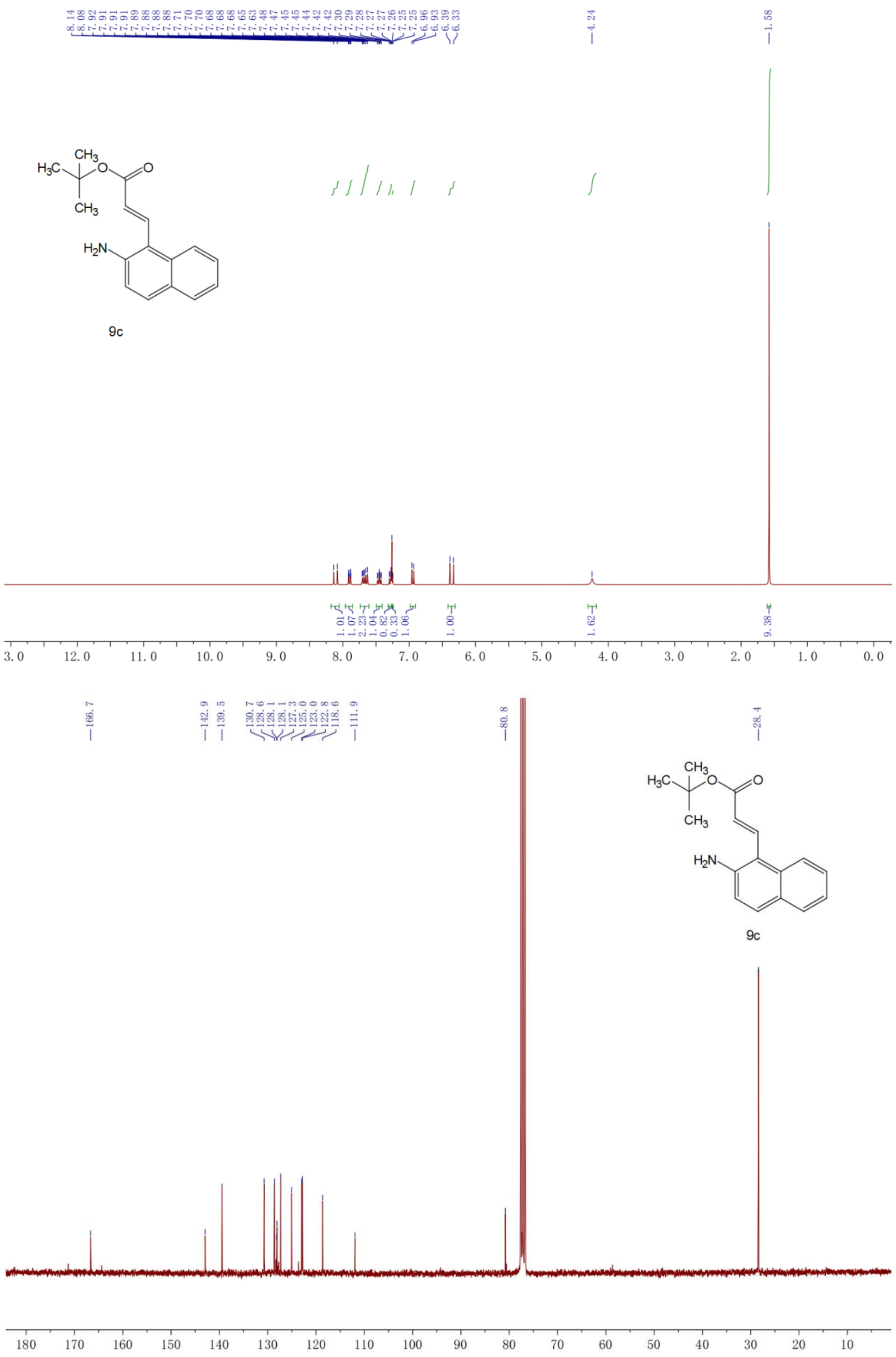


**Figure S37.** $^1$H NMR and $^{13}$C NMR of *tert*-butyl (*E*)-3-(2-aminonaphthalen-1-yl)acrylate (**9c**) recorded in $CDCl_3$.

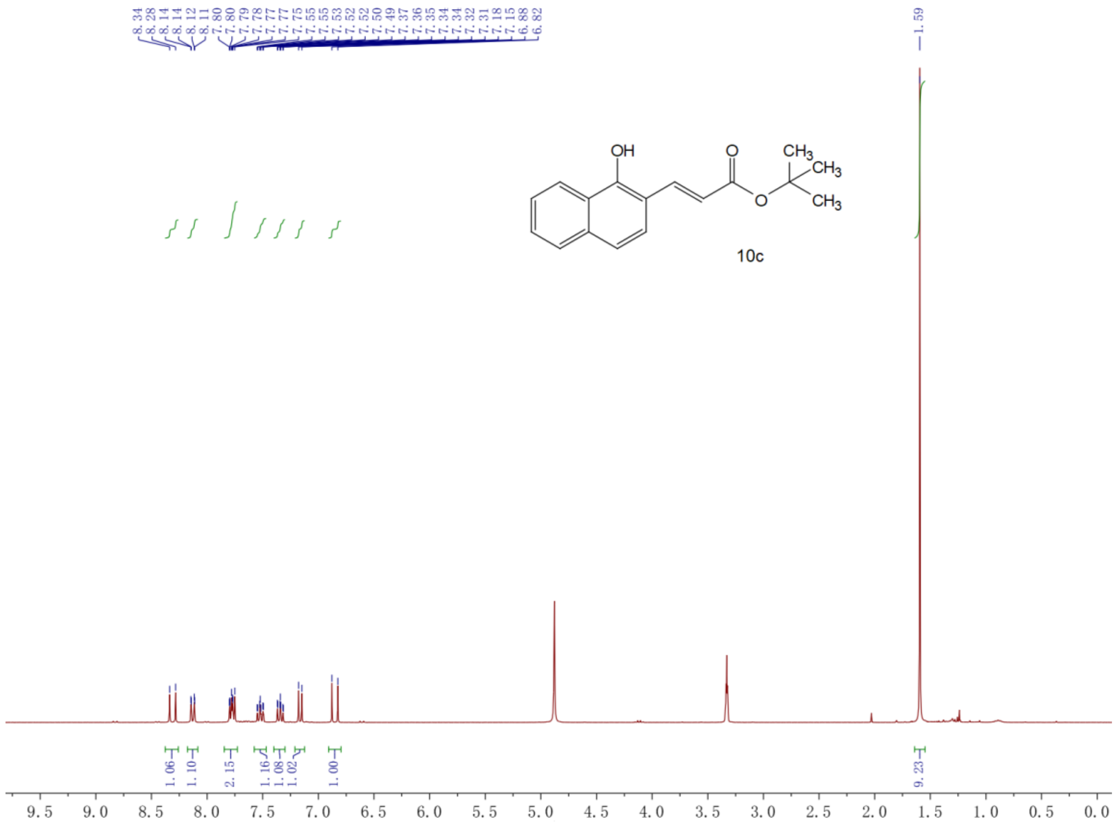

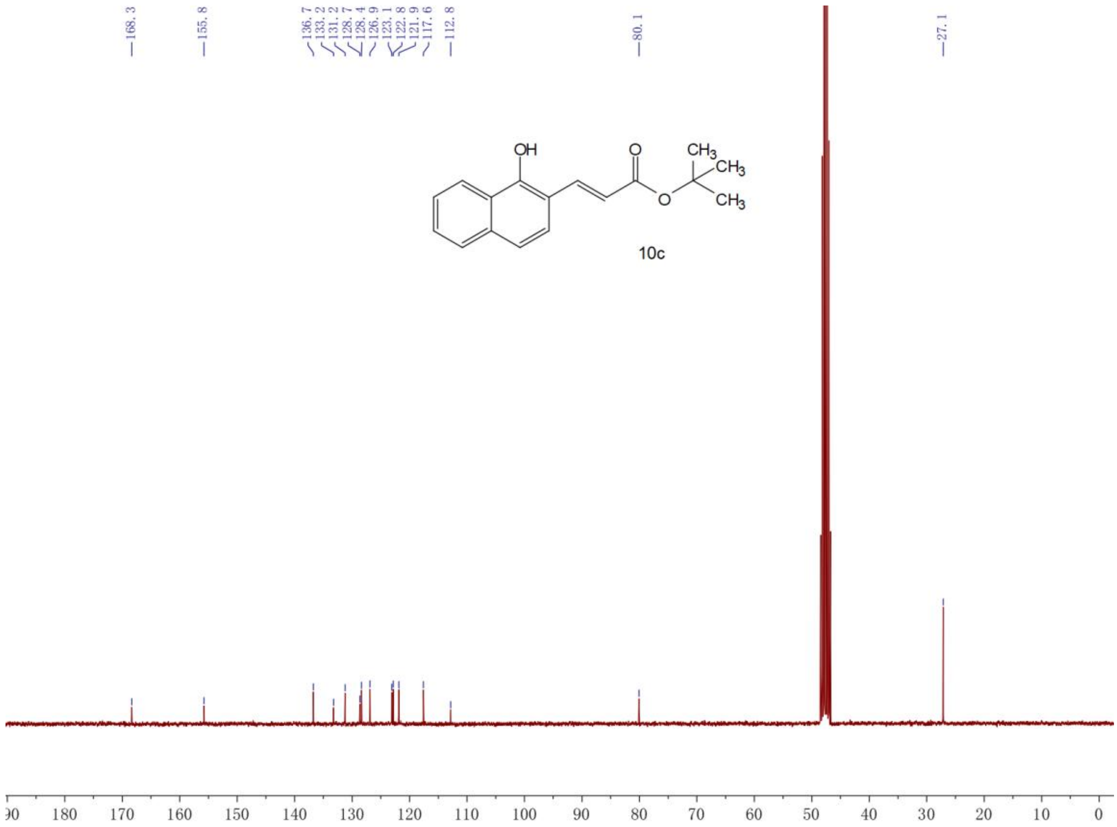

**Figure S38.** $^{1}H$ NMR and $^{13}C$ NMR of *tert*- butyl (*E*)-3-(1-hydroxynaphthalen-2-yl)acrylate (**10c**) recorded in $CD_3OD$.

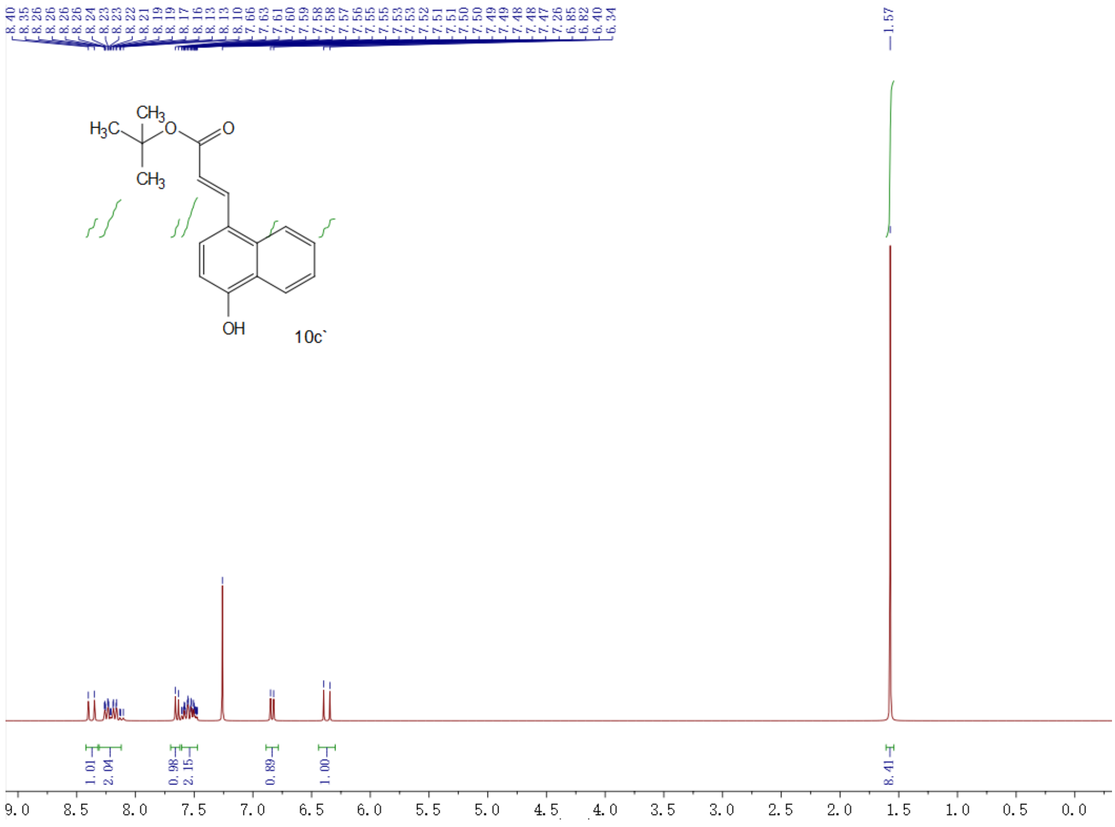

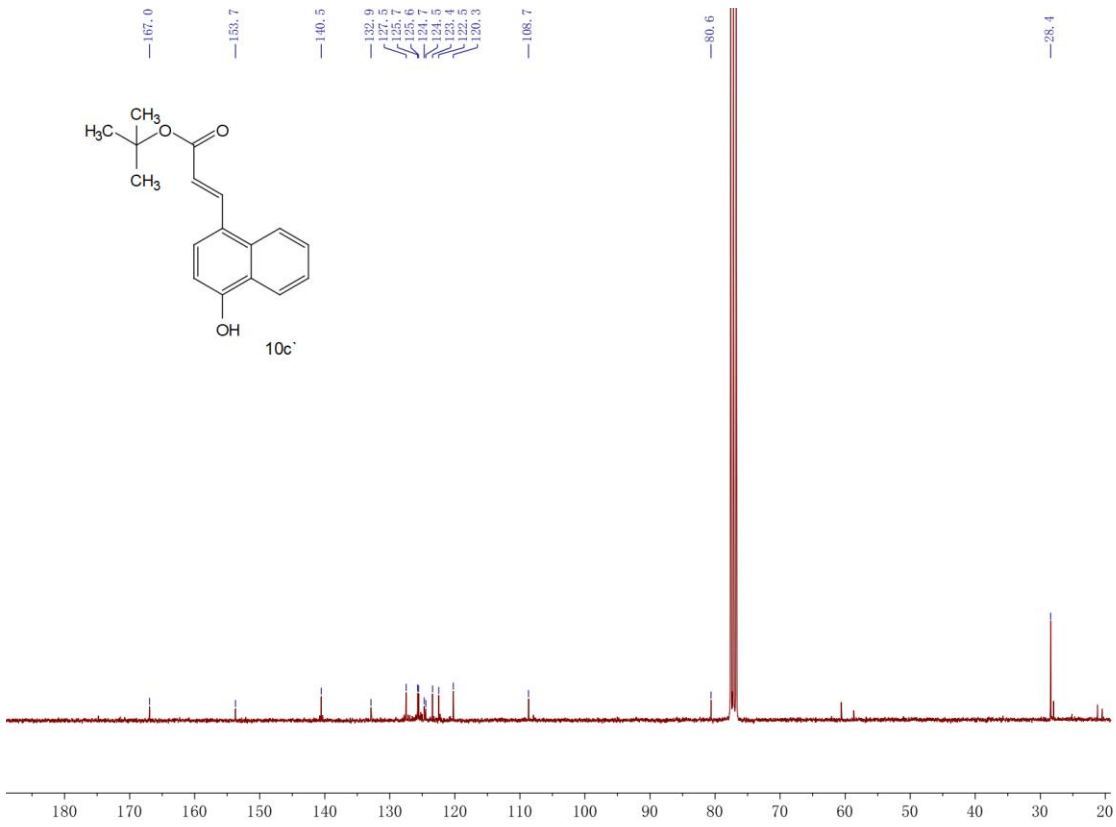

**Figure S39.** $^1$H NMR and $^{13}$C NMR of *tert*- butyl (*E*)-3-(4-hydroxynaphthalen-1-yl)acrylate (**10c'**) recorded in $CDCl_3$.

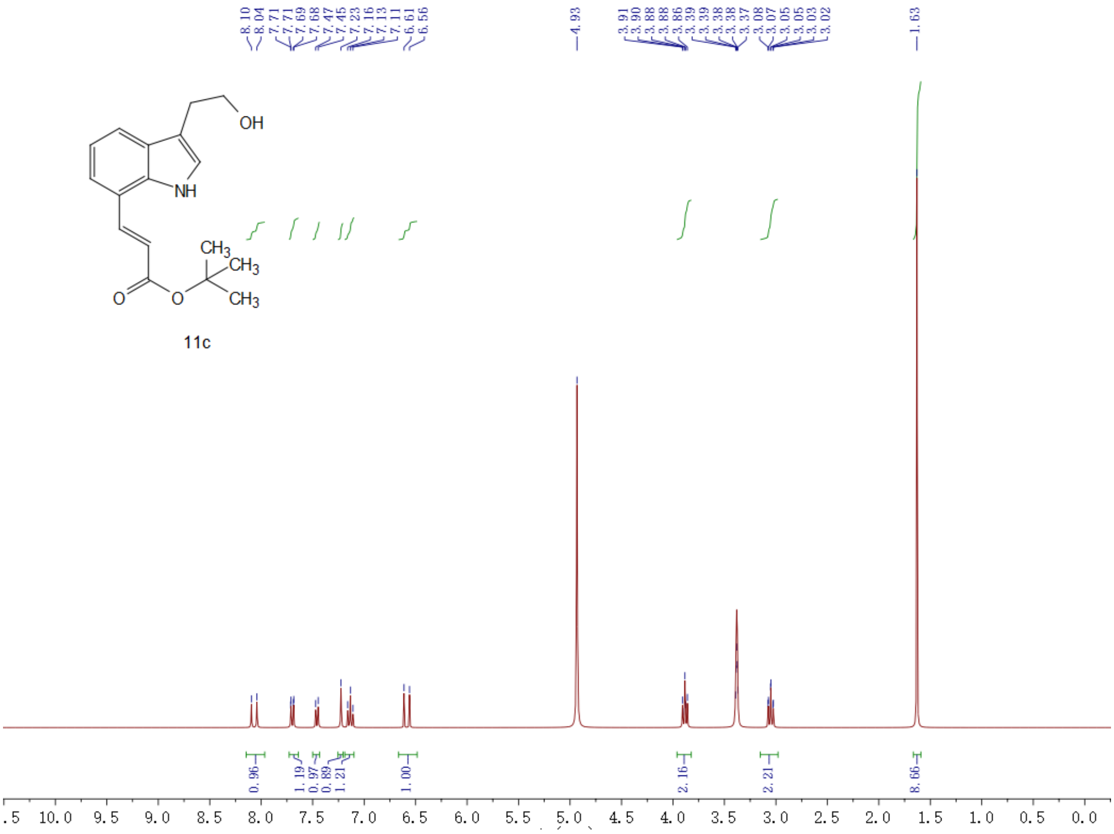


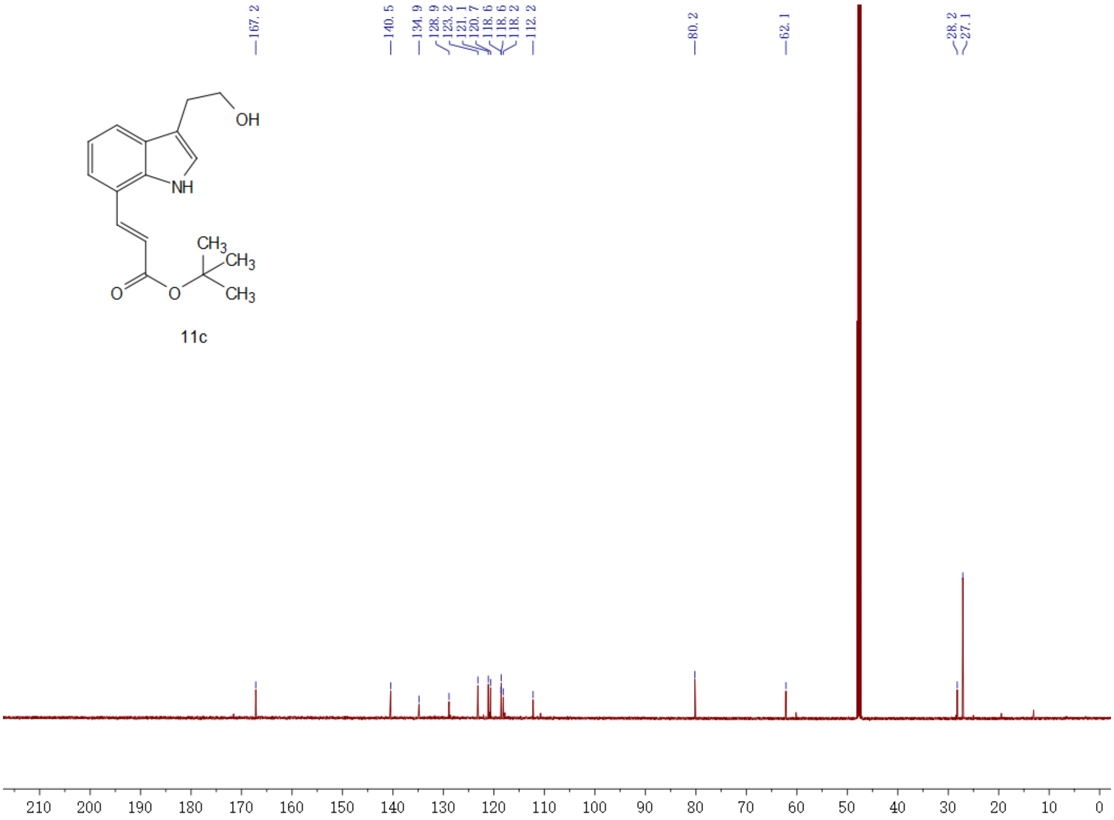


**Figure S40.** $^{1}H$ NMR and $^{13}C$ NMR of *tert*-butyl (*E*)-3-(3-(2-hydroxyethyl)-1H-indol-7-yl)acrylate (**11c**) recorded in $CD_3OD$.

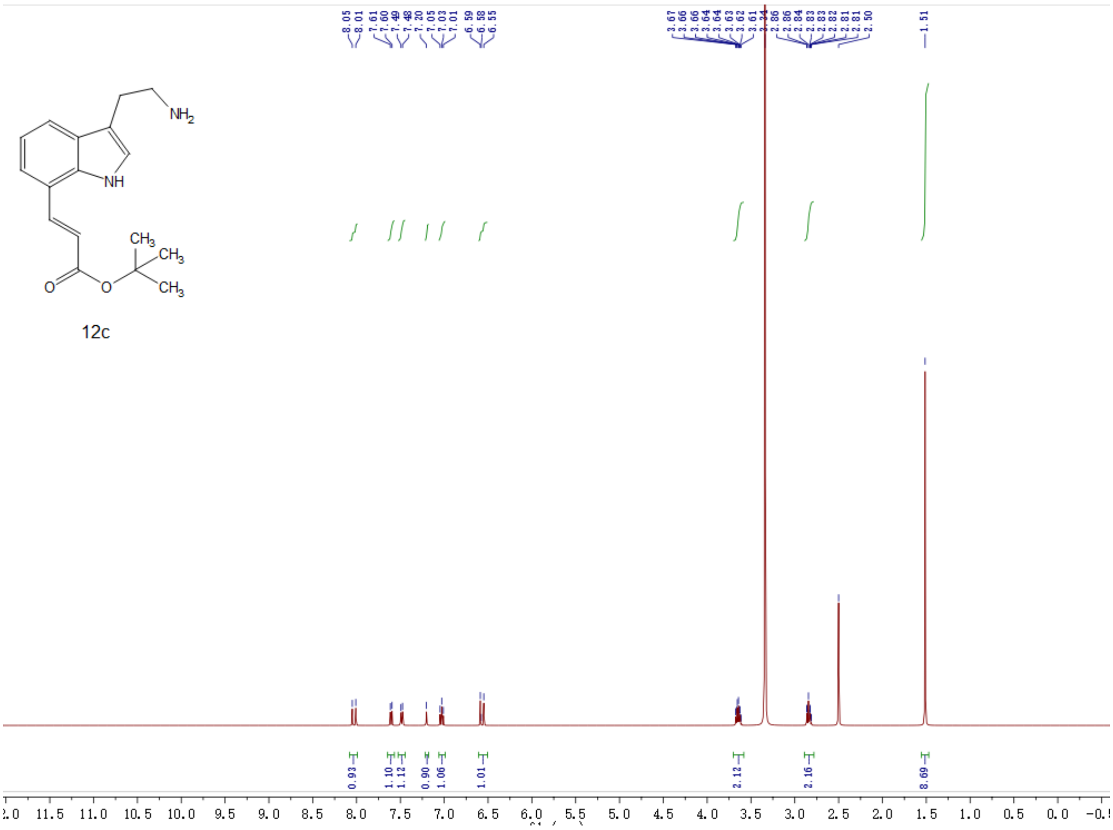

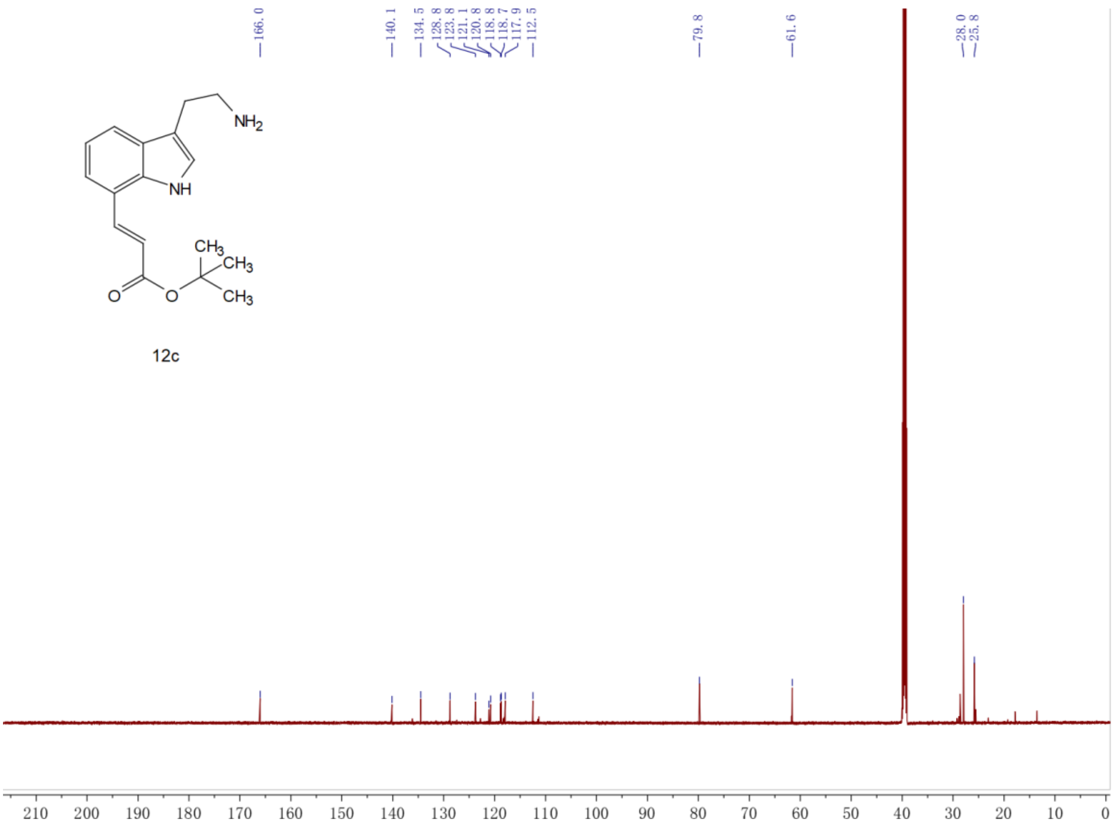

**Figure S41.** $^{1}$H NMR and $^{13}$C NMR of *tert*-butyl (*E*)-3-(3-(2-aminoethyl)-1H-indol-7-yl)acrylate (**12c**) recorded in DMSO-$d_6$.

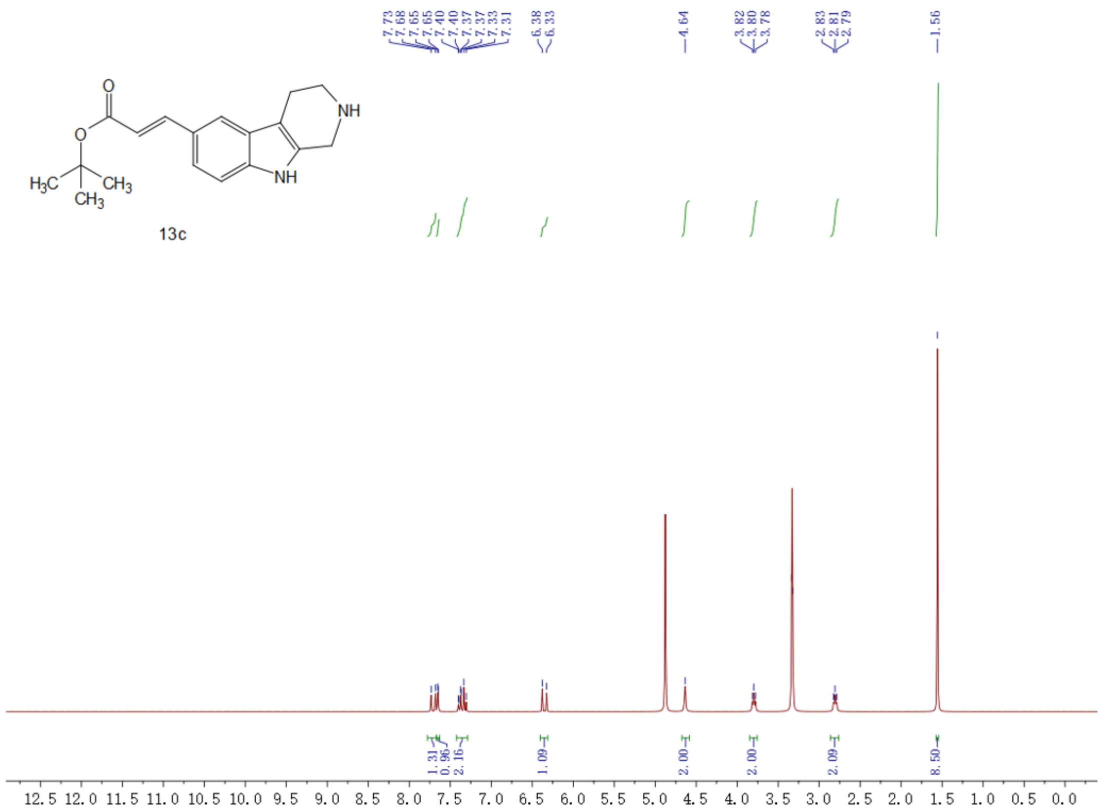


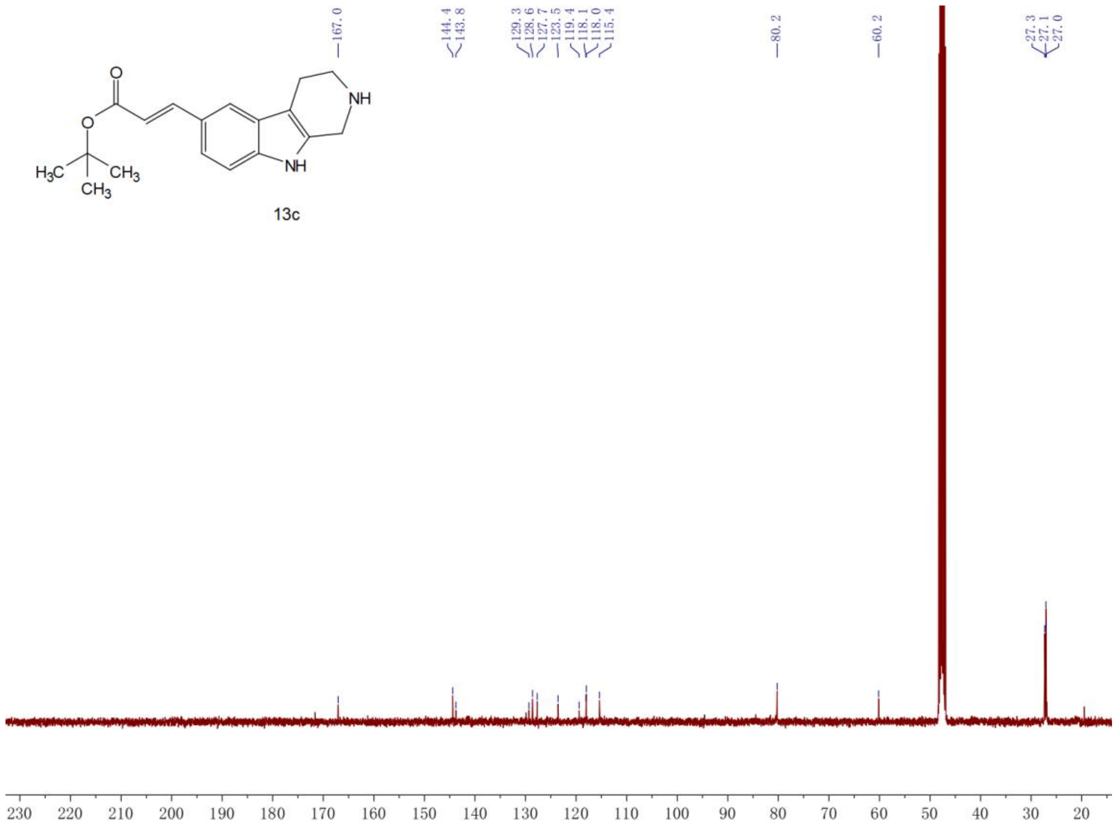


**Figure S42.** $^{1}H$ NMR and $^{13}C$ NMR of *tert*- butyl (*E*)-3-(2,3,4,9-tetrahydro-1H-pyrido[3,4-b]indol-6-yl)acrylate (**13c**) recorded in $CD_3OD$.

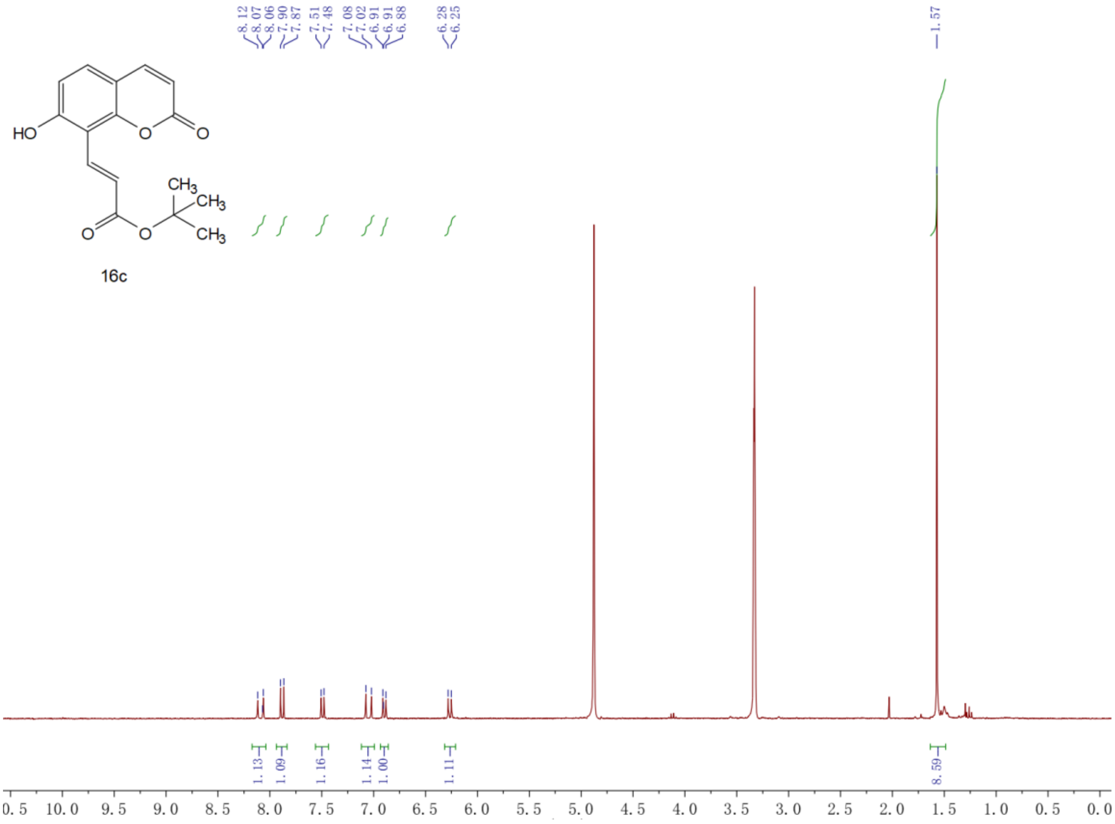

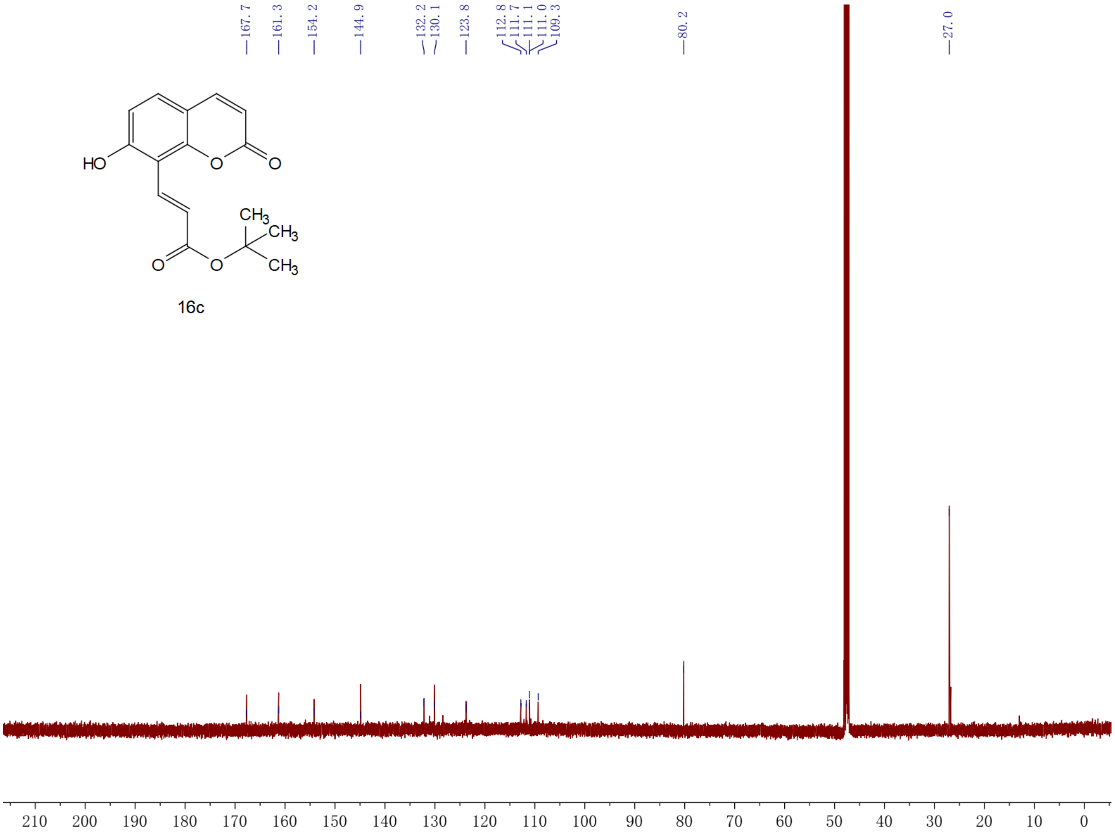

**Figure S43.** $^{1}H$ NMR and $^{13}C$ NMR of *tert*- butyl (*E*)-3-(7-hydroxy-2-oxo-2H-chromen-8-yl)acrylate (**16c**) recorded in $CD_3OD$.

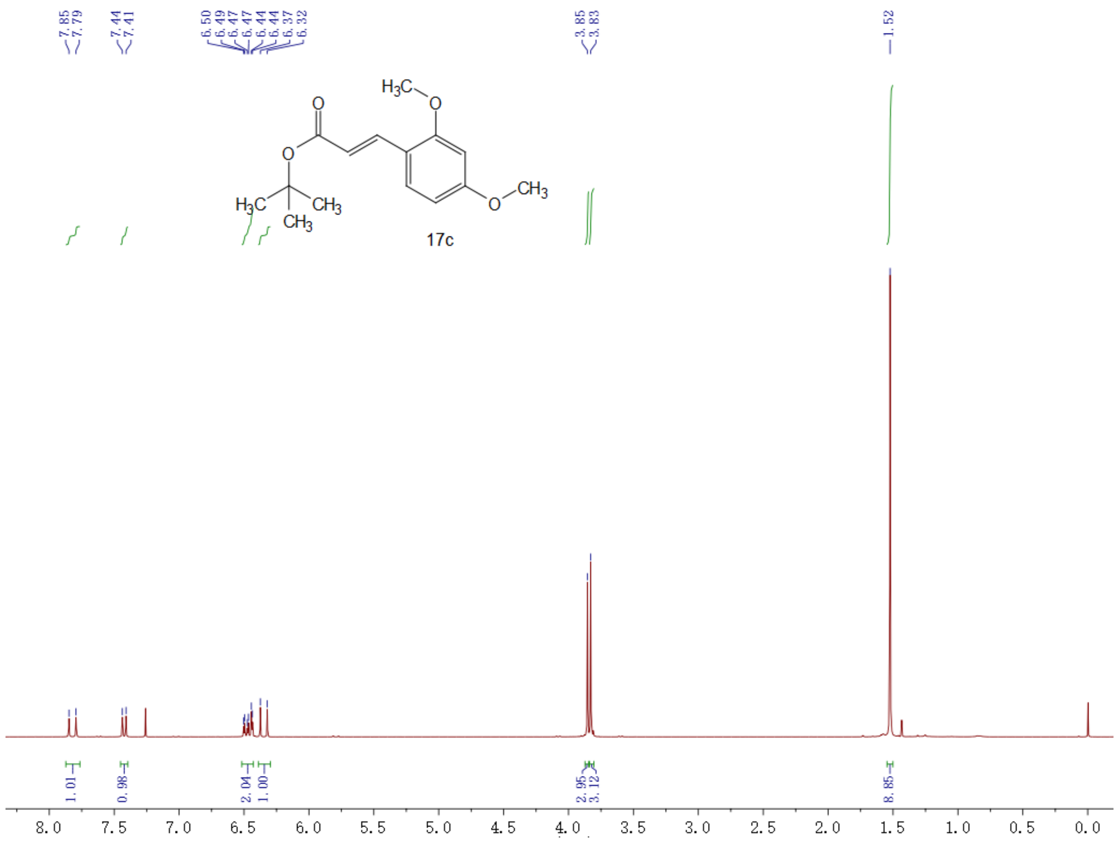


**Figure S44.** $^{1}$H NMR of *tert*- butyl (*E*)-3-(2,4-dimethoxyphenyl)propenoate (**17c**) recorded in $CDCl_3$.

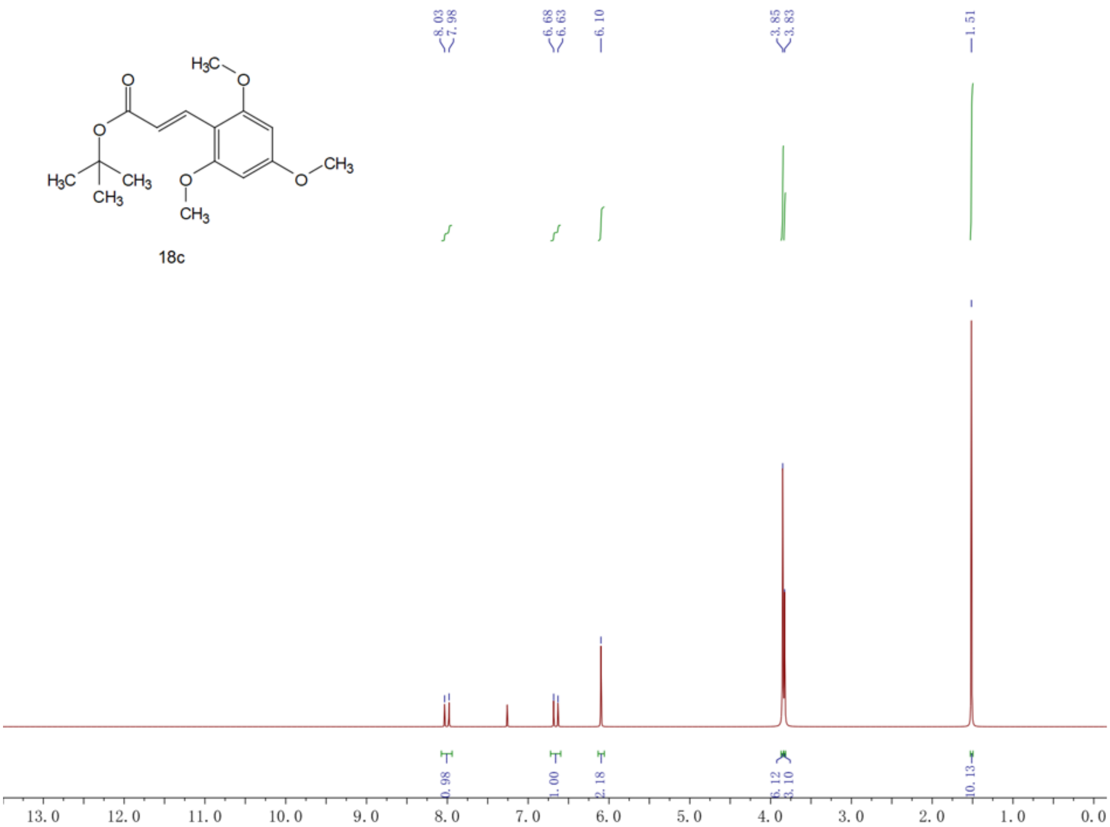


**Figure S45.** $^{1}$H NMR of *tert*- butyl (*E*)-3-(2,4,6-dimethoxyphenyl)propenoate (**18c**) recorded in $CDCl_3$.

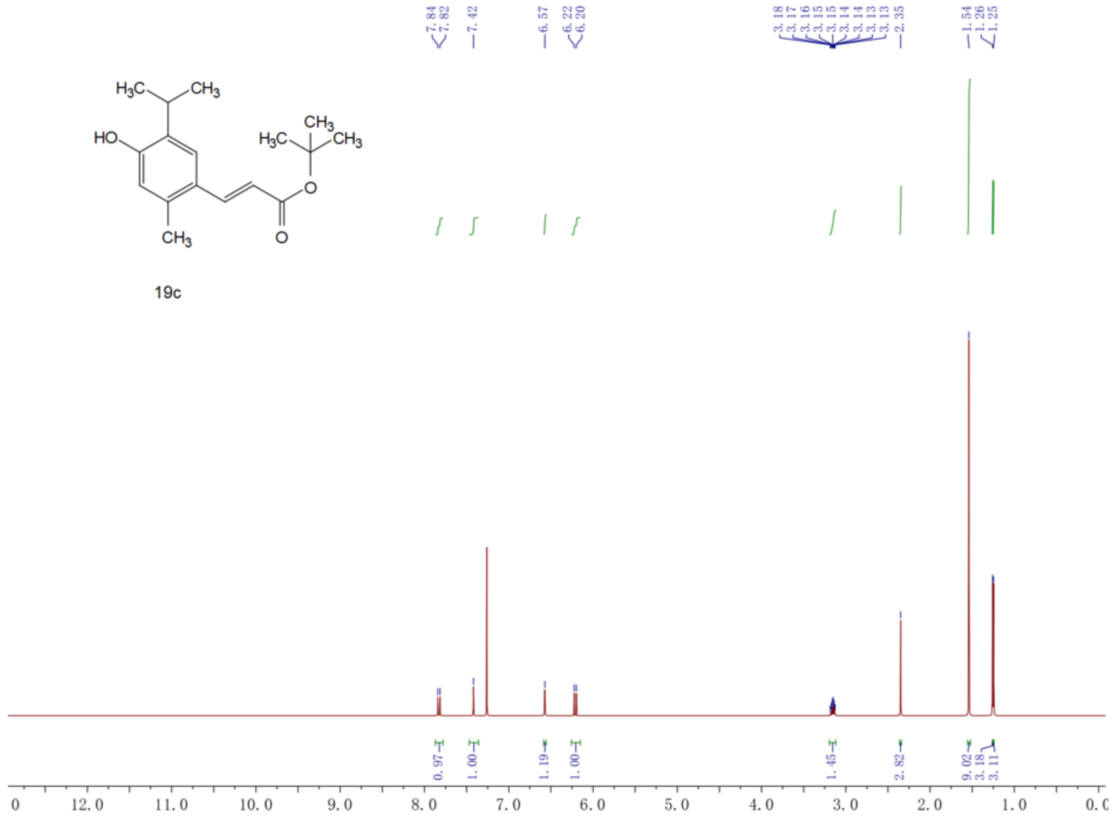


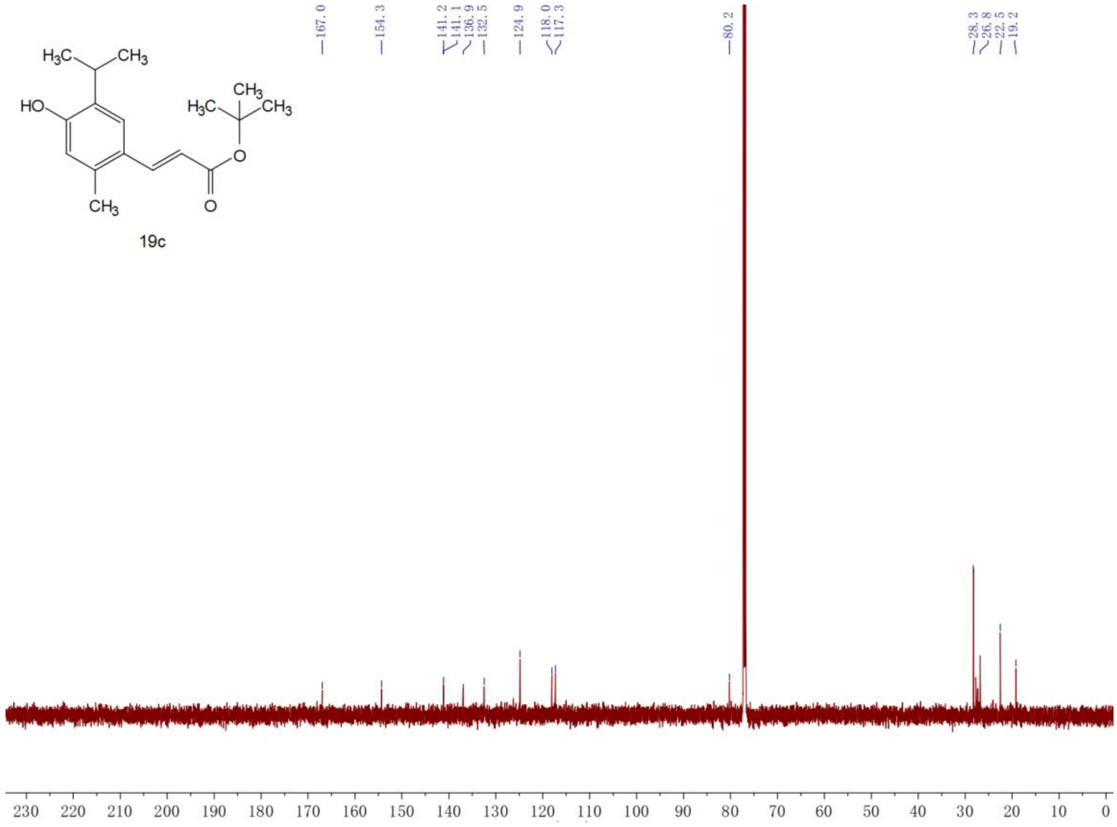


**Figure S46.** $^{1}$H NMR and $^{13}$C NMR of *tert*- butyl (*E*)-3-(4-hydroxy-5-isopropyl-2-methylphenyl)acrylate (**19c**) recorded in $CDCl_3$.

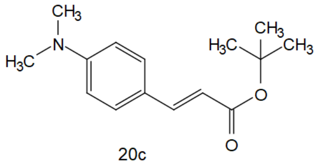

20c

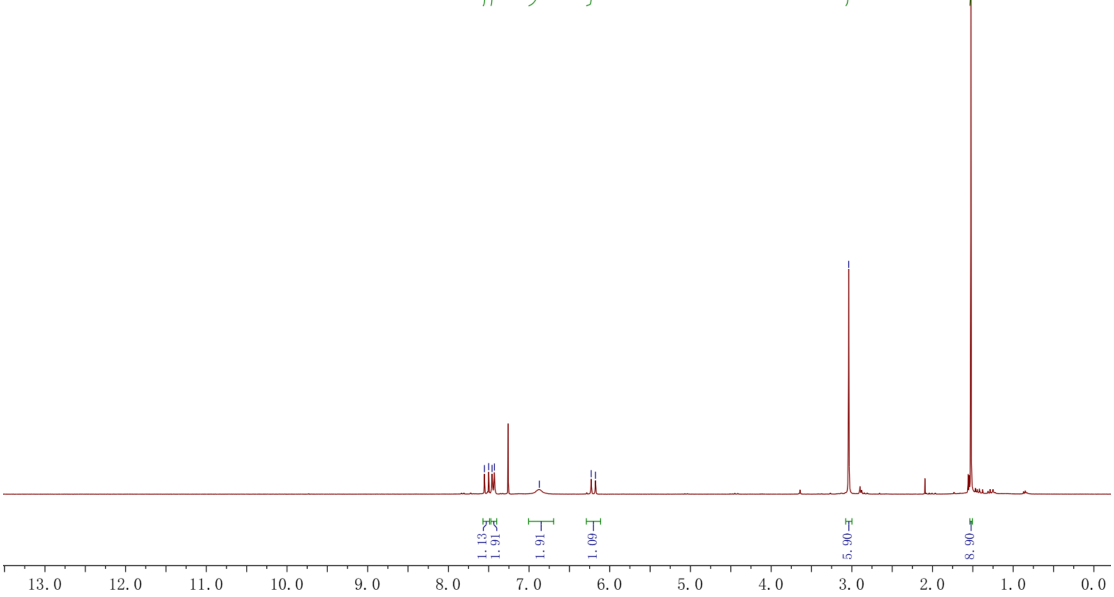


**Figure S47.** $^{1}$H NMR of *tert*- butyl (*E*)-3-(4-(dimethylamino)phenyl)acrylate (**20c**) recorded in $CDCl_3$.

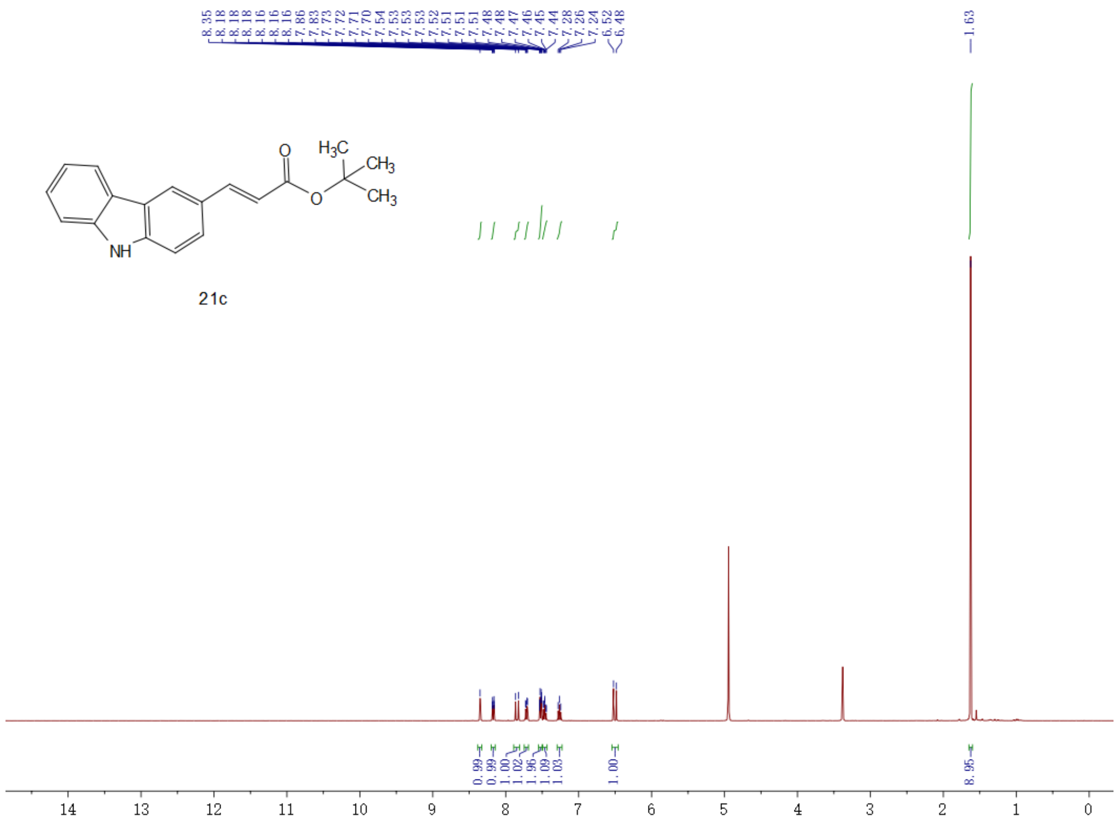

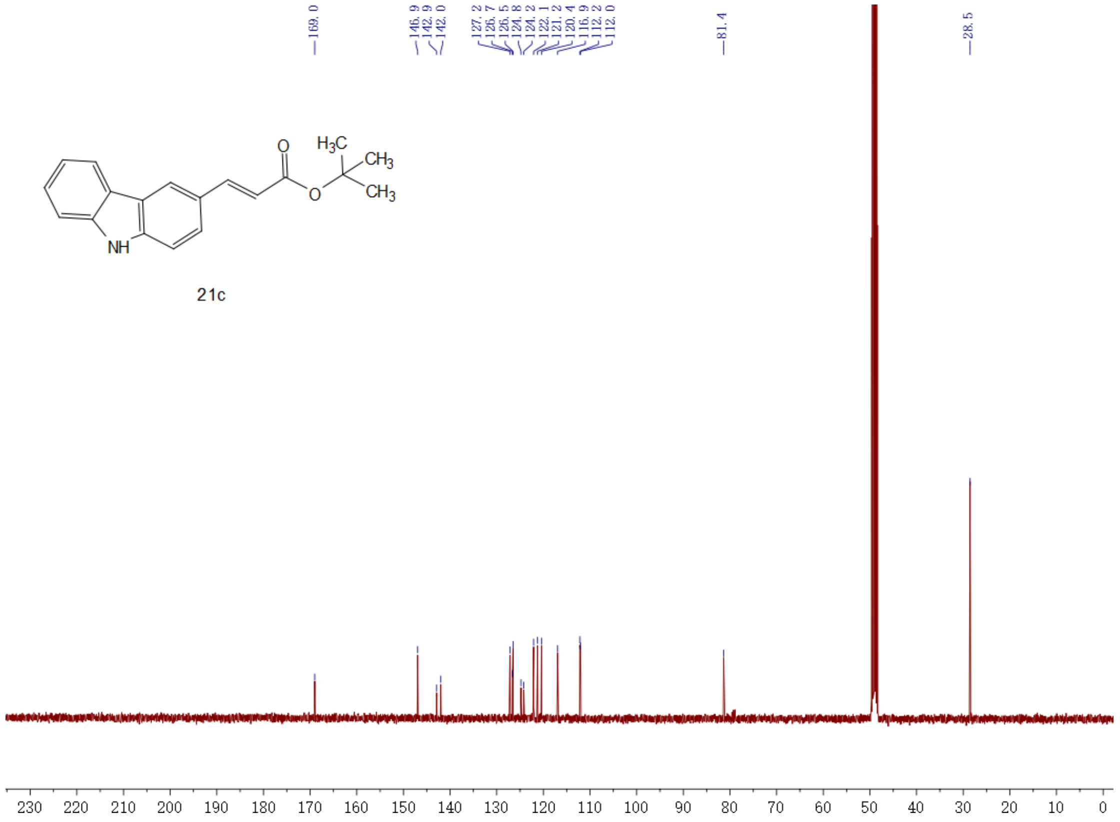

**Figure S48.** $^{1}$H NMR and $^{13}$C NMR of *tert*- butyl (*E*)-3-(9H-carbazol-6-yl)acrylate (**21c**) recorded in DMSO-$d_6$.

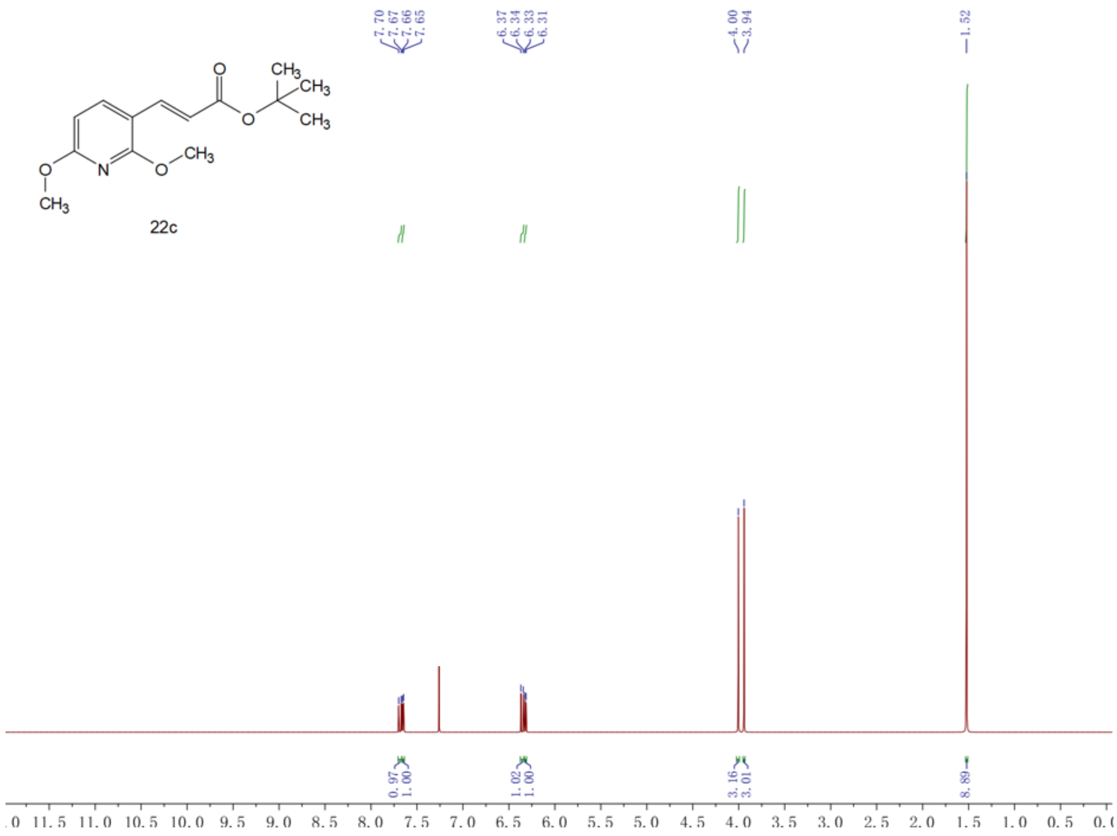


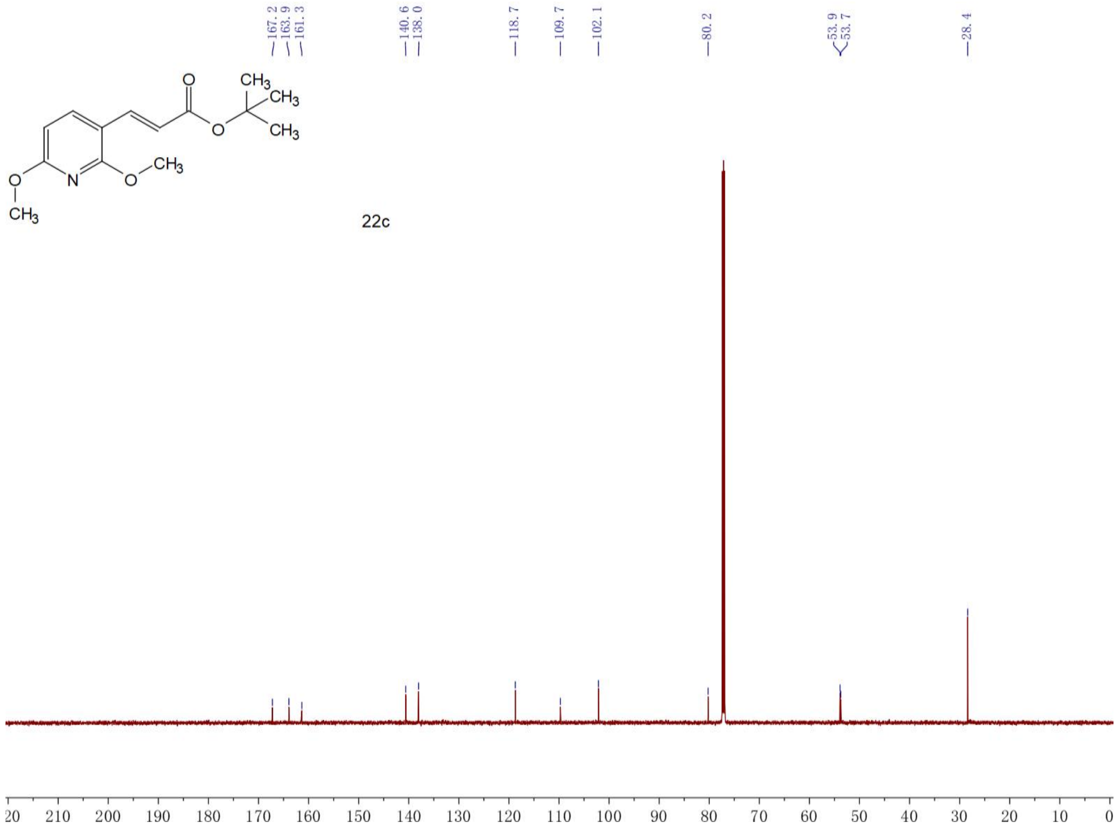


**Figure S49.** $^{1}H$ NMR and $^{13}C$ NMR of *tert*- butyl (*E*)-3-(2,6-dimethoxypyridin-3-yl)acrylate (**22c**) recorded in $CDCl_3$.

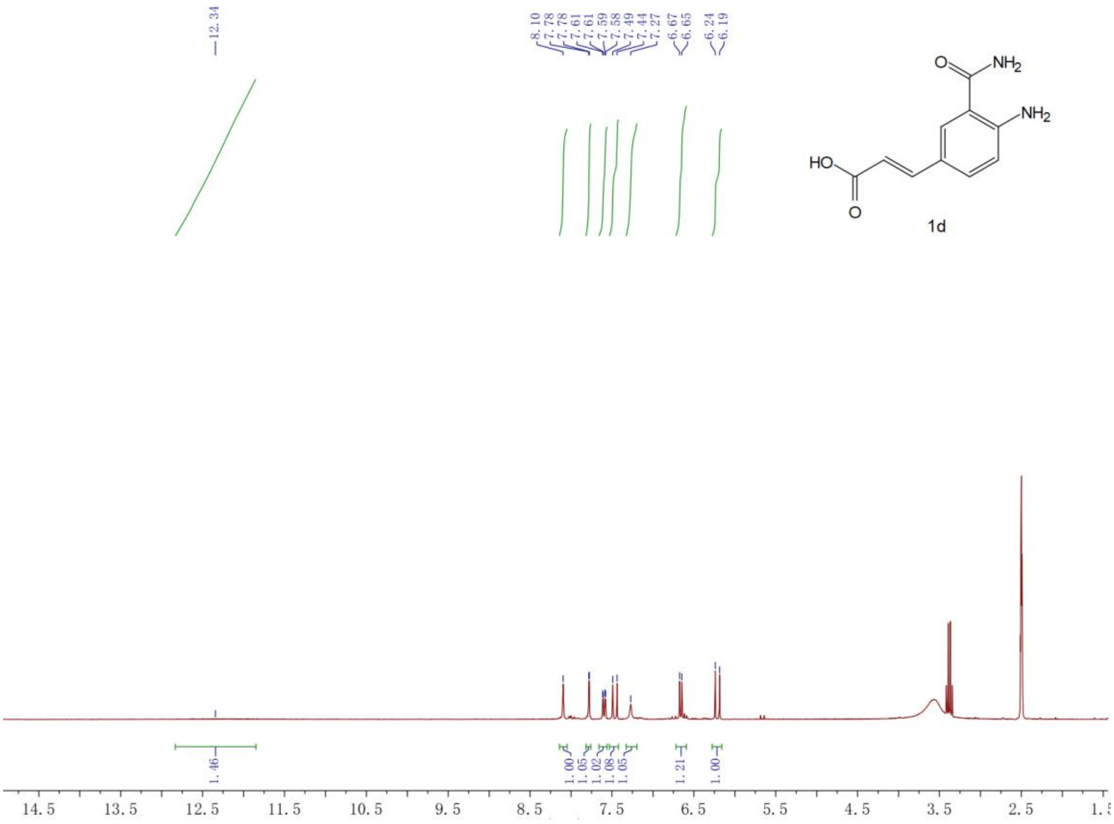


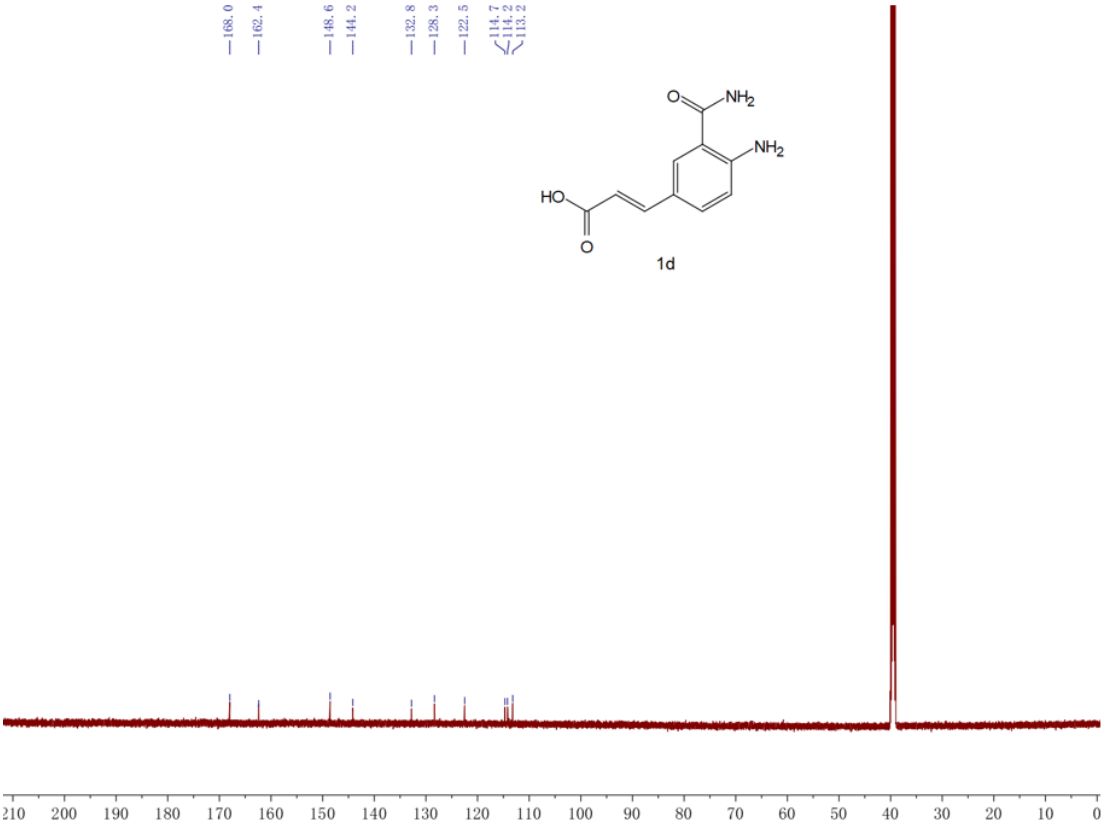


**Figure S50.** $^1$H NMR of (*E*)-3-(4-(piperazin-1-yl)phenyl)acrylic acid (**1d**) recorded in DMSO-$d_6$.

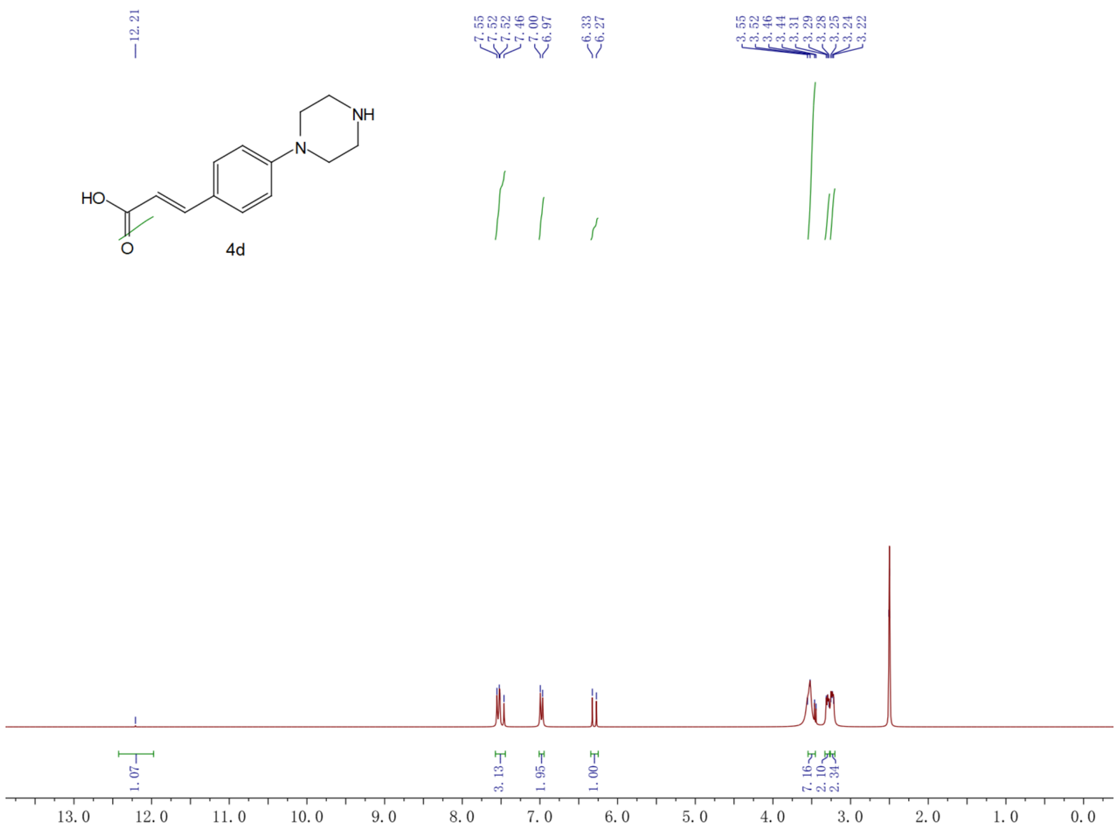

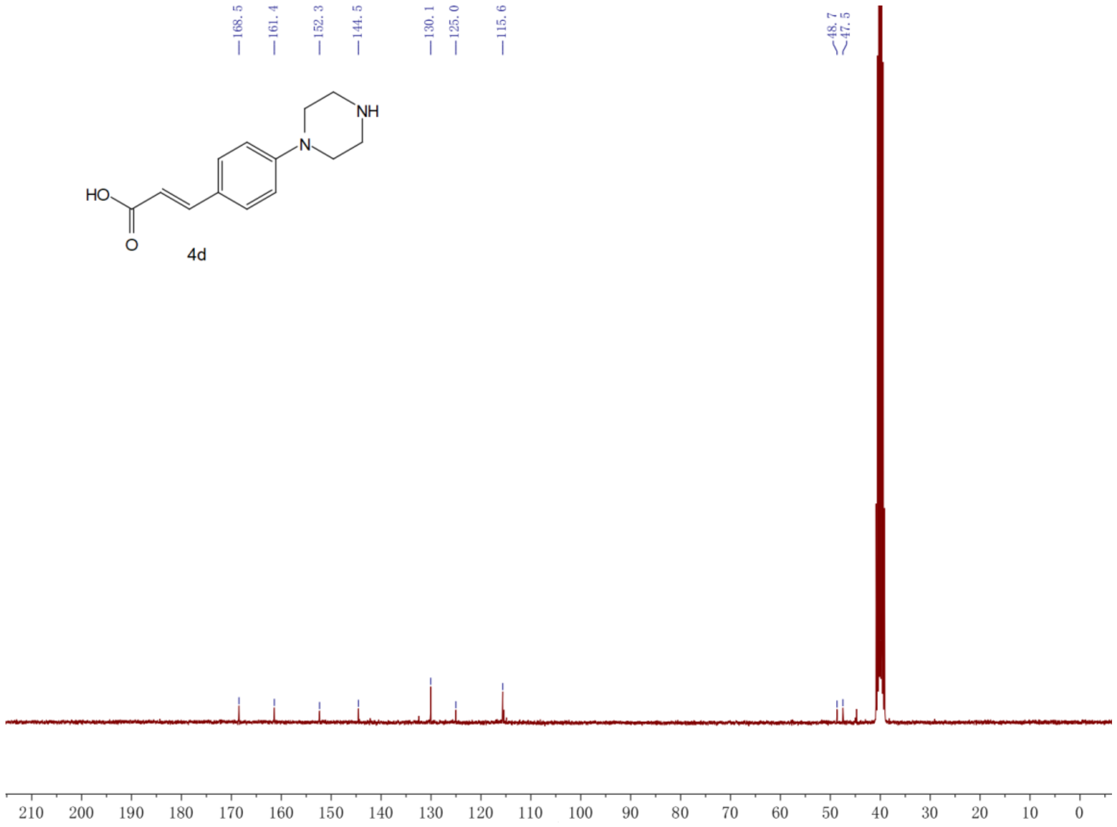

**Figure S51.** $^1$H NMR and $^{13}$C NMR of (*E*)- 3-(4-(piperazin-1-yl)phenyl)acrylic acid (**4d**) recorded in DMSO-$d_6$.

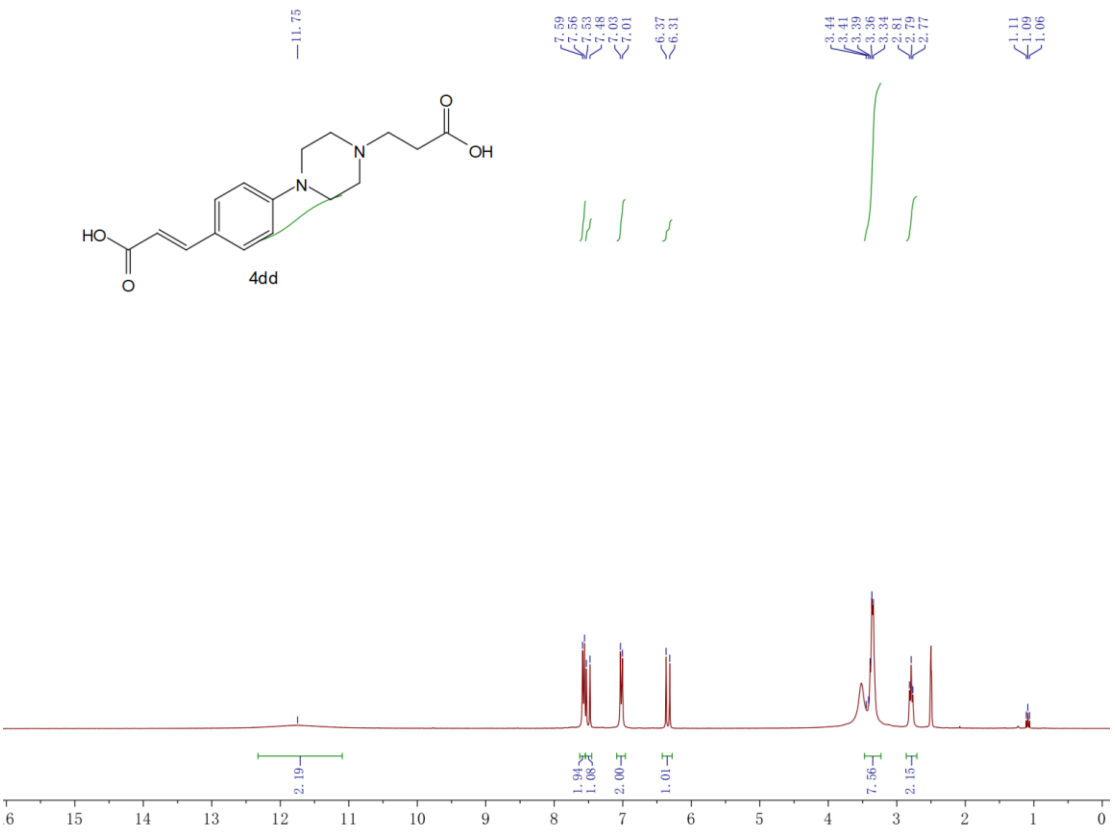


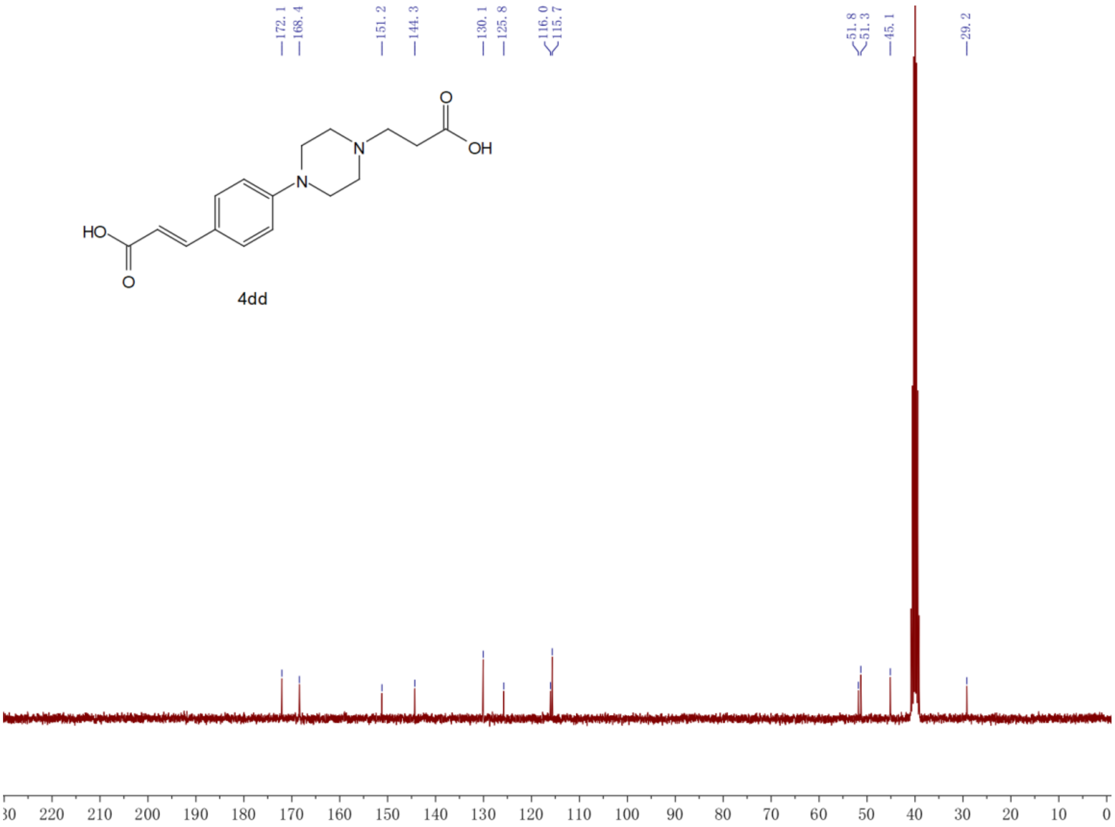


**Figure S52.** $^{1}H$ NMR and $^{13}C$ NMR of (*E*)- 3-(4-(4-(2-carboxyethyl)piperazin-1-yl)phenyl)acrylic acid (**4dd**) recorded in DMSO-$d_6$.

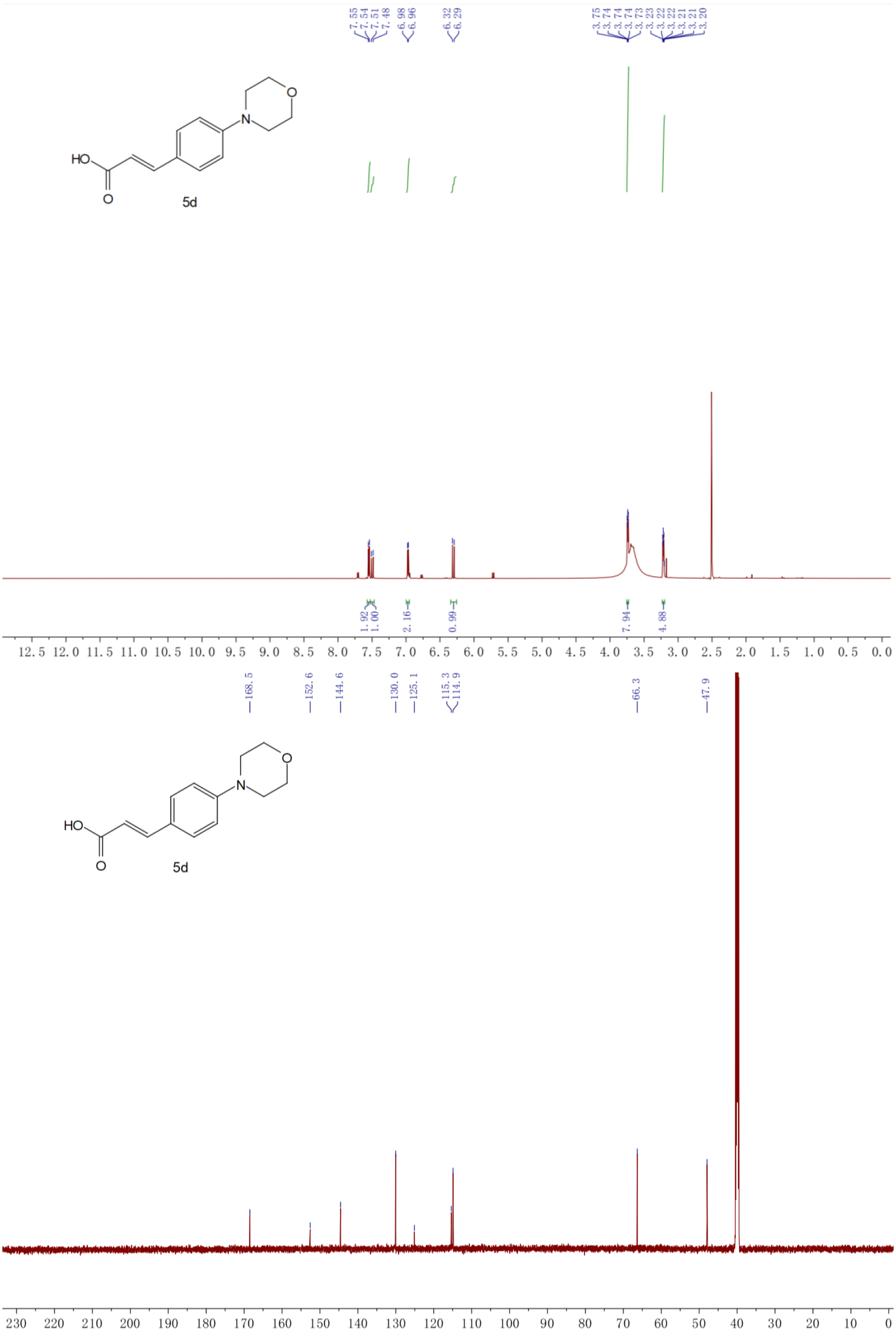


**Figure S53.** $^{1}H$ NMR and $^{13}C$ NMR of *p*-Morpholino-cinnamic acid (**5d**) recorded in DMSO-$d_6$.

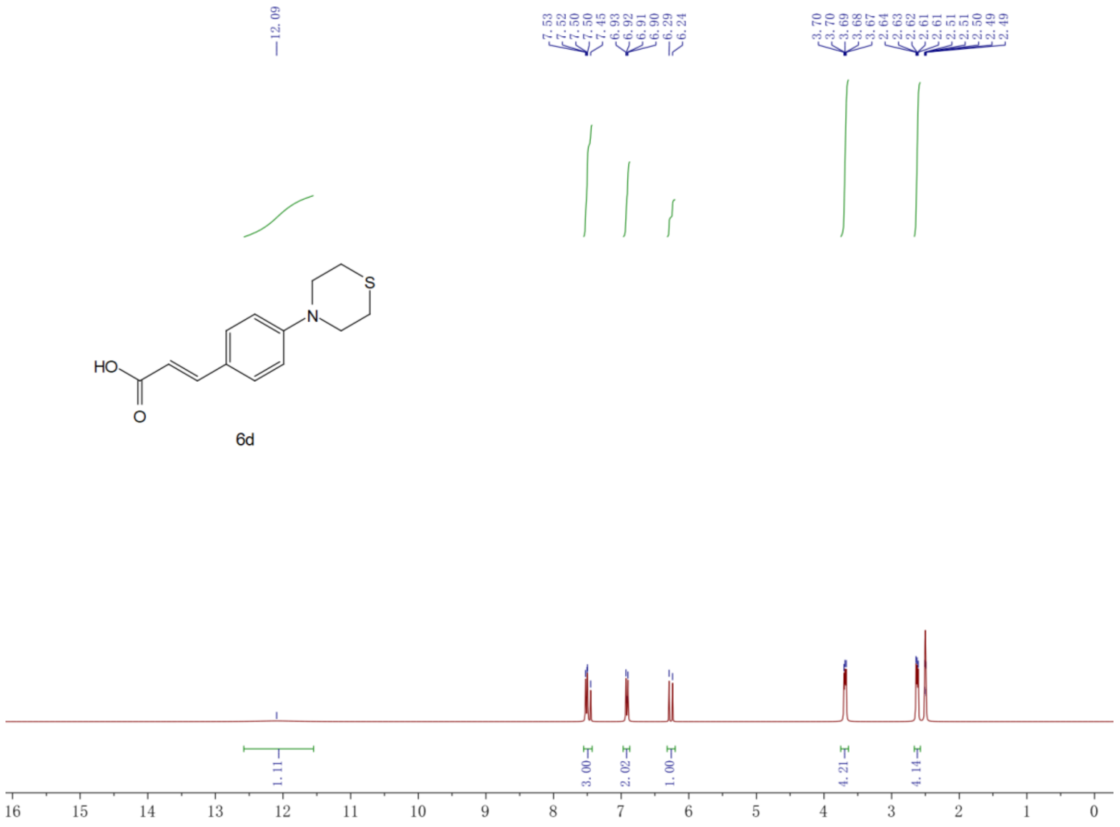


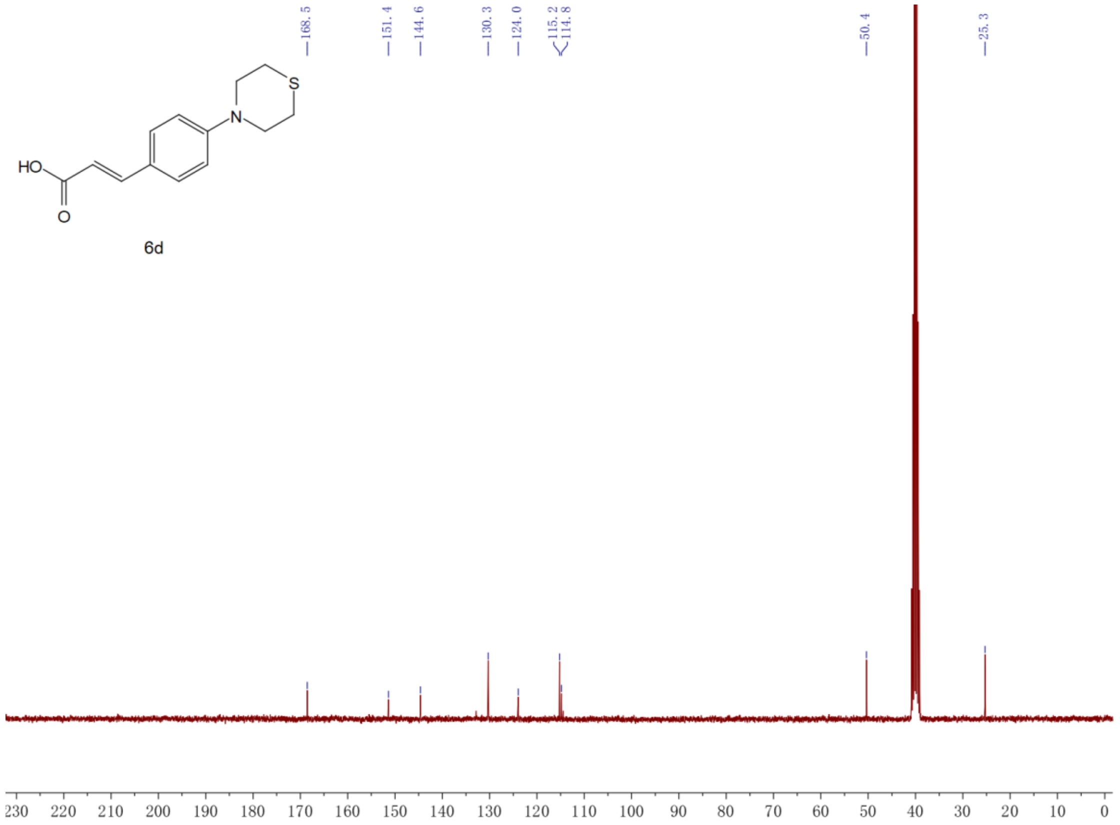


**Figure S54.** $^{1}$H NMR and $^{13}$C NMR of (*E*)- 3-(4-thiomorpholinophenyl)acrylic acid (**6d**) recorded in DMSO-$d_6$.

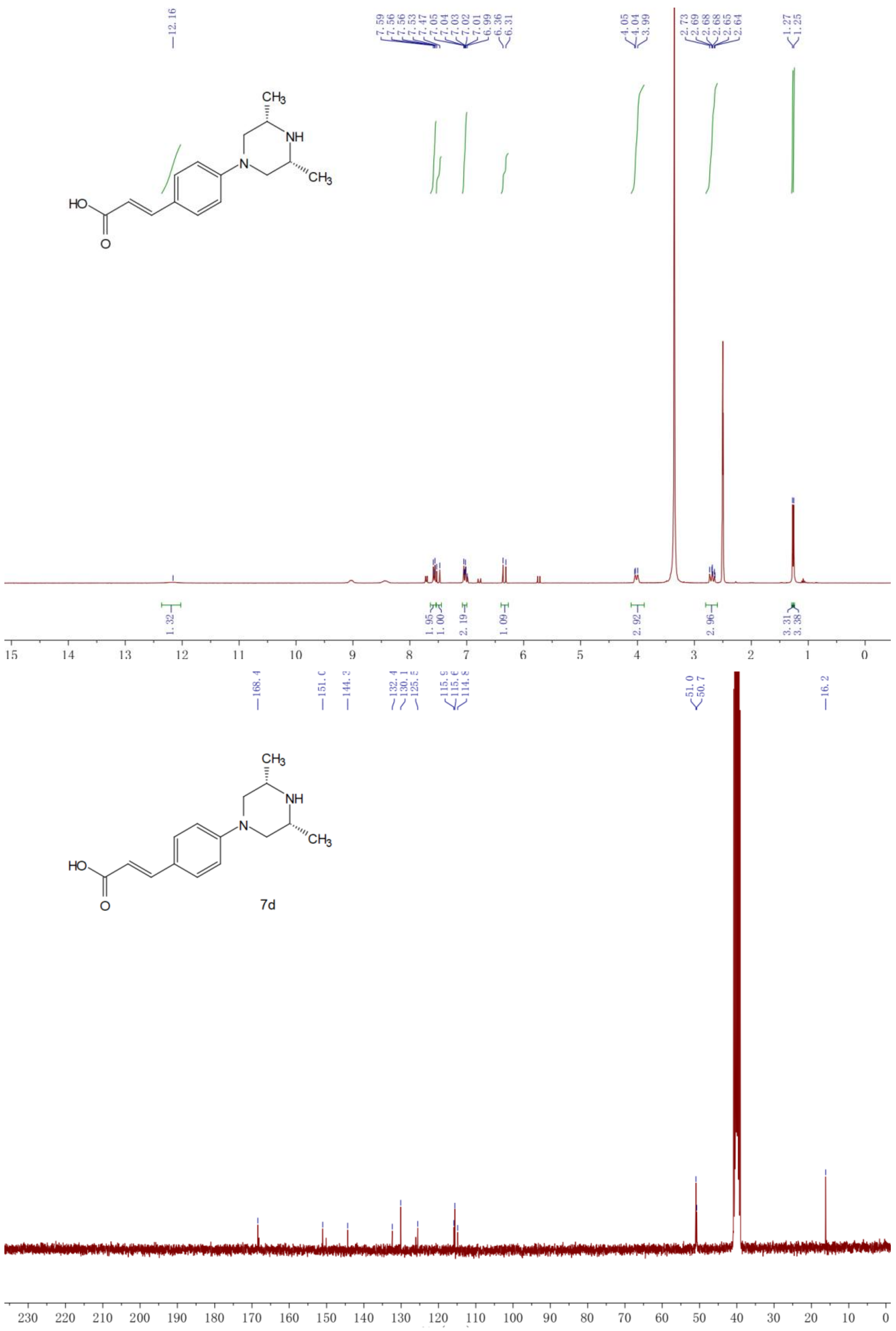


**Figure S55.** $^1$H NMR and $^{13}$C NMR of (*E*)- 3-(4-((3R,5S)-3,5-dimethylpiperazin-1-yl)phenyl)acrylic acid (**7d**) recorded in DMSO-$d_6$.

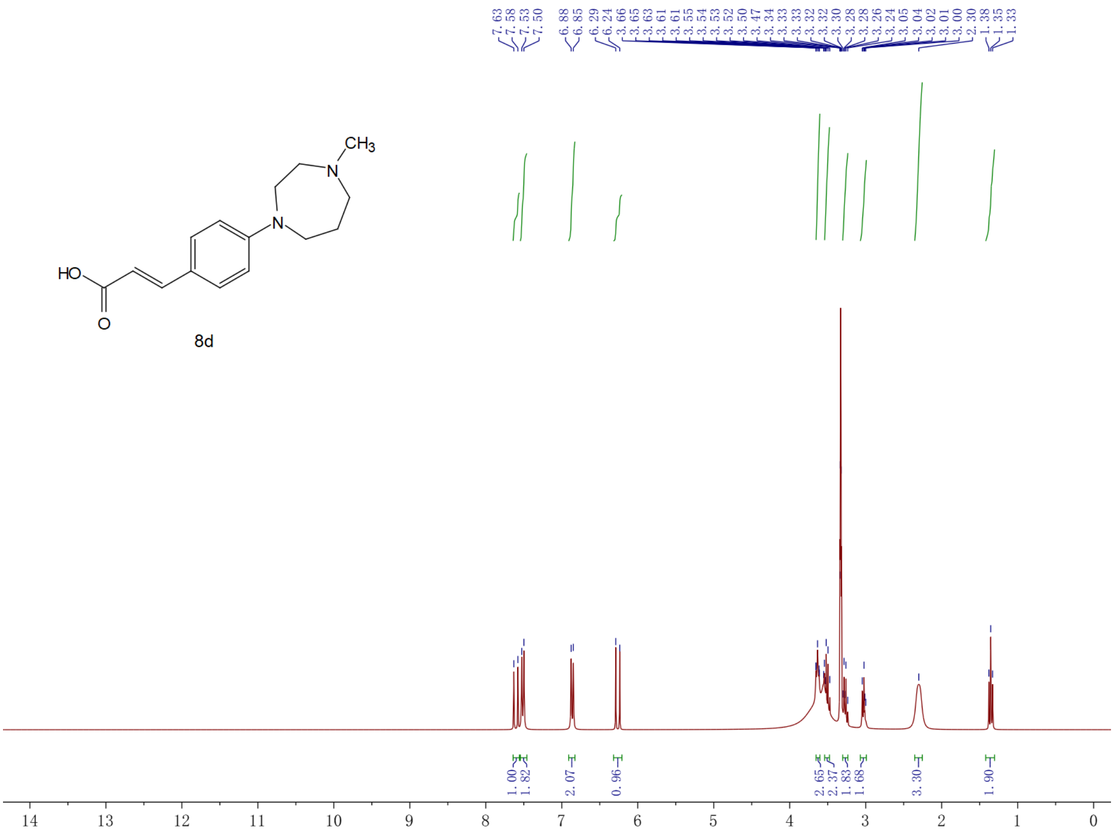

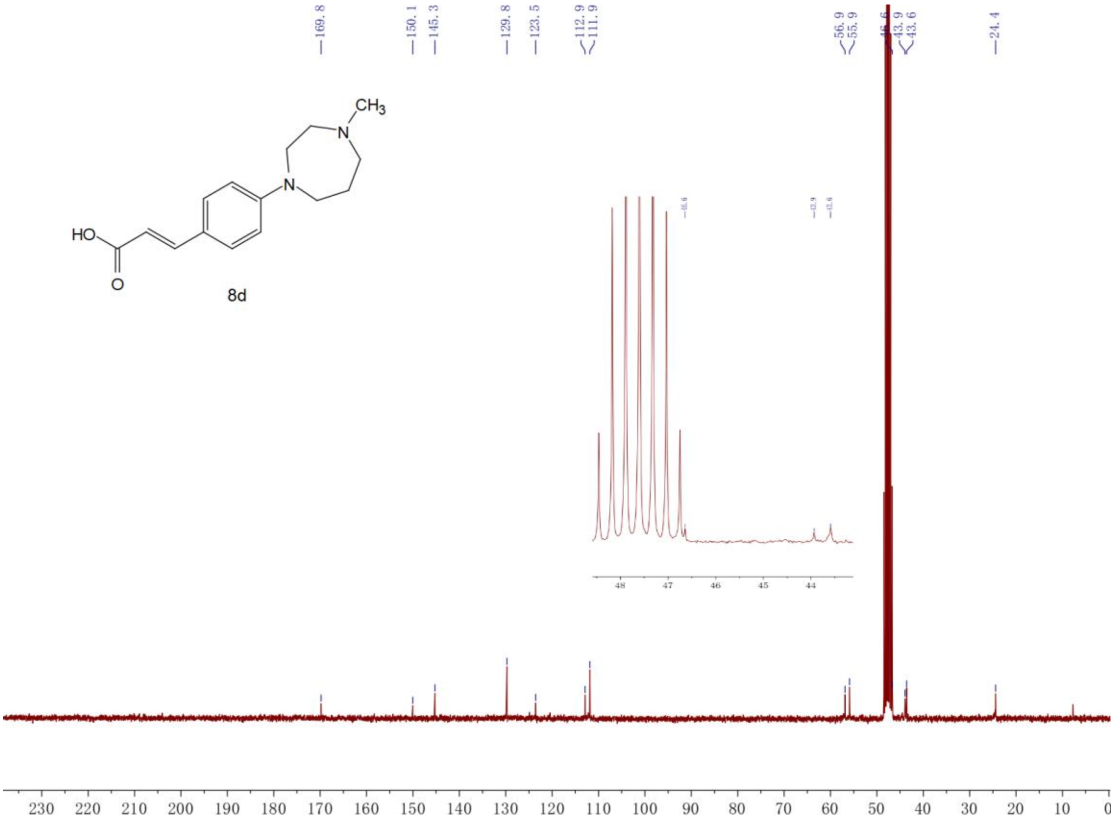

**Figure S56.** $^{1}$H NMR and $^{13}$C NMR of (*E*)- 3-(4-(4-methyl-1,4-diazepan-1-yl)phenyl)acrylic acid (**8d**) recorded in $CD_3OD$.

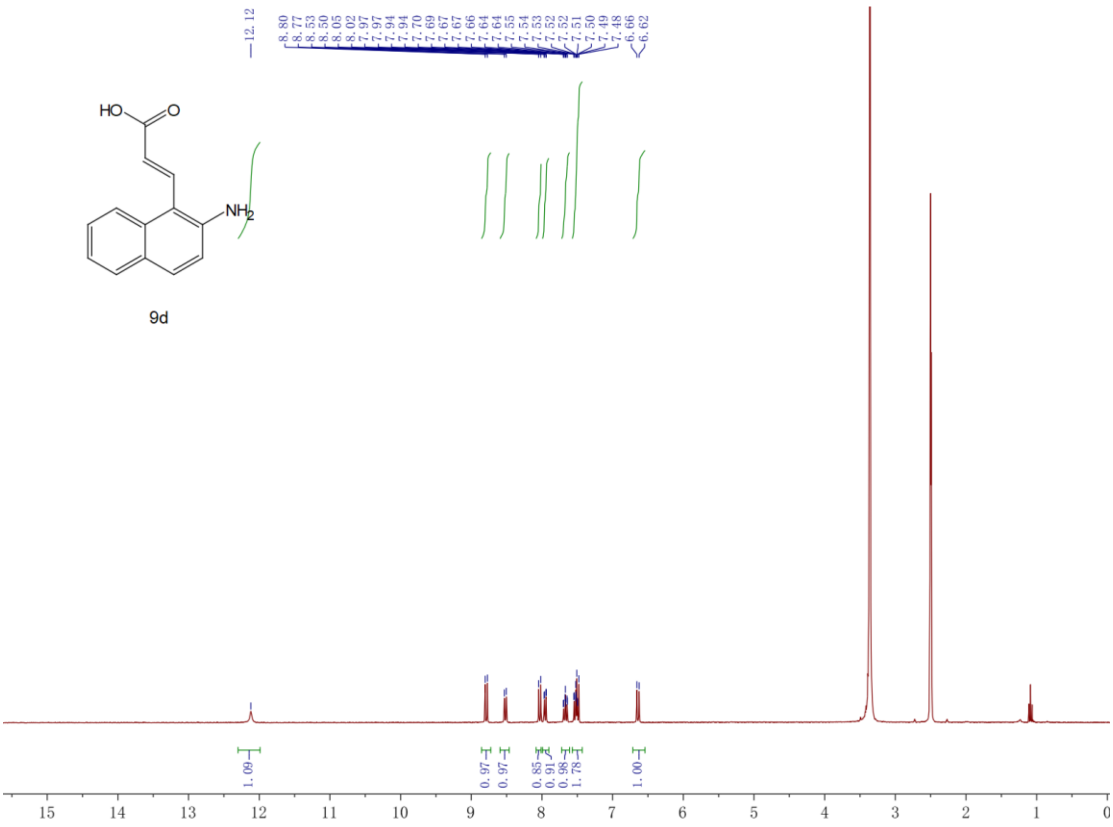

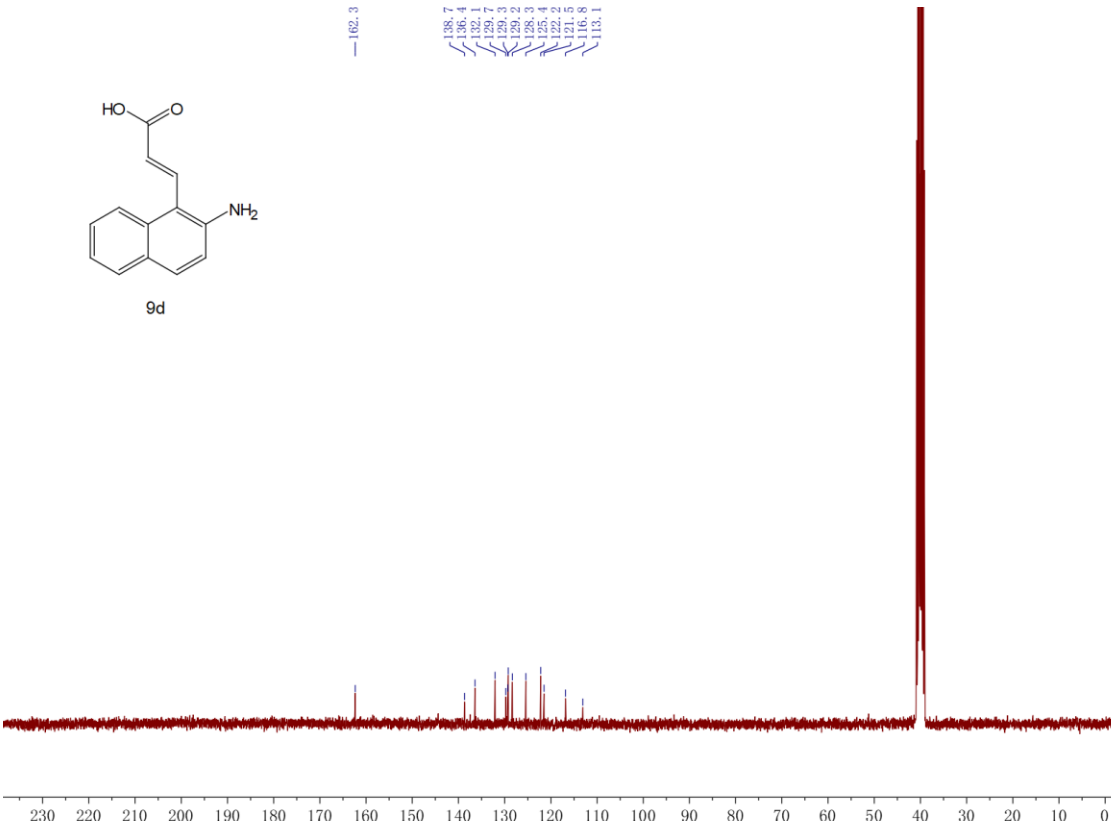

**Figure S57.** $^{1}$H NMR and $^{13}$C NMR of (*E*)- 3-(2-aminonaphthalen-1-yl)acrylic acid (**9d**) recorded in DMSO-$d_6$.

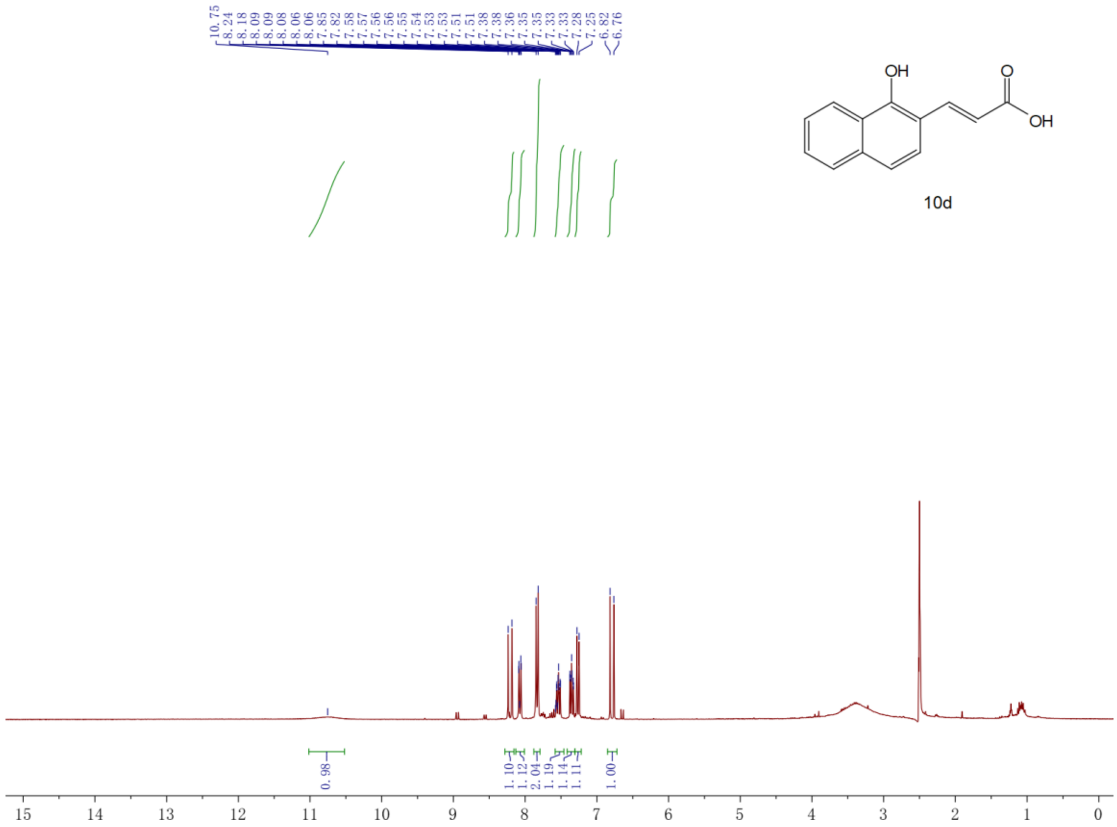

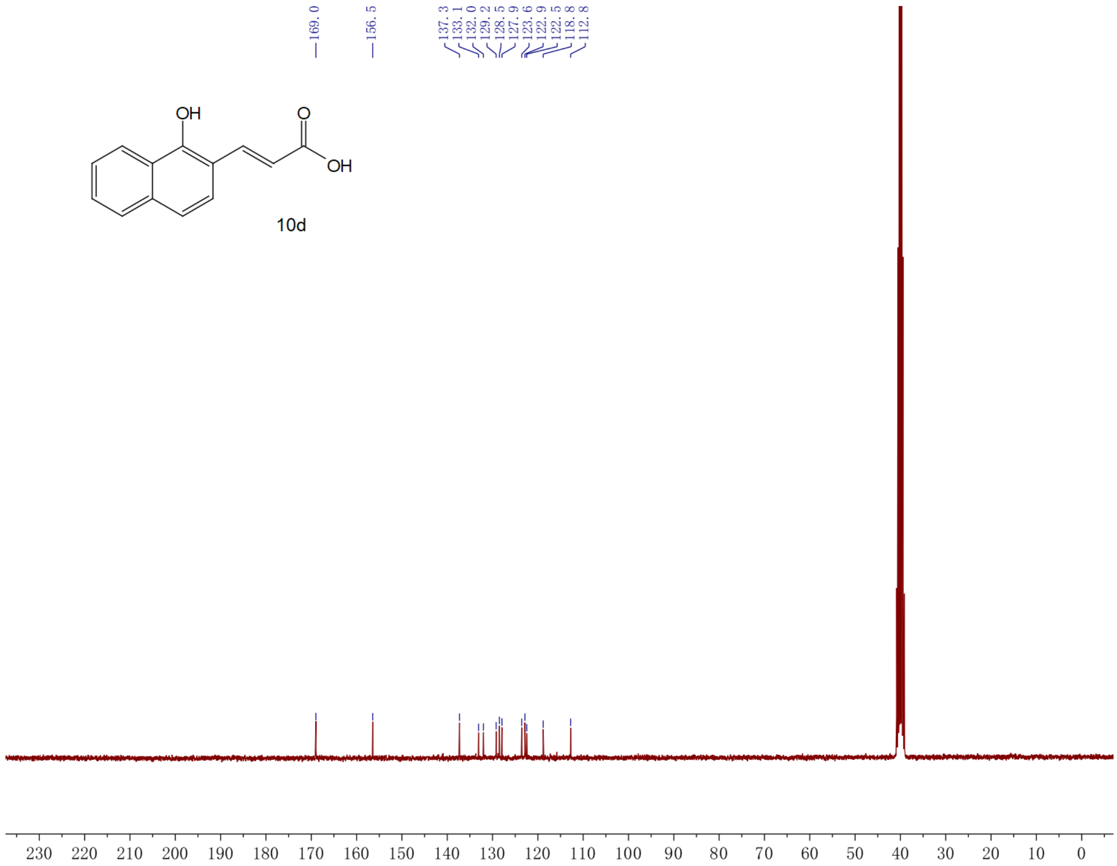

**Figure S58.** $^{1}H$ NMR and $^{13}C$ NMR of (*E*)- 3-(1-hydroxynaphthalen-2-yl)acrylic acid (**10d**) recorded in DMSO-$d_6$.

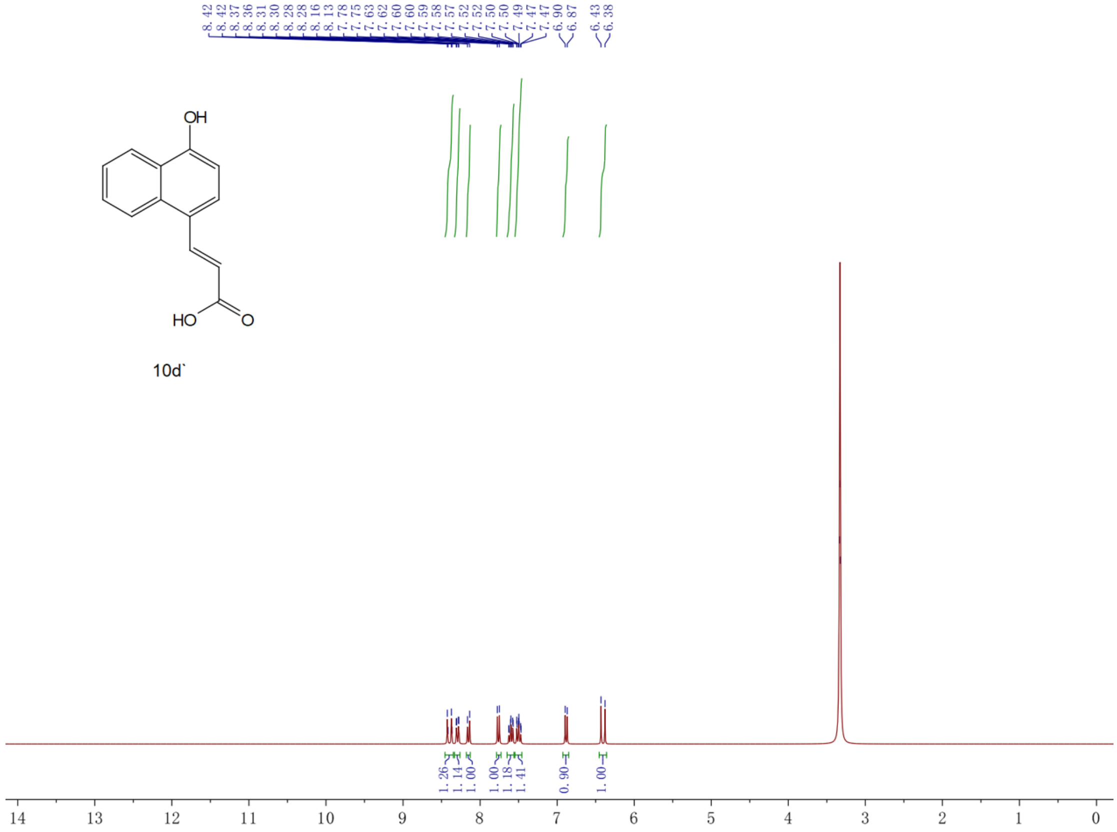

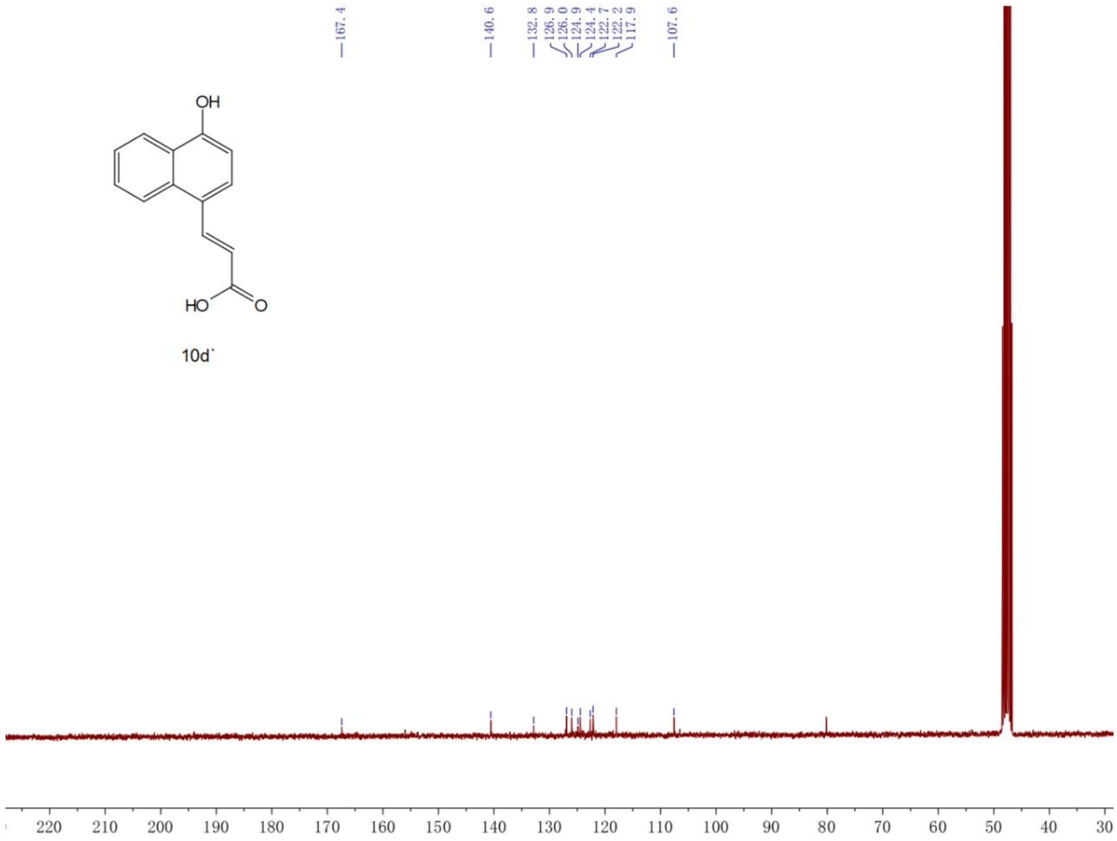

**Figure S59.** $^1$H NMR and $^{13}$C NMR of (*E*)- 3-(4-hydroxynaphthalen-1-yl)acrylic acid (**10d'**) recorded in $CD_3OD$.

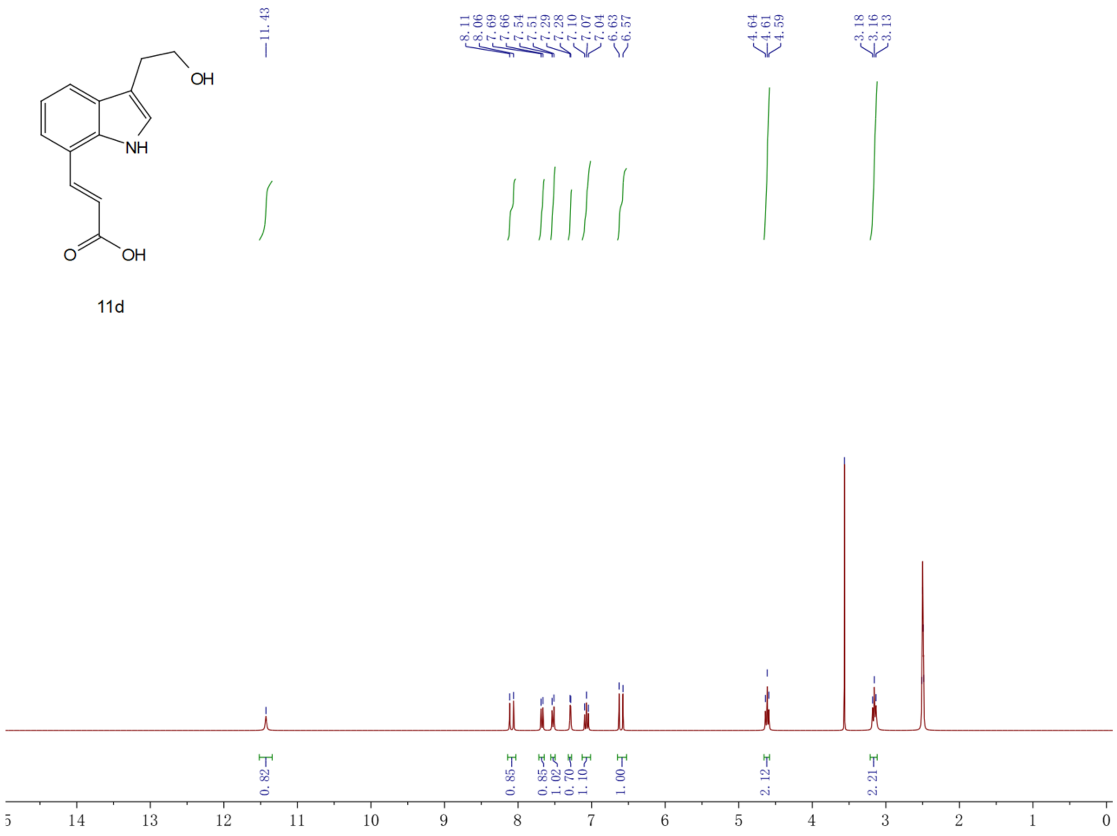


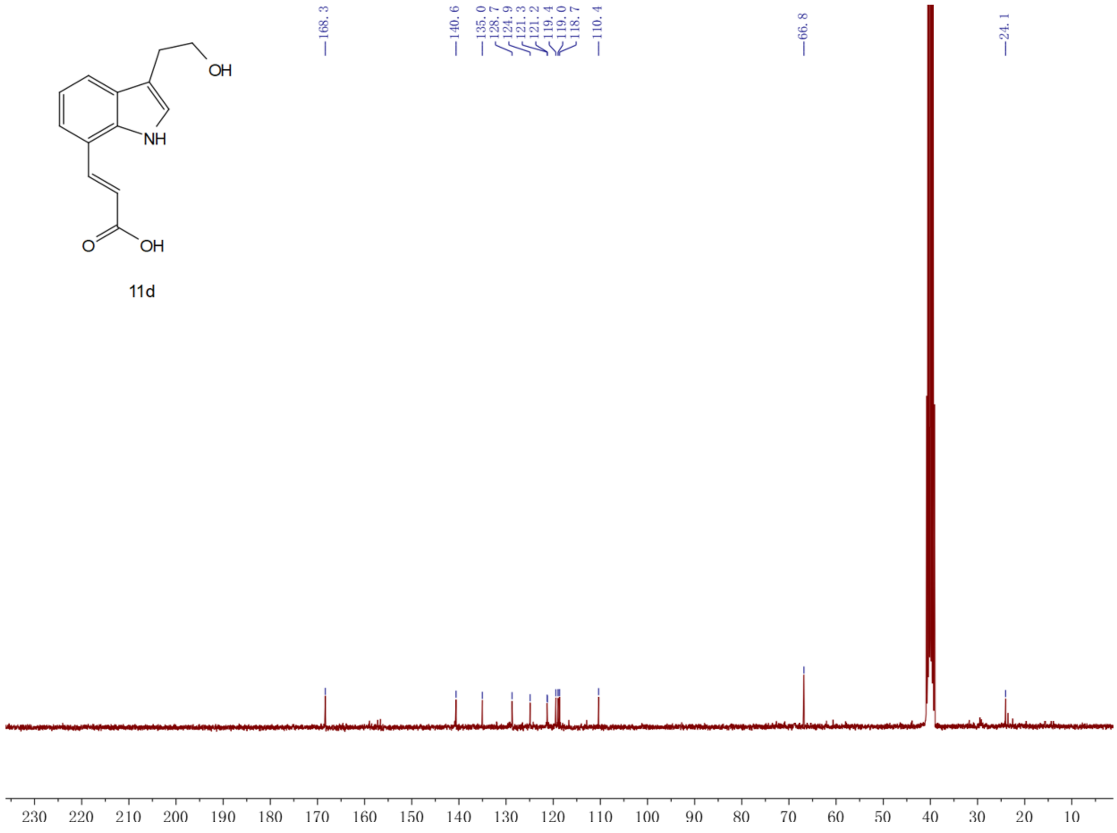


**Figure S60.** $^{1}H$ NMR and $^{13}C$ NMR of (*E*)- 3-(3-(2-hydroxyethyl)-1H-indol-7-yl)acrylic acid (**11d**) recorded in DMSO-$d_6$.

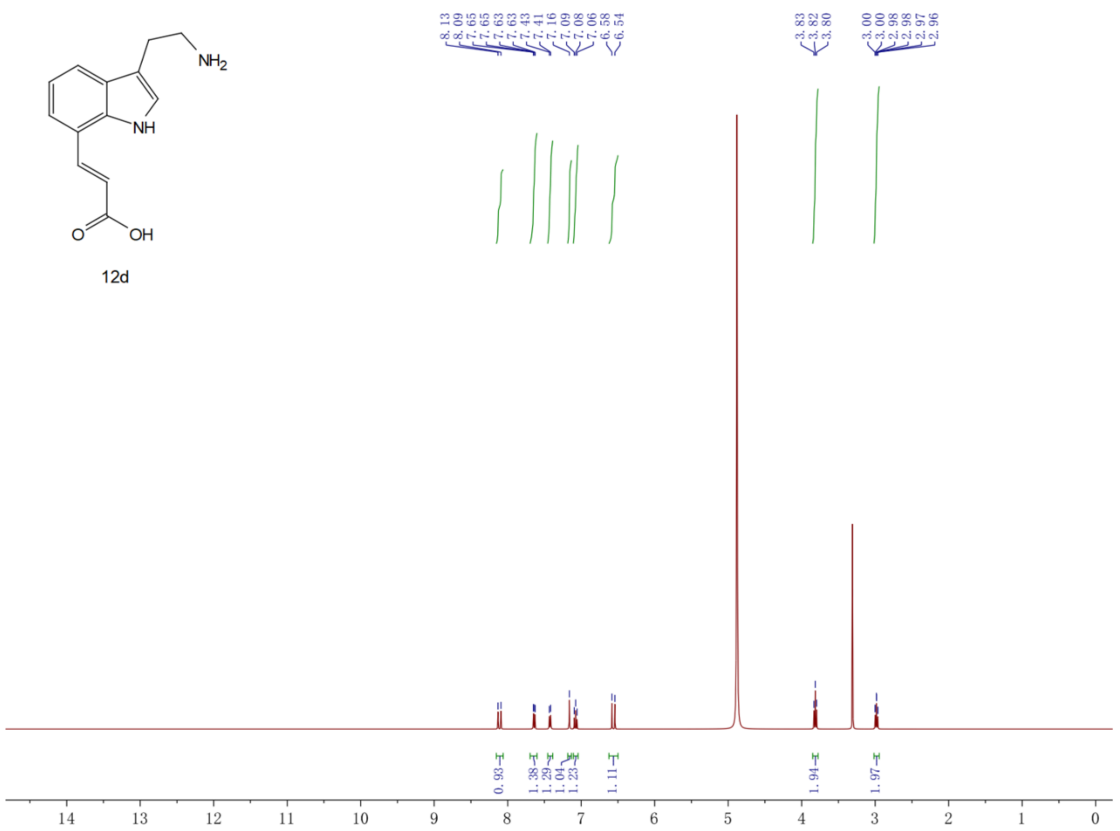


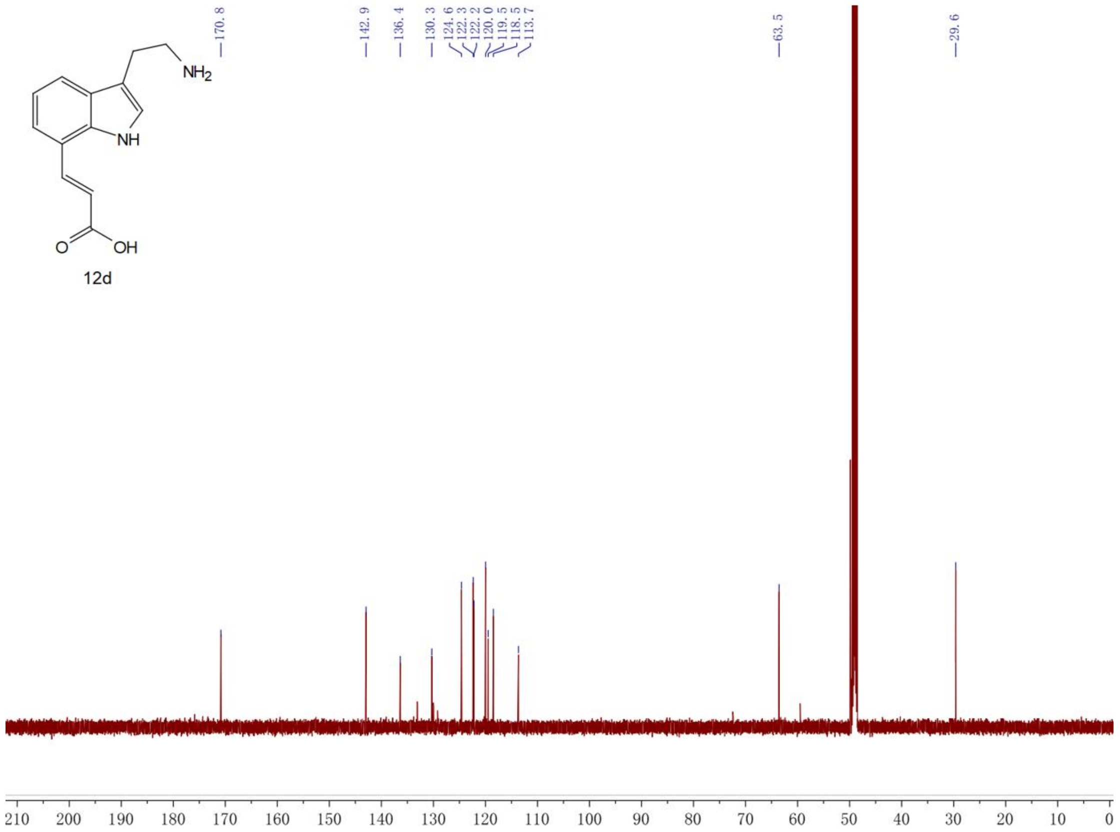


**Figure S61.** $^1$H NMR and $^{13}$C NMR of (*E*)- 3-(3-(2-aminoethyl)-1H-indol-7-yl)acrylic acid (**12d**) recorded in $CD_3OD$.

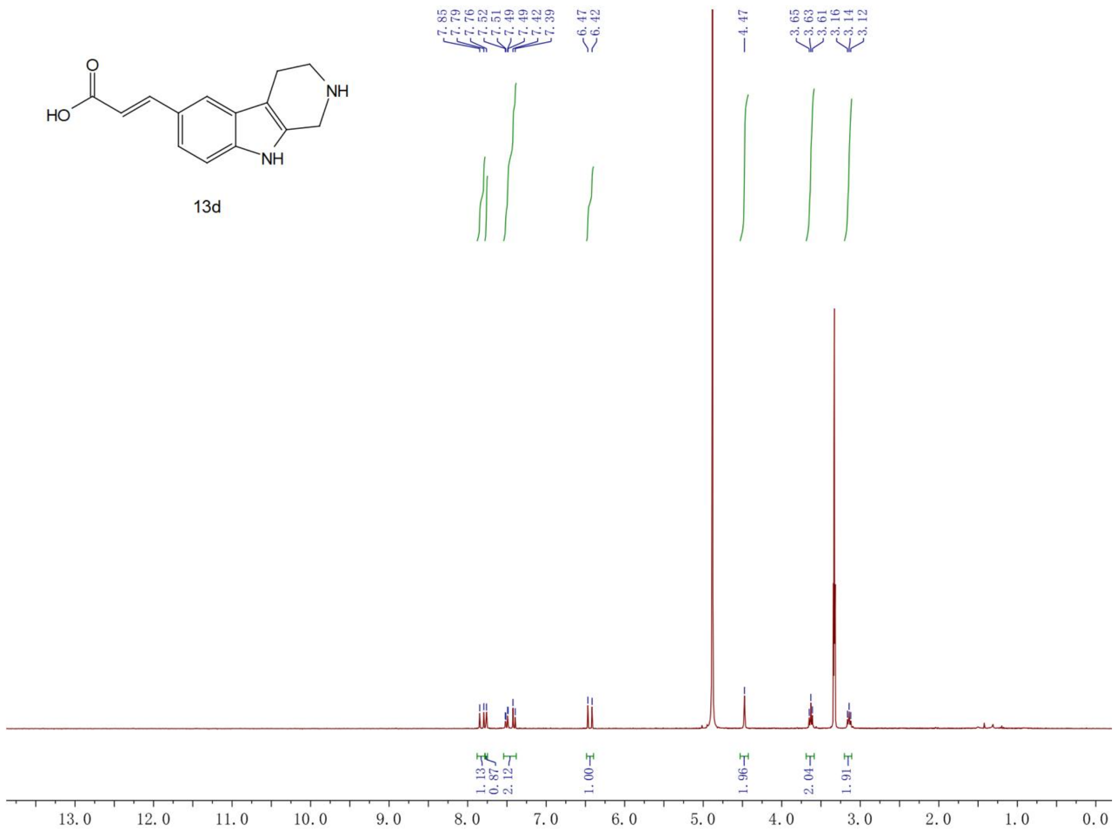

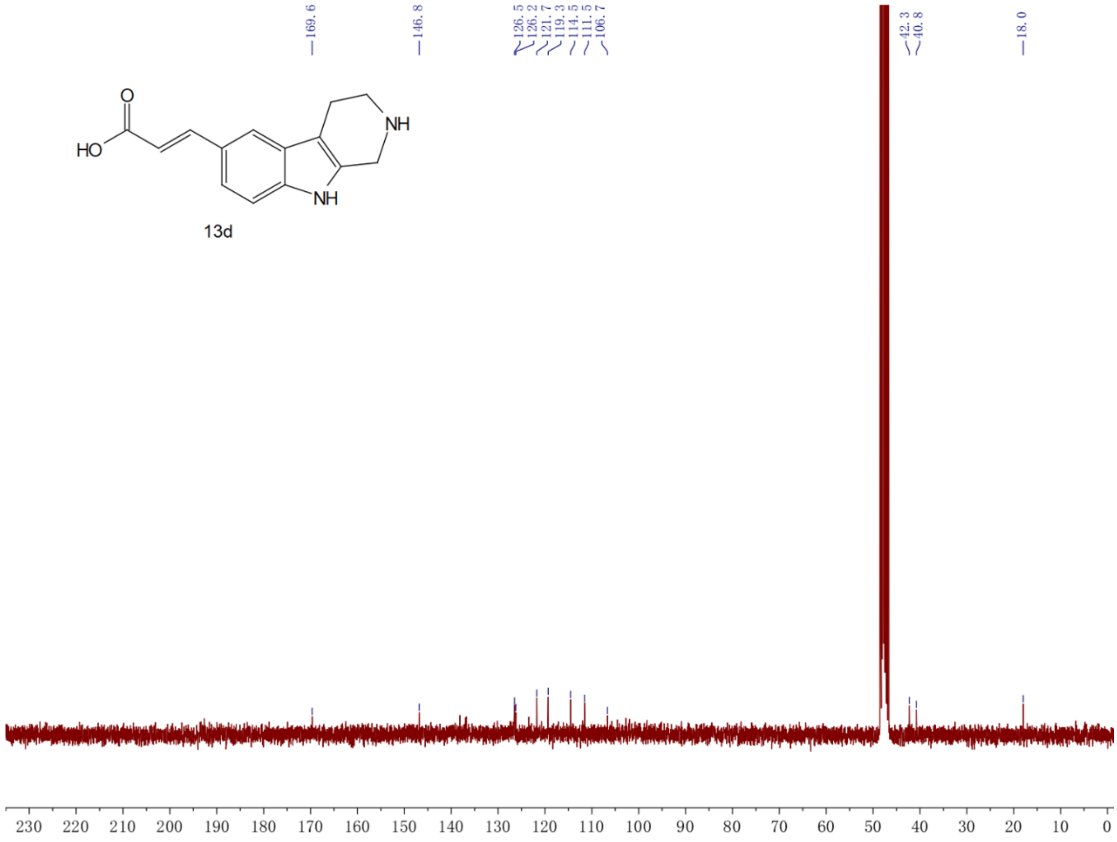

**Figure S62.** $^{1}H$ NMR and $^{13}C$ NMR of (*E*)- 3-(2,3,4,9-tetrahydro-1H-pyrido[3,4-b]indol-6-yl)acrylic acid (**13d**) recorded in $CD_3OD$.

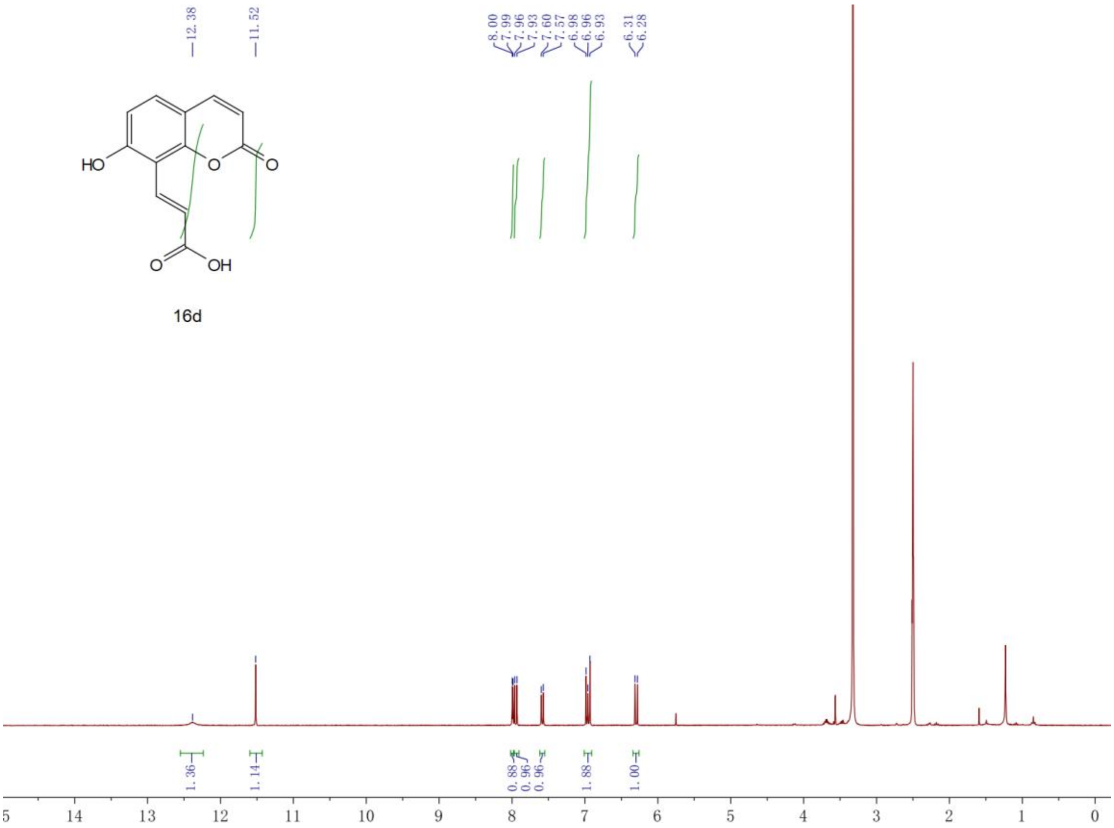


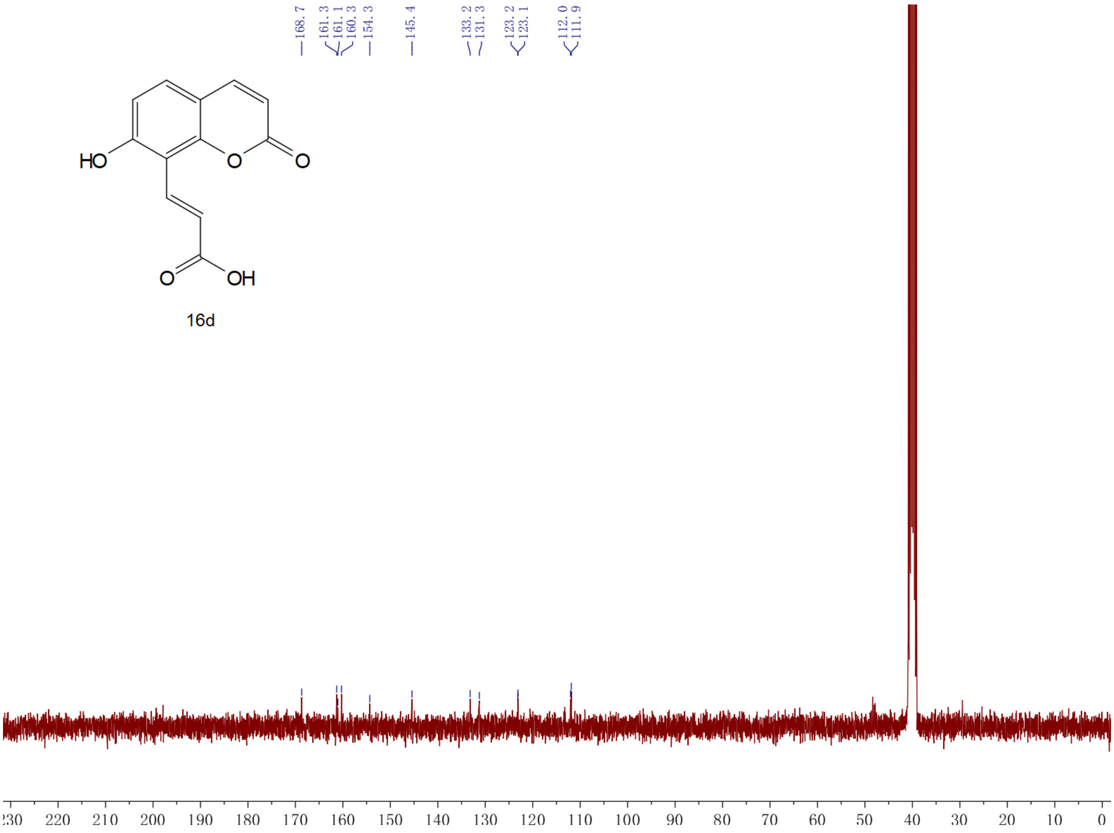


**Figure S63.** $^{1}$H NMR and $^{13}$C NMR of (*E*)- 3-(7-hydroxy-2-oxo-2H-chromen-8-yl)acrylic acid (**16d**) recorded in DMSO-$d_6$.

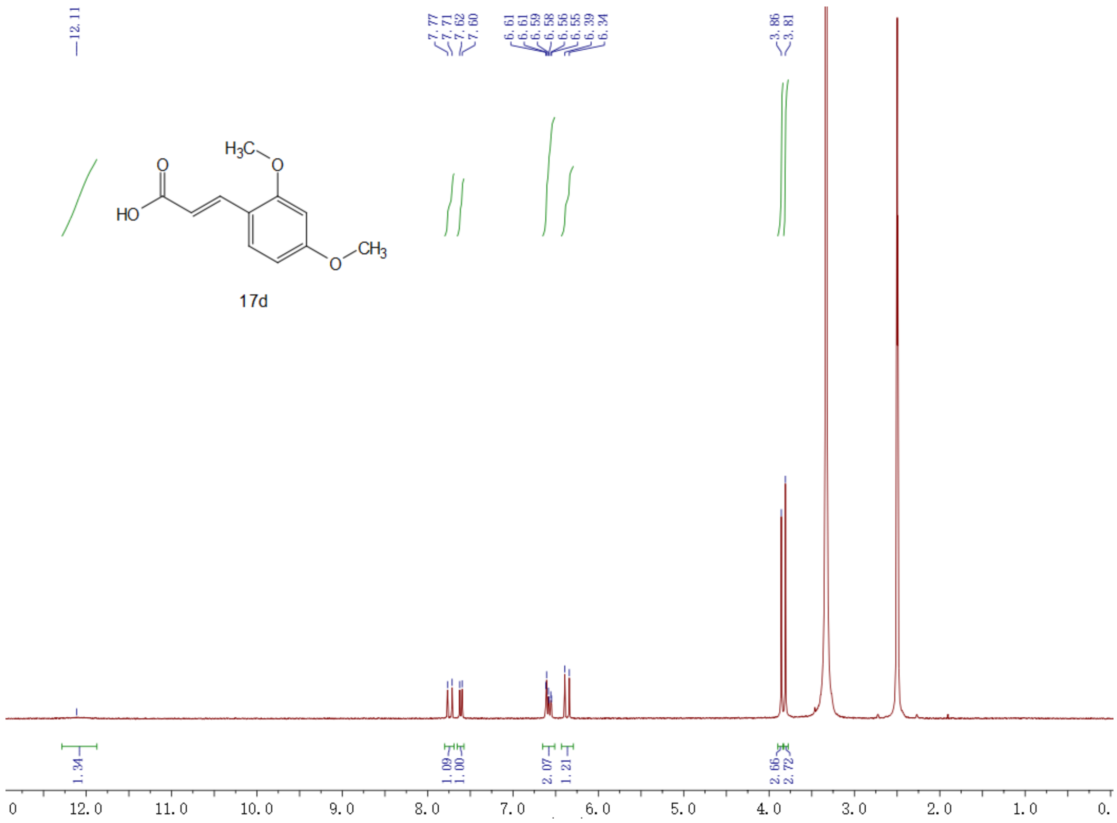

**Figure S64.** $^1$H NMR of (*E*)- 3-(2,4-dimethoxyphenyl)acrylic acid (**17d**) recorded in DMSO-$d_6$.

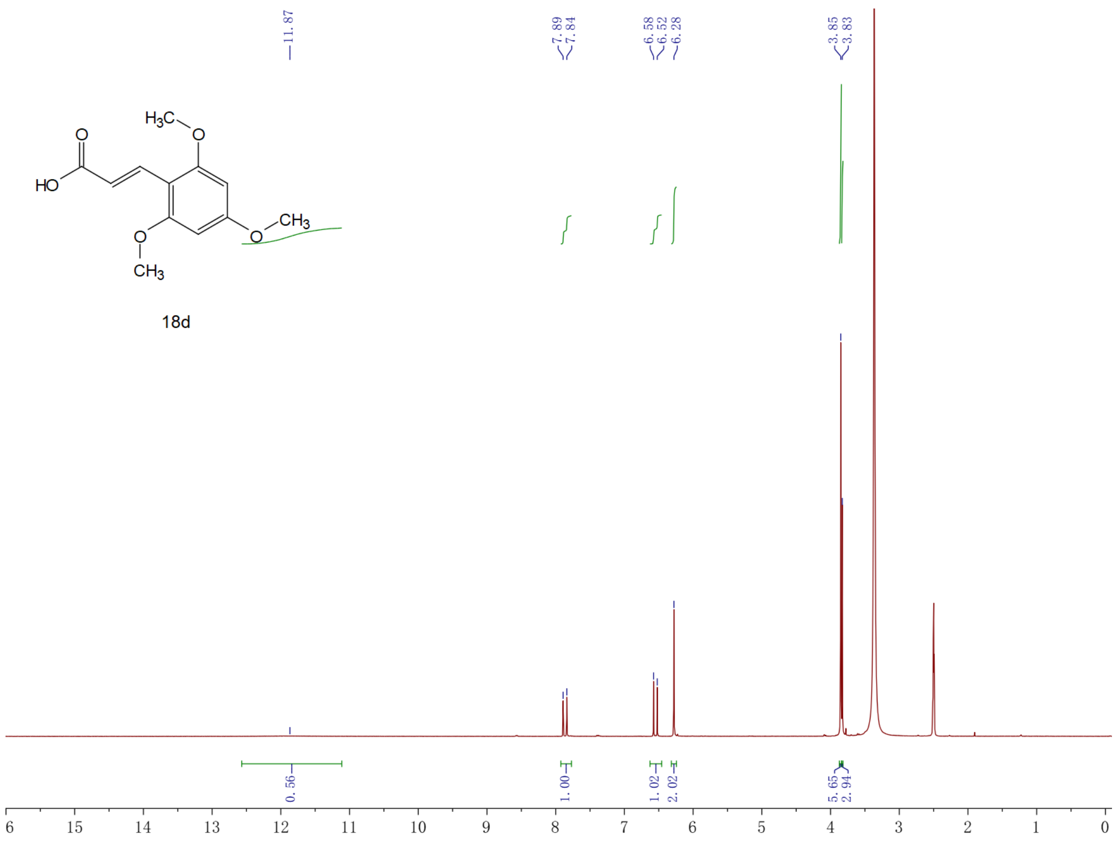


**Figure S65.** $^1$H NMR of (*E*)- 3-(2,4,6-trimethoxyphenyl)acrylic acid (**18d**) recorded in DMSO-$d_6$.

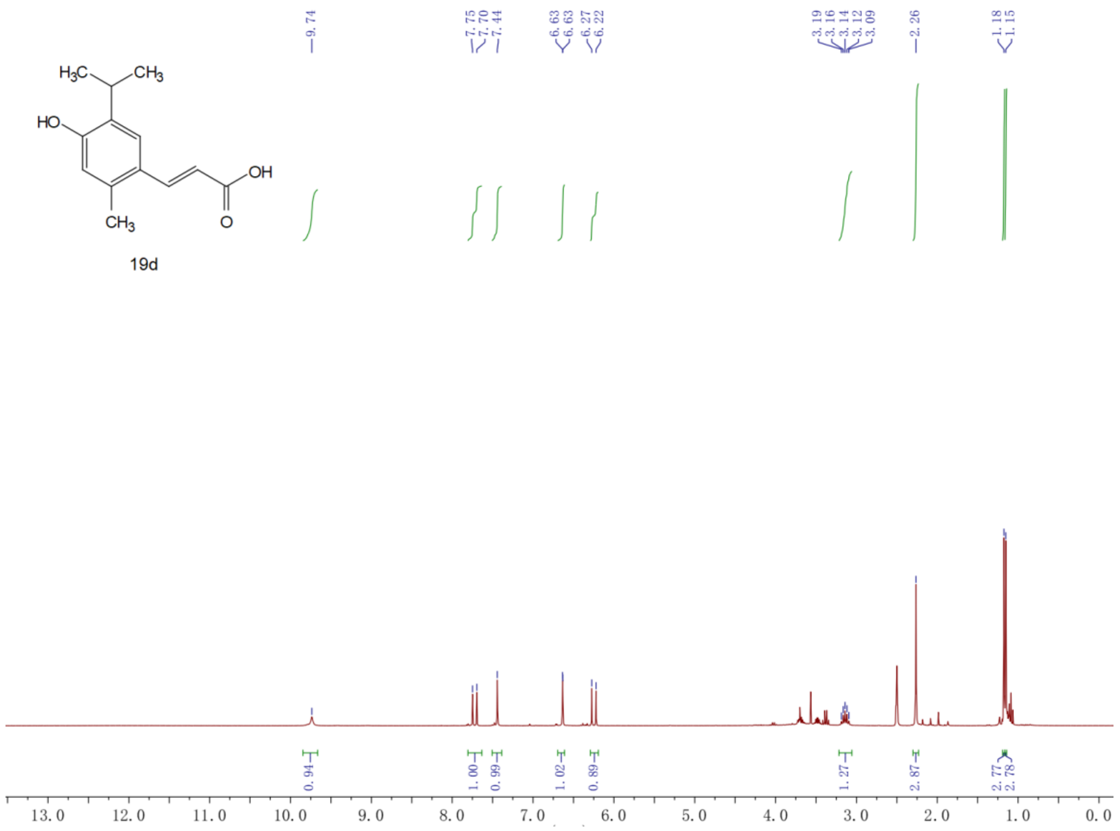


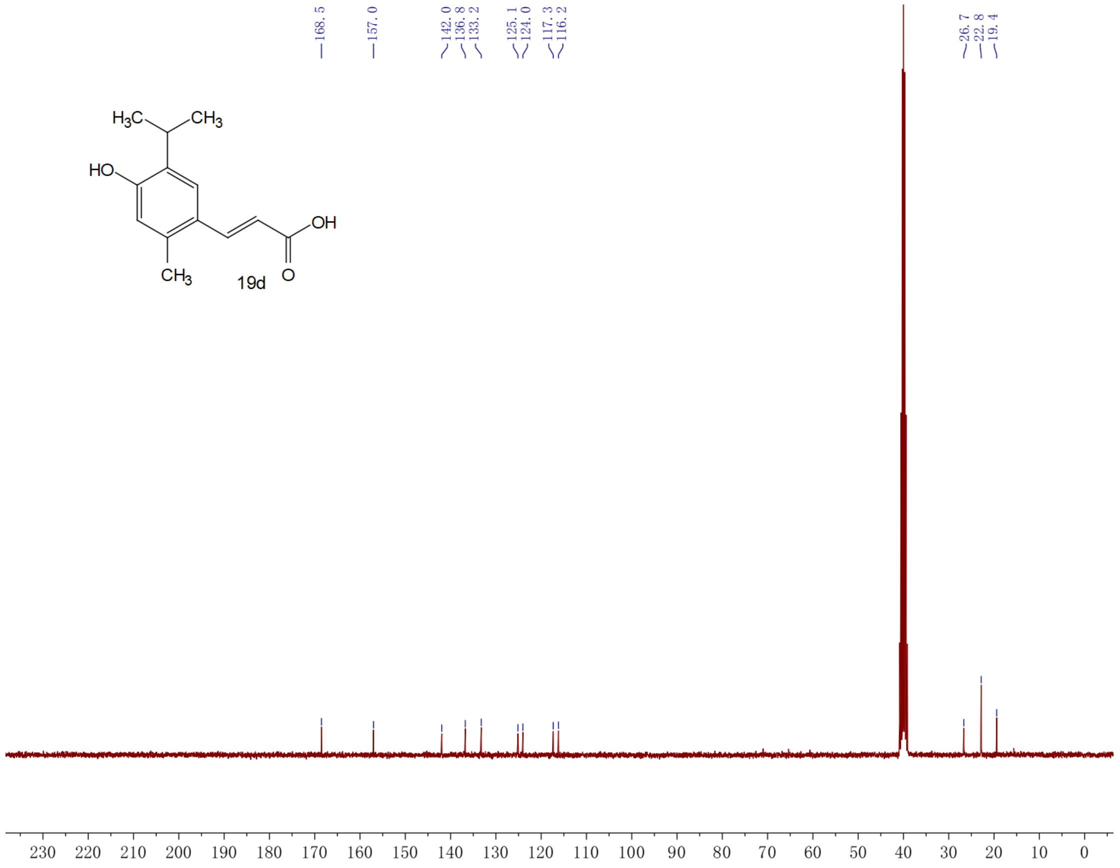


**Figure S66.** $^{1}H$ NMR and $^{13}C$ NMR of (*E*)-3-(4-hydroxy-5-isopropyl-2-methylphenyl)acrylic acid (**19d**) recorded in DMSO-$d_6$.

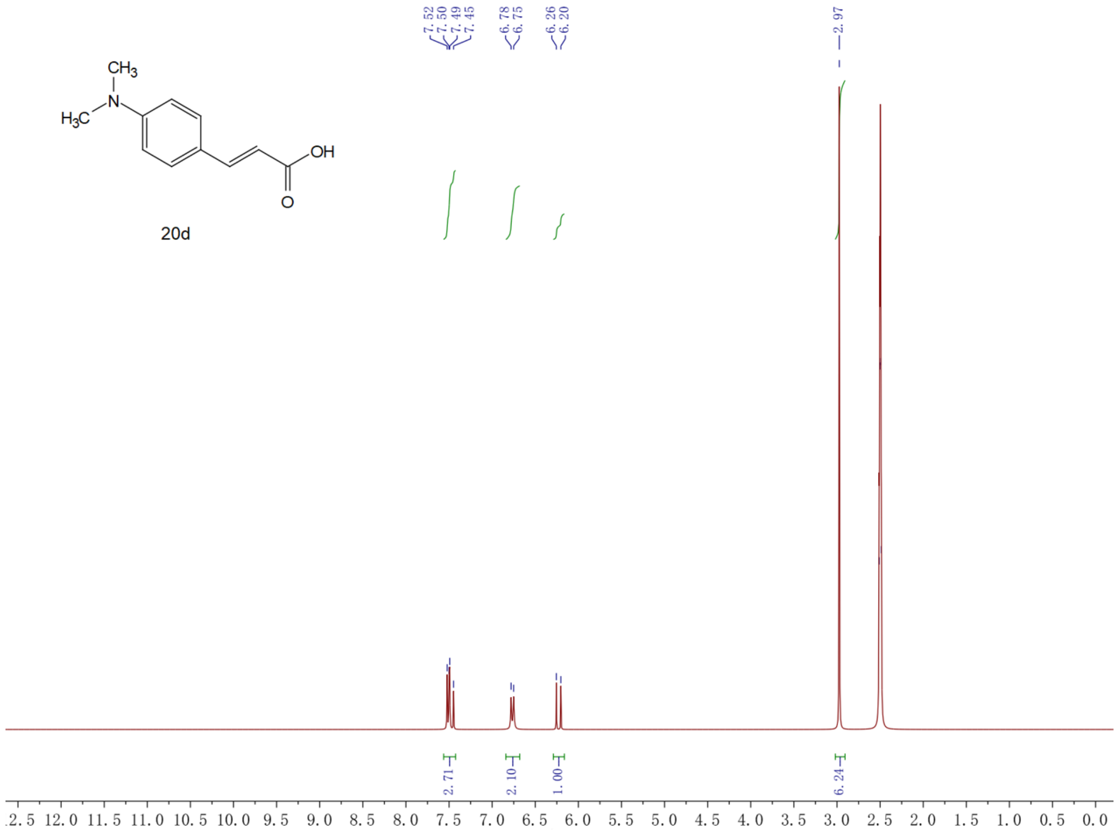


**Figure S67.** $^{1}$H NMR of *p*-dimethylaminocinnamic acid (**20d**) recorded in DMSO-$d_6$.

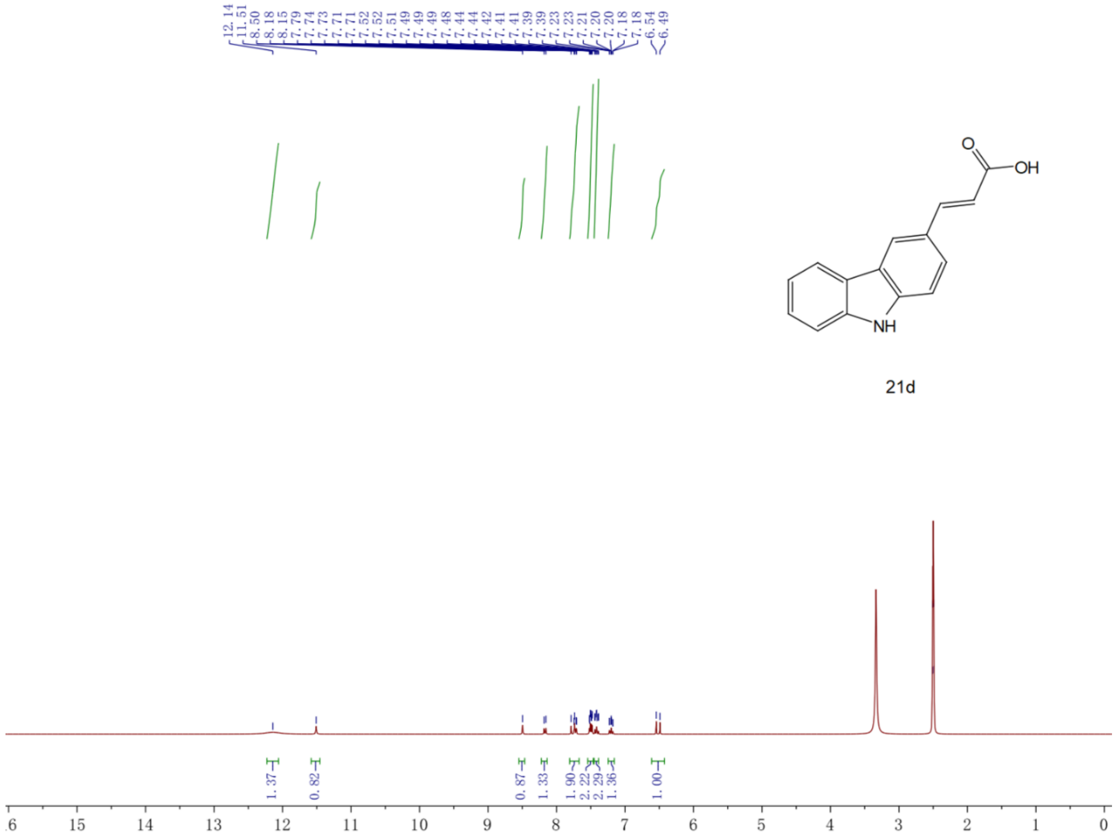


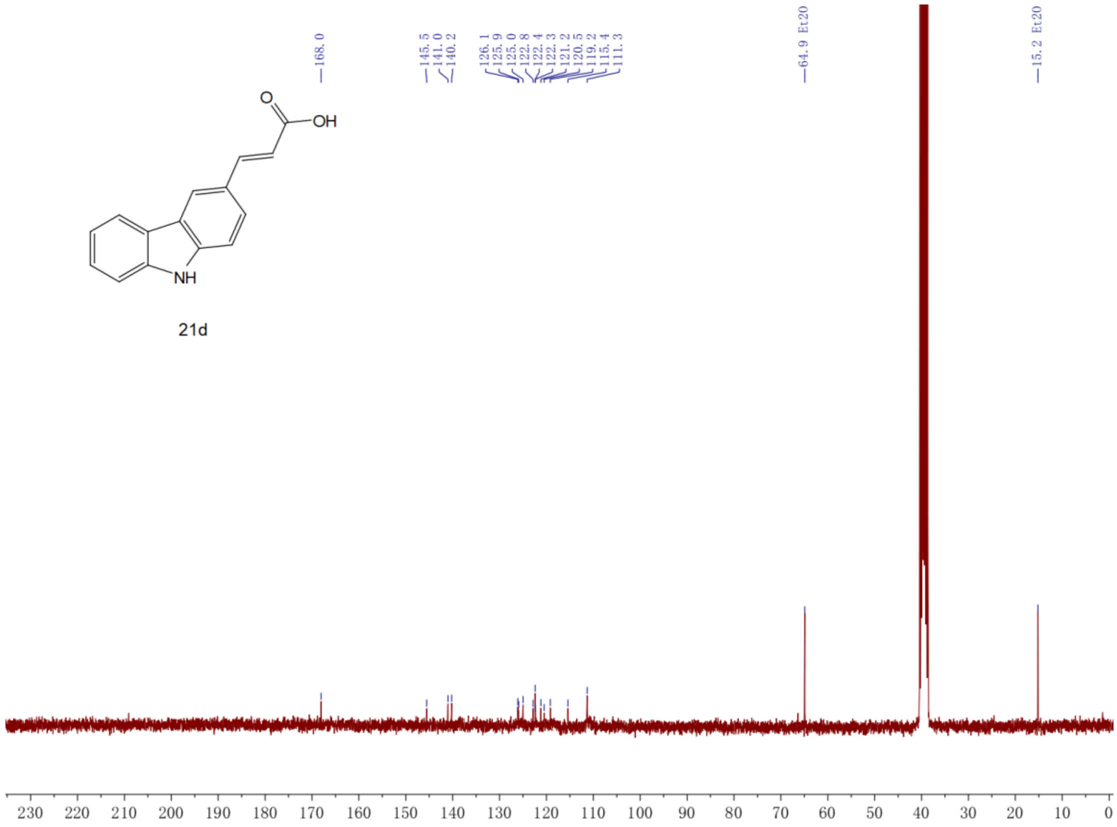


**Figure S68.** $^{1}$H NMR and $^{13}$C NMR of (*E*)- 3-(9H-carbazol-3-yl)acrylic acid (**21d**) recorded in DMSO-*d*$_6$.

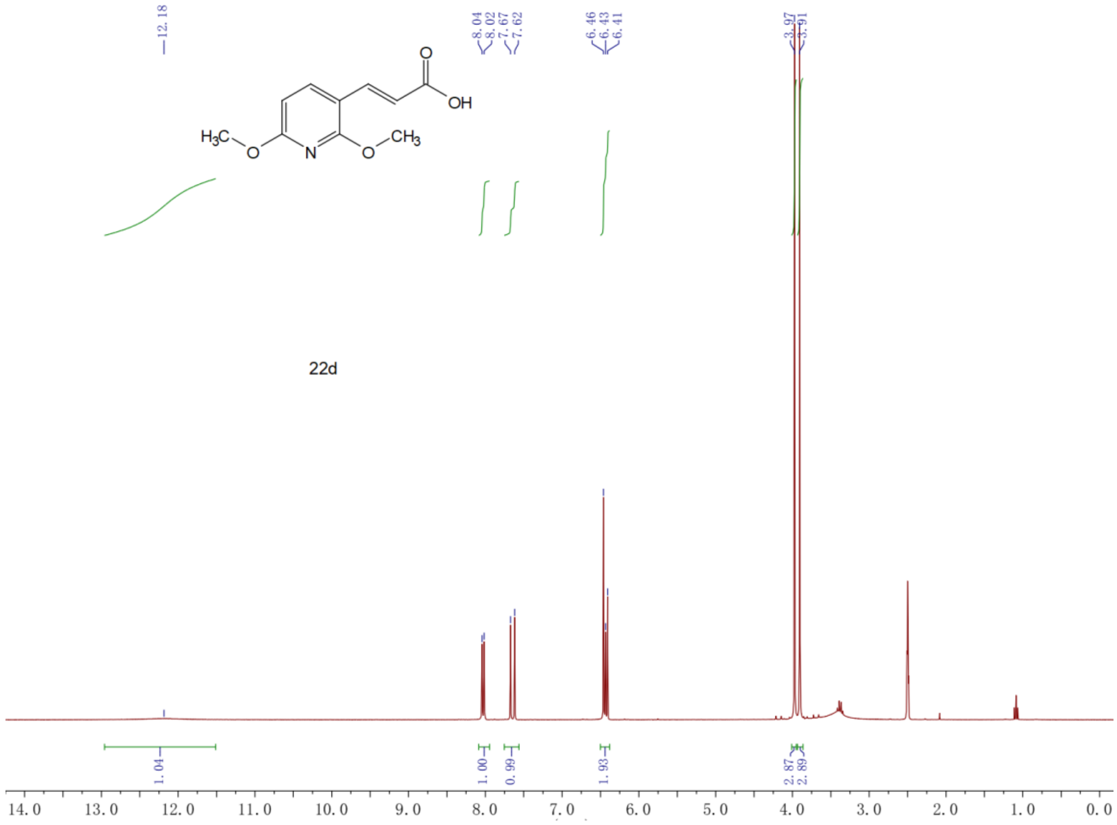


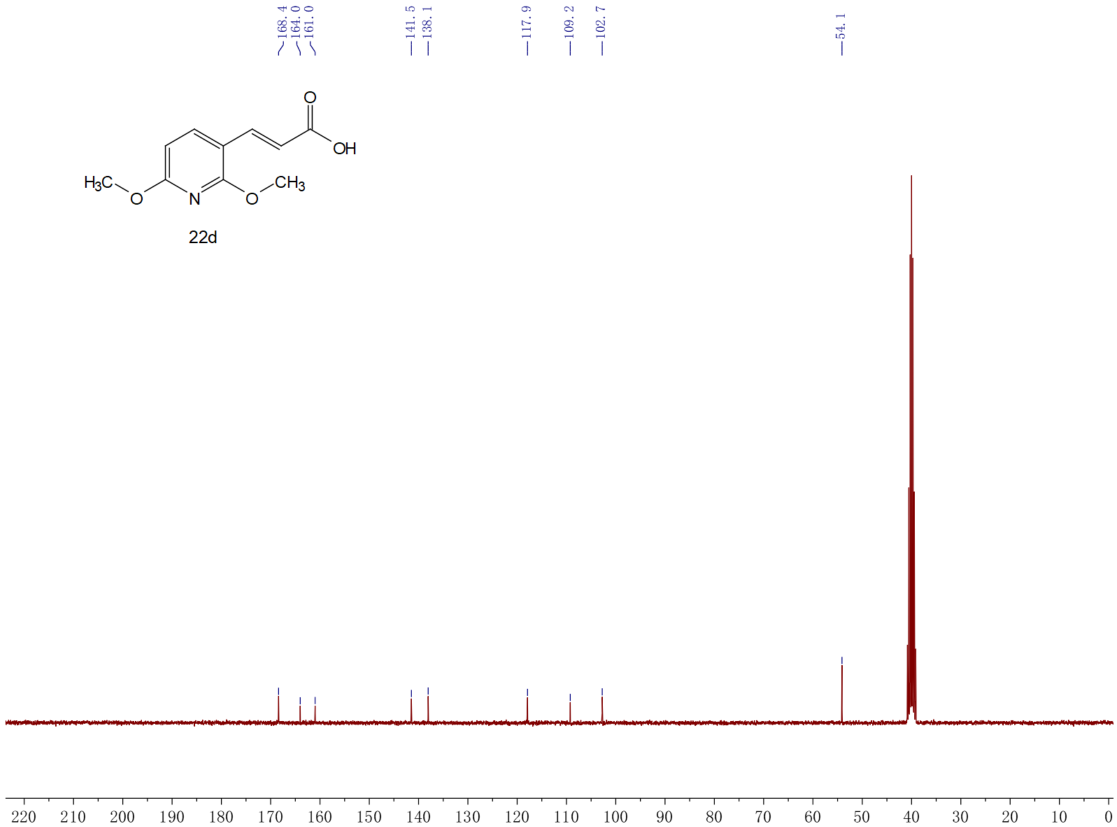


**Figure S69.** $^{1}$H NMR and $^{13}$C NMR of (*E*)- 3-(2,6-dimethoxypyridin-3-yl)acrylic acid (**22d**) recorded in DMSO-$d_6$.

# 6. References

[1] E. J. Craven, J. Latham, S. A. Shepherd, I. Khan, A. Diaz-Rodriguez, M. F. Greaney, J. Micklefield, *Nat. Catal.* **2021**, *4*, 385-394.

[2] C. Pubill-Ulldemolins, S. V. Sharma, C. Cartmell, J. Zhao, P. Cárdenas, R. J. M. Goss, *Chem. Eur. J.* **2019**, *25*, 10866-10875.

[3] Y. Xu, D. Hu, H. Zheng, D. Mei, Z. Gao, *Tetrahedron* **2019**, *75*, 130539.

[4] B. R. K. Menon, E. Brandenburger, H. H. Sharif, U. Klemstein, S. A. Shepherd, M. F. Greaney, J. Micklefield, *Angew. Chem. Int. Ed.* **2017**, *56*, 11841-11845.

[5] N. C. Ganguly, P. De, S. Dutta, *Synthesis* **2005**, *7*, 1103-1108.

[6] S. K. Chaudhuri, S. Roy, S. Bhar, *Beilstein J. Org. Chem.* **2012**, *8*, 323-329.

[7] A. Frank, C. J. Seel, M. Groll, T. Gulder, *ChemBioChem* **2016**, *17*, 2028-2032.

[8] S. Song, X. Sun, X. Li, Y. Yuan, N. Jiao, *Org. Lett.* **2015**, *17*, 2886-2889.

[9] C. J. Seel, A. Králík, M. Hacker, A. Frank, B. König, T. Gulder, *ChemCatChem* **2018**, *10*, 3960-3963.

[10] G. Wang, Z. Li, C. Li, S. Zhang, *J. Catal.* **2018**, *368*, 228-236.

[11] S. G. Davies, A. W. Mulvaney, A. J. Russell, A. D. Smith, *Tetrahedron: Asymmetry* **2007**, *18*, 1554-1566.

[12] G.-R. Peh, E. A. B. Kantchev, C. Zhang, J. Y. Ying, *Org. Biomol. Chem.* **2009**, *7*, 2110-2119.

[13] K. S. Yoo, C. H. Yoon, K. W. Jung, *J. Am. Chem. Soc.* **2006**, *128*, 16384-16393.

[14] C. Luadthong, A. Tachaprutinun, S. P. Wanichwecharungruang, *Eur. Polym. J.* **2008**, *44*, 1285-1295.

[15] Y. Bai, X. He, Y. Bai, Y. Sun, Z. Zhao, X. Chen, B. Li, J. Xie, Y. Li, P. Jia, X. Meng, Y. Zhao, Y. Ding, C. Xiao, S. Wang, J. Yu, S. Liao, Y. Zhang, Z. Zhu, Q. Zhang, Y. Zhao, F. Qin, Y. Zhang, X. Wei, M. Zeng, J. Liang, Y. Cuan, G. Shan, T.-P. Fan, B. Wu, X. Zheng, *Eur. J. Med. Chem.* **2019**, *183*, 111650.

[16] L.-Y. He, M. Schulz-Senft, B. Thiedemann, J. Linshoeft, P. J. Gates, A. Staubitz, *Eur. J. Org. Chem.* **2015**, *2015*, 2498-2502.
